# Multicellular architectures for high-rate solar evaporation with spatial salt crystallization under high-salinity

**DOI:** 10.1038/s41467-026-75913-w

**Published:** 2026-07-22

**Authors:** Josue Yaedalm Son, Yunsan Choi, Xitong Liu, Run Hu, Hyejeong Kim

**Affiliations:** 1 School of Mechanical Engineering, Korea University, Seoul, Republic of Korea; 2 Department of Civil and Environmental Engineering, The George Washington University, Washington, DC USA; 3 School of Energy and Power Engineering, Huazhong University of Science and Technology, Wuhan, China; 4 Max Planck Institute for Dynamics and Self-Organization, Göttingen, Germany

**Keywords:** Engineering, Pollution remediation

## Abstract

Solar-driven evaporation offers decentralized freshwater production and brine management, yet remains limited by inefficient water transport, thermal losses and salt fouling arising from insufficient understanding of geometry–transport–performance relationships. Here, 3D-printed multicellular solar evaporators with systematically varied lattice unit cells are developed to clarify these relationships, identifying liquid–solid contact perimeter, porosity and thermal interface area as key geometric parameters governing capillary water delivery and heat transfer. Guided by these, an optimized FBCC-+_5 evaporator achieves a high evaporation rate of 6.90 kg m^−2^ h^−1^ and sustains zero-liquid-discharge operation. A hybrid multicellular architecture combining high- and low-evaporation-rate unit cells further generates controlled evaporation gradients, thereby localizing salt crystallization, enabling stable operation for 5.5 days with 97.49% salt recovery under 20 wt% NaCl. Outdoor desalination yields up to 47.7 kg m^−2^ day^−1^ of freshwater. This work establishes unit-cell and macro-scale geometry as programmable design parameters for scalable and durable solar desalination.

## Abstract (web summary)

This study introduces a structure-guided strategy for solar desalination, showing how unit-cell and macro-scale geometry can serve as programmable design parameters to control water flow, heat transport, and salt crystallization for stable desalination.

## Introduction

Freshwater scarcity has intensified over recent decades due to population growth, rapid industrialization, and accelerating environmental degradation, positioning access to potable water as one of the most urgent global challenges^1,2^. Although oceans cover nearly 70% of Earth’s surface, only ~ 2.5% of global water exists as freshwater, and an even smaller fraction is readily accessible for human consumption^3,4^. Conventional water treatment technologies are widely implemented, yet many rely on energy-intensive processes, complex infrastructure, and costly operations^5,6^. Among these, reverse osmosis (RO) currently dominates the seawater desalination market but remains constrained by substantial energy demand, significant capital and operating expenses, and complex maintenance requirements, including extensive pretreatment and frequent membrane cleaning^7^. These challenges are further exacerbated in decentralized or resource-limited settings. Moreover, RO systems face fundamental challenges when treating highly saline brine streams, as escalating osmotic pressures and accelerated membrane fouling significantly reduce operational efficiency and system lifetime, making zero-liquid-discharge (ZLD) operation difficult to realize in practice^8^. Consequently, the treatment and management of concentrated desalination brines remain a critical sustainability bottleneck for large-scale desalination systems.

Solar-driven water treatment approaches, including photocatalysis and solar evaporation, have attracted growing interest due to their low operating cost, material stability, non-toxicity, and reusability^9–11^. In particular, solar-driven interfacial steam generation has shifted evaporation strategies from energy-intensive bulk heating to localized photothermal conversion at the air–water interface, enabling evaporation efficiencies approaching ~ 90% by suppressing radiative, convective, and conductive losses^12^. Despite these advances, practical implementation remains limited by optical reflection losses, thermal leakage, insufficient water supply under high flux, and salt or pollutant accumulation that leads to fouling and performance degradation^13^.

Achieving both high efficiency and long-term stability in solar-driven evaporative desalination requires precise control over two coupled factors: thermal management and water management^14^. Effective thermal management demands high solar absorptance, efficient photothermal conversion, and strong thermal insulation, while effective water management requires continuous liquid delivery to the evaporative interface and robust salt-rejection capability during prolonged operation^15^. To address these requirements, recent research has shifted from material-only optimization toward deliberate structural engineering of solar evaporators^16^. Structural geometry critically governs interfacial transport phenomena, including capillary-driven water supply, thermal localization, and salt crystallization behavior^17–19^. However, most reported evaporators rely on random porous structures, making it difficult to systematically correlate structural parameters with heat and mass transport. As a result, prior studies often addressed individual factors in isolation, limiting the identification of architectures that can simultaneously satisfy multiple performance criteria.

Additive manufacturing, particularly high-resolution 3D printing, provides an enabling platform for this approach by offering programmable control over lattice topology, hierarchy, curvature, and interfacial configuration^20^. Unlike stochastic porous media, 3D-printed periodic architectures allow systematic modulation of porosity, tortuosity, heat-transfer pathways, and capillary dynamics, offering a rational route to establish geometry–transport–performance relationships^21,22^. Although recent demonstrations, such as bridge-arch architectures^23^, biomimetic evaporators^24^, and concave optical concentrators^25^, highlight the promise of structural design, a unified framework linking geometric configuration to interfacial transport physics and system-level evaporation performance remains largely unexplored.

**Fig. 1:**
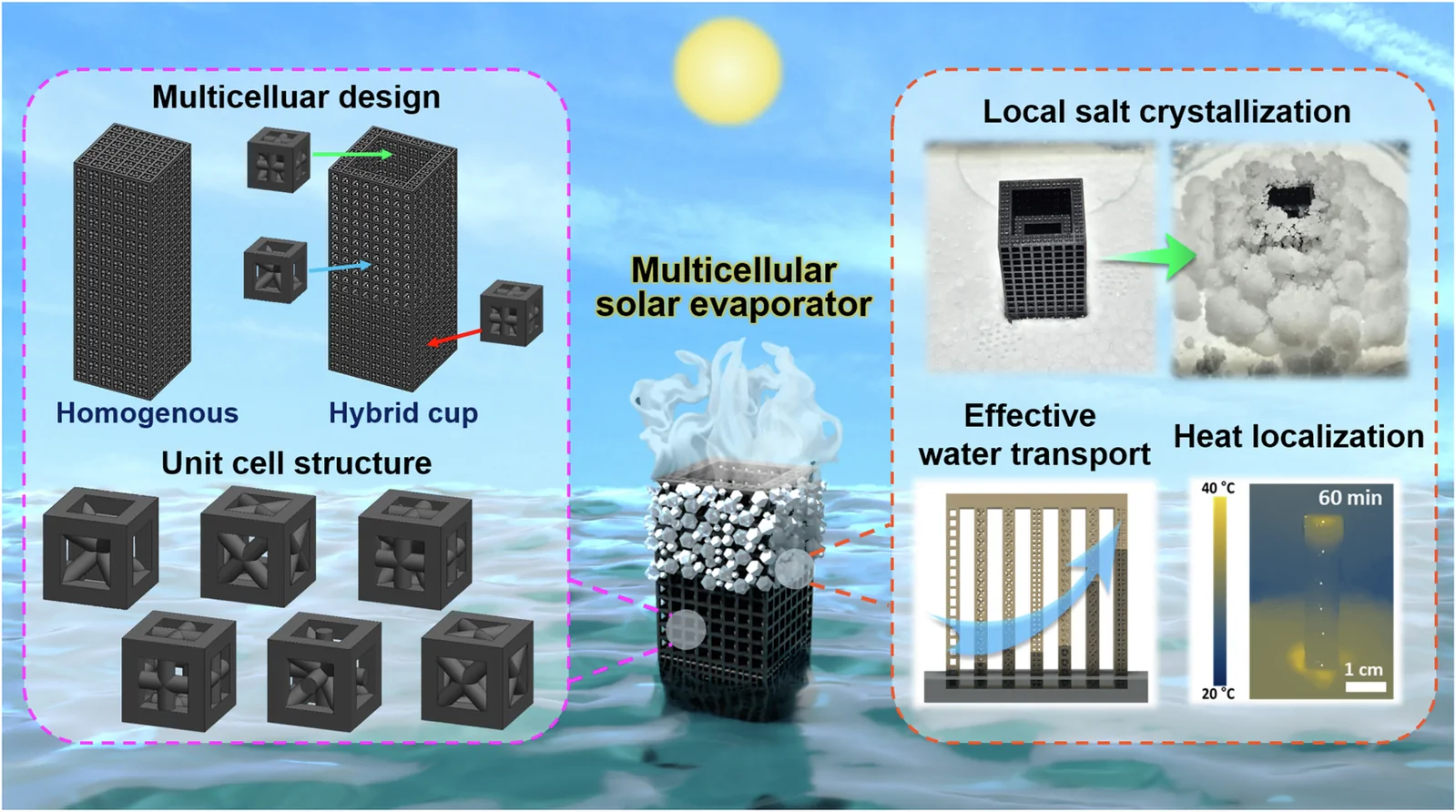
Multicellular solar evaporator concept. Schematic illustration of the overall design and working principle of the multicellular solar evaporator.

In this study, a series of 3D-printed multicellular evaporators with systematically varied lattice geometries was developed to investigate how geometric design governs water transport, heat transfer, and salt behavior during evaporation (Fig. 1). By decoupling structural effects from material properties, this study demonstrates that unit-cell geometry is a decisive factor controlling water transport and solid–liquid thermal coupling, which directly determine evaporation performance and stability. The results reveal that liquid–solid contact perimeter, porosity, and interfacial geometry collectively regulate evaporation dynamics. Building on these insights, height-modulated multicellular architectures are introduced to activate lateral evaporation and enable geometry-assisted harvesting of ambient thermal energy. Moreover, hybrid multicellular designs that integrate unit cells with distinct evaporation characteristics are shown to induce controlled evaporation rate gradients, allowing passive spatial localization of salt crystallization away from the primary evaporation surfaces. This geometry-driven salt management strategy enables sustained operation under high salinity without active intervention. Finally, the effectiveness and robustness of this structure-first design paradigm are validated under outdoor conditions, demonstrating its potential for scalable, durable, and energy-efficient solar desalination and water purification.

## Results

### Fabrication and physicochemical characteristics

**Fig. 2:**
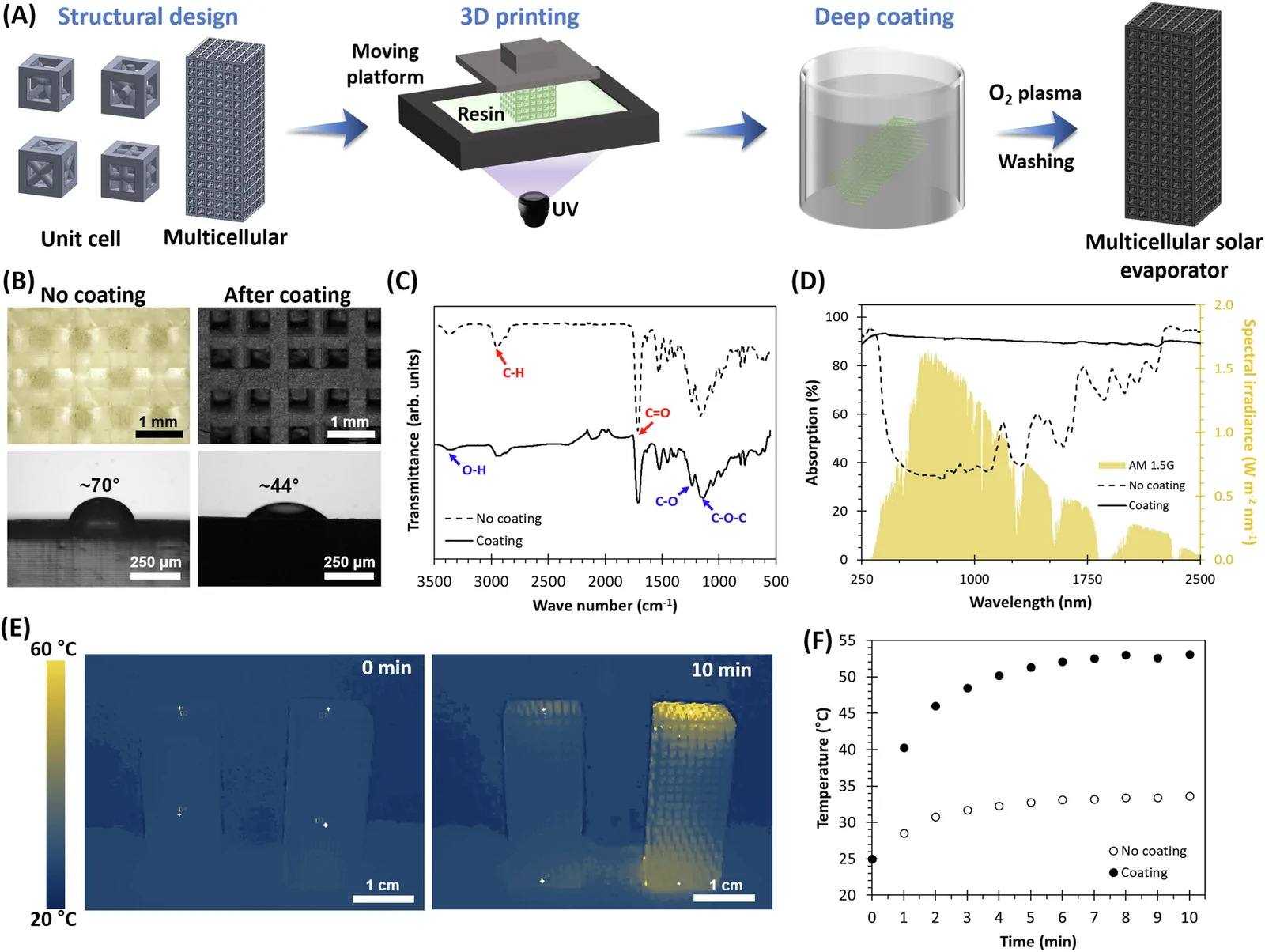
Fabrication and surface properties of MSE. **A** Schematic illustration of the fabrication process of the MSE. **B** Optical images and corresponding contact angles of the multicellular structure before and after graphite coating, confirming the transition to a hydrophilic surface. **C** FTIR spectra of the samples before and after coating. **D** UV–Vis–NIR absorption spectra of the multicellular structures, compared with the air mass 1.5 global (AM 1.5 G) solar spectrum. **E** Infrared thermal images of uncoated and coated multicellular structures under dry-state 1 sun irradiation. **F** Temperature evolution of uncoated and coated multicellular structures under 1 sun irradiation.

Various unit cell types were systematically designed and assembled into periodic multicellular structures. Following photothermal functionalization, these structures were referred to as multicellular solar evaporators (MSEs). The MSEs were fabricated via high-resolution 3D printing to form ordered lattice architectures featuring interconnected water pathways and a large effective surface area for light absorption (Fig. 2A). The printed structures were produced from an acrylate-based photopolymer resin and subsequently functionalized with a conformal graphite coating through a dip-coating process to impart photothermal activity. This method enables uniform surface coverage of complex three-dimensional lattice geometries, including internal pores and thin struts, without requiring line-of-sight deposition or inducing structural blockage^26,27^. The graphite layer adhered to the polymer scaffold primarily via van der Waals interactions and remained mechanically stable throughout all experiments, with no observable particle detachment or coating degradation (Supplementary Fig. 1)^28^. The X-ray diffraction (XRD) pattern of the coated MSE displays a (002) reflection of graphitic carbon at ~ 26.5° alongside an amorphous halo at ~ 19° from the polymer resin matrix, confirming successful incorporation of graphitic domains (Supplementary Fig. 2)^29^. Coating uniformity was further assessed by scanning electron microscopy (Supplementary Fig. 3), revealing a conformal and homogeneous graphite layer on the 3D-printed framework. The average frame thickness increased from 400 ± 2 μm (pristine) to 413 ± 1 μm after coating, corresponding to an approximate graphite layer thickness of 6.5 μm. Optical imaging and static contact angle measurements confirmed that the combined graphite coating and oxygen plasma treatment effectively transformed the initially hydrophobic resin surface into a hydrophilic interface (Fig. 2B). This surface modification enabled rapid wetting and continuous capillary water transport, which are essential for sustained interfacial evaporation. The graphite-coated surface alone exhibited a slightly higher contact angle (77.6 ± 7.4°) than that of the pristine resin (70.6 ± 8.4°), indicating that graphite itself did not enhance wettability. In contrast, oxygen plasma treatment reduced the contact angle to 44.7 ± 8.8°, which remained stable even after 6 months (46.6 ± 5.0°), demonstrating long-term hydrophilicity arising from permanent surface modification of the graphite layer (Supplementary Fig. 4). This behavior was further examined by Fourier-transform infrared spectroscopy (FTIR) (Fig. 2C). The pristine resin exhibited characteristic vibrational features of acrylate polymers, including aliphatic C–H stretching near ~ 2956 cm^−1^, a strong carbonyl (C = O) band at ~ 1714 cm^−1^, and C–O/C–O–C stretching modes in the 1300–1100 cm^−1^ region^30,31^. After graphite coating followed by oxygen plasma treatment and water rinsing, the O–H stretching band around ~ 3300 cm^−1^ became broader, and the C–O and C–O–C bands at 1236–1135 cm^−1^ exhibited increased band broadening and shoulder formation. These spectral features are indicative of hydrogen-bonded hydroxyl and ether-type oxygen functionalities, consistent with partial surface oxidation of the graphite layer rather than chemical modification of the underlying polymer matrix^32,33^. In addition, most resin-related C–H and C–O bands became attenuated due to the strong IR absorption by the graphite layer^34^. Collectively, these results confirm the formation of a partially oxidized, hydrophilic graphite surface that is responsible for the enhanced wettability observed after plasma treatment.

Broadband optical absorption is critical for efficient solar energy utilization. After graphite coating, the MSEs exhibited markedly enhanced absorption across the entire solar spectrum, from 250 to 2500 nm, confirming effective broadband light harvesting (Fig. 2D and Supplementary Fig. 5). Correspondingly, the photothermal conversion performance was significantly improved. Under 1 sun illumination (1000 W m^−2^), the surface temperature of the graphite-coated MSE increased rapidly from ambient temperature (25.0 °C) to 40.3 °C within 1 min and stabilized at 53.1 °C within 10 min. In contrast, the uncoated resin sample exhibited only a slight temperature increase of 8.6 °C under the same conditions (Fig. 2E, F), validating the improved photothermal conversion performance of the MSE after graphite coating.

### Influence of unit cell geometry on water transport

**Fig. 3:**
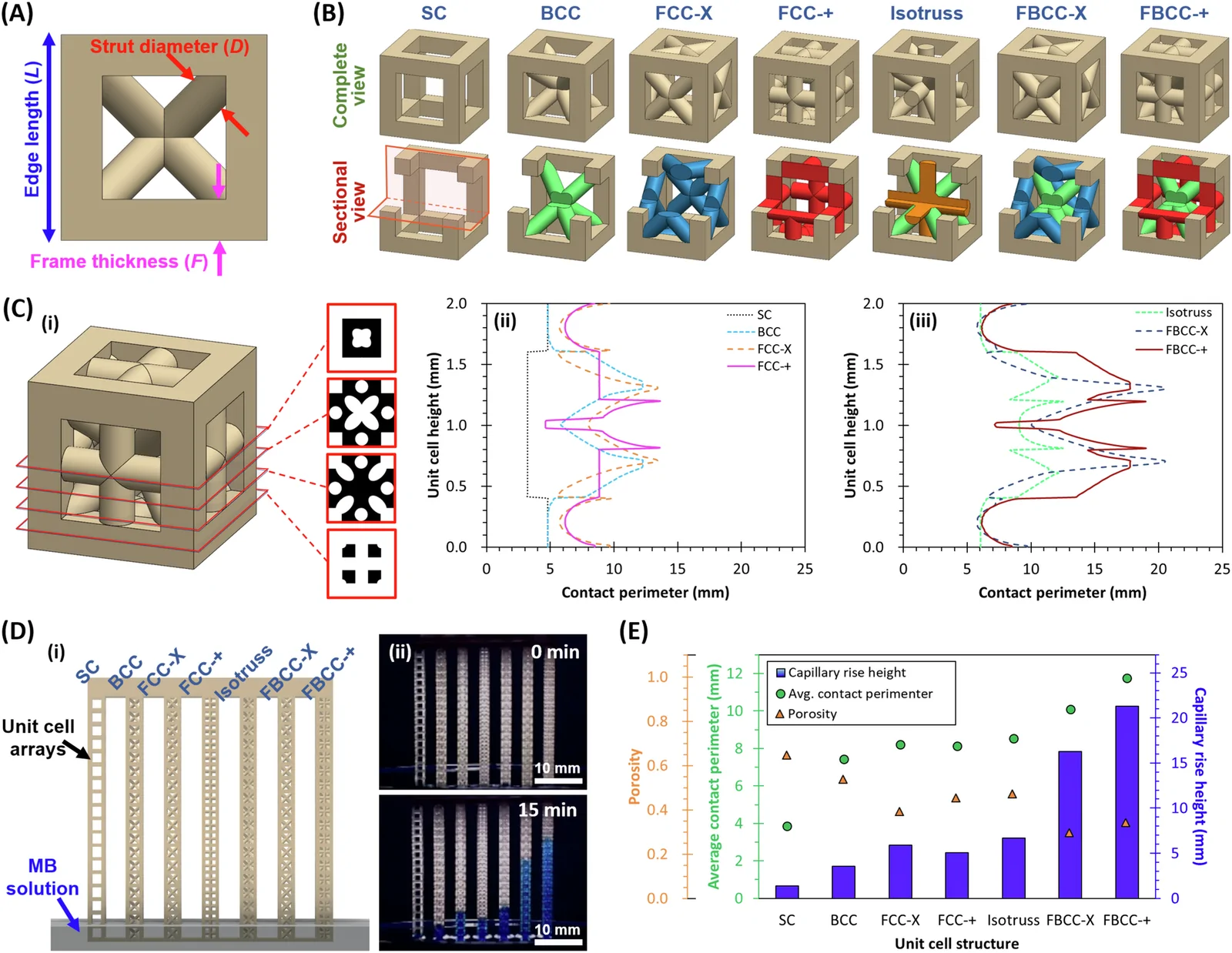
Effect of unit cell geometry on water transport. **A** Geometrical characteristics of the unit cell structures. **B** Complete and sectional views of each unit cell type, showing the internal architectures. **C** (i) Cross-sectional images of a unit cell illustrating the liquid–solid contact perimeter at various heights; (ii–iii) quantitative plots of the contact perimeter as a function of height within the unit cell. **D** Capillary rise behavior in single-column multicellular structures: (i) schematic representation of the experimental setup; (ii) time-dependent water rise images. **E** Comparative analysis of porosity, average contact perimeter, and maximum capillary rise across the different unit cell geometries.

Efficient water transport is important for sustained solar evaporation, as continuous liquid replenishment to the heated evaporation interface is required. To elucidate how unit cell geometry governs capillary-driven liquid transport, a systematic comparison of multiple 3D-printed lattice architectures was performed. The key geometrical parameters of each unit cell design are summarized in Fig. 3A. All architectures were fabricated with identical dimensional baselines, including a cylindrical strut diameter of 400 µm, an external frame thickness of 400 µm, and a unit cell size of 2 × 2 × 2 mm^3^. This ensured that differences in water transport arose solely from geometric configuration rather than dimensional variation.

Seven distinct unit cell geometries were designed and systematically investigated. Complete and sectional cutaway views of each architecture are presented in Fig. 3B, allowing direct visualization of both external and internal morphology. The simple cubic (SC) lattice consists of struts aligned orthogonally along the three principal axes, with nodes located exclusively at the cube corners. Introducing a body-centered node forms the body-centered cubic (BCC) lattice, in which diagonal struts connect the cube center to its corners. The face-centered cubic (FCC) architecture incorporates nodes at the center of each cube face, increasing the density of struts at the external surfaces. Depending on the strut orientation, FCC lattices with diagonal interconnections were denoted as FCC-X, whereas FCC lattices with struts extending from the center of the face frames were denoted as FCC-+. The isotruss unit cell features a body-centered node connected by struts extending toward both the face centers and the diagonal directions of the cubic frame. Finally, the face–body-centered cubic (FBCC) lattice was designed by integrating FCC and BCC unit cells, resulting in two variants, FBCC-X and FBCC-+, which are distinguished by the strut configuration at the face planes.

Capillary rise in porous structures is governed by the balance between cohesive forces within the liquid and adhesive interactions at the liquid–solid interface^35^. While classical capillary models assume a constant wetted boundary, this assumption does not hold for architected lattices in which the contact perimeter varies periodically along the vertical direction. Consequently, differences in capillary rise height and wicking kinetics among unit cell designs originate from geometry-dependent variations in available wetting perimeter and pore volume^36^.

For these architected structures, the adhesive force driving capillary rise can be expressed as^37^:

1 $${F}_{{{{\rm{adhesive}}}}}=\gamma \cos \theta P(h)$$

where *γ* is the surface tension, *θ* is the contact angle, and *P(h)* is the height-dependent liquid–solid contact perimeter. The opposing gravitational force acting on the liquid volume is given by refs. ^27,38^:

2 $${F}_{{{{\rm{g}}}}}=\,\rho {gh}{L}_{1}{L}_{2}\varphi$$

where *ρ* is the liquid density, *g* is gravitational acceleration, h is the rise height, *L*_*1*_ and *L*_*2*_ are the lateral dimensions of the unit cell, and *φ* is the porosity of the structure.

To quantify geometric effects, the liquid–solid contact perimeter along the vertical axis was extracted for each unit cell type by analyzing sliced cross-sectional images of the lattice structures (Fig. 3C(i)). The resulting perimeter profiles reveal distinct geometry-dependent signatures, with pronounced variations in contact perimeter as a function of height (Fig. 3C(ii) and (iii)). Notably, FBCC-based architectures exhibited a consistently larger liquid-solid contact perimeter than other designs, resulting from the synergistic integration of BCC-derived internal struts and the high face density of FCC lattices. These geometric features were expected to enhance capillary adhesion and promote rapid liquid transport^39^. Capillary rise experiments were performed to validate this prediction (Fig. 3D). For visualization, poly(vinyl alcohol) (PVA)-coated samples were used instead of the graphite-coated, O_2_ plasma–treated evaporators, enabling clear observation of methylene blue transport within the lattice. The PVA-coated structures exhibited a contact angle (43.8 ± 5.9°) comparable to that of the graphite-coated and O_2_ plasma–treated samples (44.7 ± 8.8°), ensuring similar capillary driving forces (Supplementary Fig. 6). The experiments confirmed the geometric trend, with the FBCC-+ architecture exhibiting both the fastest rise kinetics and the highest equilibrium water elevation within 15 min of wicking (Supplementary Fig. 7 and Supplementary Movie 1).

A comparative summary of the average contact perimeter, porosity, and experimentally measured rise height is presented in Fig. 3E and Supplementary Table 1. A clear correlation was observed across all metrics, where structures with larger wetting contact perimeter and lower porosity exhibited faster wicking kinetics. This trend reflects the combined effect of enhanced capillary adhesion and reduced gravitational resistance, which together promoted efficient liquid transport through the lattice, consistent with geometry-governed capillary behavior in 3D porous architectures^40,41^.

To further isolate geometric influences within a single unit cell topology, water transport was additionally examined using the FBCC-+ unit cell as a representative structure. The frame thickness (*F*) and strut diameter (*D*) were varied simultaneously while maintaining a constant unit cell edge length of 2 mm. Among the tested configurations, the FBCC-+ structure with *F* = *D* = 400 μm exhibited the fastest capillary rise, consistent with its larger liquid–solid contact perimeter and lower porosity (Supplementary Fig. 8 and Supplementary Table 2). This behavior followed the same trend observed across different unit-cell geometries, confirming that enhanced adhesion forces associated with increased wetted perimeter, together with reduced liquid gravitational resistance from lower porosity, collectively govern water transport performance (Supplementary Fig. 9)^41^.

Collectively, these results establish that capillary transport in ordered porous systems can be predictably tuned through rational geometric design, providing a critical foundation for optimizing subsequent solar evaporation performance.

### Influence of unit cell geometry on evaporation performance

**Fig. 4:**
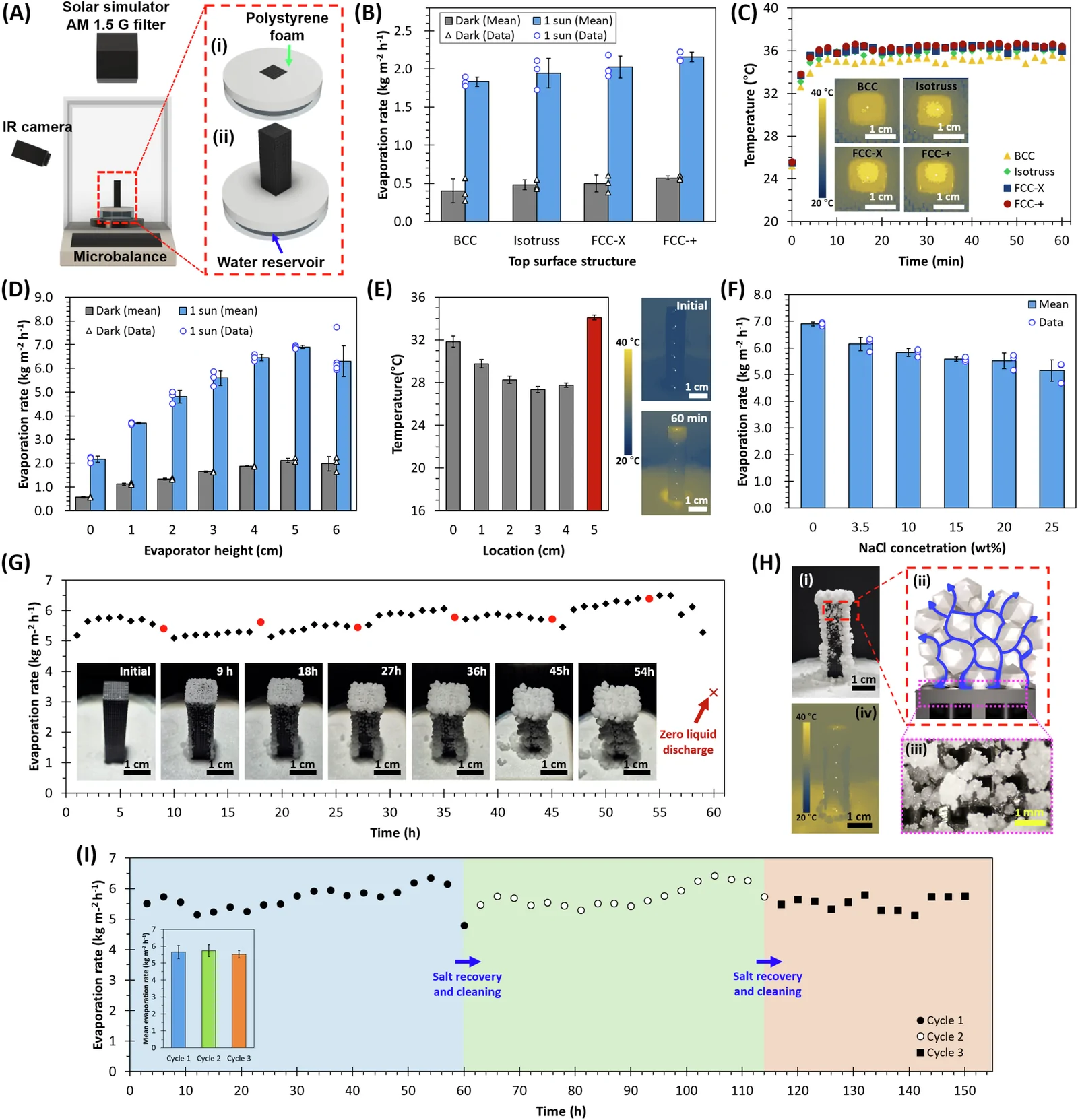
Evaporation performance of various MSEs. **A** Experimental setups for evaporation measurements; (i) 0 cm and (ii) controlled exposed height. **B** Evaporation rates of MSEs with different unit cell structures at 0 cm height. **C** Time-resolved temperature profiles at 0 cm height (inset: IR images at equilibrium during evaporation). **D** Height-dependent evaporation rate of FBCC-+ MSE. **E** Time-averaged temperature distribution of FBCC-+_5 at selected locations during evaporation, with IR images at the initial state and under 1 sun irradiation; error bars indicate the standard deviations (SDs) of the temperature measured at 1 min intervals. **F** Evaporation rate of FBCC-+_5 under different NaCl concentrations. **G** Long-term evaporation stability in 20 wt% NaCl (inset: optical images corresponding to red dots). **H** Water transport through salt crystals enabling sustained evaporation: (i) FBCC-+_5 after 60 h, (ii) transport schematic, (iii) wetted internal salt crystals, and (iv) infrared thermal images under salt-covered conditions. (I) Evaporation performance over three salt-recovery cycles in 20 wt% NaCl (inset: average evaporation rate per cycle). Values in B, D, and F are means ± SDs from *n* = 3 independent samples, except for the 6 cm condition in (**D**) (*n* = 7); bars and symbols in these panels represent mean values and individual measurements, respectively. Source data are provided as a Source Data file.

The solar evaporation performance of the MSEs was evaluated under 1 sun irradiation using the setup shown in Fig. 4A. The MSEs were constructed by overlapping adjacent unit-cell frames to form a continuous top surface consisting of a 6 × 6 array (Supplementary Fig. 10 and Supplementary Table 3). To evaluate the effect of unit cell structure, evaporation measurements were first conducted on MSEs constructed from different unit cell types while maintaining an identical exposed height (0 cm) above the insulating polystyrene (PS) foam. Although seven unit cell geometries were systematically investigated in the water-transport study, evaporation tests were performed on four representative architectures. This reduction is justified because FBCC-based structures share the same evaporative face geometry as their corresponding FCC counterparts, rendering their top-surface evaporation behavior effectively identical under zero-height conditions. The results showed clear structure-dependent evaporation behavior (Fig. 4B). Although all samples exhibited nearly identical surface temperature profiles during operation (Fig. 4C), the FCC-+ MSE achieved the highest evaporation rate of 2.16 ± 0.06 kg m^−2^ h^−1^, whereas the BCC MSE showed the lowest evaporation rate of 1.83 ± 0.06 kg m^−2^ h^−1^. Notably, all architectures also exhibited distinct evaporation rates under dark conditions, ranging from 0.40 to 0.57 kg m^−2^ h^−1^, which exceeded that of bulk water and followed the same structural trend (Supplementary Fig. 11).

To identify the geometric characteristics governing the observed evaporation differences, the light-harvesting area, defined as the projected solid area of the uppermost surface directly exposed to incident irradiation, was quantified (Supplementary Table 4). Despite substantial variation in light-harvesting area, ranging from 48.16 to 85.52 mm^2^, the surface temperatures during evaporation remained nearly identical (35.12–36.36 °C; Fig. 4C and Supplementary Fig. 12), and no correlation with evaporation rate was observed (Supplementary Fig. 13). For instance, although FCC-X exhibits the largest light-harvesting area, its evaporation performance was lower than that of FCC-+. These results indicate that the projected light-harvesting area alone does not govern the evaporation performance under the present configuration.

Since surface temperature and light-harvesting area could not fully explain these differences, the observed variations are attributed to geometry-dependent heat transfer through the solid–liquid interface. Assuming that thermal energy is primarily transferred through regions of solid–liquid contact near the pore edges, the effective thermal transfer area, *A*_eff_, can be approximated as:

3 $${A}_{{eff}}\approx {P}_{{pore}}\cdot H$$

and the rate of heat (*Q*) delivery scales as^42^:

4 $$Q\propto {k}_{s}\cdot {A}_{{eff}}$$

where *P*_pore_ is the pore perimeter, *H* is the frame thickness and *k*_s_ is the thermal conductivity of the lattice material. This effective thermal transfer area represents the solid–liquid interfacial region through which heat is delivered from the photothermal solid to the liquid at the active evaporation sites. Since all structures were fabricated from the same material, differences in evaporation performance originate from variations in effective solid–liquid contact area. Notably, the calculated effective thermal transfer areas showed a strong correlation with the experimentally measured evaporation rates (Supplementary Fig. 14), indicating that heat delivery to the evaporation interface is governed by a geometry-dependent solid–liquid contact region. This parameter, therefore, plays a dominant role in determining evaporation performance.

Based on the combined analyses of water transport and evaporation performance, the FBCC-+ geometry was selected for further optimization. FBCC-+ preserves the same evaporative surface as FCC-+ while exhibiting superior capillary transport and among the highest evaporation rates, making it an optimal candidate for subsequent parametric investigations (Supplementary Table 5). Evaporation measurements were conducted with only the top surface exposed (i.e., at 0 cm height), confirming comparable performance between FBCC-+ and FCC-+, consistent with their identical evaporative surface geometry (Supplementary Fig. 15). To further isolate geometric effects, evaporation performance was examined within a single topology using FBCC-+ unit cells with systematically varied frame thickness (*F*) and strut diameter (*D*), while maintaining a constant unit-cell edge length of 2 mm. The FBCC-+ structure with *F* = *D* = 400 μm exhibited the highest evaporation rate (2.16 ± 0.06 kg m^−2^ h^−1^), whereas the structure with *F* = *D* = 200 μm exhibited the lowest value (1.93 ± 0.16 kg m^−2^ h^−1^) (Supplementary Fig. 16). This result follows the variation in effective thermal transfer area (Supplementary Fig. 16 and Supplementary Table 6), and it is consistent with the behavior observed across different unit-cell geometries. Collectively, these results confirm that the effective thermal transport area serves as a governing parameter for heat delivery and dominates the evaporation performance.

To evaluate the effect of exposed geometry, a series of FBCC-+ MSEs with increasing height were fabricated (Supplementary Fig. 17 and Supplementary Table 7). Increasing the height introduced additional lateral surface area, enabling supplementary evaporation pathways beyond the top surface. Accordingly, the evaporation rate increased from 2.16 ± 0.06 kg m^−2^ h^−1^ at 0 cm height to 6.90 ± 0.07 kg m^−2^ h^−1^ for the 5 cm configuration, denoted as FBCC-+_5 (Fig. 4D). However, a further increase to 6 cm reduced the evaporation rate to 6.30 ± 0.65 kg m^−2^ h^−1^, indicating the existence of an optimal height under the present design conditions. This decline is attributed to increased capillary transport resistance associated with the longer water transport pathway, which ultimately outweighs the benefit of the additional evaporative area. As FBCC-+_5 exhibited the highest evaporation performance among all tested configurations, it was selected as the representative geometry for subsequent salinity, crystallization, and long-term stability studies. The contribution of the lateral surface was quantified (Supplementary Table 8), showing that the lateral evaporation area increases monotonically with height and accounts for 68.70% of the total evaporation for the FBCC-+_5. In parallel, the evaporation rate under dark conditions also increased with height, reflecting the enlarged evaporative area and enhanced energy harvesting from the surrounding environment through the lateral surface (Supplementary Fig. 18). Infrared thermal imaging confirms effective heat localization within the FBCC-+_5 structure (Fig. 4E). During evaporation, the top surface rapidly reached a steady-state temperature of 34.1 °C, while the lateral surfaces remained cooler at approximately 27.7 °C, slightly below the ambient temperature ( ~ 28.9 °C). This temperature gradient creates cold-evaporation zones along the sidewalls, enabling additional convective and radiative heat uptake from the environment and thereby enhancing evaporation performance^43,44^. A slight temperature increase near the base ( ~ 31.8 °C) was observed, which is attributed to partial light reflection from the PS foam rather than thermal leakage from the evaporation surface, further confirming efficient heat localization. Control experiments using a non-reflective sheet placed on the PS foam yielded comparable evaporation rates, while the lateral surface temperature remained lower than the top surface, indicating that reflected light has a negligible influence on evaporation performance and confirming effective heat localization (Supplementary Fig. 19). This synergistic coupling of environmental heat with incident solar energy yields an apparent energy utilization efficiency of 326.9% under 1 sun illumination (Method S1 and Supplementary Fig. 20), exceeding that of conventional two-dimensional evaporators^45–47^ (Supplementary Table 9). It should be noted that this value includes additional heat uptake from the surrounding environment through the lateral surfaces; therefore, the calculated efficiency should be interpreted as an apparent metric rather than a purely solar-to-vapor conversion efficiency. Together, these observations demonstrate that increasing evaporator height simultaneously introduces lateral evaporation pathways and enables passive environmental heat harvesting, both of which contribute to the superior performance of FBCC-+_5.

The salt tolerance of the MSE was further evaluated across a wide range of NaCl concentrations using FBCC-+_5 configurations (Fig. 4F). For reference, 3.5 wt% NaCl corresponds to the average salinity of natural seawater. High evaporation rates were maintained across a broad range of salinity levels, confirming strong resistance to salt-induced performance degradation. A gradual decline in evaporation rate was observed at very high salt concentrations, decreasing to 5.58, 5.52, and 5.16 kg m^−2^ h^−1^ at 15 wt%, 20 wt%, and 25 wt% NaCl, respectively. This decrease in evaporation rate was attributed to increased solution viscosity and thermal conductivity, which slowed water transport and enhance heat dissipation, as well as reduced saturated vapor pressure in high salt solutions^48,49^.

Long-term evaporation tests using 20 wt% NaCl were conducted to assess operational robustness under hypersaline conditions. The FBCC-+_5 evaporator maintained a stable evaporation rate throughout extended operation without measurable performance decay (Fig. 4G). During the initial hours, only small salt crystallites formed near the exposed upper region, while interconnected capillary pathways continued to supply water to the evaporation interface. As the operation progressed beyond 27 h, large salt crystals progressively covered the external surface. However, unlike conventional systems where salt accumulation suppresses evaporation, the evaporation rate remained unchanged.

This sustained performance arises from the open-cell architecture of FBCC-+_5, which enables continuous brine transport through the interconnected lattice and supports the growth of hydrated, micron-scale porous salt structures^26^. The relatively large pore openings direct salt nucleation along the lattice frames while maintaining open transport channels, allowing crystals to grow outward through continuous brine uptake without blocking liquid pathways (Supplementary Fig. 21). This multiscale porosity preserves hydraulic continuity and provides additional evaporative area, effectively offsetting geometric surface coverage^50^. Moreover, the porous salt layer formed on FBCC-+_5 reduced vapor diffusion resistance, allowing rapid vapor escape through interconnected pores while preventing mass-transfer limitations caused by vapor retention^51^. Post-operation analysis revealed that the salt layer remained hydrated and porous rather than forming a dry insulating crust, allowing continuous liquid migration from the lattice into the salt matrix (Fig. 4H(i) and (ii)). Optical observations beneath the salt layer confirmed a wet evaporation interface, indicating that evaporation occurred directly from the hydrated salt layer rather than from the underlying evaporator (Fig. 4H(iii) and Supplementary Fig. 22)^52^. Infrared imaging further corroborated this behavior, showing that salt-covered regions exhibited temperatures comparable to uncovered evaporating surfaces, confirming their active role in evaporation (Fig. 4H(iv) and Supplementary Fig. 23)^53^. This wet-crystal evaporation mechanism enables salt to function as an auxiliary evaporating layer rather than a failure mode, allowing FBCC-+_5 MSE to sustain high-flux evaporation under extreme salinity.

After 60 h of continuous operation, the liquid reservoir was fully depleted, and a dense salt crystal remained on the evaporator surface, signifying successful achievement of ZLD (Supplementary Fig. 24). Approximately 96% of the initially dissolved NaCl was recovered, indicating efficient salt crystallization with minimal ion loss. Following gentle salt removal and rinsing with deionized water, the evaporator fully recovered its original performance without structural or functional degradation (Supplementary Fig. 25 and Fig. 4I). Repeated crystallization–cleaning cycles further confirmed excellent durability and reusability, with minimal variation in evaporation rate, with the first cycle yielded 5.66 ± 0.38 kg m^−2^ h^−1^ and the final cycle yielded 5.53 ± 0.22 kg m^−2^ h^−1^, indicating negligible performance decay (Supplementary Fig. 26). Collectively, these results demonstrate that the homogeneous FBCC-+ MSE enables high evaporation rates, robust salt tolerance, and long-term reusability, highlighting the advantages of geometry-engineered evaporators for solar-driven desalination and salt crystallization.

### Geometry-modulated salt crystal localization

**Fig. 5:**
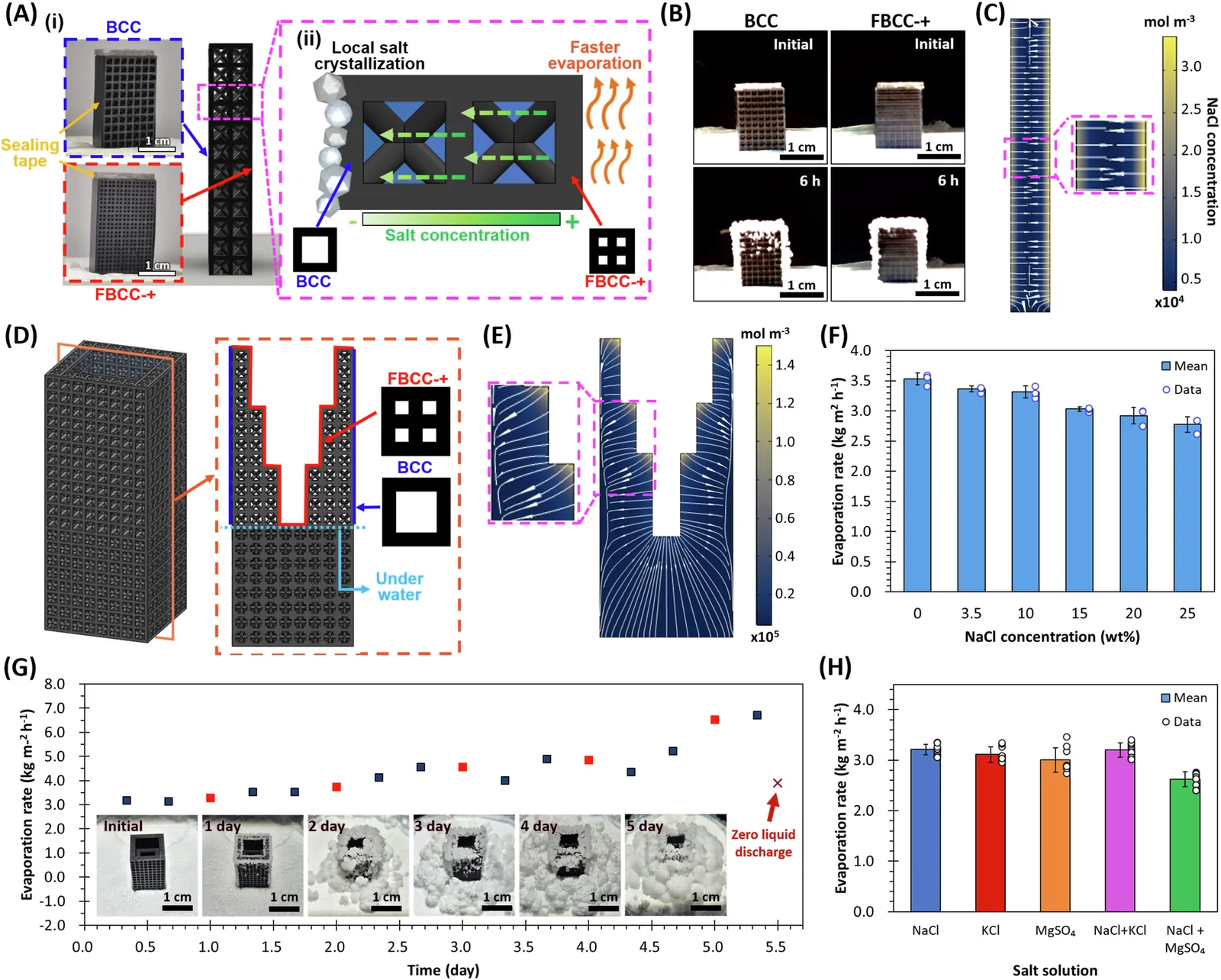
Geometric-modulated salt crystal localization. **A** Illustration of (i) hybrid-cell structures and (ii) diffusion-driven salt localization induced by geometry-dependent evaporation rates. **B** Optical images of hybrid structures at the initial state and after 6 h in 20 wt% NaCl, comparing BCC and FBCC-+ surfaces. **C** Numerical simulation of concentration gradients and ion-transport flux; arrows indicate ion-transport directions. **D** Geometrical configuration of the hybrid cup-shaped evaporator. **E** Simulated ion concentration distribution and transport flux in the hybrid cup-shaped structure; arrows indicate ion-transport directions. **F** Evaporation rate of the hybrid cup-shaped evaporator under different NaCl concentrations. **G** Long-term stability in 20 wt% NaCl (inset: optical images showing localized salt accumulation corresponding to red dots). **H** Evaporation performance under various high-salinity solutions. Values in (**F**) are means ± SDs from *n* = 3 independent samples; bars and symbols represent mean values and individual measurements, respectively. In (**H**), symbols indicate time-resolved evaporation rates over 9 h. Source data are provided as a Source Data file.

As demonstrated in the previous section, the FBCC-+ MSE maintains stable evaporation performance even under hypersaline conditions. To further achieve spatial control over salt accumulation and more effectively suppress salt fouling, hybrid multicellular evaporators were designed by integrating unit cells with distinct evaporation characteristics. Among the architectures investigated, the BCC unit cell exhibited the lowest evaporation rate, whereas FBCC-+ showed the highest performance (Influence of unit cell geometry on evaporation performance, Fig. 4B). By deliberately combining these two unit cell types, a controlled evaporation-rate gradient was introduced within a single hybrid structure. Specifically, a hybrid evaporator comprising a BCC unit cell wall on one side and an FBCC-+ unit cell wall on the opposite side was fabricated (Fig. 5A(i)). This geometry-induced asymmetric evaporation rate induces a non-uniform salt concentration field within the interconnected liquid phase. Although the high-evaporation FBCC-+ region initially experiences stronger local salt enrichment, the resulting salinity gradient continuously redistributes ions toward the adjacent lower-flux BCC region through diffusive transport. The BCC domain, where water removal is slower and ion clearance is limited, therefore acts as a terminal accumulation zone in which supersaturation is reached preferentially^54^. As a result, salt ions preferentially accumulate and reach supersaturation in the low-evaporation region, leading to localized salt crystallization on the BCC side (Fig. 5A(ii)). Importantly, this salt redistribution and localization are governed solely from the unit cell geometric modulation, without any external energy input, active control, or selective membranes, thereby constituting a fully passive salt localization mechanism. Experiments conducted under hypersaline conditions using 20 wt% NaCl solution confirmed this behavior. After 6 h of solar irradiation, extensive salt crystallization was observed on the BCC region, while the FBCC-+ region remained largely free of surface salt accumulation (Fig. 5B and Supplementary Movie 2). These results demonstrate that evaporation flux modulated by structural geometry can actively guide salt crystallization and mitigate fouling during high-salinity operation. Numerical simulations further corroborated this mechanism, revealing pronounced ion concentration gradients and a directional ionic flux from regions of higher evaporation flux toward lower-flux regions (Fig. 5C).

Based on these results, a cup-shaped hybrid configuration was developed to amplify crystallization localization by spatially biasing ion redistribution while preserving open pathways for vapor escape (Fig. 5D and Supplementary Table 10). In this architecture, high-evaporation FBCC-+ unit cells were positioned on the top and inner surfaces, whereas low-evaporation BCC unit cells formed the outer region. This arrangement shortens the redistribution pathway between the evaporation and crystallization domains and promotes outward ion accumulation within a predefined peripheral zone. Numerical simulations revealed enhanced salt concentration buildup near the high-evaporation interface, accompanied by a directed ion flux toward the outer region, which served as a predefined crystallization zone (Fig. 5E). Detailed simulation procedures are provided in the Supporting Information (Supplementary Method S2 and Supplementary Fig. 27).

Evaporation measurements showed that the hybrid evaporator maintained stable and high evaporation performance over a wide range of NaCl concentrations (Fig. 5F). Similar to the homogeneous FBCC-+_5 evaporator, a modest decrease in evaporation rate was observed at very high salt concentrations, attributable to increased solution viscosity and thermal conductivity, as well as reduced saturated vapor pressure under hypersaline conditions^49^. Notably, during prolonged operation in the hypersaline 20 wt% NaCl solution, the hybrid evaporator sustained a constant evaporation rate without measurable degradation, while salt crystallization occurred selectively at the designated outer region rather than on the primary evaporation surface, achieving a salt recovery of 97.49% (Fig. 5G and Supplementary Table 11). The small unrecovered fraction was mainly attributed to minor losses during mechanical collection and trace residues remaining within the lattice, which could subsequently be dissolved during rinsing for reuse. The increased evaporation rate observed after the second day can be attributed to the formation of a porous salt layer. Similar to the homogeneous FBCC-+_5 MSE, hydrated porous salt crystals developed on the outer surface of the structure. These porous deposits enable continuous brine uptake through capillary transport while growing outward without blocking liquid pathways^50^. Moreover, as shown in Fig. 5G (inset), the salt crystals progressively extend outward, forming a coral-like porous network. This evolving porous salt structure effectively expands the evaporation interface and continuously harvests environmental heat, allowing the salt layer to function as an auxiliary evaporating medium rather than a failure mode^51^. This wet-crystal evaporation mechanism enables the cup-shaped hybrid MSE to sustain high-flux evaporation under extreme salinity.

Under the ZLD-type batch operation employed here, salt crystallization remained confined to the designated BCC region and grew outward without bridging to the FBCC-+ evaporation surface. Under continuous feed, salt accumulation would eventually reach a critical volume determined by the available crystallization space and device geometry. Although porous salt growth delays bridging and performance decay, regeneration would be required once the accumulated layer begins to impede vapor escape by extending toward the FBCC-+ region. This operational limit can be further extended through geometric optimization, such as increasing the designated crystallization volume.

The robustness of this geometry-driven strategy was further validated across diverse saline environments, including monovalent salts such as NaCl and KCl (20 wt% each), a divalent salt MgSO_4_ (15 wt%), and mixed-salt systems with total concentrations of 20 wt% for NaCl + KCl (10 wt% each) and 15 wt% for NaCl + MgSO_4_ (10 wt% NaCl and 5 wt% MgSO_4_) (Fig. 5H). All evaporation rates were measured under continuous 9 h illumination, during which no performance decay was observed (Supplementary Fig. 28).

Collectively, these results demonstrate that hybrid multicellular geometry enables programmable salt crystallization and effective salt self-management, allowing long-term stable evaporation under hypersaline conditions and advancing fully passive ZLD desalination.

### Freshwater yield performance of MSE

**Fig. 6:**
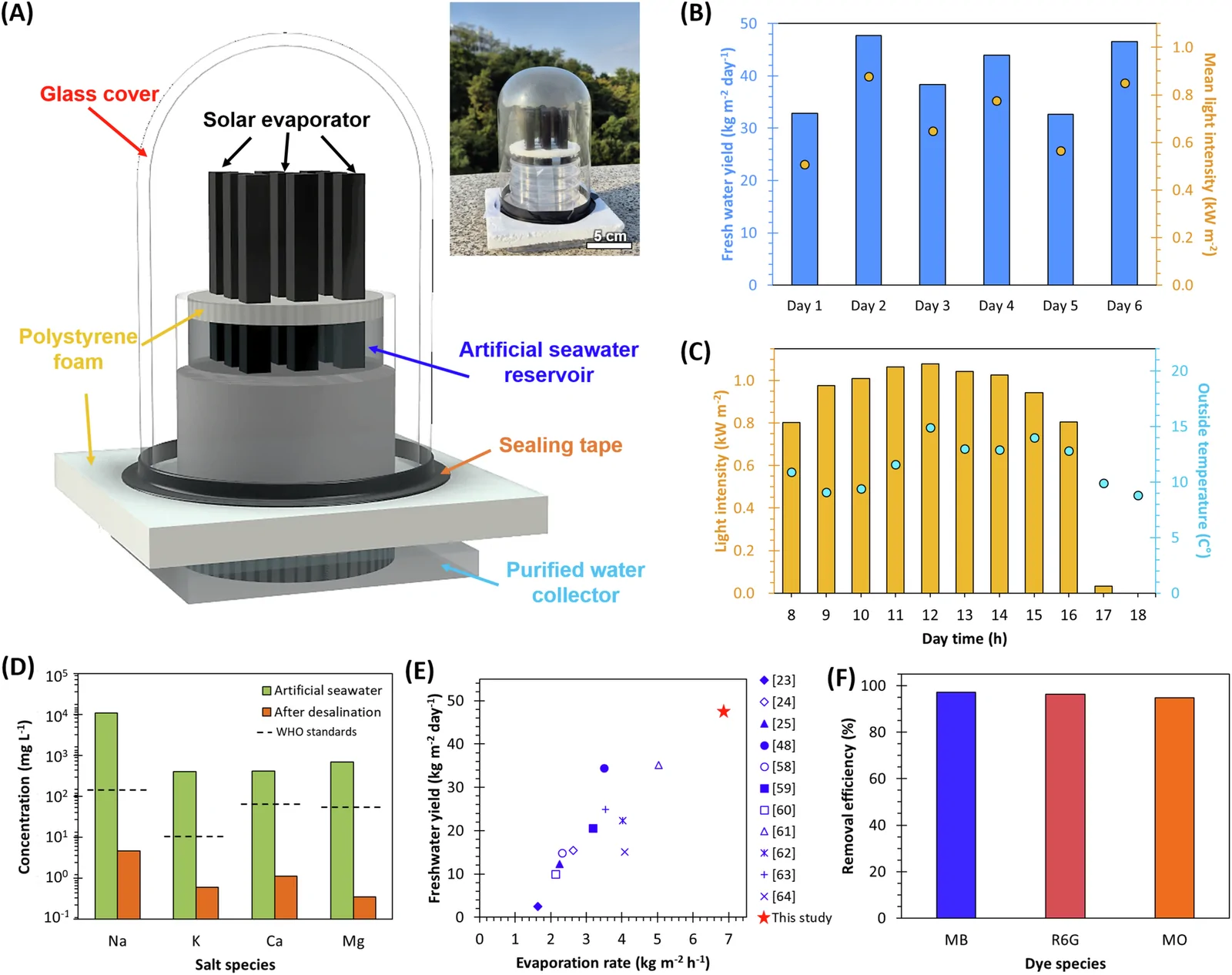
Outdoor freshwater production and water purification performance. **A** Schematic and photographs of the closed solar desalination chamber; all evaporators had an exposed height of 50 mm. **B** Outdoor freshwater yield of the evaporators and the corresponding average solar irradiance for each experimental day. **C** Time-resolved solar irradiance and ambient temperature profiles recorded on Day 2. **D** Ion concentrations in the simulated seawater and collected purified water; dashed lines indicate World Health Organization (WHO) drinking-water standards. **E** Comparison of freshwater yield with reported solar-evaporation systems; data are summarized in Supplementary Table 12. Evaporation rates were measured indoors under 1 sun, and maximum freshwater yields outdoors. Symbols denote literature data; the red star indicates this study. **F** Dye removal efficiency determined from the collected purified water using various dyes with an initial concentration of 5 mg L^−1^ under indoor 1 sun illumination; evaporation rates were not measured.

To evaluate practical feasibility beyond controlled laboratory conditions, outdoor desalination experiments were conducted using artificial seawater prepared according to established literature protocols^55,56^ (Method S3). Nine FBCC-+_5 evaporators were deployed in a seawater reservoir and enclosed within a dome-shaped glass cover to enable vapor condensation and collection (Fig. 6A). Under natural sunlight, water vapor generated at the evaporative surfaces condensed upon contact with the cooler glass cover, and the resulting droplets flowed downward into a collection chamber at the base of the system (Supplementary Fig. 29). Expanded PS foam was strategically placed on the water surface to suppress unintended solar absorption and evaporation from the regions outside the evaporator region.

The system exhibited stable freshwater production under outdoor conditions, with daily yields ranging from 32.6 to 47.7 kg m⁻² day⁻¹, which were closely correlated with fluctuations in solar irradiance (Fig. 6B and Supplementary Fig. 30). A maximum freshwater yield of 47.7 kg m^−2^ day^−1^ was achieved on Day 2, corresponding to the highest recorded average solar intensity of 0.878 kW m^−2^ (Fig. 6C). The average freshwater collection rate ( ~ 4.77 kg m^−2^ h^−1^) was lower than the evaporation rate measured in laboratory open-system experiments (6.90 kg m^−2^ h^−1^), which was attributed to elevated humidity in the enclosed configuration and temporal fluctuations in solar irradiation.

The quality of the collected freshwater was evaluated by inductively coupled plasma optical emission spectroscopy (ICP-OES) analysis of major ions (Na, K, Mg, and Ca) (Fig. 6D). Relative to the original artificial seawater, Na, K, and Mg concentrations were reduced by approximately three orders of magnitude, while Ca concentration decreased by two orders of magnitude. All ion concentrations in the collected water fell well below the drinking water limits specified by the World Health Organization, confirming the system’s ability to produce potable-quality water under realistic outdoor conditions^57^.

The developed MSE outperformed both the freshwater yield under natural sunlight and the evaporation rate measured under 1 sun illumination of those reported for previously demonstrated resin- and filament-based 3D-printed solar evaporators^23–25,48,58–64^. While many prior systems improved either evaporation rate or freshwater yield, the present multicellular evaporator achieved substantial enhancement in both metrics simultaneously (Fig. 6E and Supplementary Table 12), underscoring the effectiveness of geometry-driven design. Moreover, dye-removal experiments performed under 1 sun illumination demonstrated high purification efficiencies for multiple organic contaminants (Fig. 6F and Supplementary Fig. 31), highlighting the capability of the system to remove both salts and chemical pollutants.

From a manufacturing perspective, digital light processing (DLP) 3D printing was selected for its high precision and structural fidelity, enabling systematic exploration of geometry–property–performance relationships. The evaporators are composed of periodically repeated lattice units, enabling larger-area devices to be realized through modular tiling or replication while preserving the governing capillary transport and lateral evaporation pathways. Nevertheless, scaling this approach to larger-area systems will require addressing challenges associated with fabrication size limits while maintaining high resolution. Future efforts may focus on enhancing system-level performance through solar concentration strategies, optimized evaporator spacing, and architectural arrangements that balance vapor generation with optical and mass-transfer interactions^65,66^. Such advances are expected to further facilitate the translation of geometry-engineered solar evaporators toward scalable, real-world desalination and water purification applications.

## Discussion

This study establishes geometric design as a decisive factor governing the performance, stability, and salt tolerance of solar evaporators, beyond material composition alone. By systematically engineering ordered unit cell architectures using high-resolution 3D printing, the coupled roles of capillary water transport, solid–liquid heat transfer, and salt crystallization were systematically elucidated. Key geometric parameters, including liquid-solid contact perimeter, porosity, and thermal interface area, were identified as primary regulators of water delivery, energy transfer, and evaporation efficiency. Architectures with enlarged wetting perimeters and optimized porosity exhibited accelerated and elevated capillary rise, demonstrating that liquid transport in ordered porous systems can be predictably programmed through geometry. Moreover, differences in evaporation performance are unambiguously attributed to geometry-dependent solid-liquid heat transfer, with architectures providing larger effective contact areas consistently achieving superior evaporation rates.

Among the investigated architectures, the FBCC-+ unit cell provided an optimal balance between capillary transport and thermal coupling. Increasing the evaporator height activated lateral evaporation and passive environmental heat harvesting, yielding a peak evaporation rate of 6.90 kg m^−2^ h^−1^ for the FBCC-+_5. Under hypersaline conditions of 20 wt% NaCl, this architecture sustained stable long-term operation through a geometry-enabled wet-crystal evaporation mechanism, achieving ZLD desalination with full reusability. Importantly, extending geometric control beyond homogeneous designs, hybrid multicellular architectures were shown to induce spatially selective salt crystallization through evaporation-rate gradients. By integrating unit cells with distinct evaporation behaviors, salt accumulation was deliberately directed away from active evaporation surfaces, enabling spatial salt crystallization without sacrificing evaporation performance and achieving a salt recovery up to 97.49%. Furthermore, outdoor demonstrations further confirmed practical relevance, with freshwater production reaching 47.7 kg m^−2^ day^−1^ under natural sunlight.

Collectively, these results demonstrate that unit cell and macro-scale geometry can be used as programmable design variables to control interfacial transport, heat localization, and salt deposition pathways in solar evaporators. The structure–function relationships identified here suggest general design principles under controlled material and wettability conditions. While these geometric effects are expected to be broadly applicable, the absolute performance may depend on material-specific properties, including wettability, optical absorption, and photothermal conversion efficiency. Although the periodic lattice design provides a clear route toward larger-area implementation through modular replication, further advances in manufacturing scalability and system-level optimization, including evaporator spacing, solar concentration, thermal and mass-transfer management, will be required to maximize practical water production. This work thus establishes a geometry-driven framework for designing scalable, durable, and high-performance solar evaporation systems, advancing solar-driven desalination and ZLD toward real-world implementation.

## Methods

### Design and fabrication of MSEs

The unit cell and multicellular models of the solar evaporators were designed using SolidWorks 2024 SP4.0 software (Dassault Systèmes). Each unit cell was defined with dimensions of 2 mm × 2 mm × 2 mm, a frame thickness of 400 μm, and cylindrical struts with a diameter of 400 μm. Multicellular assemblies were generated by periodic repetition of the unit cells to achieve the desired macroscopic geometries. All structures were fabricated via DLP 3D printing using a commercial printer (IM2, Carima) and a photocurable resin (Micro 150, CUKH15G, Carima). Printing was performed with a layer thickness of 25 μm to ensure high geometric fidelity. After printing, the samples were sonicated in isopropyl alcohol ( ≥ 99.5%, Samchun Chemicals) for 30 min to remove uncured resin, followed by oven-drying. The cleaned structures were then post-cured under ultraviolet irradiation (365 nm, 300 W; CL300Pro, Carima) for 30 s to complete polymer crosslinking. To impart photothermal functionality, the printed samples were dip-coated in a 2 wt% graphite-ethanol suspension, prepared using graphite powder (loss on drying ≤ 4%, Samchun Chemicals) and ethyl alcohol (70–75%, Duksan Pure Chemicals) and dried at 80 °C. This coating procedure was repeated three times to ensure uniform graphite coverage. Subsequently, oxygen plasma treatment (CUTE, Femto Science) was applied at 100 W with an oxygen flow of 20 sccm and a chamber pressure of 7.6 × 10^2^ Torr for 3 min to enhance surface hydrophilicity and promote water-solid interaction. Residual excess graphite particles were removed by immersing the samples in distilled water, followed by sonication for 40 min, until no further particle detachment was observed. Finally, the treated evaporators were oven-dried overnight at 80 °C.

### Characterization of the MSEs

The structural morphology of the MSE was examined using a stereoscopic microscope (SZ61, Olympus) equipped with a CMOS camera (KCS3-63S, Korea Lab Tech) incorporating an IMX178 sensor with a resolution of 6.44 megapixels and a sensor size of 1/1.8 inch. Surface wettability was evaluated by static contact angle measurements using a contact angle goniometer (L2004A1, Ossila). Measurements were conducted on the 3D-printed samples before and after graphite surface modification, and the contact angle values were analyzed using Ossila Contact Angle software. Microstructural features were characterized by SEM (Quanta FEG 250, FEI). Prior to SEM observation, the samples were completely dried and sputter-coated with a thin platinum (Pt) layer to improve surface conductivity and minimize charging during imaging. Crystallographic features of the surface were analyzed by XRD (SmartLab, Rigaku). Chemical changes associated with surface modification were characterized by FTIR spectroscopy (Spectrum Two, PerkinElmer) using a diamond attenuated total reflectance (ATR) accessory. Spectra were collected before and after graphite coating over the range of 4000–550 cm^−1^ at an instrument scan speed of 0.2, to verify surface functionalization.

The photothermal response of the evaporator surface was monitored in real time using an infrared thermal camera (AT313X, Iris Solution), allowing quantitative analysis of surface temperature distribution and temporal thermal evolution under light irradiation. Optical absorption properties were measured using a UV–Vis–NIR spectrophotometer (Cary 5000, Agilent Technologies) equipped with an integrating sphere over a wavelength range of 250–2500 nm. The spectral absorptance (*A*) was calculated according to:

5 A=1−R−T

where *R* and *T* represent reflectance and transmittance, respectively.

### Water transport experiments

To evaluate the influence of unit cell geometry on capillary-driven water transport, a series of single-column multicellular structures with dimensions of 1 × 1 × *N* unit cells was prepared, where *N* denotes the number of unit cells stacked along the vertical direction. Each structure was vertically mounted on a custom stand above a glass Petri dish containing 50 mL of methylene blue (MB) aqueous solution (0.2 g L^−1^), which was prepared by dissolving methylene blue hydrate ( ≥ 97.0%, Sigma-Aldrich) in distilled (DI) water. MB was employed as a tracer dye to clearly visualize liquid infiltration and transport within the lattice, enabling direct observation of the capillary rise process through the interconnected pore network. Prior to testing, the multicellular structures were treated with oxygen plasma and subsequently coated with a 1 wt% poly(vinyl alcohol) (PVA, #1500, Samchun Chemicals) solution to ensure uniform hydrophilicity and stable wetting during capillary rise experiments. The bottom surface of each structure was gently brought into contact with the MB solution, allowing the liquid to wick upward through the lattice under capillary action until a steady-state height was reached. The time-dependent capillary rise behavior was recorded for 15 min using a digital camera (APC925, ABKO), and images were captured both during the rise process and after equilibrium was established. The capillary rise height as a function of time, as well as the equilibrium rise height, were quantified by analyzing the recorded images using ImageJ software. The liquid–solid contact perimeter along the vertical direction was extracted using a custom MATLAB R2023b (MathWorks) script based on cross-sectional geometry, while the porosity of each unit cell design was calculated directly from the corresponding models in SolidWorks software.

### Solar water evaporation performance evaluation

Solar-driven water evaporation experiments were conducted using a laboratory-scale setup to evaluate the evaporation and salt crystallization performance of the multicellular solar evaporators. A solar simulator (XES-50S2, SAN-EI Electric) equipped with an AM 1.5 G filter was used as the light source. The incident solar intensity at the evaporator surface was calibrated to 1000 W m^−2^ using a solar power meter (DS-05A, Daystar). The illumination beam size (5 cm × 5 cm) exceeded the lateral dimensions of the photothermal evaporator and was aligned perpendicular to the evaporation surface to ensure uniform irradiation. Mass changes in the water reservoir were recorded in real time using an electronic analytical balance (HS-520S, Hansung Instruments) with an accuracy of 2 mg. The evaporation rate (ER, kg m^−2^ h^−1^) was calculated according to Eq. (6):

6 $${{{\rm{ER}}}}=\frac{\Delta m}{\Delta t\cdot A}$$

where Δ*m* denotes the mass of evaporated water over the experimental time Δ*t*, and *A* represents the illuminated evaporation area.

Prior to evaporation measurements, the evaporators were immersed for 30 min in deionized water or sodium chloride (NaCl, ≥ 99.0%, Samchun Chemicals) solutions at concentrations of 3.5, 10, 15, 20, and 25 wt% to ensure complete wetting of the internal porous structure. Evaporation rates were then measured under identical illumination conditions for each solution.

Surface temperature distributions during operation were monitored in real time using an infrared thermal camera (AT313X, Iris Solution). To minimize bulk water evaporation, the evaporators were embedded in thermally insulating polystyrene (PS) foam, ensuring that evaporation occurred predominantly from the photothermal surfaces.

For long-term stability experiments conducted with a 20 wt% NaCl solution, the measurements were performed in repeated illumination-rest sequences consisting of 9 h of continuous solar irradiation followed by a 3 h dark period. This sequence protocol was implemented to prevent overheating of the solar simulator. Salt crystals formed during operation were not manually removed unless otherwise specified. All laboratory measurements were conducted at ambient conditions with a temperature ranging from 27–29 °C and a relative humidity of 55 ± 5%, monitored using a thermo-hygrometer (635-2, TESTO). Unless otherwise specified, all evaporation rate measurements were performed using three independently prepared evaporator samples for each condition.

### Outdoor solar desalination and water purification

Outdoor solar desalination experiments were conducted daily from 10:00 to 18:00 at Korea University, Seoul, Republic of Korea (37.583096° N, 127.026086° E), between November 6 and November 14, 2025. During the experiments, solar irradiance and ambient temperature were recorded hourly using a solar power meter (DS-05A, Daystar) and a thermo-hygrometer (635-2, TESTO), respectively. To evaluate desalination efficiency, ion concentrations in the feed solution and collected freshwater were directly quantified using inductively coupled plasma optical emission spectroscopy (ICP-OES, Agilent 5100), without any dilution or pretreatment prior to analysis.

The freshwater yield was calculated according to Eq. (7):

7 $${{{\rm{Freshwater\; yield}}}}=\frac{m}{A\cdot \Delta t}$$

where *m* is the collected freshwater mass in the closed condensation chamber, *A* is the total cross-sectional area of the evaporators, and Δ*t* is the experimental time.

Water purification experiments targeting organic contaminants were performed under 1 sun illumination (1000 W m^-2^) using the same experimental configuration as the outdoor desalination setup. Three representative dyes, MB ( ≥ 97.0%, Sigma-Aldrich), rhodamine 6G (R6G, ~ 95% dye content, Sigma-Aldrich), and methyl orange (MO, ~ 85% dye content, Sigma-Aldrich), were employed as model pollutants. Each dye solution was prepared by dissolving the corresponding dye in DI water at an initial concentration of 5 mg L^−1^. After 9 h of evaporation, the condensed water was collected and analyzed. The residual dye content in the collected condensate was evaluated using a microplate reader (800TS, BioTek) at the characteristic wavelengths of 660 nm for MB, 540 nm for R6G, and 490 nm for MO. For each collected condensate sample, the absorbance at the corresponding characteristic wavelength was measured six times using the same condensate to reduce instrumental measurement error, and the mean absorbance value was used for the calculation.

The removal efficiency (%) was calculated using Eq. (8):

8 $${{{\rm{Removal\; efficiency}}}}\left(\%\right)=\left(1-\frac{{A}_{c}-{A}_{{water}}}{{A}_{0}-{A}_{{water}}}\right)\times 100$$

where *A*_0_, *A*_c_ and *A*_water_ represent the absorbance values of the initial dye solution, the collected condensed water, and DI water, respectively.

## Supplementary information

Supplementary information

Description of Additional Supplementary File

Supplementary Movie 1

Supplementary Movie 2

Transparent Peer Review file

## Source data

Source Data 1

## Data Availability

The data supporting the findings of the study are included in the main text, Supplementary Information, and Source Data file. Raw data can be obtained from the corresponding author upon request. Source data are provided in this paper.
