# Supplementary material for: Multicellular architectures for high-rate solar evaporation with spatial salt crystallization under high-salinity
Source: Nat Commun. 2026 Jul 22;17:8946. doi: 10.1038/s41467-026-75913-w (PMC13503716; doi:10.1038/s41467-026-75913-w)
Supplement: Supplementary file 1 [file 41467_2026_75913_MOESM1_ESM.pdf]

[Supplementary Information]

Multicellular Architectures for High-Rate Solar Evaporation with Spatial Salt Crystallization under High-Salinity

Josue Yaedalm Son[1], Yunsan Choi[1], Xitong Liu[2], Run Hu[3, †], and Hyejeong Kim[1,4,†]

[1] School of Mechanical Engineering, Korea University, Seoul 02841, Republic of Korea

2 Department of Civil and Environmental Engineering, The George Washington University, Washington, DC 20052, United States

[3] School of Energy and Power Engineering, Huazhong University of Science and Technology, Wuhan, 430074, China

[4] Max Planck Institute for Dynamics and Self-Organization, Am Faßberg 17, 37077 Göttingen, Germany

†Corresponding authors

e-mail: h_kim@korea.ac.kr, Hyejeong Kim

hurun@hust.edu.cn, Run Hu

phone: +82-2-3290-3379, Hyejeong Kim

Contents of the Supplementary Information

Supplementary figure and tables

Figure S1. Stability of the graphite coating on multicellular solar evaporator (MSE).

Figure S2. X-ray diffraction (XRD) patterns of the samples.

Figure S3. Optical and scanning electron microscopy (SEM) images of the multicellular structures before and after graphite coating.

Figure S4. Contact angle measurements of the surface wettability.

Figure S5. Transmittance and reflectance spectra of samples before and after graphite coating.

Figure S6. Rationale for using PVA-coated samples for water-transport visualization.

Figure S7. Time-dependent capillary rise behavior of different unit-cell structures.

Table S1. Geometric characteristics and capillary rise of different unit cell architectures.

Figure S8. Capillary transport analysis FBCC-+ unit cells with varying frame thickness (*F*) and strut diameter (*D*), with constant unit-cell edge length of 2 mm.

Table S2. Geometric parameters and measured capillary rise of FBCC-+ unit cells

Figure S9. Correlation between geometrical characteristics and capillary performance of different unit cell structures.

Figure S10. Schematic illustration of the geometrical characteristics of the MSE.

Table S3. Geometric parameters of MSEs at 0 cm exposed height.

Figure S11. Mass change during evaporation at 0 cm exposed height.

Table S4. Light-harvesting area of different unit cell architectures

Figure S12. Average surface temperature during evaporation for different unit-cell architectures.

Figure S13. Correlation between evaporation rate and light-harvesting area.

Figure S14. Correlation between evaporation rate and effective thermal transfer area (A_{eff})

Table S5. Effective thermal transport area of different unit cell architectures.

Figure S15. Evaporation performance analysis of FCC-+ and FBCC-+ MSE.

Figure S16. Evaporation performance analysis of FBCC-+ unit cells with varying frame thickness (*F*) and strut diameter (*D*), with constant unit-cell edge length of 2 mm on evaporation rate

Table S6. Geometric parameters and evaporation rate of FBCC-+ unit cells.

Figure S17. Optical images of FBCC-+ evaporators with varying heights.

Table S7. Geometric parameters of FBCC-+ MSEs at different exposed height.

Table S8. Lateral surface contribution to evaporation performance of MSEs.

Figure S18. Mass change during evaporation at controlled exposed height.

Figure S19. Experiments to identify the effect of partial light reflection from the polystyrene (PS) foam.

Figure S20. Apparent energy utilization efficiency of FBCC-+ evaporators with different heights.

Table S9. Apparent energy utilization efficiency of different solar evaporators find in literature

Figure S21. Schematic image illustrating the formation process of porous wet salts crystals on the surface of the MSE during continuous solar desalination.

Figure S22. Optical images of wet salt crystals formed during long-term solar evaporation.

Figure S23. Thermal behavior of salt-covered FBCC-+_5 during evaporation.

Figure S24. Optical image of FBCC-+_5 after complete evaporation of the liquid reservoir.

Figure S25. Salt collection and regeneration process following long-term desalination.

Figure S26. Long-term cycling performance of FBCC-+_5 after repeated salt removal and cleaning.

Table S10. Geometric parameters of cup-shaped hybrid evaporator.

Figure S27. Geometry and evaporation analysis of the cup-shaped hybrid evaporator.

Figure S28. Long-term evaporation behavior under various high-concentration salt solutions.

Table S11. Salt recovery efficiencies of different MSEs.

Figure S29. Optical images recorded during outdoor desalination experiments.

Figure S30. Time-resolved solar irradiance and ambient temperature during outdoor tests on 2025.

Figure S31. Optical images of dye solutions before and after solar evaporation and water collection.

Table S12. Comparison of recent resin- and filament-based 3D-printed solar evaporators.

Supplementary methods

Method S1. Calculation of apparent energy utilization efficiency.

Method S2. Numerical simulation methodology.

Method S3. Preparation of artificial seawater.

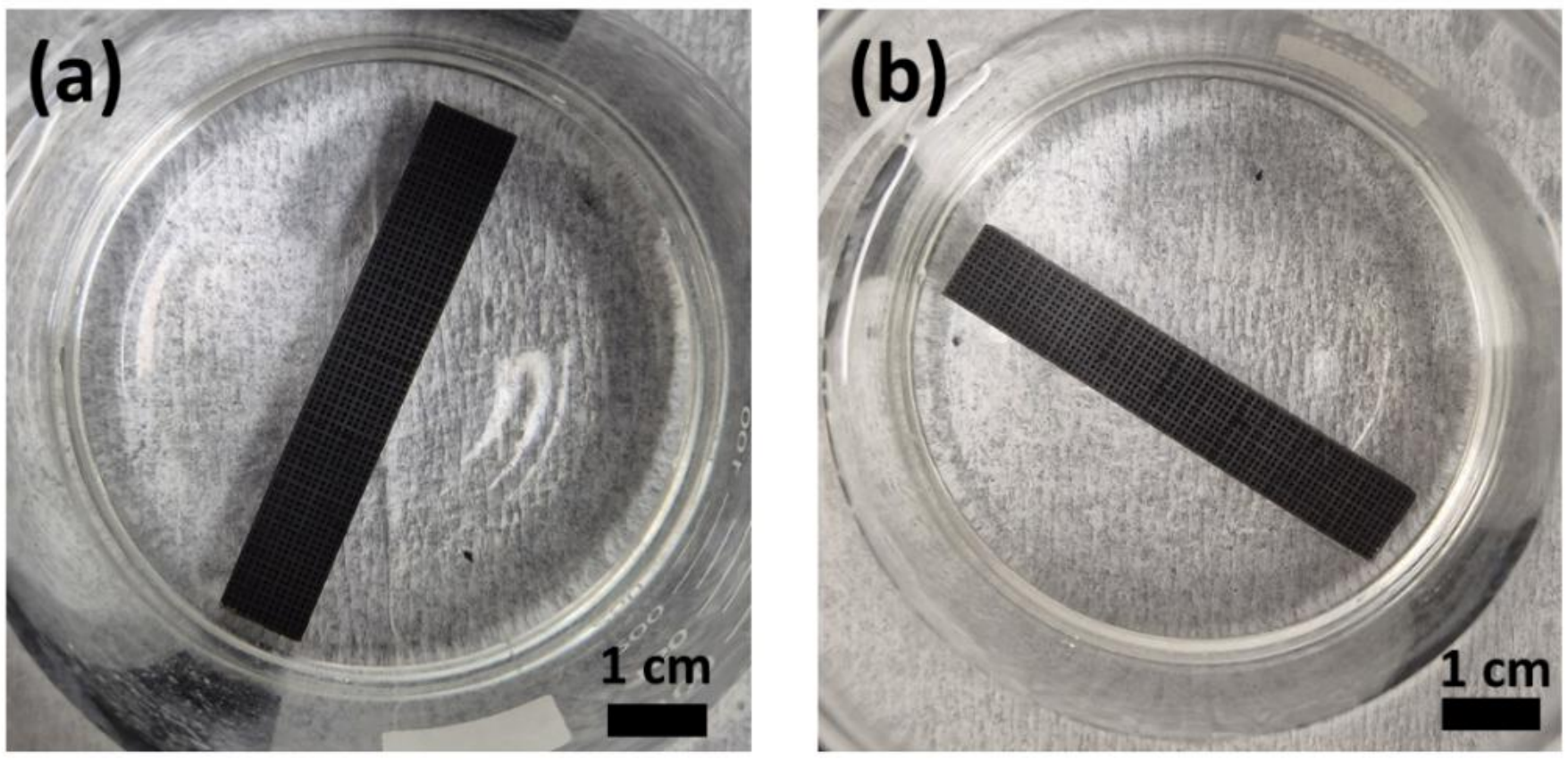

Figure S1. Stability of the graphite coating on the MSE. (a) before ultrasonication and (b) after 30 min of ultrasonication, showing no observable detachment or residual loss of the graphite layer.

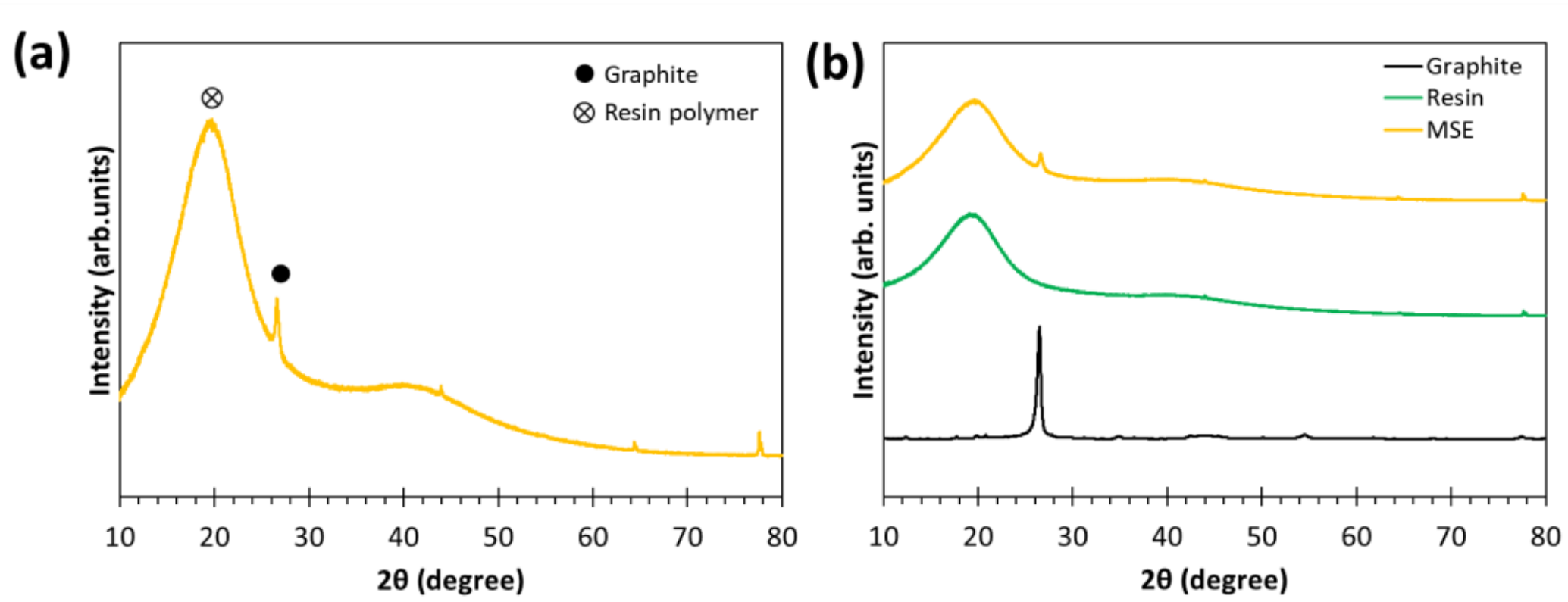

Figure S2. X-ray diffraction (XRD) patterns of the samples. (a) MSE after graphite coating and oxygen plasma treatment; (b) comparison of XRD patterns of commercial graphite, bare resin, and MSE.

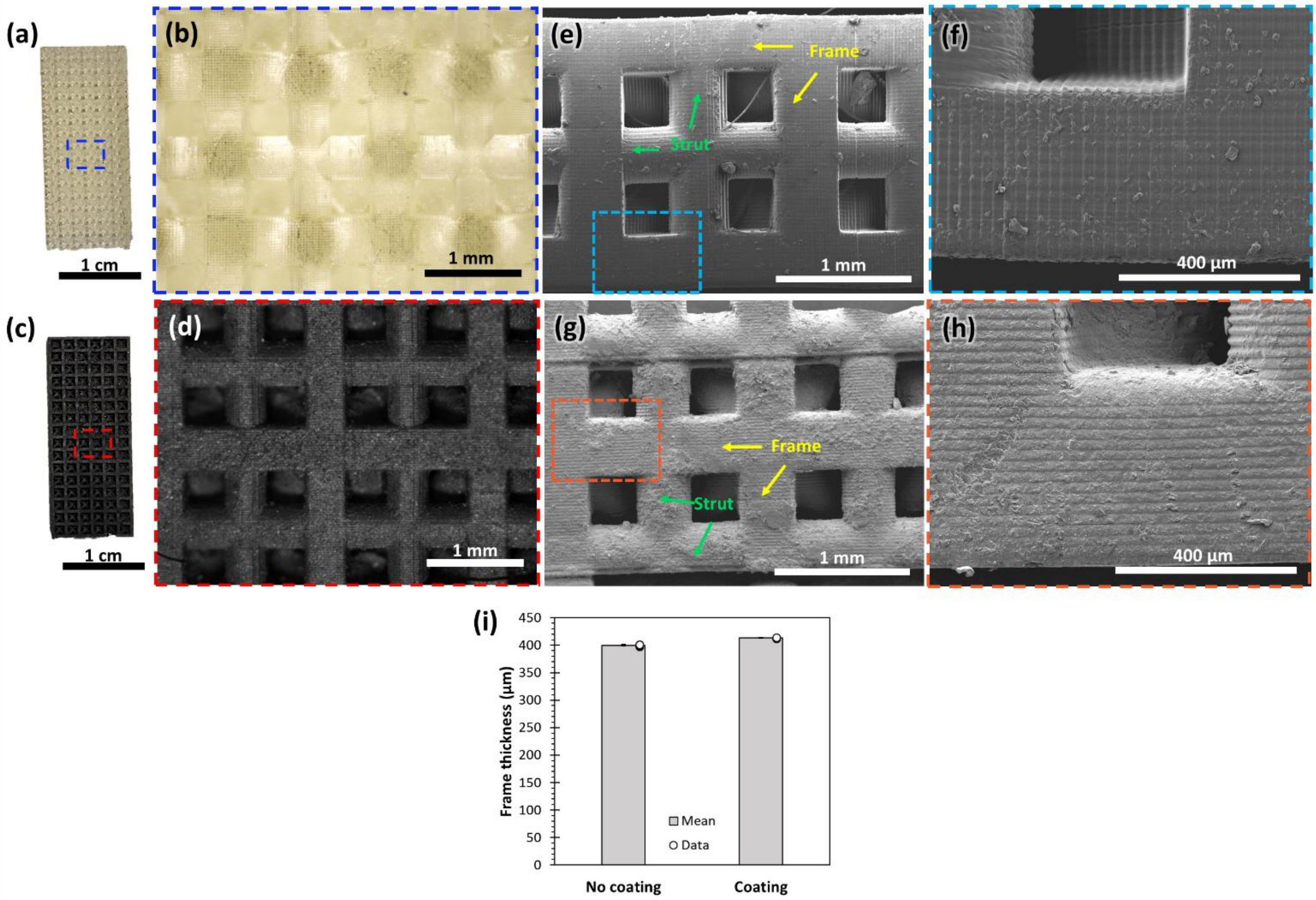

Figure S3. Optical and scanning electron microscopy (SEM) images of the multicellular structures before and after graphite coating. Optical images with corresponding higher-magnification views of (a, b) structure before coating and (c, d) after coating. SEM images of the structure (e) before coating and (g) after coating, with arrows indicating the frame and strut features. (f, h) Higher-magnification SEM images of the frame before and after coating, respectively, confirming homogeneous surface coverage. (i) Frame thickness before and after coating, quantified using ImageJ software. Bars and symbols represent mean values and individual measurements, respectively. Error bars indicate standard deviation.

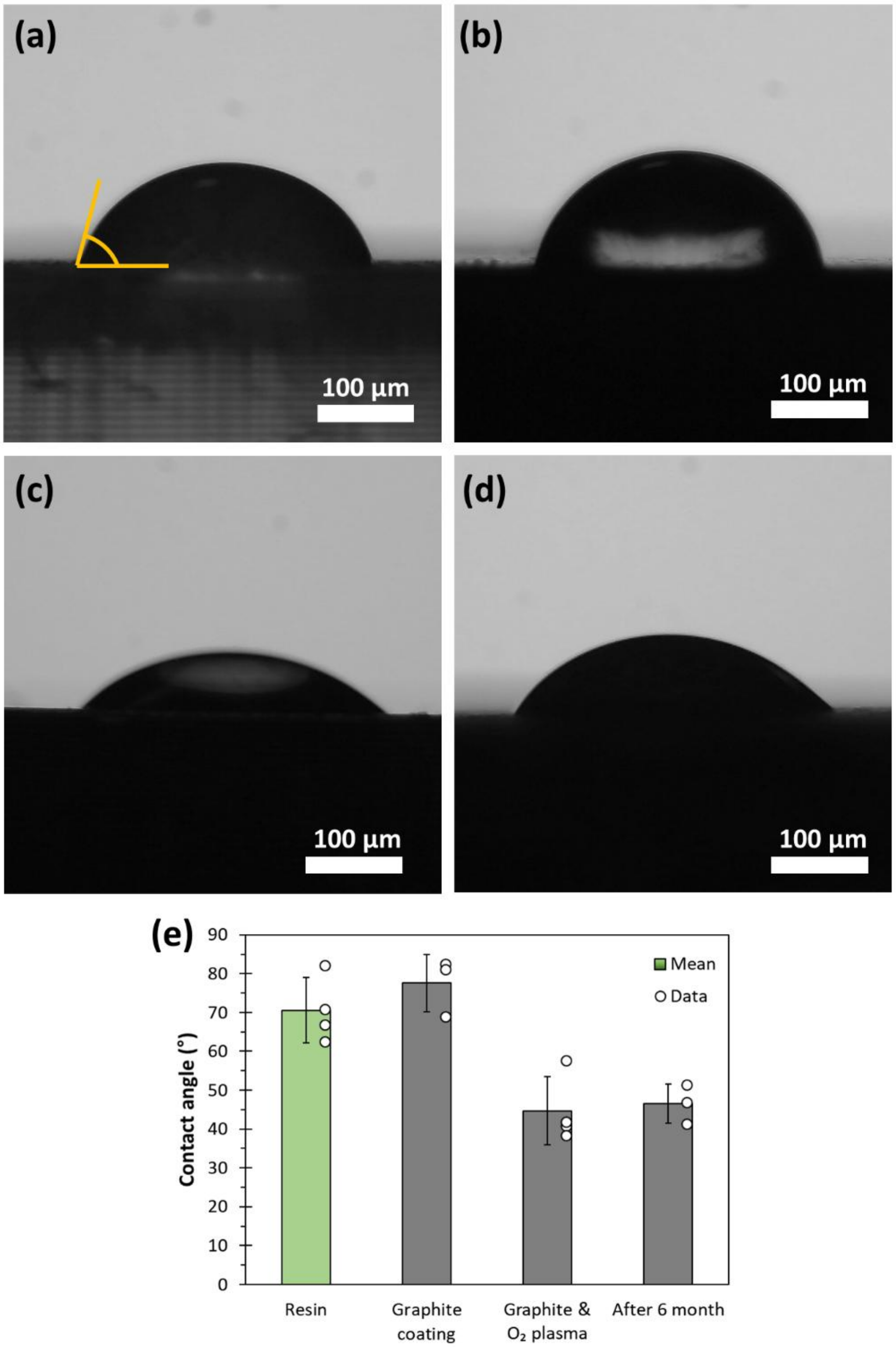

Figure S4. Contact angle measurements of the surface wettability. Optical images of water droplets on (a) pristine resin, (b) graphite-coated surface without O_2 plasma treatment, (c) graphite-coated surface after O_2 plasma treatment, and (d) the plasma-treated sample after 6 months. (e) Comparison of the measured contact angles for each condition. Bars and symbols represent mean values and individual measurements, respectively. Error bars indicate standard deviation.

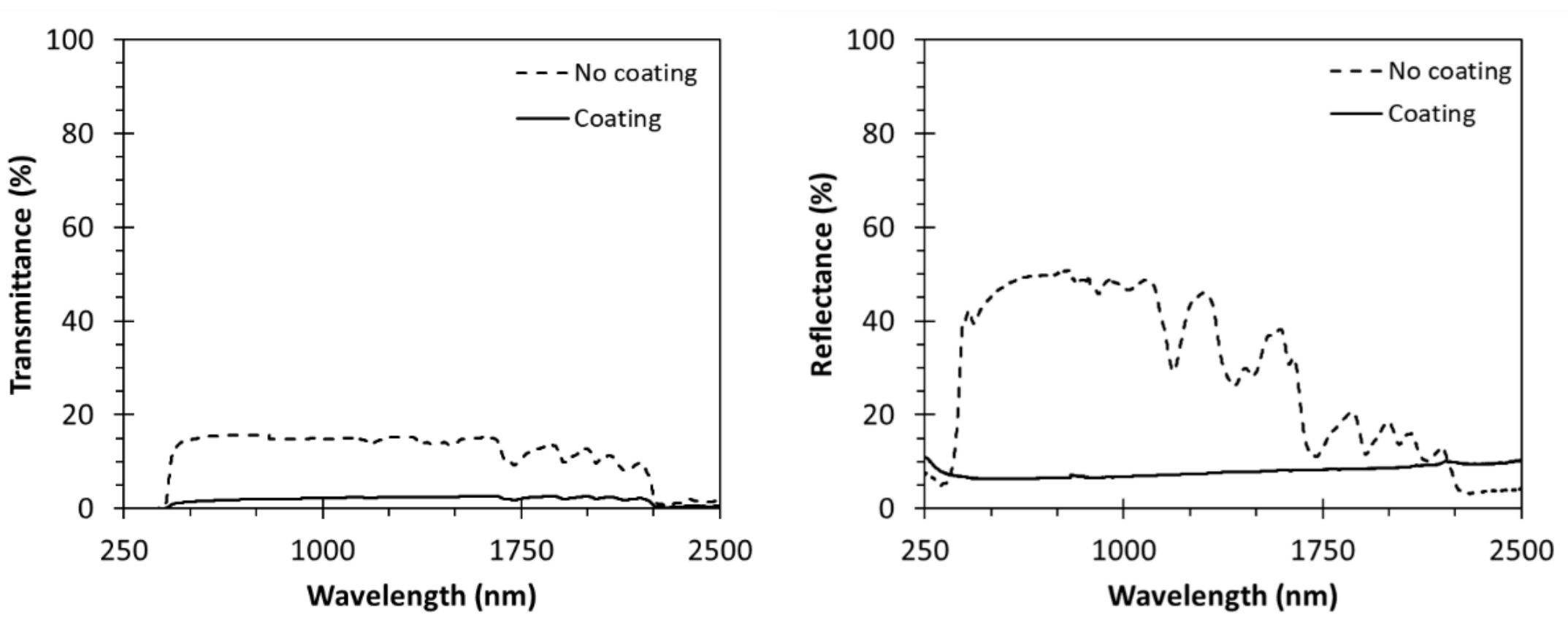

Figure S5. Transmittance and reflectance spectra of samples before and after graphite coating.

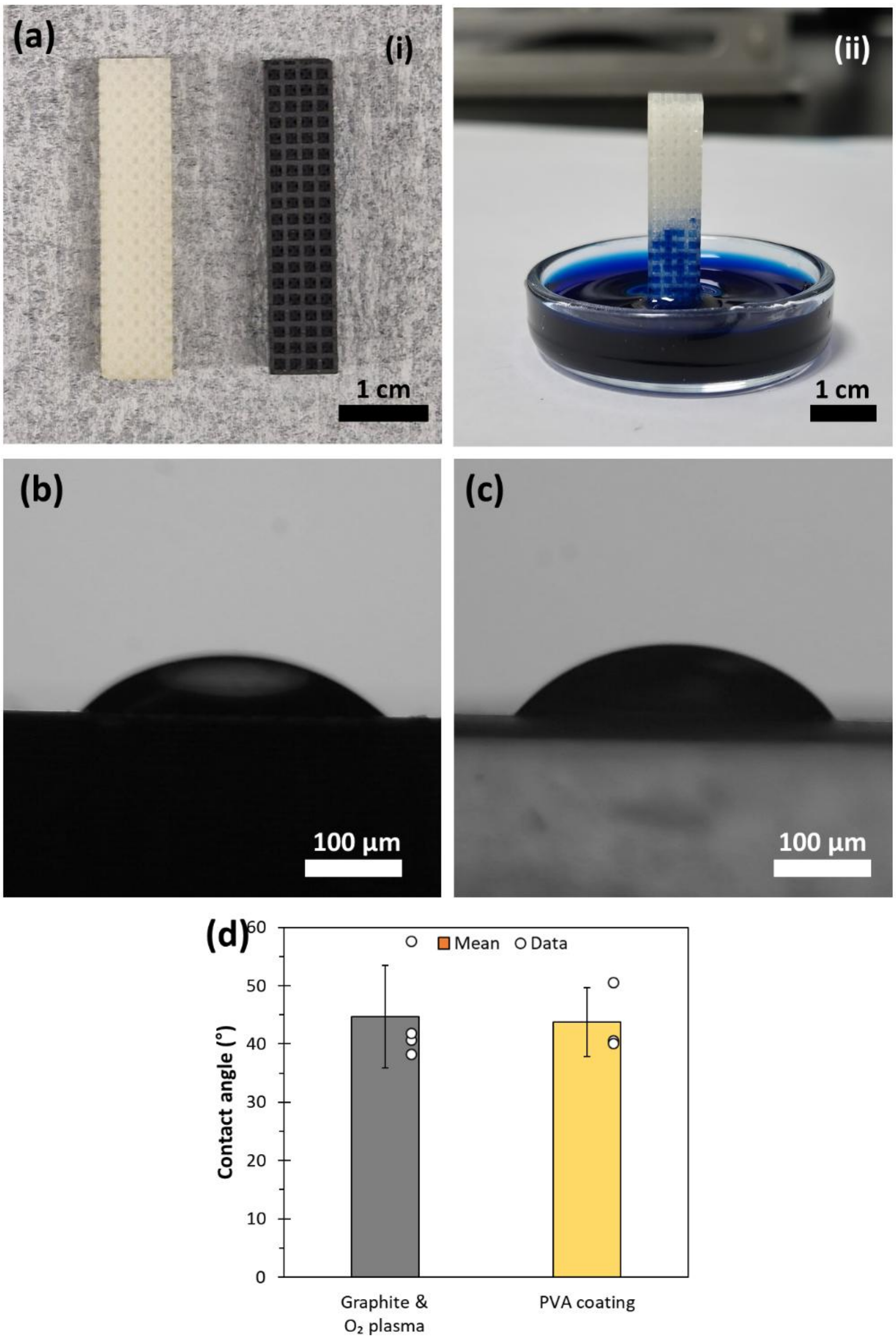

Figure S6. Rationale for using PVA-coated samples for water-transport visualization. (a) Optical images of (i) multicellular structures coated with PVA (left) and graphite only (right), and (ii) capillary-driven water transport in the PVA-coated sample. Contact angle images of (b) the graphite-coated surface after O_2 plasma treatment and (c) the PVA-coated surface. (d) Measured contact angles for each condition. Bars and symbols represent mean values and individual measurements, respectively. Error bars indicate standard deviation.

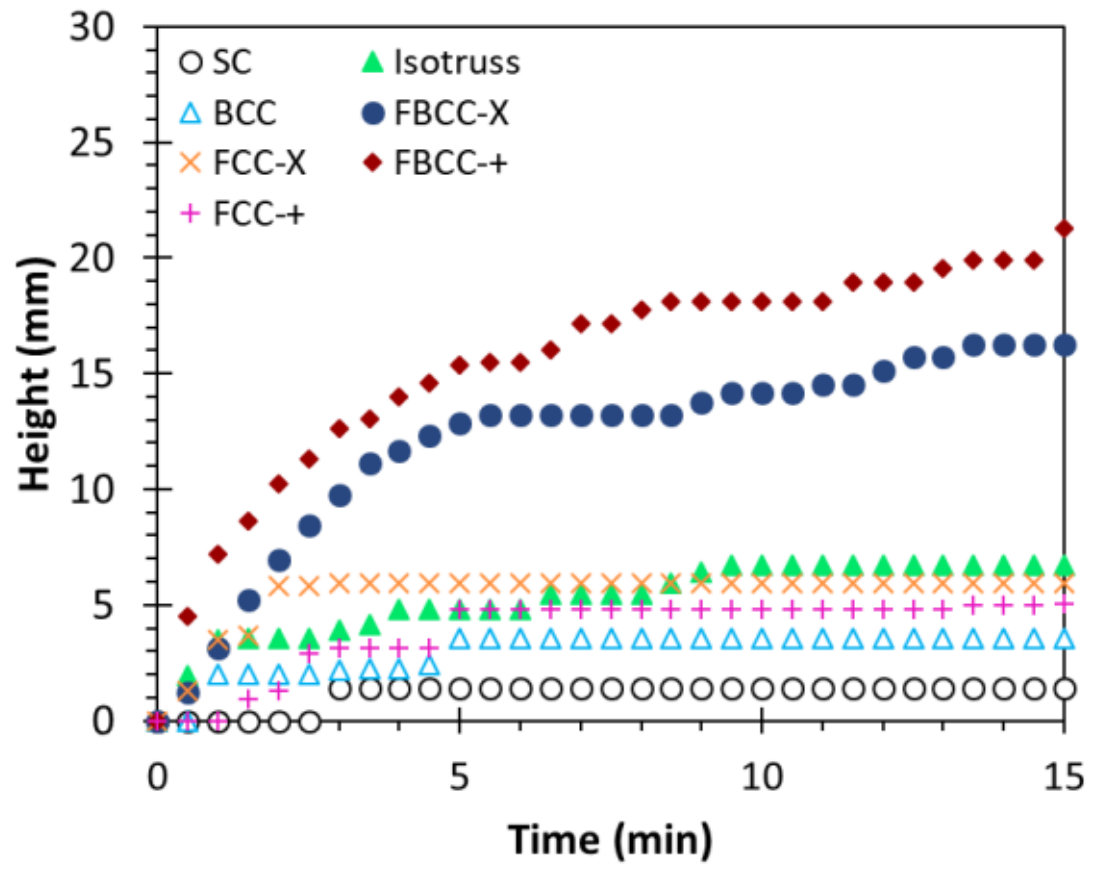

Figure S7. Time-dependent capillary rise behavior of different unit-cell structures.

Table S1. Geometric characteristics and capillary rise of different unit cell architectures

Unit cell	Dimensions* ($W \times L \times H$, mm)	Porosity**	Avg. contact perimeter*** (mm)	Capillary rise height**** (mm)
SC	2 × 2 × 2	0.65	3.85	1.39
BCC	2 × 2 × 2	0.54	7.43	3.57
FCC-X	2 × 2 × 2	0.39	8.22	5.91
FCC-+	2 × 2 × 2	0.45	8.11	5.04
Isotruss	2 × 2 × 2	0.47	8.52	6.70
FBCC-X	2 × 2 × 2	0.30	10.10	16.26
FBCC-+	2 × 2 × 2	0.34	11.75	21.31

*Single unit cell

**$\text{Porosity} = 1 - \frac{Solid\ Volume}{Total\ volume}$

***Average liquid–solid contact perimeter of single unit cell (normal to capillary direction)

****After 15 minutes

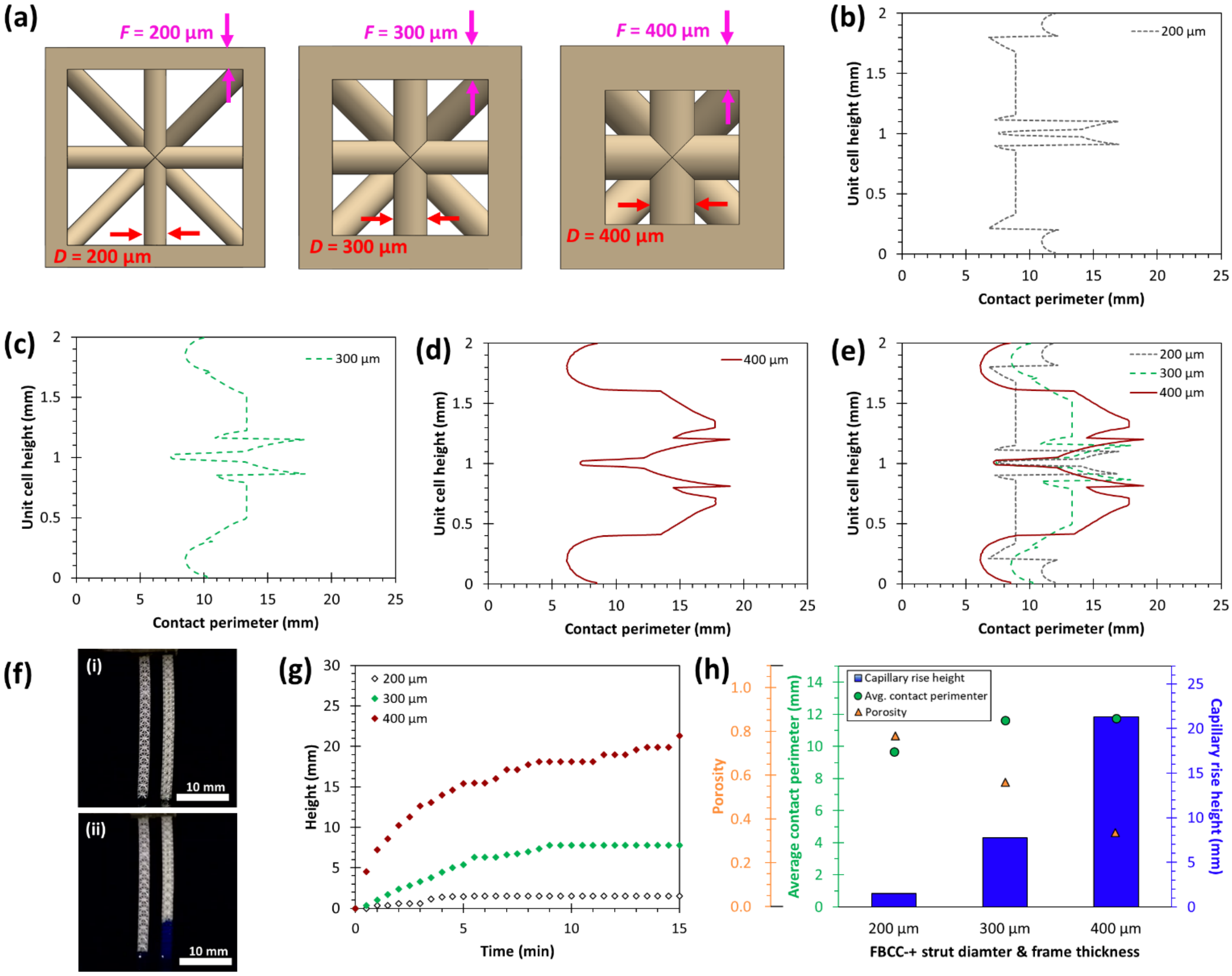

Figure S8. Capillary transport analysis FBCC-+ unit cells with varying frame thickness (*F*) and strut diameter (*D*), with constant unit-cell edge length of 2 mm. (a) Geometrical characteristics of the FBCC-+ structures. (b–d) Contact perimeter as a function of height within FBCC-+ unit cells with F and D of 200, 300, and 400 µm, respectively; (e) comparison of the three cases. (f) Time-dependent capillary rise images of 200 and 300 µm FBCC-+ cells at (i) initial state and (ii) after 15 min. (g) Corresponding time-dependent capillary rise behavior. (h) Comparative summary of porosity, average contact perimeter, and maximum capillary rise for the different unit-cell geometries.

Table S2. Geometric parameters and measured capillary rise of FBCC-+ unit cells with different frame thicknesses and strut diameters at a fixed unit-cell edge length of 2 mm.

Frame thickness (μm)	Strut diameter (μm)	Porosity*	Avg. contact perimeter** (mm)	Capillary rise height*** (mm)
200	200	0.78	9.67	1.52
300	300	0.57	11.62	7.76
400	400	0.34	11.75	21.31

*Porosity $= 1 - \frac{Solid\ Volume}{Total\ volume}$

**Average liquid–solid contact perimeter of single unit cell (normal to capillary direction)

***After 15 minutes

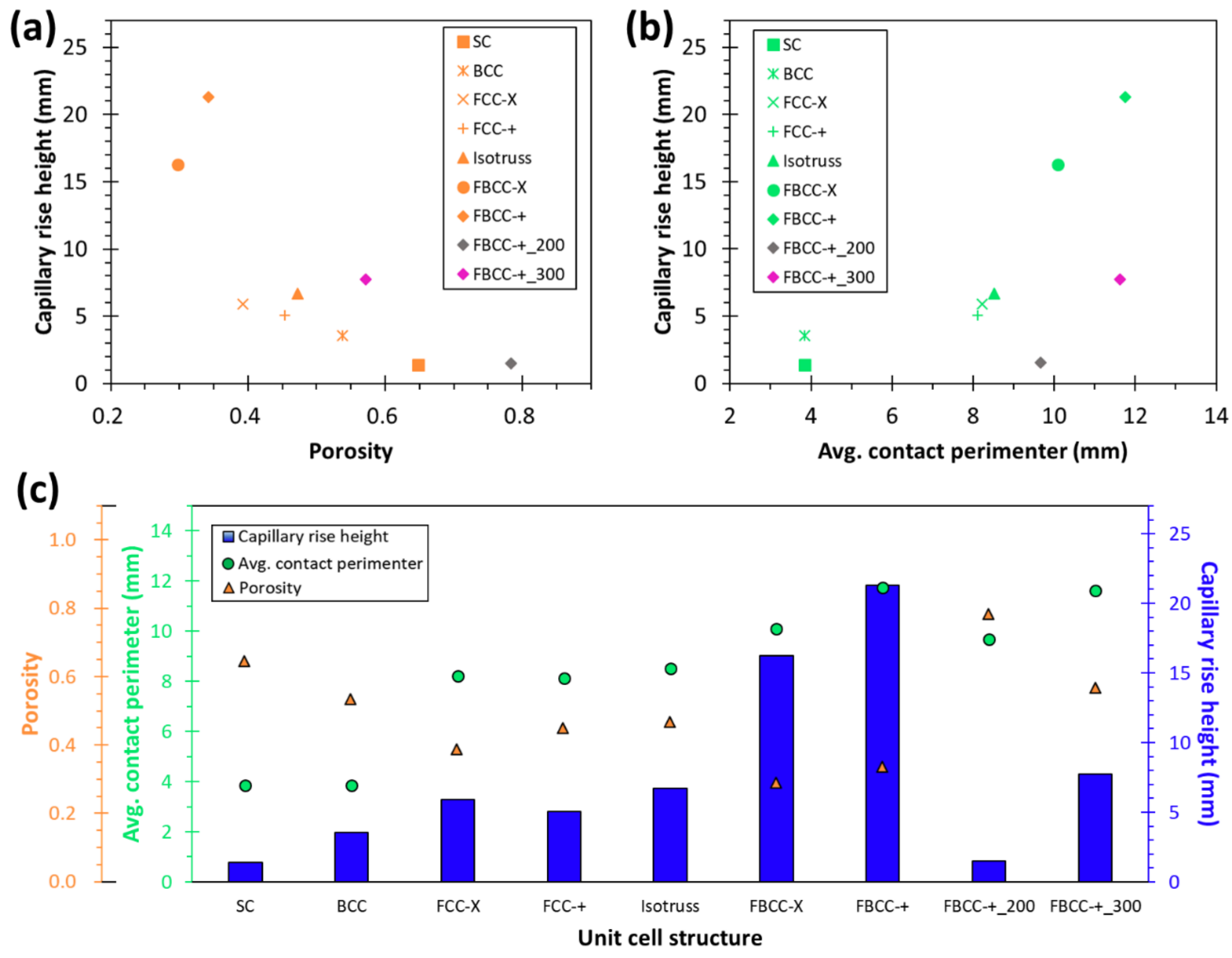

Figure S9. Correlation between geometrical characteristics and capillary performance of different unit cell structures. (a) Relationship between porosity and capillary rise height. (b) Relationship between average contact perimeter and capillary rise height. (c) Comparative summary of porosity, average contact perimeter, and maximum capillary rise for the different unit-cell geometries.

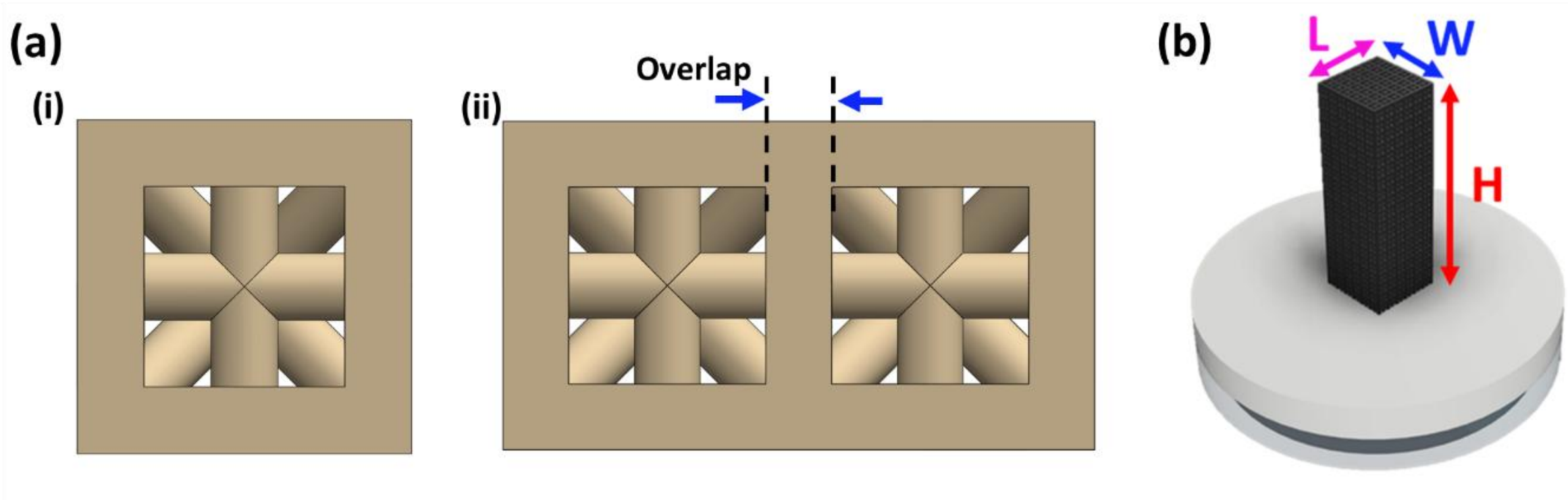

Figure S10. Schematic illustration of the geometrical characteristics of the MSE. (a) Front view of (i) single-cell and (ii) multicellular configurations, highlighting the overlapping region corresponding to the frame thickness in the multicellular structure. (b) Definition of geometric parameters of the MSE, including length (L), width (W), and height (H).

Table S3. Geometric parameters of MSEs at 0 cm exposed height.

Unit cell	Dimensions* ($W \times L$, cells)	Dimensions* ($W \times L$, cm)	Light illuminated area (cm^2)
BCC	6 × 6	1 × 1	1
Isotruss	6 × 6	1 × 1	1
FCC-X	6 × 6	1 × 1	1
FCC-+	6 × 6	1 × 1	1

*H = 0 for 0 cm exposed height case

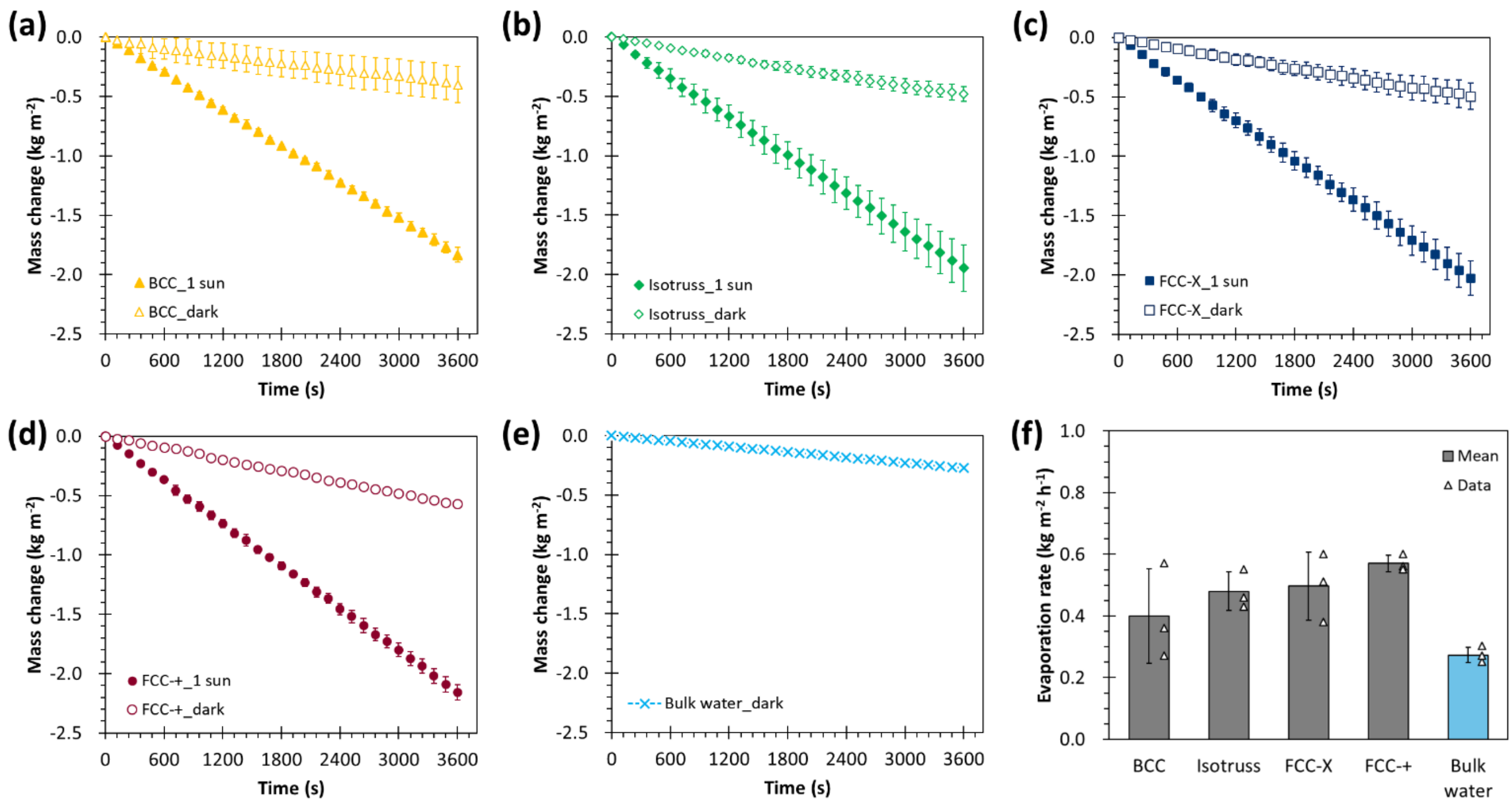

Figure S11. Mass change during evaporation at 0 cm exposed height. (a–d) BCC, Isotruss, FCC-X, and FCC-+ MSEs, respectively; (e) bulk water. (f) Evaporation rates under dark conditions. Bars and symbols in (f) represent mean values and individual measurements, respectively. Error bars indicate standard deviation.

Table S4. Light-harvesting area of different unit cell architectures

Unit cell	Light-harvesting area in a single unit cell (mm^2)	Light-harvesting area in MSE (mm^2)
SC	2.26	48.16
BCC	2.26	48.16
FCC-X	3.60	85.52
FCC-+	3.36	76.96
Isotruss	2.39	52.84
FBCC-X	3.60	85.52
FBCC-+	3.36	76.96

Light harvesting area: Projected solid area of the uppermost surface directly exposed to incident illumination.

MSE: 36 unit cells on 6 × 6 (width × length) cells configuration

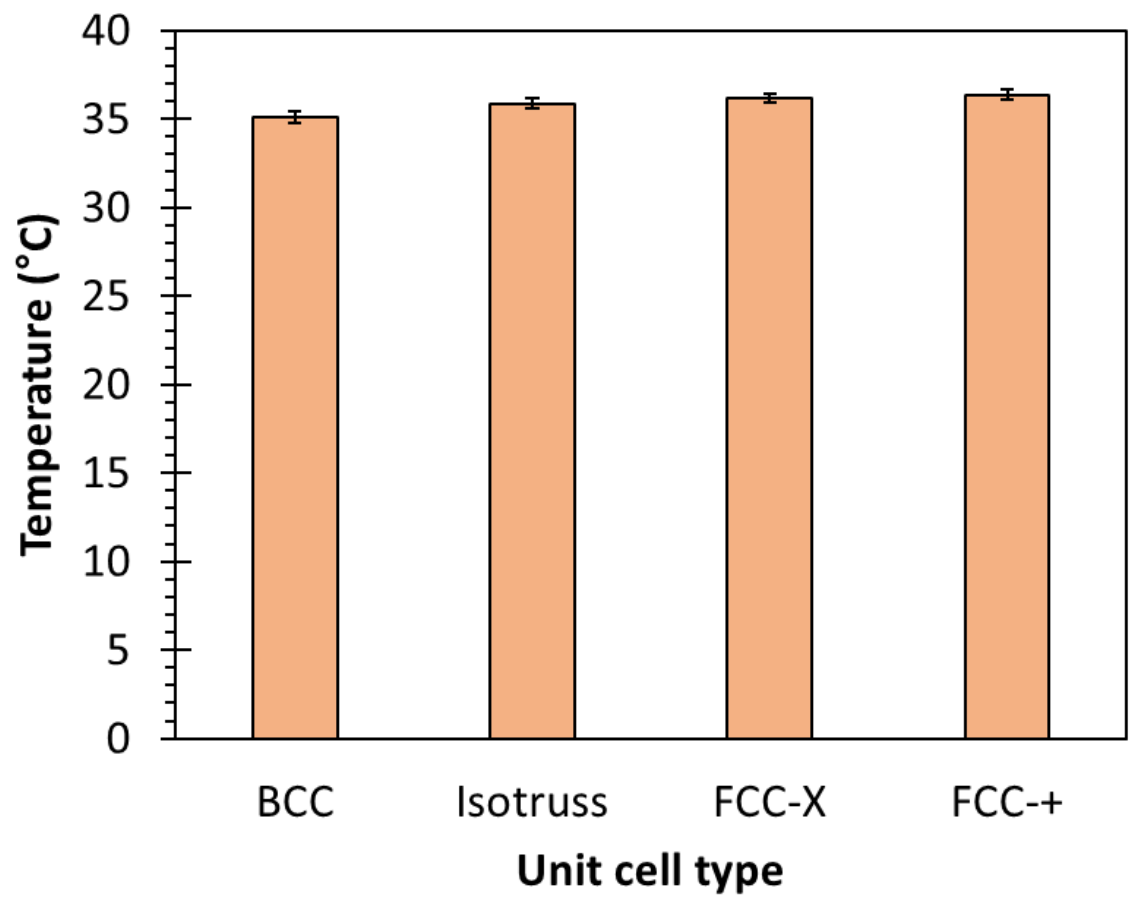

Figure S12. Time-averaged equilibrium surface temperature during evaporation for different unit-cell architectures.

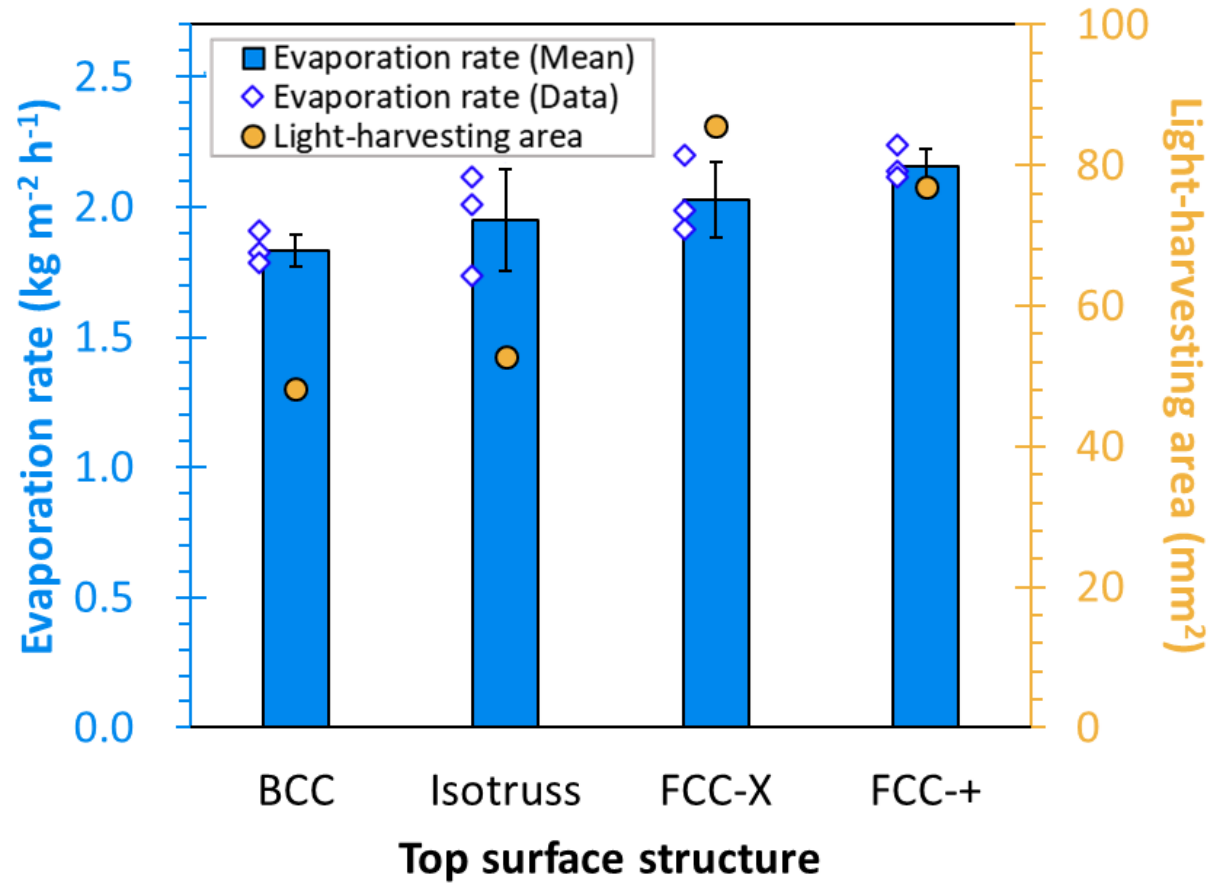

Figure S13. Correlation between evaporation rate and light-harvesting area. Bars and diamond symbols represent mean values and individual measurements, respectively, whit error bars indicating standard deviation.

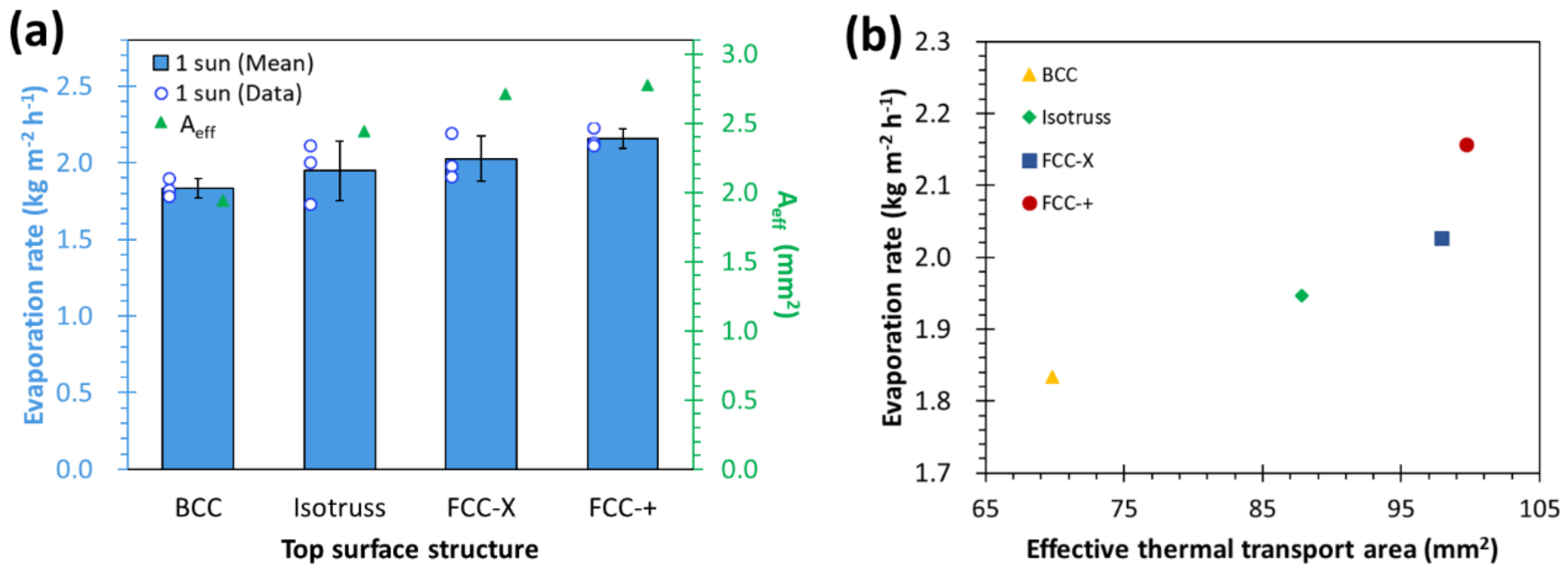

Figure S14. Correlation between evaporation rate and effective thermal transfer area. (a) Comparison of evaporation rates for different top-surface unit-cell architectures together with the effective thermal transport area (A_{eff}) of a single unit cell. Bars and circular symbols represent mean values and individual measurements, respectively. Error bars indicate standard deviation. (b) Relationship between evaporation rate and the total effective thermal transport area of the corresponding structures.

Table S5. Effective thermal transport area of different unit cell architectures

Unit cell	Effective thermal transport area* in a single unit cell (mm^2)	Effective thermal transport area in MSE (mm^2)
SC	1.93	69.12
BCC	1.94	69.84
FCC-X	2.72	97.92
FCC-+	2.77	99.72
Isotruss	2.44	87.84
FBCC-X	2.71	97.92
FBCC-+	2.77	99.72

*Calculated as $A_{eff} \approx P_{pore} \cdot H$; P_{pore} : pore perimeter, H : frame thickness

MSE: 36 unit cells on 6 × 6 (width × length) cells configuration

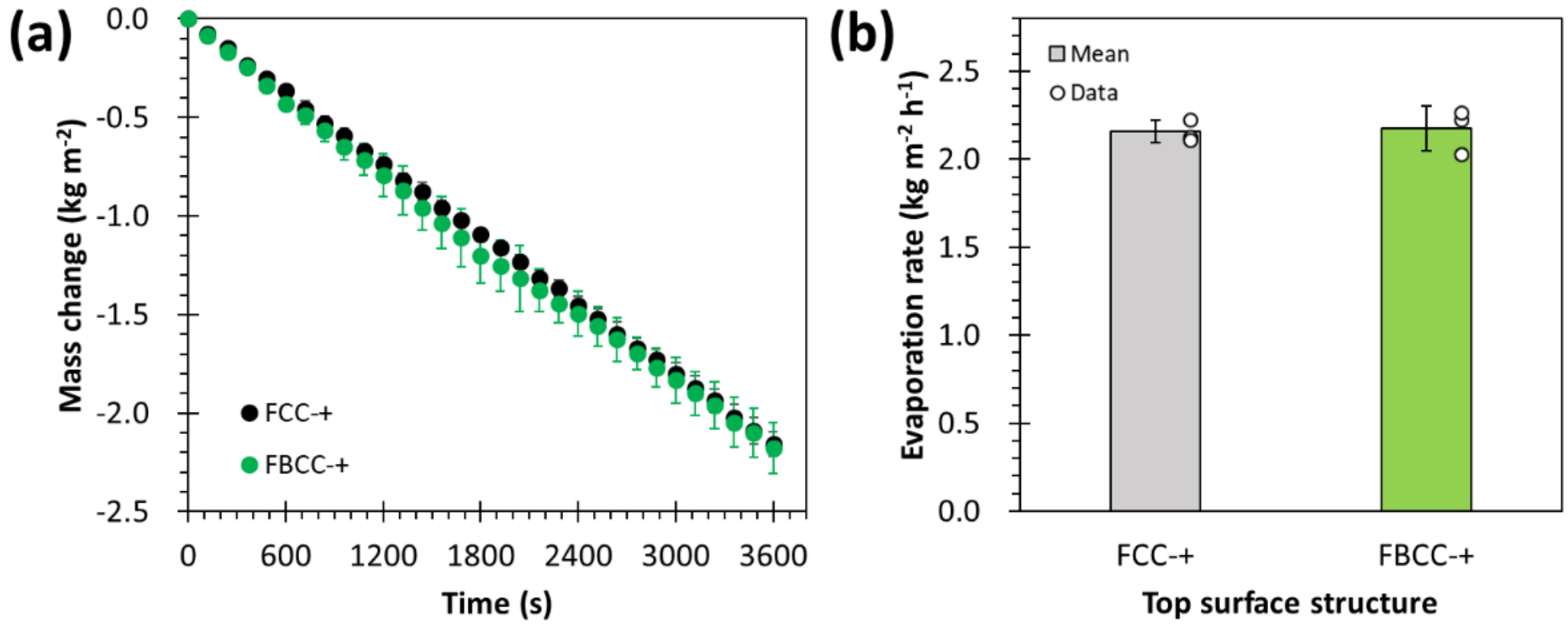

Figure S15. Evaporation performance analysis of FCC-+ and FBCC-+ MSE. (a) Mass change during evaporation, and (b) evaporation rate comparison. Bars and symbols in (b) represent mean values and individual measurements, respectively. Error bars indicate standard deviation.

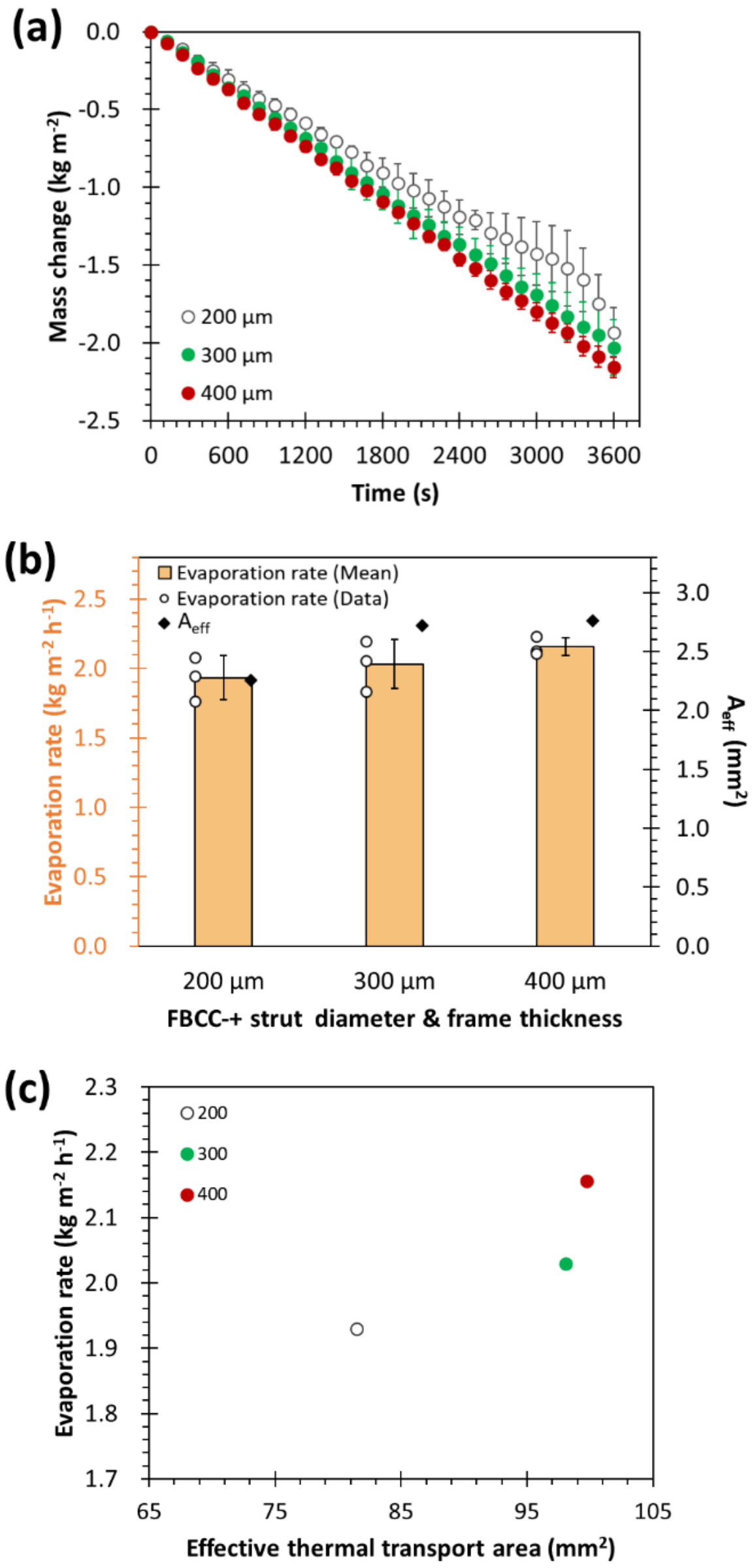

Figure S16. Evaporation performance analysis of FBCC-+ unit cells with varying frame thickness (F) and strut diameter (D), with constant unit-cell edge length of 2 mm on evaporation rate. (a) Mass change during evaporation, (b) Correlation between evaporation rate and effective thermal transfer area (A_{eff}) of a single unit cell, and (c) Relationship between evaporation rate and the total effective thermal transport area. Bars and circular symbols in (b) represent mean values and individual measurements, respectively. Error bars indicate standard deviation.

Table S6. Geometric parameters and evaporation rate of FBCC-+ unit cells with different frame thicknesses and strut diameters at a fixed unit-cell edge length of 2 mm.

Frame thickness (μm)	Strut diameter (μm)	Effective thermal transport area* in a single unit cell (mm^2)	Evaporation rate ($kg\ m^{-2}\ h^{-1}$)
200	200	2.26	1.93
300	300	2.72	2.03
400	400	2.77	2.16

*Calculated as $A_{eff} \approx P_{pore} \cdot H$; P_{pore} : pore perimeter, H : frame thickness

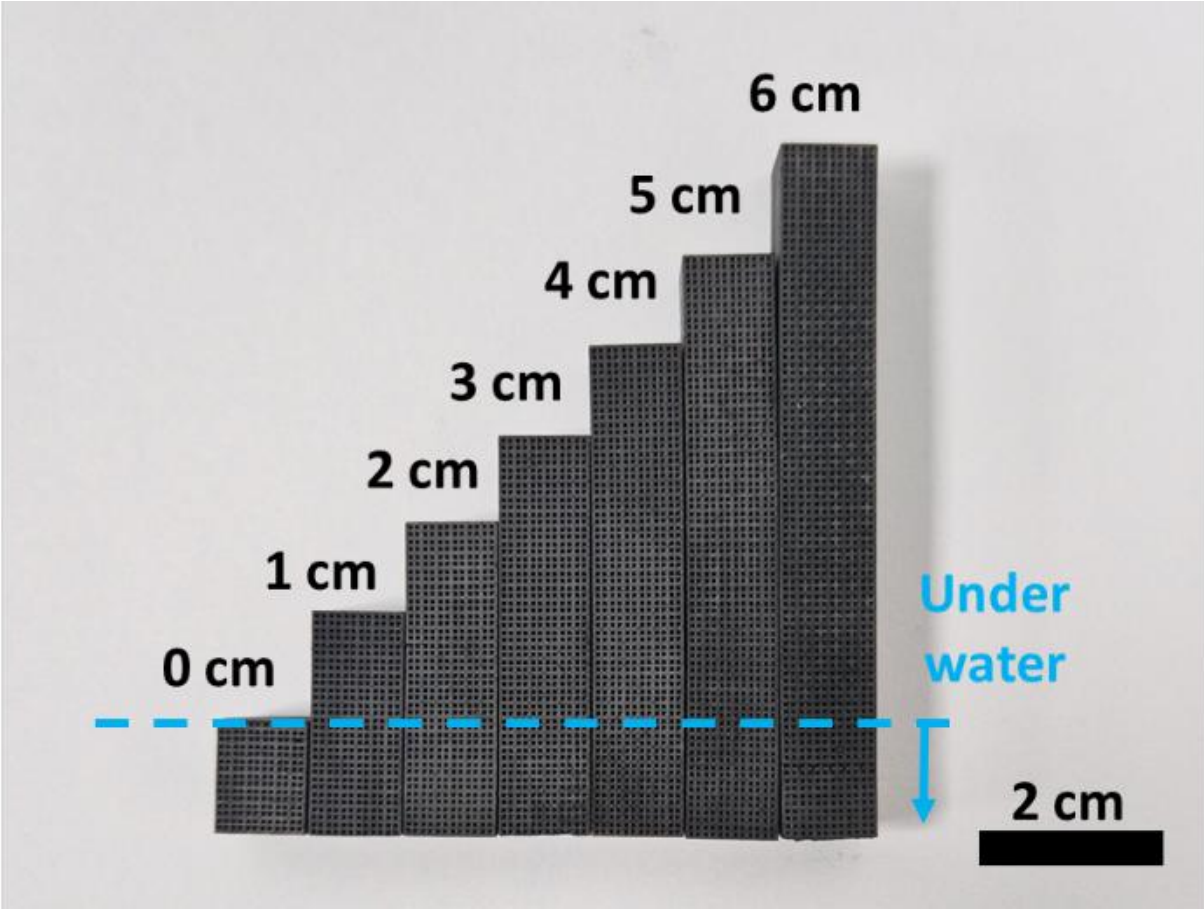

Figure S17. Optical images of FBCC-+ evaporators with varying heights.

Table S7. Geometric parameters of FBCC-+ MSEs at different exposed height.

MSE configuration	Dimensions* ($W \times L \times H$, cells)	Dimensions* ($W \times L \times H$, cm)	Light illuminated area (cm^2)
FBCC-+_0	6 × 6 × 0	1 × 1 × 0	1
FBCC-+_1	6 × 6 × 6	1 × 1 × 1	1
FBCC-+_2	6 × 6 × 12	1 × 1 × 2	1
FBCC-+_3	6 × 6 × 18	1 × 1 × 3	1
FBCC-+_4	6 × 6 × 24	1 × 1 × 4	1
FBCC-+_5	6 × 6 × 30	1 × 1 × 5	1
FBCC-+_6	6 × 6 × 36	1 × 1 × 6	1

*Exposed height refers to the portion of the structure extending above the water reservoir.

Table S8. Lateral surface contribution to evaporation performance of MSEs.

MSE configuration	Top surface area (cm^2)	Lateral surface area (cm^2)	ER_{total}* (kg m^{-2} h^{-1})	$ER_{lateral}$** (kg m^{-2} h^{-1})	Lateral contribution*** (%)
FBCC-+_0	1	0	2.16	-	-
FBCC-+_1	1	4	3.69	1.53	41.46
FBCC-+_2	1	8	4.80	2.64	55.00
FBCC-+_3	1	12	5.59	3.43	61.36
FBCC-+_4	1	16	6.46	4.30	66.56
FBCC-+_5	1	20	6.90	4.74	68.70
FBCC-+_6	1	24	6.30	4.14	65.69

*Total evaporation rate.

**Evaporation rate contributed by lateral surfaces, calculated as $ER_{lateral} = ER_{total} - ER_{0cm}$

***Lateral contribution $= \frac{ER_{lateral}}{ER_{total}} \times 100$

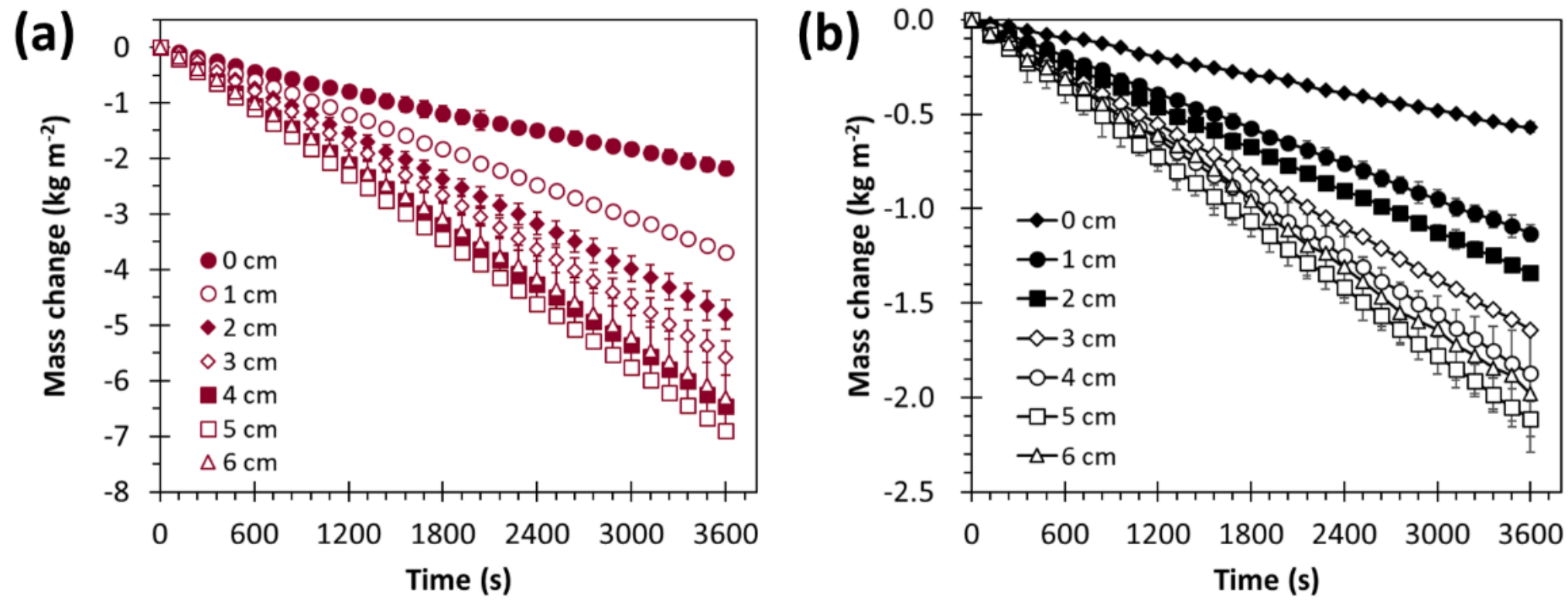

Figure S18. Mass change during evaporation at controlled exposed height. (a) Under 1 sun illumination and (b) under dark condition.

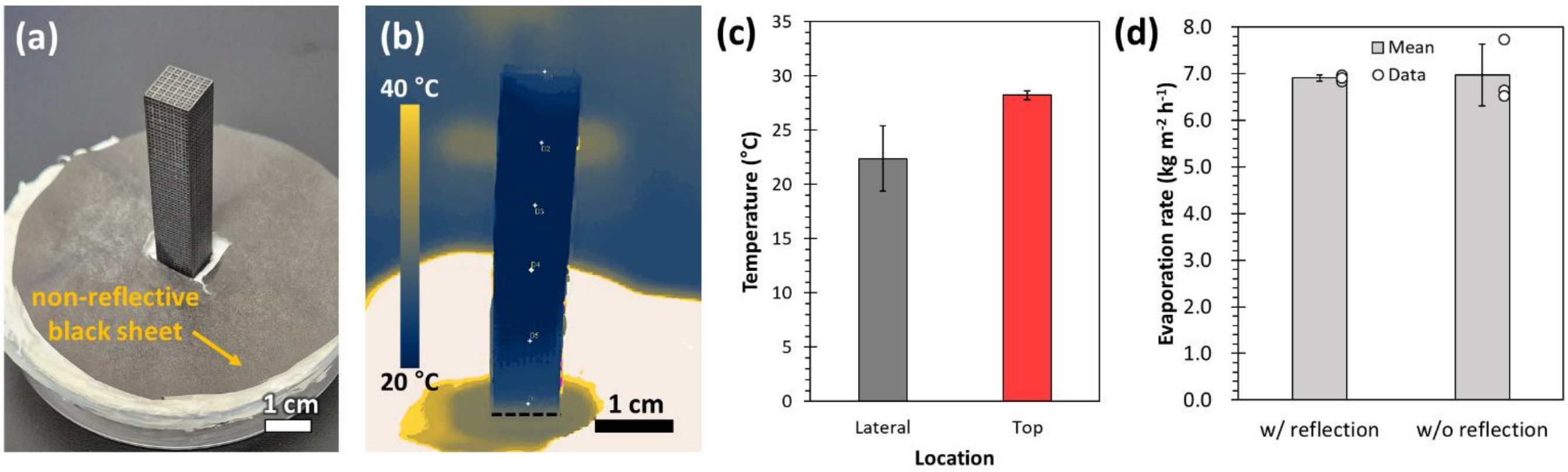

Figure S19. Experiments to identify the effect of partial light reflection from the polystyrene (PS) foam. (a) Optical image of the experimental setup incorporating a non-reflecting sheet on the PS foam. (b) Infrared thermal images of FBCC-+_5 during evaporation with non-reflecting sheet incorporated. (c) Time-averaged temperatures of the lateral and top surfaces during evaporation under 1 sun irradiation; error bars indicate the standard deviation of the temperature measured at 1 min intervals. (d) Comparison of evaporation rates with and without PS foam reflection. Bars and symbols in (d) represent mean values and individual measurements, respectively. Error bars indicate standard deviation.

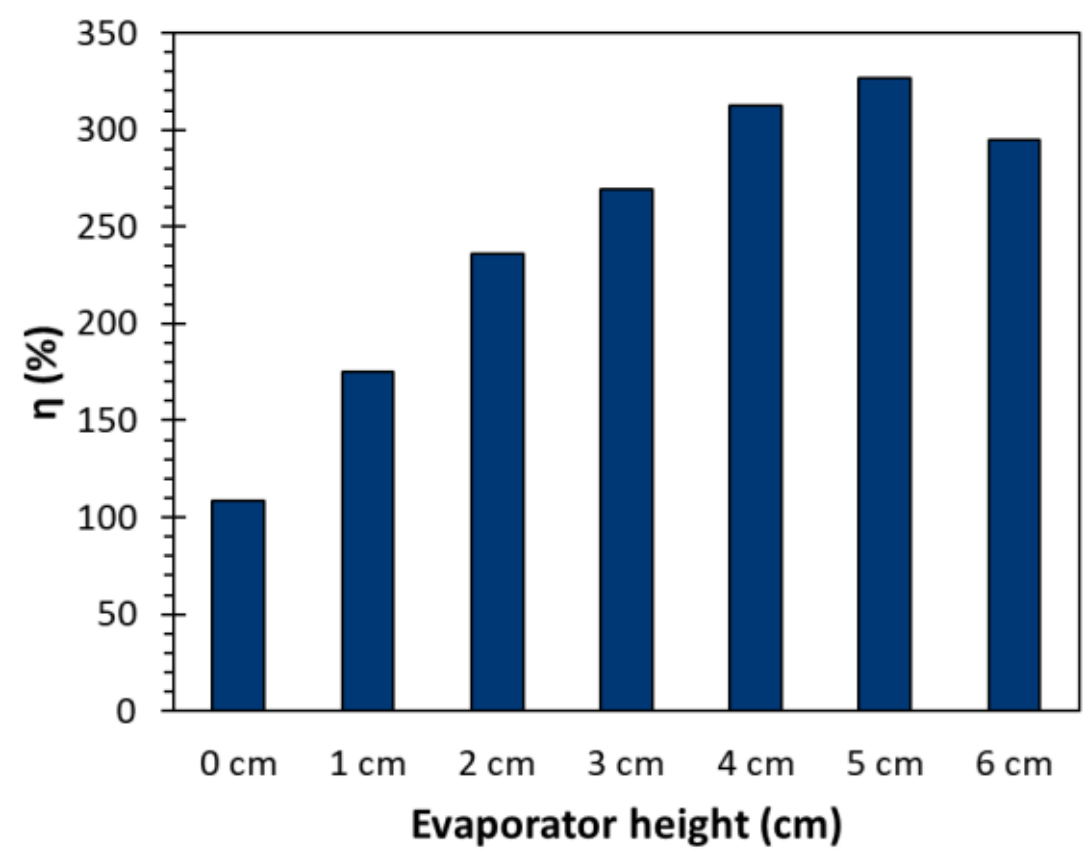

Figure S20. Apparent energy utilization efficiency of FBCC-+ evaporators with different heights.

Notably, the apparent energy utilization efficiency for the 0 cm configuration exceeds unity even in the absence of lateral evaporation. This behavior arises from the elevated interfacial temperature (~35 °C) combined with the small projected evaporation area (1 cm^2), which promotes efficient vapor removal from the entire surface. Under these conditions, the vapor boundary layer is continuously refreshed, suppressing local humidity buildup and enhancing the evaporation flux. Similar behavior has been reported in small-area planar evaporators, where efficient boundary-layer renewal prevents the formation of evaporation dead zones and yields unusually high area-normalized evaporation rates[1,2]. This mass-transfer enhancement allows additional environmental heat uptake even in nominally planar configurations, resulting in apparent efficiencies exceeding 100%.

Table S9. Apparent energy utilization efficiency of different solar evaporators find in literature

Solar evaporator	Configuration	Apparent energy utilization efficiency (%)	Ref.
rGO foam	2D	99.4	[3]
Janus CB-PDMS/PMF	2D	75	[4]
PC@ PPy porous membrane	2D	93.57	[5]
PDA/CB@MS salt crystallization system	2D	93	[6]
NC@Cu core–shell nanostructure	2D	137.1	[7]
Umbrella evaporator	3D	85.5	[8]
3D solar crystallizer	3D	94.3	[9]
Biomimetic 3D solar evaporator	3D	98.9	[10]
ATSC	3D	175	[11]
Pyramid-shaped solar evaporator	3D	186.76	[12]
Volcanic evaporator	3D	220.34	[13]
FBCC-+_5	3D	326.9	This study

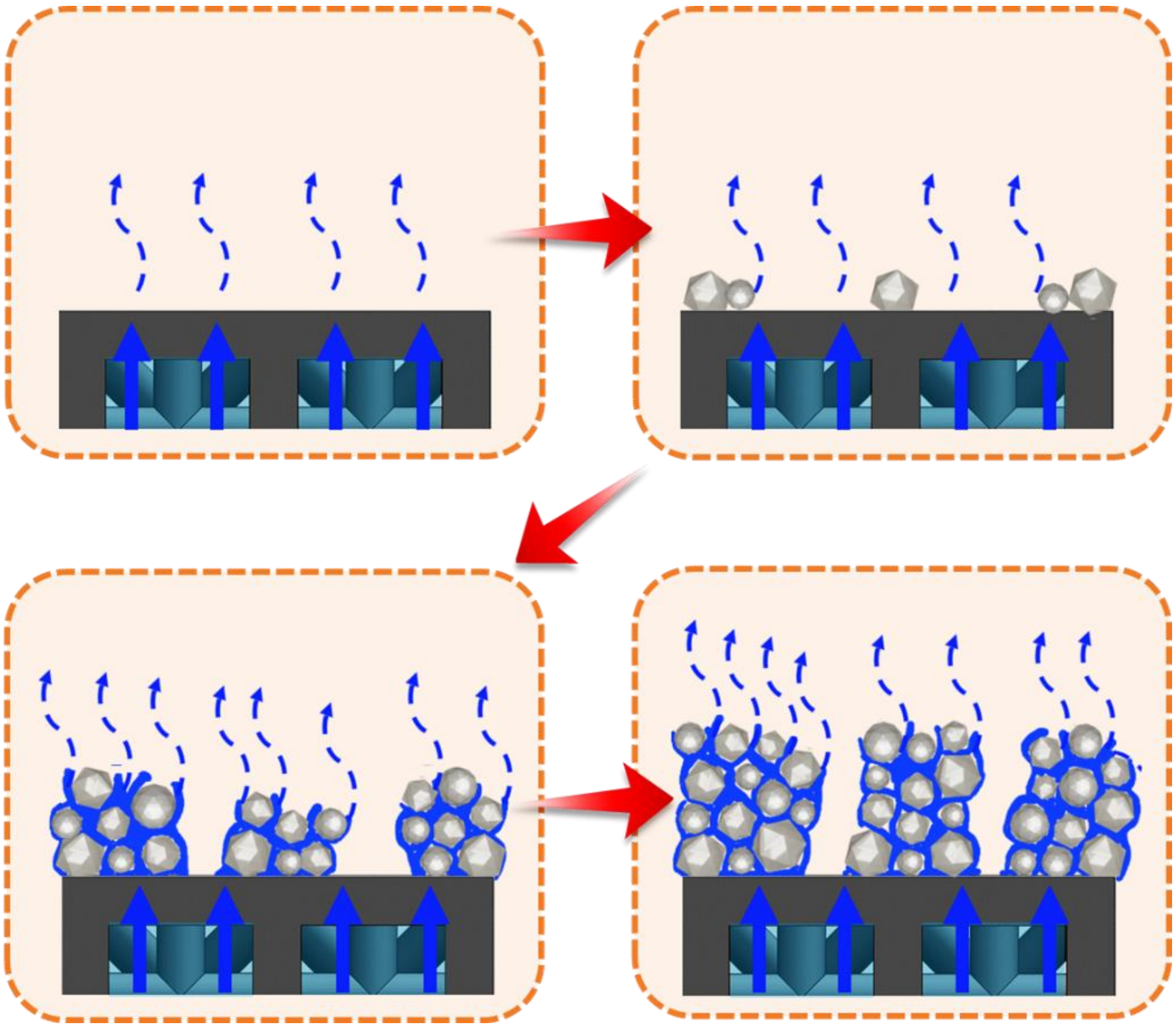

Figure S21. Schematic image illustrating the formation process of porous wet salts crystals on the surface of the MSE during continuous solar desalination.

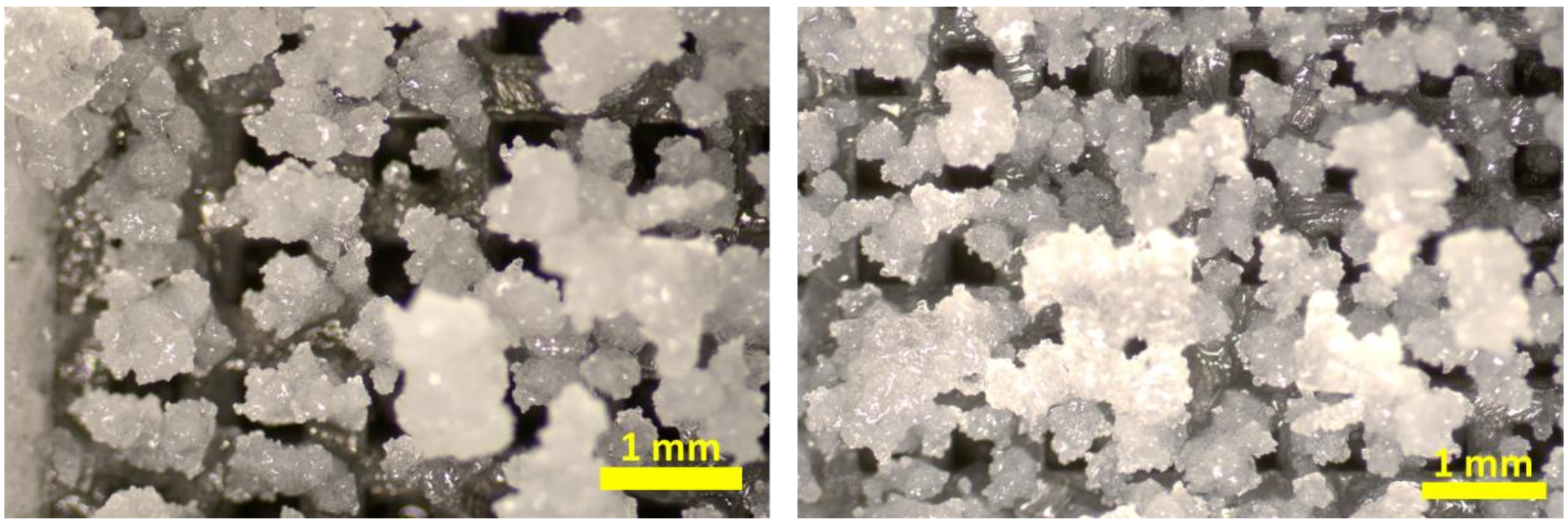

Figure S22. Optical images of wet salt crystals formed during long-term solar evaporation in 20 wt% NaCl solution.

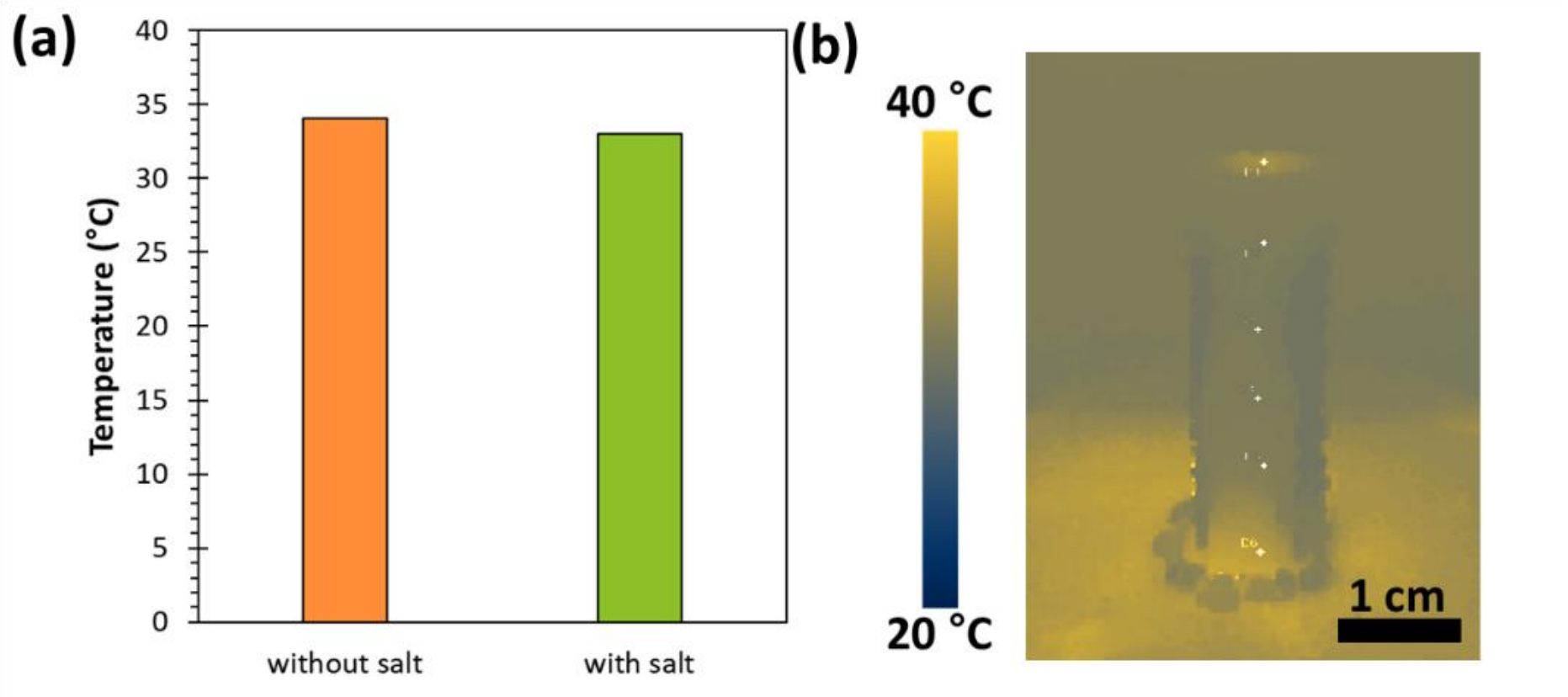

Figure S23. Thermal behavior of salt-covered FBCC-+_5 during evaporation. (a) Comparison of surface temperature profiles with and without salt coverage at the top surface; (b) infrared thermal images of the evaporator under salt-covered conditions.

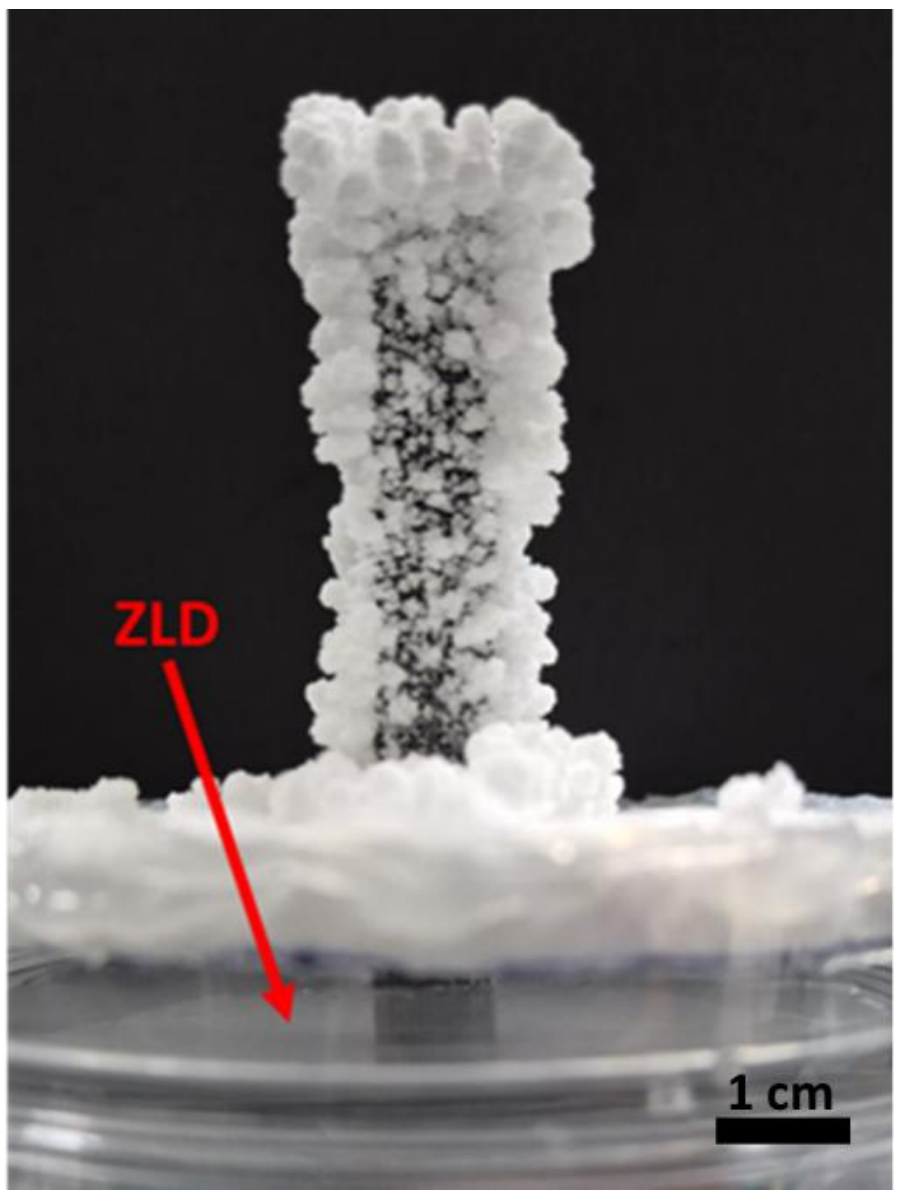

Figure S24. Optical image of FBCC-+_5 after complete evaporation of the liquid reservoir.

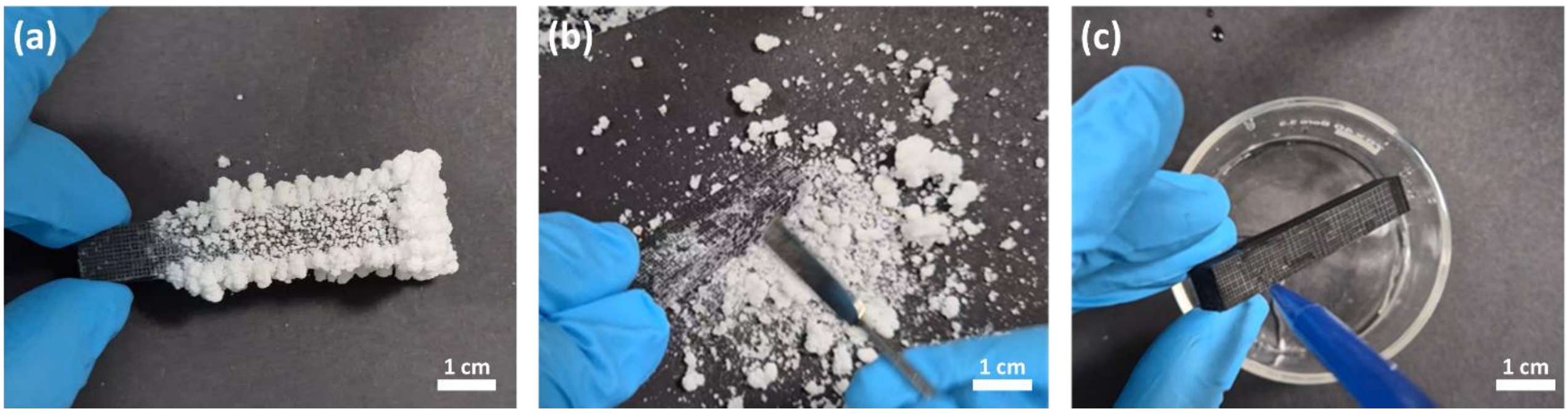

Figure S25. Salt collection and regeneration process following long-term desalination. (a) FBCC-+_5 after prolonged operation, showing localized NaCl crystal accumulation. (b) Mechanical salt recovery by gentle scraping. (c) Water rinsing to remove residual salts.

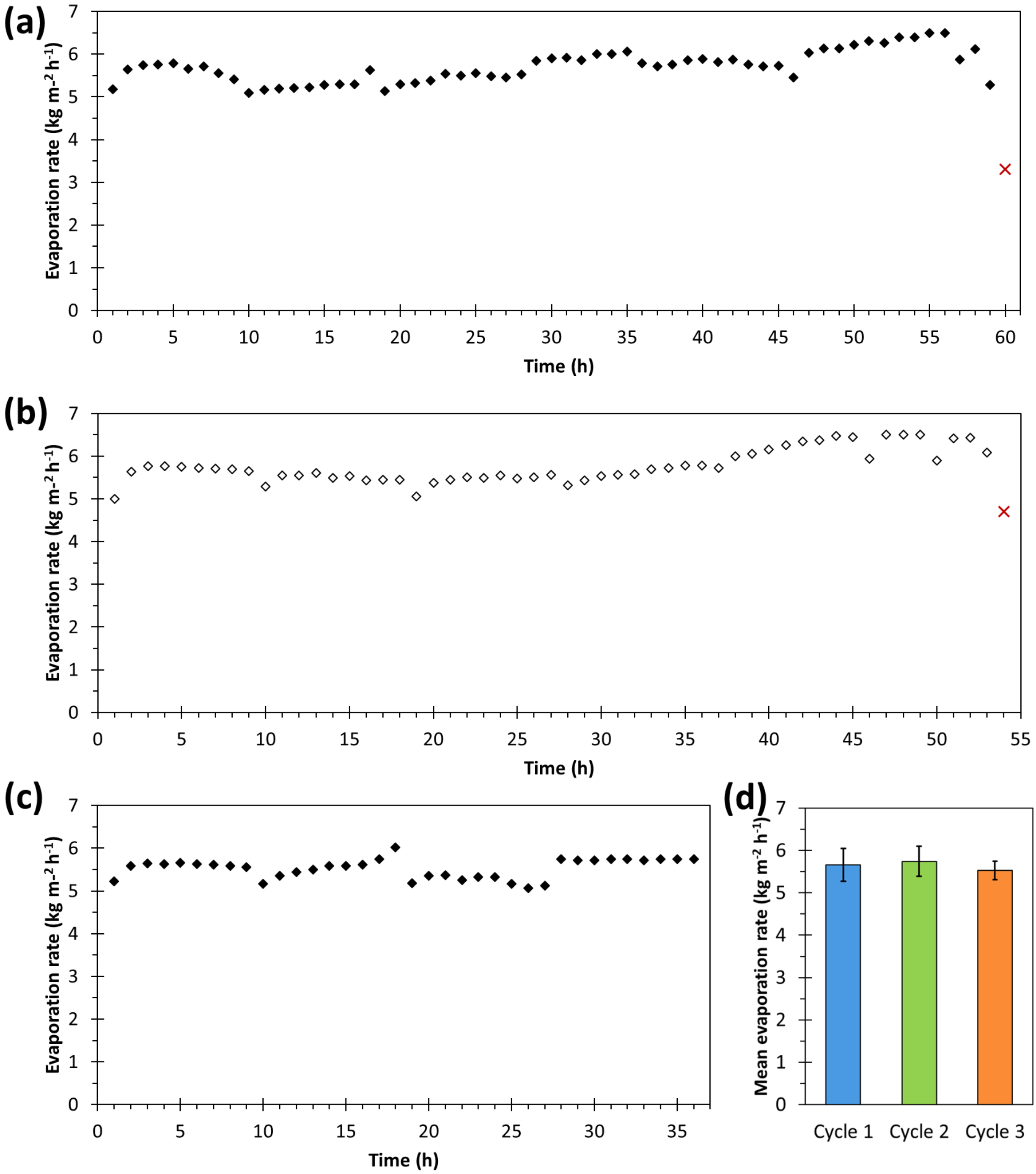

Figure S26. Long-term cycling performance of FBCC-+_5 after repeated salt removal and cleaning. Evaporation rate during (a) first cycle, (b) second cycle, (c) third cycle and (d) time-averaged evaporation rate in each cycle. The final cycle was terminated prior to reaching ZLD conditions, as a stable evaporation rate was maintained throughout operation.

Table S10. Geometric parameters of cup-shaped hybrid evaporator.

**Dimensions*** **(*W* × *L* × *H*, cells)**	**Dimensions*** **(*W* × *L* × *H*, cm)**	**Light illuminated area (cm^2)**
8 × 8 × 12	1.32 × 1.32 × 2	1.74

*Exposed height refers to the portion of the structure extending above the water reservoir.

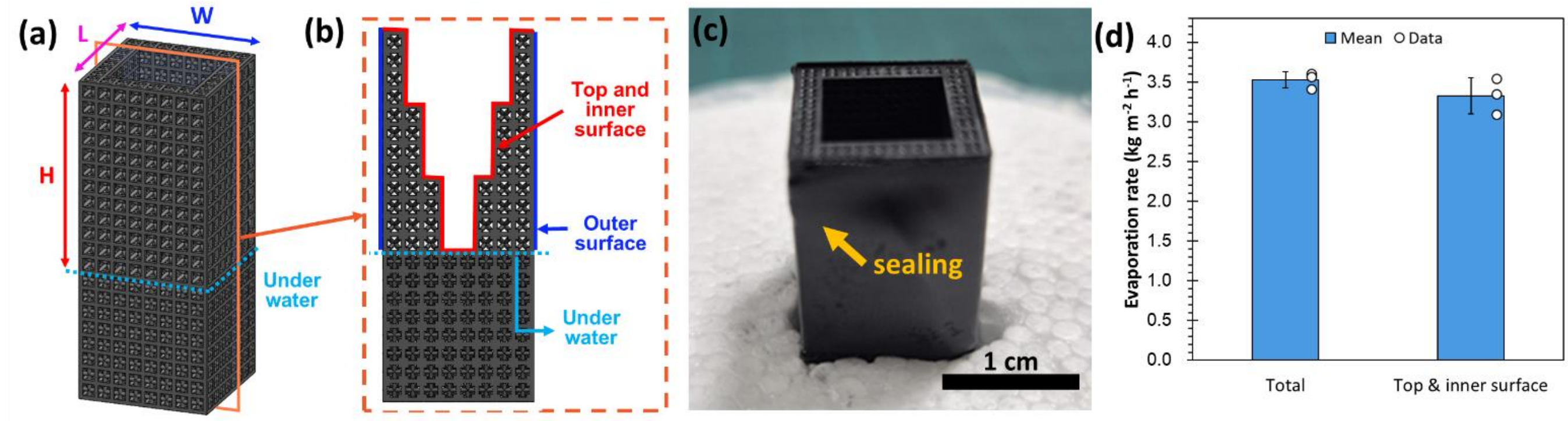

Figure S27. Geometry and evaporation analysis of the cup-shaped hybrid evaporator. (a) Schematic illustration of the geometrical configuration. (b) Cross-sectional view highlighting the outer region (BCC) and the top and internal surfaces (FBCC-+). (c) Optical images of the experimental setup used to isolate evaporation occurring exclusively from the internal surfaces. (d) Comparison of evaporation rates under total (uncovered) and top and inner surface-only evaporation conditions (outer surface sealed). The measured evaporation rates were used as input parameters for the numerical simulations. Bars and symbols represent mean values and individual measurements, respectively. Error bars indicate standard deviation.

Table S11. Salt recovery efficiencies of different MSEs.

Evaporator	Salt (conc.)	Initial (g)	Recover (g)	Recovery efficiency* (%)
FBCC-+_5	NaCl (20 wt%)	9.49	9.01	95.85
		9.05	8.54	94.32
Hybrid cup	NaCl (20 wt%)	20.89	20.36	97.49

$$^{*}\text{Recovery efficiency }(\%) = \frac{Recovered\ amount\ (g)}{Initial\ amount\ (g)} \times 100$$

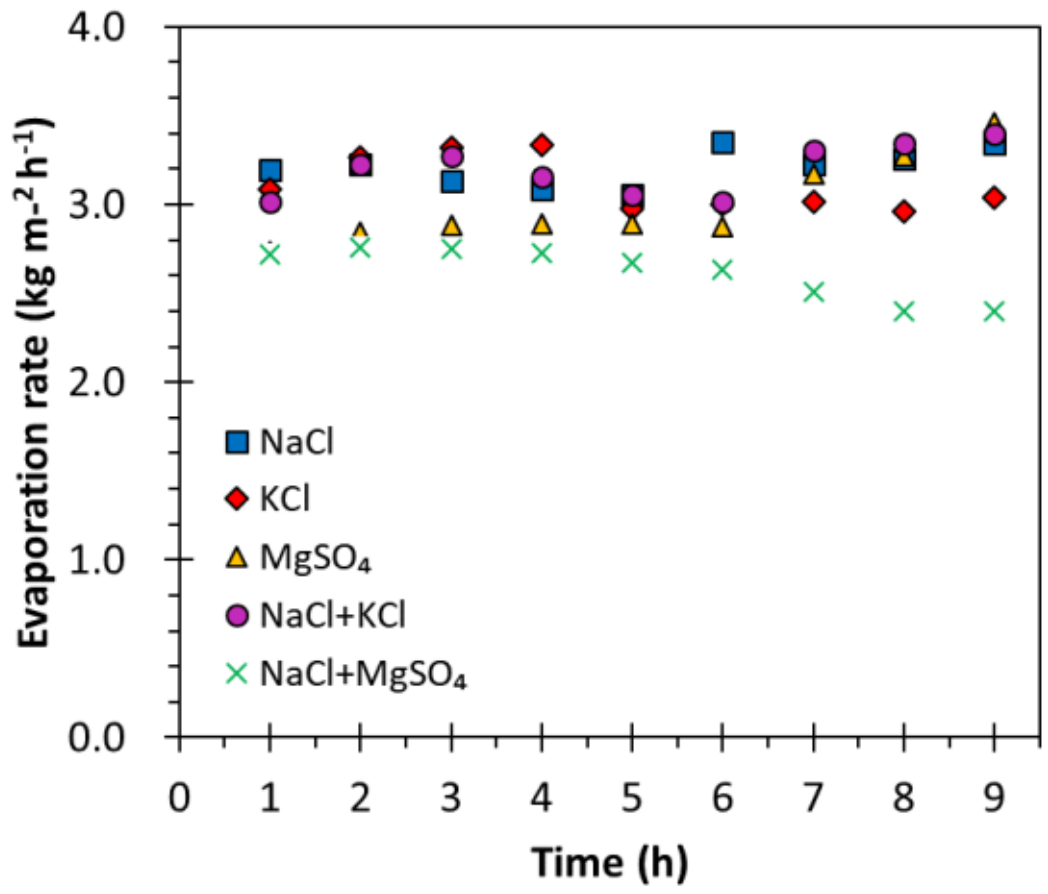

Figure S28. Long-term evaporation behavior under various high-concentration salt solutions.

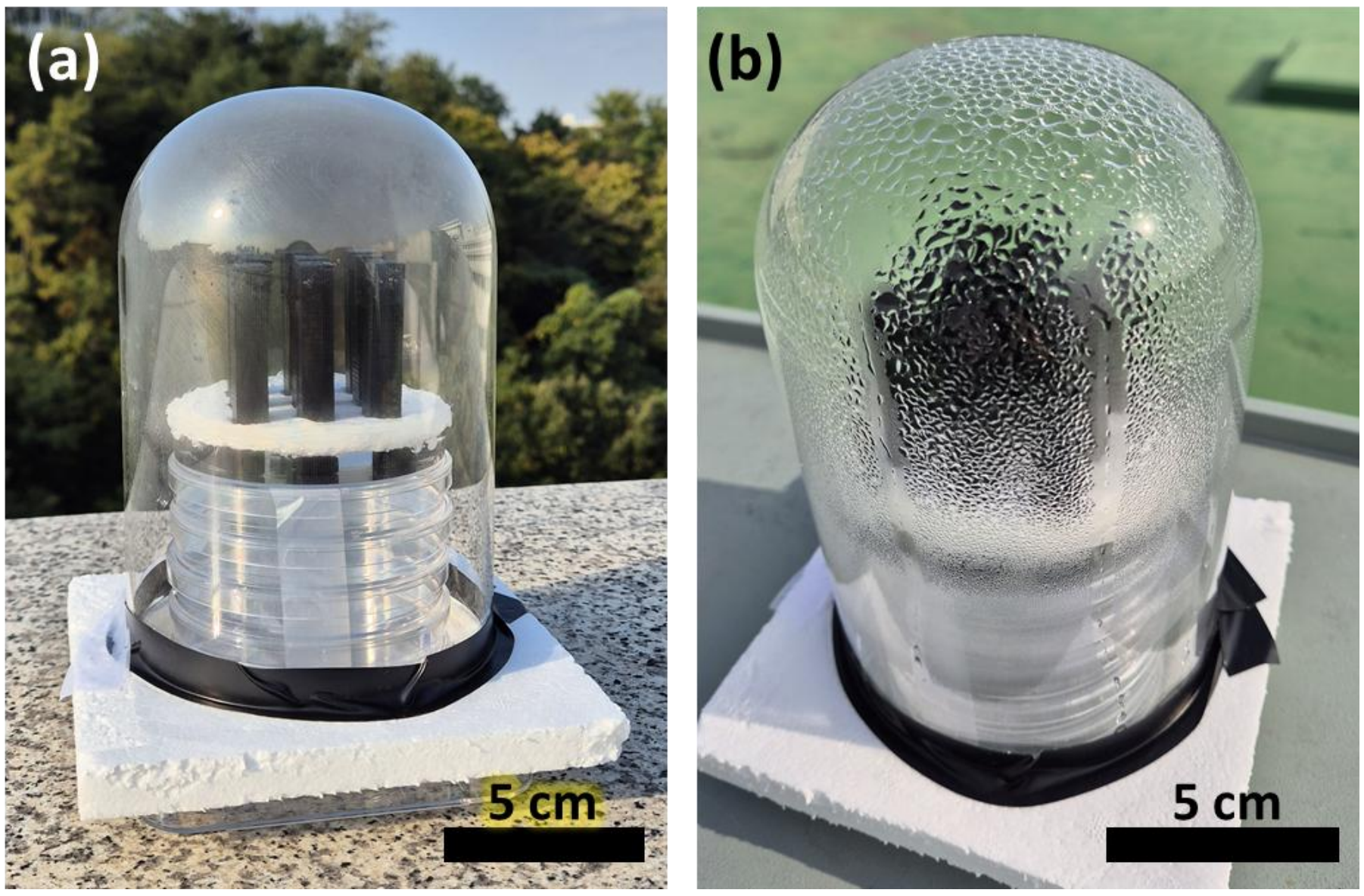

Figure S29. Optical images recorded during outdoor desalination experiments. (a) Initial state of the system prior to operation and (b) during desalination, showing progressive condensation and droplet formation on the inner surface of the glass cover.

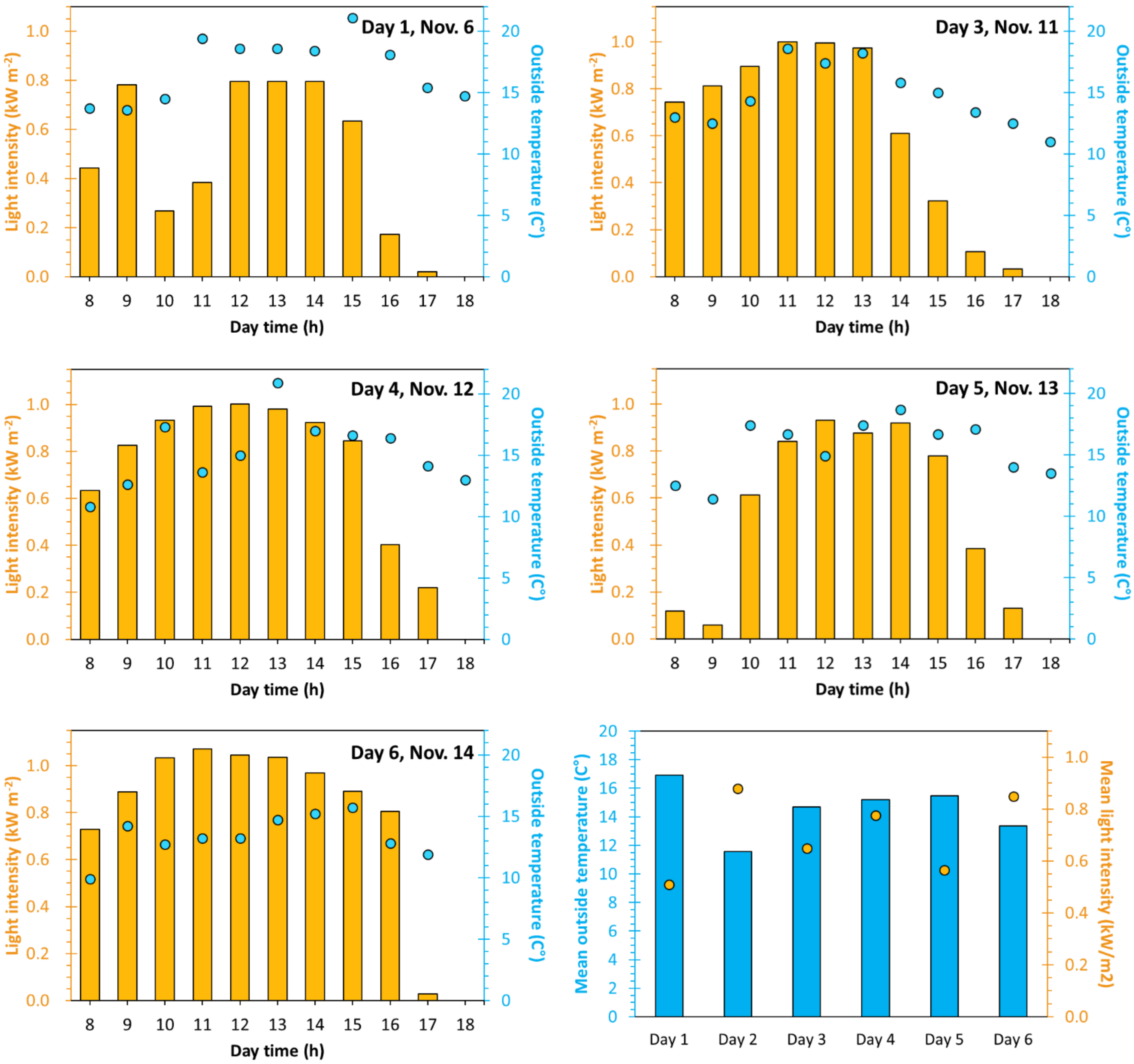

Figure S30. Time-resolved solar irradiance and ambient temperature during outdoor tests on 2025.

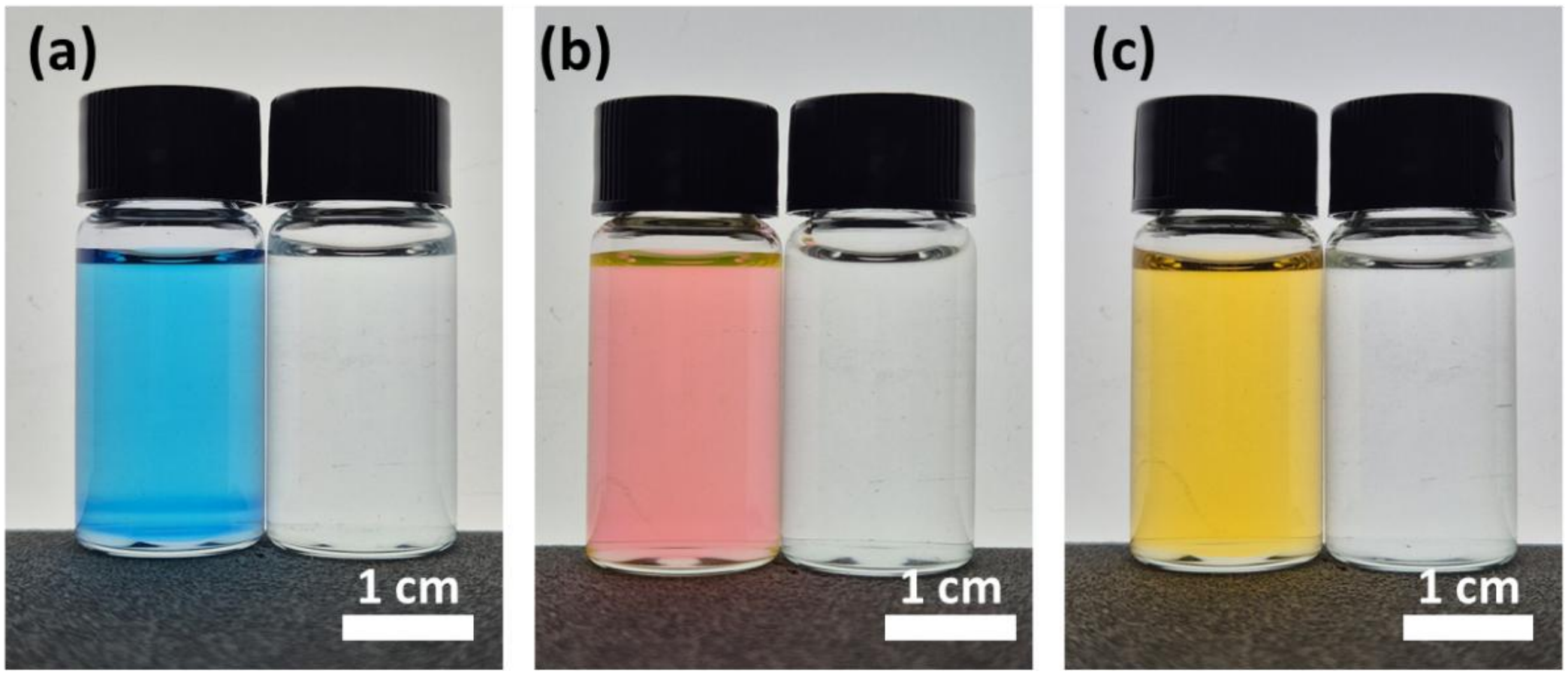

Figure S31. Optical images of dye solutions before and after solar evaporation and water collection under 1 sun irradiation: (a) methylene blue, (b) rhodamine 6G, and (c) methyl orange.

Table S12. Comparison of recent resin- and filament-based 3D-printed solar evaporators.

Base material	Fabrication method	Dimensions (area × height, $cm^2 \times cm$)	Evaporation rate* ($kg\ m^{-2}\ h^{-1}$)	Maximum water yield** ($kg\ m^{-2}\ day^{-1}$)	Ref
Acryl resin	DLP 3D printing	4 × 5	3.5	34.4	[14]
Acryl resin	DLP 3D printing	0.13 × 1.5	2.32	14.8	[15]
Acryl resin	DLP 3D printing	Not reported	1.64	2.5	[16]
Resin	DLP 3D printing	0.95 × 2.2	2.63	15.48	[10]
Resin	DLP 3D printing	1 × 1.2	3.18	20.52	[17]
Acryl resin	DLP 3D printing	7.07 × 1.75	2.13	9.95	[18]
Homemade resin	DLP 3D printing	3.14 × 2	2.249	12.23	[19]
Acryl resin	PμSL 3D printing	0.62 × 0.6	5.03	35.1	[20]
Filament	FDM 3D printing	1.77 × 1.5	4.02	22.27	[13]
Filament	FDM 3D printing	1.30 × 2	3.54	24.93	[12]
Resin	DLP 3D printing	7.07 × 3	4.08	15.1	[21]
Acryl resin	DLP 3D printing	1 × 5	6.9	47.68	This study

*Under constant 1 sun illumination, **Outdoor condition

DLP: Digital Light Processing

FDM: Fused Deposition Modeling

PμSL: Projection Micro-Stereolithography

"Not reported" indicates that the specific dimension was not provided in the original reference. When the illuminated area was not explicitly reported, it was calculated based on the available geometrical dimensions, such as the lateral length or diameter of the evaporator.

Supplementary methods

Method S1. Calculation of apparent energy utilization efficiency.

Evaporation can proceed even in the absence of direct solar irradiation when the surrounding air is not saturated, driven by a vapor concentration gradient between the evaporating surface and the ambient environment. This process, commonly referred to as dark evaporation or ambient-energy-assisted evaporation, enables vapor generation without solar input and reflects the ability of an evaporator to harvest environmental thermal energy through convective and radiative heat exchange.

To quantify dark evaporation behavior, MSE samples with different exposed heights were placed inside a light-tight enclosure maintained at an ambient temperature of 28 °C. No external illumination was applied during the measurements. Water mass loss was monitored gravimetrically using an analytical balance, and each experiment was repeated three times to ensure reproducibility, with average values reported. The results show that the dark evaporation rate increased monotonically with exposed height, consistent with the enlarged lateral evaporation area and enhanced interaction with ambient dry air. When the exposed height approached zero, the evaporation rate converged to that of a planar surface, representing the intrinsic dark evaporation of the evaporator material.

These dark evaporation results indicate that taller MSE architectures, particularly the FBCC-+ designs, are capable of harvesting ambient thermal energy through lateral evaporation surfaces. This ambient energy contribution supplements direct solar heating during illuminated operation and plays an important role in enhancing the overall evaporation flux.

The apparent energy utilization efficiency (η) of the solar evaporators under illumination was calculated using the conventional latent-heat-based definition widely adopted in solar evaporation studies[3-5]. To estimate the additional evaporation associated with solar irradiation under identical environmental conditions, the net evaporation rate was first determined by subtracting the dark evaporation component:

$$\dot{m}_{net} = \dot{m}_{light} - \dot{m}_{dark} \quad \text{(S1)}$$

The energy utilization efficiency was then calculated as:

$$\eta = \frac{\dot{m}_{net}(h_{lv}+C_p\Delta T)}{P_{solar}} \tag{S2}$$

where $\dot{m}_{net}$ is the net mass evaporation rate (kg m^{-2} s^{-1}), h_{lv} is the latent heat of vaporization of water (2.26 MJ kg^{-1}), C_p is the specific heat capacity of water (4.18 kJ kg^{-1} K^{-1}), ΔT and P_{solar} is the incident solar power density (1 kW m^{-2}).

Values of η exceeding unity reflect the combined contribution of direct solar energy and harvested ambient thermal energy to vapor generation, rather than a violation of energy conservation. In this context, the subtraction-based formulation serves as a baseline-normalized, comparative metric, and does not represent a strict physical separation of solar and environmental contributions, particularly in three-dimensional architectures where environmental energy exchange is significant.

Method S2. Numerical simulation

Numerical simulations were performed using COMSOL Multiphysics 5.5 to investigate the diffusion-driven redistribution of salt inside porous evaporators with spatially asymmetric evaporation rates. Only the Transport of Diluted Species (TDS) physics interface was employed. No thermal or hydrodynamic modules were activated, because all evaporation fluxes were taken directly from experimental measurements and the liquid inside the lattice remained fully saturated and isothermal during operation. The goal of the simulations was therefore to isolate the effect of evaporation-induced concentration gradients on the diffusion of NaCl.

S1.1 Model Geometry and Material Properties

Two numerical geometries corresponding to the experimental devices were constructed in two-dimensional cross-section: (i) a symmetric wall-type evaporator (Case 1), and (ii) a cup-shaped evaporator with inner fast-evaporating (BCC) and outer slow-evaporating (FCC-+) surfaces (Case 2).

The liquid domain was modeled as a homogenized porous region, reflecting the interconnected lattice architecture of the experimental samples. Because the experimental evaporators consist of periodically repeated lattice units, transport in the lateral direction is spatially repetitive. Therefore, the three-dimensional structure can be reasonably represented by a two-dimensional homogenized cross-sectional model capturing the dominant vertical concentration gradients. The pore pathways were relatively straight, therefore, the porosity and tortuosity were taken as $\varepsilon = 0.534$ and $\tau = 1$, respectively. The domain was assumed to be fully saturated with 20 wt% NaCl brine, with a density of $\rho = 1160$ kg m^{-3}. The bulk diffusion coefficient of NaCl ($D = 1.0 \times 10^{-9}$ m^2 s^{-1}) was adjusted for porosity and tortuosity to obtain an effective value.

$$D_{eff} = \frac{D \cdot \varepsilon}{\tau} \qquad \text{(S3)}$$

The initial concentration throughout the domain and the bottom supply boundary was set to $c_0 = 4.0 \times 10^3$ mol m^{-3}, corresponding to 20 wt% NaCl.

S1.2 Governing Equation

Salt transport within the saturated porous region was described solely by the transient diffusion equation:

$$\frac{\partial c}{\partial t} = \nabla \cdot (D_{eff} \nabla c) \quad \text{(S4)}$$

where c is the NaCl concentration (mol m^{-3}), t is time (s), and D_{eff} is the effective diffusion coefficient. No advective term appears because the evaporators operated under negligible flow conditions.

S1.3 Boundary Conditions

S1.3.1 Evaporative boundaries.

Local water evaporation leads to salt enrichment at the liquid–air interface. This enrichment was imposed as a boundary flux:

$$\mathrm{n} \cdot \left(-D_{eff} \nabla c\right) = \frac{m_{evap}}{\rho} c \quad \text{(S5)}$$

where n is the outward normal vector, m_{evap} is the experimentally measured local water-evaporation flux (kg m^{-2} s^{-1}), ρ is the brine density, and c is the local NaCl concentration at the boundary.

The following experimentally measured values were applied:

Hybrid wall:

Fast evaporation surface: $m_{\mathrm{evap}} = 6.0 \times 10^{-4}$ kg m^{-2} s^{-1} (ER of FCC-+, Figure 4B)

Slow evaporation surface: $m_{\mathrm{evap}} = 5.1 \times 10^{-4}$ kg m^{-2} s^{-1} (ER of BCC, Figure 4B)

Hybrid cup-shape evaporator:

Top and inner surface: $m_{\mathrm{evap}} = 9.25 \times 10^{-4}$ kg m^{-2} s^{-1} (Top and inner ER, Figure S27)

Outer surface: $m_{\mathrm{evap}} = 5.56 \times 10^{-5}$ kg m^{-2} s^{-1} (Total – Top and inner ER, Figure S27)

S1.3.2 Reservoir boundary.

The bottom boundary was assigned a fixed concentration: $c = c_0$ to represent continuous replenishment by the underlying brine reservoir.

S1.3.2 Non-evaporative walls.

Remaining boundaries were treated as impermeable:

$$n \cdot \left(-D_{eff} \nabla c\right) = 0 \tag{S6}$$

S1.4 Numerical Procedure

A time-dependent study was employed over a total duration of 1000 s with a time step of 10 s. Shorter simulations (< 500 s) did not permit the development of lateral concentration gradients, whereas a 1000 s horizon reliably produced a quasi-steady distribution. The mesh consisted of triangular finite elements with refined resolution near all evaporative boundaries to resolve steep diffusion layers. The default implicit backward-differentiation (BDF) solver was used with a relative tolerance of 10^{-3}.

S1.5 Output Quantities

The simulation produced spatial maps of NaCl concentration c(x, y, t) and total diffusive flux vectors $\mathbf{N} = -D_{eff} \nabla c$. Streamline visualizations of **N** were used to identify the direction of ion migration. The evolution of local supersaturation, salt accumulation near specific surfaces, and concentration gradients across the liquid domain were extracted directly from the computed fields.

Method S3. Preparation of artificial seawater.

Artificial seawater was prepared to simulate the ionic composition of natural seawater while ensuring experimental reproducibility and controlled salinity. Sodium chloride ($NaCl$, ≥99.0%, Samchun Chemicals), potassium chloride (KCl, ≥99.0%, Duksan Pure Chemicals), calcium chloride ($CaCl_2$, 96.0%, Samchun Chemicals), and magnesium sulfate ($MgSO_4$, 99.5%, Samchun Chemicals) were used as received without further purification. Specifically, 16.490 g of $NaCl$, 0.457 g of KCl, 0.699 g of $CaCl_2$, and 2.060 g of $MgSO_4$ were sequentially dissolved in 600 mL of distilled water under continuous magnetic stirring at room temperature until complete dissolution was achieved.

The salt composition was selected to approximate the major ionic constituents of seawater, with Na^+ and Cl^- as the dominant species, followed by Mg^{2+}, SO_4^{2-}, Ca^{2+}, and K^+, consistent with previously reported artificial seawater formulations used in solar desalination studies[7,22]. The resulting solution corresponds to a total salinity comparable to natural seawater and was used as the feed solution for outdoor solar desalination experiments. Prior to use, the solution was visually inspected to confirm the absence of undissolved solids and was equilibrated to ambient temperature to ensure consistent initial conditions across experiments.

Supplementary references

1 Gao, T. *et al.* More from less: improving solar steam generation by selectively removing a portion of evaporation surface. *Science bulletin* **67**, 1572-1580 (2022).

2 Li, X. *et al.* Less is more: reducing evaporation dead zones to tune thermal convection in donut-structure evaporators for high-efficiency interfacial solar steam generation. *Advanced Functional Materials* **35**, 2504502 (2025).

3 Liang, H. *et al.* Thermal efficiency of solar steam generation approaching 100% through capillary water transport. *Angewandte Chemie International Edition* **58**, 19041-19046 (2019).

4 Chen, D. *et al.* A Self-Floating Solar Evaporator Based on Carbon Black/Polydimethylsiloxane for Highly Efficient and Stable Desalination. *ACS Applied Materials & Interfaces* (2025).

5 Yan, L. *et al.* PC@ PPy porous membrane prepared by breath figure method with superior mechanical property for efficient solar interfacial evaporation. *Chemical Engineering Journal* **469**, 144059 (2023).

6 Liu, Y., Li, Q., Li, Y., Feng, H. & Deng, R. Directional salt crystallization system for high-efficiency solar-driven evaporation to achieve zero-liquid discharge. *Separation and Purification Technology* **354**, 129412 (2025).

7 Meng, F. *et al.* N-doped carbon@ Cu core–shell nanostructure with nearly full solar spectrum absorption and enhanced solar evaporation efficiency. *Journal of Materials Chemistry A* **10**, 9575-9581 (2022).

8 Ma, X., Jia, X., Yao, G. & Wen, D. Umbrella evaporator for continuous solar vapor generation and salt harvesting from seawater. *Cell Reports Physical Science* **3** (2022).

9 Zhang, C. *et al.* Designing a next generation solar crystallizer for real seawater brine treatment with zero liquid discharge. *Nature Communications* **12**, 998 (2021).

10 Wu, L. *et al.* Highly efficient three-dimensional solar evaporator for high salinity desalination by localized crystallization. *Nature Communications* **11**, 521 (2020).

11 Wang, C., Zhang, H., Kang, Z. & Fan, J. 3D cellular solar crystallizer for stable and ultra-efficient high-salinity wastewater treatment. *Advanced Science* **11**, 2305313 (2024).

12 Li, Y. *et al.* Pyramid-shaped solar evaporator with high-efficient interfacial evaporation and salt harvesting capability. *Journal of Cleaner Production* **434**, 139956 (2024).

13 Zhu, J. *et al.* FDM 3D-printed volcanic-shaped structure for ultrafast solar-driven interfacial evaporation and efficient energy utilization. *Desalination* **548**, 116275 (2023).

14 Li, X. *et al.* 3D micro-lattice structural hydrogel evaporator with super salt resistance for solar desalination of high-salinity. *Chemical Engineering Journal* **502**, 158019 (2024).

15 Li, L. *et al.* Long-term salt resistance in hypersaline, multi-ion brines via bionic solar evaporator based on thermal field optimization. *Applied Thermal Engineering*, 128057 (2025).

16 Zou, M. *et al.* 3D printing a biomimetic bridge-arch solar evaporator for eliminating salt

accumulation with desalination and agricultural applications. *Advanced Materials* **33**, 2102443 (2021).

17 Mao, W. *et al.* Highly Energy-Efficient 3D-Printed Solar Evaporator with Integrated Salt Self-Collection for Zero Liquid Discharge Desalination. *Environmental Science & Technology* (2025).

18 Zhang, X. *et al.* Bioinspired and 3D-printed solar evaporators for highly efficient freshwater-electricity co-generation. *Materials Horizons* (2025).

19 Liu, X., Liu, Q., Liu, Z. & Liu, G. 3D printing cup evaporator with open architecture for seawater desalination. *Chemical Engineering Journal* **505**, 159221 (2025).

20 Li, L. *et al.* Bioinspired Body-Centered Cubic Evaporators: Leveraging Cellular Fluidics for Ultra-Efficient Solar-Driven Water Evaporation via PμSL. *ACS Applied Materials & Interfaces* **17**, 45200-45212 (2025).

21 Wang, D. *et al.* Dyson sphere-like evaporators enhanced interfacial solar evaporation via self-generated internal convection. *Nature Communications* **16**, 7985 (2025).

22 Nie, B. *et al.* A porous dome array evaporator for high-performance photothermal water evaporation and thermoelectric power generation. *Journal of Materials Chemistry A* **12**, 293-302 (2024).
