# Supplementary material for: Multicellular architectures for high-rate solar evaporation with spatial salt crystallization under high-salinity
Source: Nat Commun. 2026 Jul 22;17:8946. doi: 10.1038/s41467-026-75913-w (PMC13503716; doi:10.1038/s41467-026-75913-w)
Supplement: Supplementary file 2 [file 41467_2026_75913_MOESM2_ESM.pdf]

Description of the Additional Supplementary Files

Supplementary Movie 1: Capillary-driven water transport within the lattice structures.

Supplementary Movie 2: Spatial localization of salt crystallization in hybrid-cell architectures.
