# Supplementary material for: Multicellular architectures for high-rate solar evaporation with spatial salt crystallization under high-salinity
Source: Nat Commun. 2026 Jul 22;17:8946. doi: 10.1038/s41467-026-75913-w (PMC13503716; doi:10.1038/s41467-026-75913-w)
Supplement: Supplementary file 3 [file 41467_2026_75913_MOESM5_ESM.pdf]

nature portfolio

Peer Review File

Multicellular Architectures for High-Rate Solar Evaporation with Spatial Salt Crystallization under High-Salinity

Corresponding Author: Professor Hyejeong Kim

This file contains all reviewer reports in order by version, followed by all author rebuttals in order by version.

Version 0:

Reviewer comments:

Reviewer #1

(Remarks to the Author)
In this study, various 3D-printed porous solar evaporators were designed and fabricated, and the effects of unit cell geometric structures on capillary water transport, solid-liquid heat transfer, and salt crystallization behavior were systematically investigated. The authors demonstrate the pivotal role of geometric structures in achieving high evaporation rates, zero liquid discharge (ZLD), and spatial salt crystallization. Nevertheless, several important issues need addressing prior to formal publication, with specific comments as follows:

1.The graphite coating is characterized merely by Fourier Transform Infrared Spectroscopy (FTIR) and contact angle measurements in the paper, with no characterizations conducted on its thickness, uniformity, crystalline structure and surface elemental composition. It is recommended to supplement the following characterization data:
① X-ray Diffraction (XRD) analysis to characterize the crystalline structure of graphite, thus verifying the material basis for its photothermal conversion;
② X-ray Photoelectron Spectroscopy (XPS) measurements to determine the elemental valence states on the coating surface, which, in combination with FTIR results, can further analyze the variations in hydrophilic functional groups after oxygen plasma treatment;
③ Scanning Electron Microscopy (SEM)/Transmission Electron Microscopy (TEM) observations to characterize the microtopography and thickness of the coating and quantify its uniformity.

2. How could the uniformity of this graphite coating be verified? Did the thickness of this graphite coating also affect the water transport and the effect of photothermal effect?

3. The manuscript mainly focused on the effects of different geometric shapes. But did the process of graphene coating also ensure the stability and uniformity of the coating for overly complex and delicate printing devices?

4.The paper identifies liquid-solid contact perimeter, porosity and thermal interface area as key parameters, yet the coupling effects among these three parameters are not analyzed. It is recommended to supplement the following content:
① Plot the relevant correlation curves to quantify the regulatory effect of liquid-solid contact perimeter on evaporation performance under different porosity conditions;
② Adopt the control variable method to investigate the contribution of the variation of a single parameter to evaporation performance, thus clarifying the core regulatory parameter.

5. In Figures 4B, 4C and S5, the evaporation efficiencies of various shapes were compared. But why was the FBCC sample selected for the subsequent experiments?

6. This study adopts a system consisting of acrylate-based resin and graphite coating. Is this geometric design strategy applicable to other photothermal materials (e.g., carbon nanotubes, MXene, polypyrrole) and substrate materials?

7. In Section 4.3 (Water transport experiments), the manuscript notes treatment of the structures with 1 wt% PVA to ensure hydrophilicity. Please provide detailed process parameters and address the following questions:
① Was the PVA treatment applied in the solar evaporation performance tests? If not, clearly justify why this treatment is

required for capillary transport tests but not for evaluation performance tests.
② Elucidate whether PVA treatment affects the photothermal conversion efficiency and surface hydrophilicity of the graphite coating. Supplement characterization data (optical absorption spectra, contact angle measurements) for PVA-treated samples to eliminate PVA-induced interference on evaporator performance, and thoroughly discuss the potential limitations and impacts of this treatment.

8. It is suggested to conduct simulation analysis on the impact of different geometric structures on water transport efficiency.

9. Did the excessive accumulation of salt on the BCC surface affect the photothermal efficacy?

10. Line 129: The paper attributes the absorption bands at 1236-1135 cm-1 to C-O and C-O-C stretching vibrations, yet they are labeled as O-H and C-O-C in Figure 2C; this inconsistency should be corrected.

11. Line 246: The unit of the evaporator's evaporation rate under dark conditions is annotated incorrectly. It should be revised from g m-2 h-1 to kg m-2 h-1 to maintain consistency with the unit notations in the context and Figure S5.

Reviewer #2

(Remarks to the Author)
This manuscript reports a systematic investigation of 3D-printed multicellular solar evaporators with tailored lattice unit cells, aiming to elucidate the geometry-transport-performance relationships for high-salinity solar evaporation and spatial salt crystallization. The research topic is highly relevant to the sustainable desalination field, and the proposed geometry-engineered design strategy for solar evaporators presents novel insights. The experimental design is relatively systematic, and the obtained results show promising potential for scalable solar desalination applications. However, the manuscript suffers from several critical limitations. Major revisions are required to address these issues and improve the scientific rigor and completeness of the work. Detailed comments are as follows:
1.The liquid-solid contact perimeter and porosity are identified as key geometric parameters governing evaporation rates, while the projected solid area of unit cells directly determines the light-harvesting area, a critical factor correlated with solar evaporation performance. Especially for the 2 mm × 2 mm × 2 mm unit cells used in this study, the influence of projected solid area cannot be neglected. The authors are requested to supplement the projected solid area data of all investigated unit cells (SC, BCC, FCC-X, FCC-+, Isotruss, FBCC-X, FBCC-+), and analyze the contribution of light-harvesting area to the evaporation performance of each unit cell to improve the comprehensiveness of the geometric parameter-evaporation rate correlation system.
2.The manuscript only compares the porosity, average contact perimeter, capillary rise height and evaporation performance of different unit cell types, revealing a higher correlation between water transport capacity and average contact perimeter. However, no tests on water transport capacity and evaporation performance of the same unit cell type with different dimensional scales are conducted. The authors are suggested to select a typical unit cell (e.g., FBCC-+), design 3-4 groups of gradient dimensions, test its capillary rise kinetics, maximum capillary rise height, evaporation rate and other indicators, analyze the regulation law of unit cell dimensions on water transport and evaporation performance, and discuss its influence on salt ion migration capacity.
3.The manuscript focuses on the effect of evaporator height on evaporation rate and emphasizes the contribution of lateral surface evaporation, but fails to provide the specific top surface dimensions (length, width/diameter) of all experimental evaporators, making it impossible to calculate the lateral/top surface area ratio and lack a basis for the quantitative analysis of lateral surface evaporation contribution. The authors are required to supplement the top surface dimensions of the evaporators, and quantitatively analyze the evaporation contribution ratio of lateral and top surfaces in combination with the lateral surface area at different heights.
4.The manuscript proposes that in the hybrid multicellular structure, salt ions preferentially crystallize in the BCC region with low evaporation rate, attributing this phenomenon to ion diffusion induced by evaporation rate gradients. However, Figure 3D clearly shows that the capillary rise capacity of the BCC unit cell is significantly weaker than that of FBCC-+, which is mainly due to the combined characteristics of porosity, pore size and contact perimeter of the BCC unit cell leading to poor water transport capacity and limited salt ion migration. Furthermore, under sufficient water supply, a lower evaporation rate should result in less salt accumulation rather than more. The direct correlation between salt ion accumulation and low evaporation rate lacks physical rationality.
5.Figure S9 shows that the evaporation efficiency is significantly higher than 100% when the evaporator height is 0 cm (without lateral evaporation contribution), and the manuscript does not provide a reasonable interpretation for this phenomenon.
6.Figure S15b shows that lateral surface evaporation has almost no contribution to evaporation rate, while Figure 4D clearly reveals that lateral evaporation surfaces at different heights have a significant positive contribution to evaporation rate. The contradictory results seriously affect the credibility of the core conclusion of lateral surface evaporation contribution. The authors are requested to supplement targeted verification experiments,

Reviewer #3

(Remarks to the Author)
The manuscript presents an interesting study on lattice-structured evaporators and their role in salt management and water purification. The work is generally well organized and the concept of geometry-enabled performance enhancement is appealing and interesting. However, several points would benefit from further clarification or discussion to strengthen the mechanistic interpretation and the practical implications of the design. The following comments are offered for the authors' consideration.

1. Although seven unit-cell architectures were analyzed in the capillary transport study, evaporation performance was evaluated for only four representative designs. Since the manuscript notes that FBCC structures share similar top-surface geometries with their FCC counterparts, it would be helpful to briefly verify whether the omitted architectures exhibit comparable evaporation behavior under the zero-height condition.
2. The evaporation rate increases from 2.16 to 6.90 kg $m^{-2} h^{-1}$ as the exposed height increases. Clarification of the extent to which this enhancement arises from an increase in effective evaporation area, as opposed to an intrinsic improvement in evaporation efficiency per unit active surface, would strengthen the interpretation of the results.
3. The experimental device consists of a three-dimensional ordered lattice, whereas the simulation is conducted using a two-dimensional homogenized cross-section. Additional justification of why this reduced model is sufficient to capture the experimentally observed salt localization behavior would be valuable.
4. The manuscript reports salt recovery efficiencies of approximately 96% and 97.49%. A brief discussion clarifying the origin of the unrecovered fraction would improve the completeness of the analysis. In particular, it would be helpful to indicate whether this portion remains trapped within the lattice structure, dissolved in residual brine, or is lost during the mechanical collection process.
5. The evaporator is regenerated through gentle scraping and rinsing. Further discussion of the long-term durability of the graphite coating and the structural integrity of the printed lattice under repeated operational cycles would strengthen the practical relevance of the work. In particular, it would be useful to consider whether repeated saline wetting/drying, salt crystallization, and mechanical salt removal might gradually affect coating adhesion, surface wettability, or the geometric fidelity of the lattice.
6. While the manuscript emphasizes unit-cell architecture and macro-scale geometry as scalable design parameters, the current implementation relies on high-resolution DLP printing combined with multiple post-processing steps. A brief discussion on how this design could be translated to larger-scale manufacturing would strengthen the practical perspective of the work.

Reviewer #4

(Remarks to the Author)
In this manuscript, the authors employ high-resolution, resin-printed materials to develop 3D evaporators with enhanced evaporation rates for zero-liquid-discharge applications. Specifically, they report that regular geometric patterns can be engineered to control wetting in interfacial experiments, thereby directing salt crystallization to regions away from the evaporating surface. While the material preparation and the 3D-printed configurations are exciting, the novelty in solar interfacial evaporation is unclear. I do not believe the work demonstrates the technical novelty and rigor expected for Nature Communications, and I would strongly suggest that the authors consider a more specialized journal. In addition, my assessment is based on the concerns discussed below:

1) In figure 5g, despite blockage of the material due to increasing salt crystallization, the evaporation continues to increase. How is this possible? As the authors claimed, the purpose of the photo-thermal material was to increase absorption. However, as figs. 4g and 5g suggest, the evaporation rate increases regardless of the photo-thermal surface that is exposed to the incoming sunlight. If white scattering salt surface can already enhance evaporation rates to such large values, then what is the necessity of surface modification?
2) Further, they argue that the lateral surfaces absorb environmental energy leading to the higher evaporation rates observed. However, as seen in the picture corresponding to 4-day mark in Fig. 5g, the lateral surfaces are completely covered with crystallized salts, attenuating environmental heat input via convection and radiation. These unexplainable trends in evaporation rates could be due to experimental artefacts which the authors should carefully check.
3) The authors claim that oxygen plasma treatment renders the surface hydrophilic. However, it is well-known that plasma-treated surfaces return to hydrophobic state due to organic contaminants from the ambient, effectively suppressing the effect of their surface treatment. Since the authors claim that this is essential, I wonder how this fits into their narrative of a sustained interfacial evaporation.
4) In method 1, the authors subtract the dark condition's evaporation rate from the solar evaporation. This is largely erroneous and displays a lack of understanding of mass transfer. The convection-driven evaporation process is driven by a difference in water vapor concentration at the evaporating surface and the ambient.
$m\_evap''=g\_m*C\_g*[c_{v,s}(T\_s)- RH*c_{v,s}(T\_\infty)]$
Where m_evap^" is the evaporation flux, C_g is the molar density of dry air, $c_{v,s}(T\_s)$ is the saturated vapor mole fraction at the surface temperature, RH is the ambient relative humidity, $c_{v,s}(T\_\infty)$ is the saturated vapor mole fraction at the ambient temperature, and g_m is the mass transfer coefficient.
One can see that the evaporation rate is determined by the surface and ambient temperatures, and the relative humidity of the ambient. Subtracting the obtained value from the dark condition is erroneous because the dark/natural convection experiment is a separate, independent physical process. The buoyancy flow is downwards as the surface temperature is colder than the ambient. The value obtained for that has nothing to do with the value obtained for the solar evaporation.
5) The authors did not mention the dimensions of the sample used for the indoor and outdoor solar evaporation tests. Also, the area used in all the calculations should be specified. Wetted area or top surface area?
6) In Figure S9, the authors report an apparent energy utilization of >200% for the 2 cm geometry and above. This is an improbably high value. Considering that the heat transfer coefficient (radiation + natural convection) is ≈10〖 " W/m" 〗 ^2 "K" , and ΔT≈1^∘ "C" , achieving a 100% power enhancement from the environment would require an area roughly 100 times larger than the top, incident area. This claim raises serious concerns about how the ambient energy absorption and evaporation rates were calculated.
7) Comparing the modified geometries with a baseline geometry is a good strategy to understand the effects of internal structure on the evaporation rate. However, a question remains: is this observed effect universally valid for all hydrophilic

materials, or is it heavily dependent on the specific surface chemistry of the material used in this study? The authors should comment on this limitation.
8) For the evaporation rate with tall structures shown in Figure 4, there might be issues with diffuse light reflections from the polystyrene foam to the tall structure, this should be considered in energy conservation. This might be reason for the enhanced efficiency rather than just from the environmental energy absorption.
9) The authors should comment on the ultimate limits of this spatial localization under a continuous feed scenario. Specifically, if the liquid reservoir is continuously replenished, at what point does the salt volume on the BCC side become so massive that it physically bridges over to the FBCC side or structural degradation occurs? Clarifying the mechanical limits of salt accumulation before a cleaning/washing cycle is triggered would better address the long-term, continuous practicality of this hybrid design

Reviewer #5

(Remarks to the Author)
I co-reviewed this manuscript with one of the reviewers who provided the listed reports. This is part of the Nature Communications initiative to facilitate training in peer review and to provide appropriate recognition for Early Career Researchers who co-review manuscripts.

Version 1:

Reviewer comments:

Reviewer #1

(Remarks to the Author)
I have carefully reviewed the revised manuscript and the authors' point-by-point response to the initial round of reviews. The authors have provided substantial new experimental data and analysis that significantly strengthen the study's conclusions and overall impact. Nevertheless, some issues are still needed to be considered before it can be published on Nature Communications.
1. The manuscript enhanced capillary force and water transport capacity through its printed structure. However, excessive water transport might lead to the excessive water required at the evaporation interface, which in turn affected the evaporation efficiency. Therefore, achieving a proper balance in this aspect was of utmost importance. It is recommended to solve this problem through experiments or simulations.
2. As shown in Figure 4D, the printed device significantly improved the evaporation efficiency by increasing the height. But what would happen if the height exceeded 5 cm?
3. Because the height of the evaporation device also had a significant impact on the evaporation efficiency, therefore, when comparing with the efficiency of other tasks, it is recommended to include the height parameter of the devices.

Reviewer #2

(Remarks to the Author)
The author have addressed what I am concerned about. Agree to accept.

Reviewer #3

(Remarks to the Author)
The authors have made good revisions to address the issues raised, and I believe this version of the paper is worth publishing.

Reviewer #4

(Remarks to the Author)
The authors have sufficiently addressed my comments and the work is technically accurate. They have also made modifications in response to all the reviewers' comments and the manuscript reads well.

Version 2:

Reviewer comments:

Reviewer #1

(Remarks to the Author)
The authors have made relevant revision and it can be considered for publication on Nature Communications.

Open Access This Peer Review File is licensed under a Creative Commons Attribution 4.0 International License, which permits use, sharing, adaptation, distribution and reproduction in any medium or format, as long as you give appropriate credit to the original author(s) and the source, provide a link to the Creative Commons license, and indicate if changes were made.
In cases where reviewers are anonymous, credit should be given to 'Anonymous Referee' and the source.
The images or other third party material in this Peer Review File are included in the article's Creative Commons license, unless indicated otherwise in a credit line to the material. If material is not included in the article's Creative Commons license and your intended use is not permitted by statutory regulation or exceeds the permitted use, you will need to obtain permission directly from the copyright holder.
To view a copy of this license, visit https://creativecommons.org/licenses/by/4.0/

Response to the reviewer's comments

We sincerely appreciate the reviewer's effort for careful and valuable comments on this manuscript. The insightful feedback you provided has significantly contributed to improve our manuscript. We have thoroughly reviewed and incorporated your comments into our work. Each of your points has been addressed, and the corresponding parts were highlighted in the revised manuscript.

Reviewer #1: In this study, various 3D-printed porous solar evaporators were designed and fabricated, and the effects of unit cell geometric structures on capillary water transport, solid-liquid heat transfer, and salt crystallization behavior were systematically investigated. The authors demonstrate the pivotal role of geometric structures in achieving high evaporation rates, zero liquid discharge (ZLD), and spatial salt crystallization. Nevertheless, several important issues need addressing prior to formal publication, with specific comments as follows:

1. The graphite coating is characterized merely by Fourier Transform Infrared Spectroscopy (FTIR) and contact angle measurements in the paper, with no characterizations conducted on its thickness, uniformity, crystalline structure and surface elemental composition. It is recommended to supplement the following characterization data:

a. X-ray Diffraction (XRD) analysis to characterize the crystalline structure of graphite, thus verifying the material basis for its photothermal conversion;

b. X-ray Photoelectron Spectroscopy (XPS) measurements to determine the elemental valence states on the coating surface, which, in combination with FTIR results, can further analyze the variations in hydrophilic functional groups after oxygen plasma treatment;

c. Scanning Electron Microscopy (SEM) / Transmission Electron Microscopy (TEM) observations to characterize the microtopography and thickness of the coating and quantify its uniformity.

Reply: We sincerely thank the reviewer for this constructive comment and for suggesting additional characterization techniques (XRD, XPS, and SEM/TEM) to further elucidate the properties of the graphite coating.

In this study, the graphite coating and subsequent oxygen plasma treatment were applied identically to all samples to ensure consistent surface chemistry, wettability, and photothermal characteristics. As such, the coating serves as a controlled background

condition, while the primary objective of this work is to isolate and evaluate geometry-dependent transport and evaporation behavior.

With regard to the analysis on the crystalline structure, XRD analysis has now been conducted and included in the revised manuscript. The XRD patterns exhibit a characteristic diffraction peak at ~26.5°, corresponding to the (002) plane of graphitic carbon and an amorphous halo at ~19° associated with the polymer matrix is observed[1]. The coexistence of these features confirms the successful incorporation and retention of crystalline graphitic domains within the composite structure. While XRD does not directly quantify photothermal conversion, it provides clear structural evidence for the presence of graphitic carbon, which is widely recognized as an efficient broadband photothermal material.

Importantly, the photothermal performance of the coating were directly validated by UV–vis–NIR absorption spectra analysis and thermal measurements as mentioned in the manuscript (Figure 2D). The graphite-coated samples exhibit strong broadband absorption (250–2500 nm) and a rapid temperature rise from 25.0 °C to 53.1 °C under 1 sun within 10 minutes, whereas the uncoated resin shows negligible heating (Figure 2F). Together with XRD (structural confirmation), these results provide complementary and sufficient evidence of the coating's photothermal functionality.

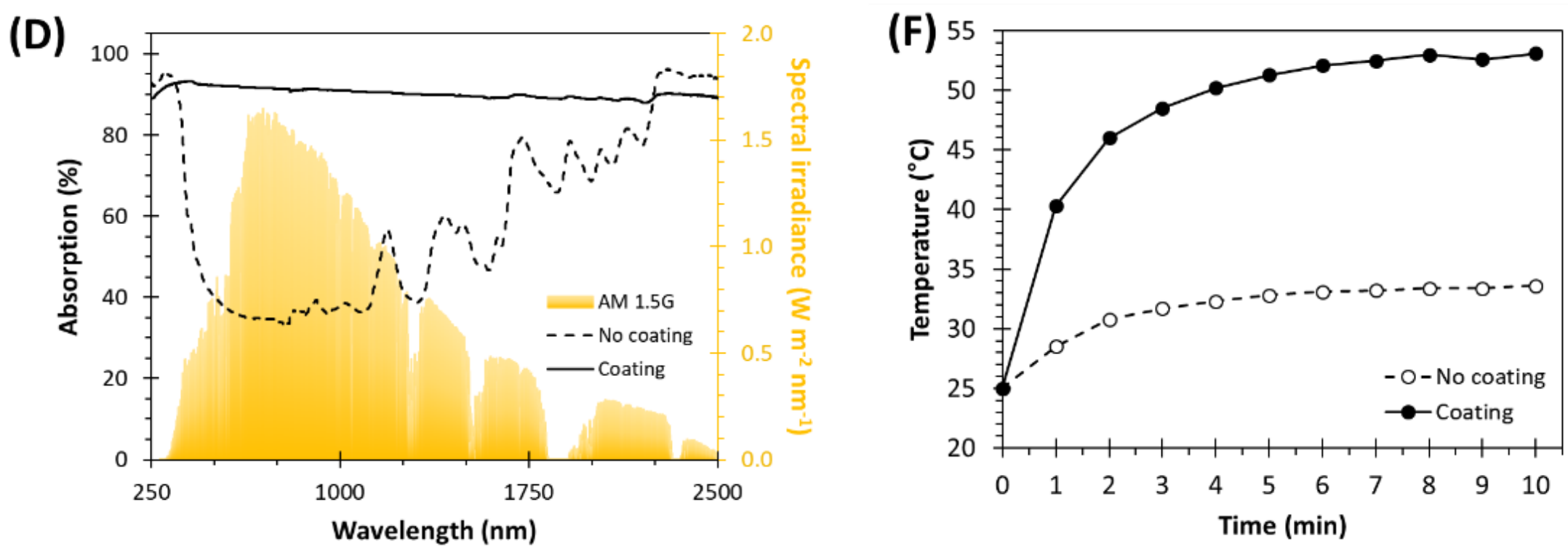

Figure 2. (D) UV–vis–NIR absorption spectra of the multicellular structures. (F) Temperature evolution of uncoated and coated multicellular structures under 1 sun irradiation.

Regarding XPS analysis, we fully agree that XPS is a powerful tool for probing surface elemental composition and chemical states. However, in the present study, the role of oxygen plasma treatment is to induce surface hydrophilicity rather than to systematically investigate surface chemistry as an independent variable. The effectiveness of this modification has been consistently verified through complementary experimental approaches. Static contact angle measurements demonstrate a clear transition from hydrophobic (70.6 ± 7.4°) to hydrophilic (44.7 ± 8.8°) behavior after treatment and post-drying. In addition, Fourier-

Transform Infrared Spectroscopy analysis shows the broadening of the O–H stretching band (~3300 cm^{-1}) and enhanced C–O/C–O–C features (1236–1135 cm^{-1}), indicating the formation of oxygen-containing functional groups. These results are consistent with partial oxidation of the graphite surface and the establishment of a hydrophilic interface.

Regarding the coating morphology and thickness, scanning electron microscopy (SEM) characterization has been performed and is included in the revised manuscript. The SEM images reveal a conformal and uniform graphite layer on the 3D-printed framework. Quantitative analysis using ImageJ software indicates that the average frame thickness increases from 400 ± 2 μm (pristine) to 413 ± 1 μm after coating. Based on this change, the graphite coating thickness is estimated to be approximately ~6.5 μm. These results confirm the uniformity and controlled thickness of the coating.

We believe that these additional analyses and clarifications enhances the completeness and contributes to a more comprehensive understanding of the physicochemical characteristics of the pristine and graphite coated 3D-printed multicellular structures.

On the revised manuscript; results

<From>

[Line 121-122] with no observable particle detachment or coating degradation (Figure S1).

<To>

[Line 123-130] with no observable particle detachment or coating degradation (Figure S1). The X-ray diffraction (XRD) pattern of the coated MSE displays a (002) reflection of graphitic carbon at ~26.5° alongside an amorphous halo at ~19° from the polymer resin matrix, confirming successful incorporation of graphitic domains (Figure S2)[1]. Coating uniformity was further assessed by scanning electron microscopy (Figure S3), revealing a conformal and homogeneous graphite layer on the 3D-printed framework. The average frame thickness increased from 400 ± 2 μm (pristine) to 413 ± 1 μm after coating, corresponding to an approximate graphite layer thickness of ~6.5 μm.

On the revised manuscript; methods

<Addition; text>

[Line 610-612] Microstructural features were characterized by scanning electron microscopy (SEM; Quanta FEG 250, FEI). Crystallographic features of the surface were analyzed by X-ray diffraction (XRD; SmartLab, Rigaku).

On the revised supplementary materials

<Addition; Figure S2>

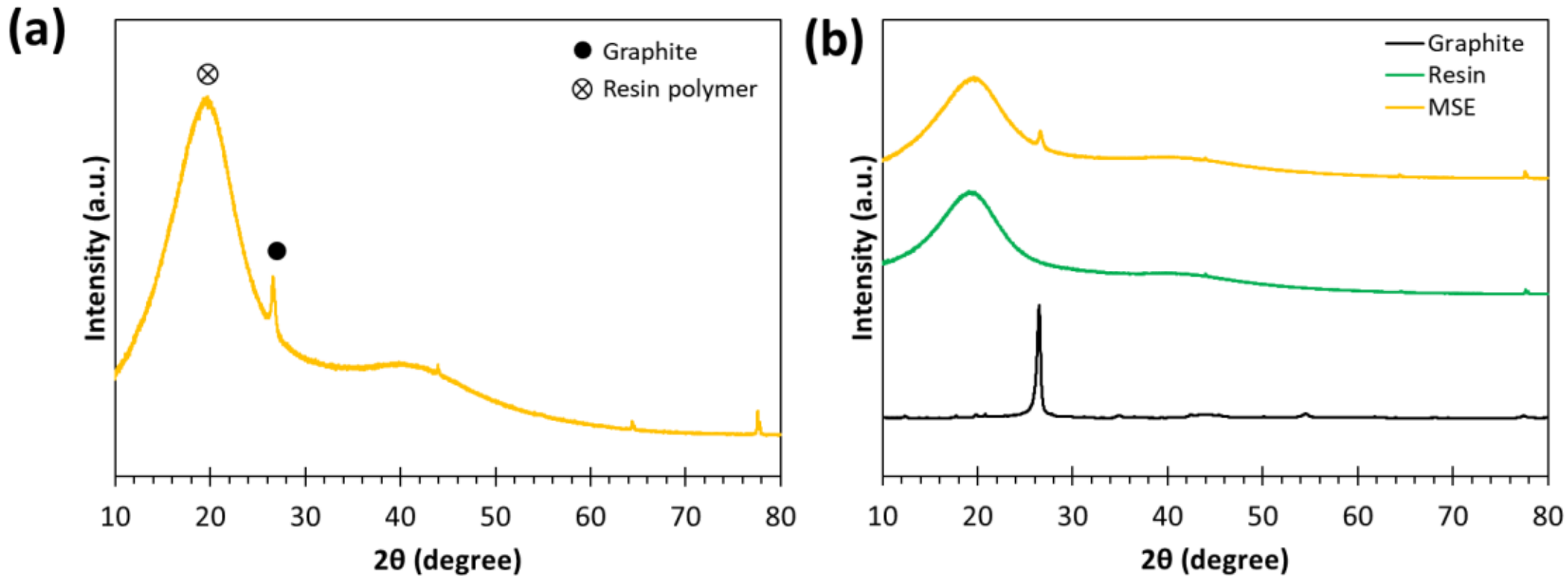

Figure S2. X-ray diffraction (XRD) patterns of the samples. (a) MSE after graphite coating and oxygen plasma treatment; (b) comparison of XRD patterns of commercial graphite, bare resin, and MSE.

<Addition; Figure S3>

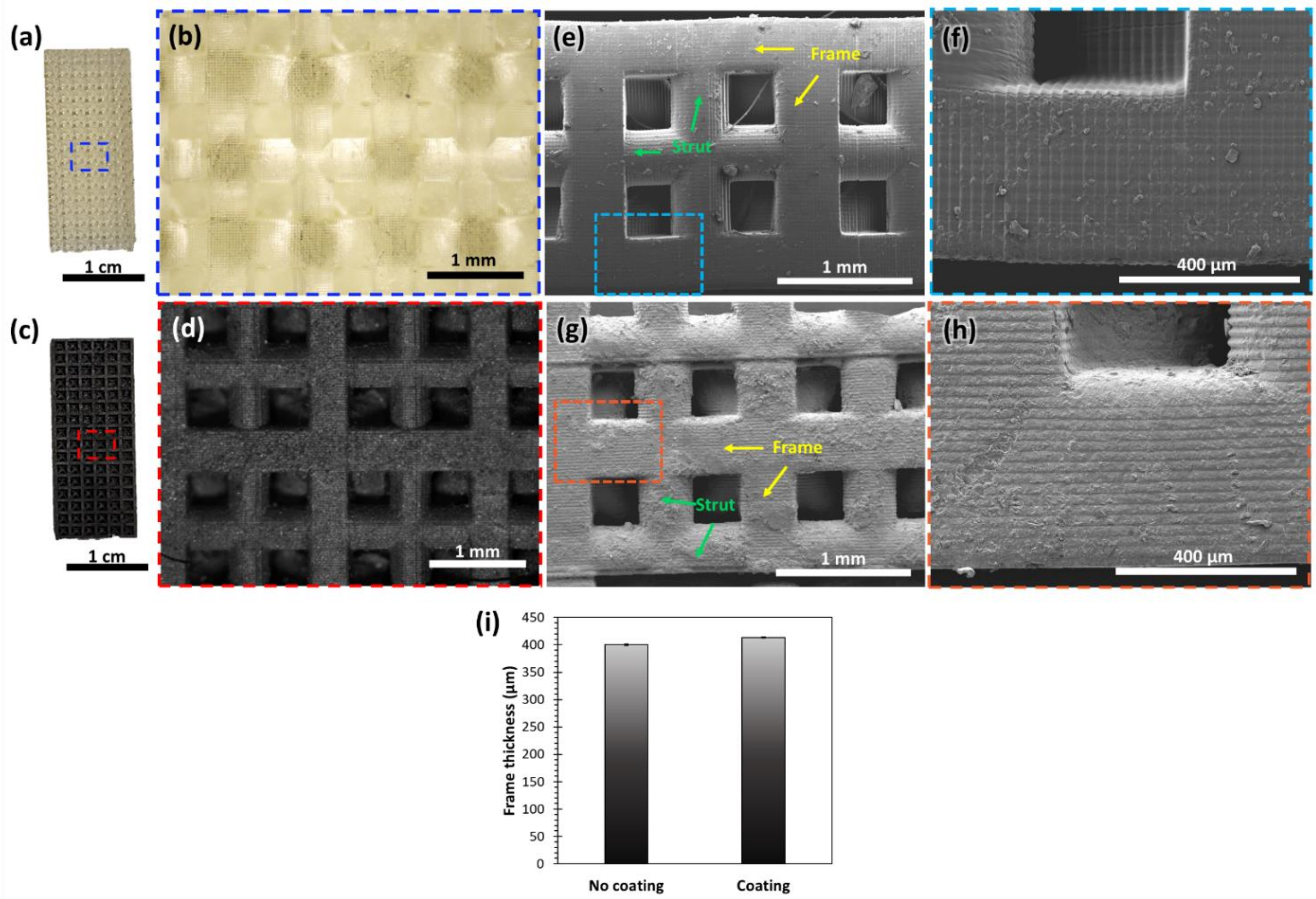

Figure S3. Optical and scanning electron microscopy (SEM) images of the multicellular structures before and after graphite coating. Optical images with corresponding higher-magnification views of (a, b) structure before coating and (c, d) after coating. SEM images of the structure (e) before coating and (g) after coating, with arrows indicating the frame and strut features. (f, h) Higher-magnification of the frame before and after coating, respectively, confirming homogeneous surface coverage. (i) Quantitative comparison of frame thickness before and after coating, analyzed using ImageJ software.

2. How could the uniformity of this graphite coating be verified? Did the thickness of this graphite coating also affect the water transport and the effect of photothermal effect?

Reply: We thank the reviewer for this insightful question regarding the uniformity of the graphite coating and its potential influence on water transport and photothermal performance.

The uniformity of the graphite coating has been verified through scanning electron microscopy (SEM) analysis, and included in the revised manuscript and supplementary information (Figure S3). The SEM images show that the graphite forms a conformal and continuous layer over the entire 3D-printed framework, uniformly covering both the frames and struts without observable aggregation or localized defects. This confirms that the coating process yields a homogeneous surface modification across the complex lattice geometry.

To further quantify the coating thickness and assess its uniformity, cross-sectional analysis was conducted using SEM images in combination with ImageJ measurements. The average frame thickness increases from 400 ± 2 μm (pristine) to 413 ± 1 μm after coating. Based on this change, the graphite layer thickness is estimated to be approximately ~6.5 μm, indicating a thin and well-controlled coating that is consistently applied across the structure.

With regard on the effect of coating thickness on water transport, the graphite layer is sufficiently thin relative to the characteristics dimensions of the unit cell ($2 \times 2 \times 2$ mm^3, with frame thickness and strut diameter of 400 μm, which forms a millimeter-scale pore size). Therefore, it does not significantly alter the intrinsic pore geometry or capillary pathways that govern liquid transport. Instead, the primary role of the coating is to modify surface wettability and provide a hydrophilic interface after plasma treatment, which facilitates continuous capillary-driven water supply. As the coating thickness is uniform across all samples, it does not introduce variability in transport behavior between different geometries.

Similarly, for photothermal performance, the graphite layer serves as a broadband light absorber. Within the thickness range used in this study, the coating is sufficient to achieve strong optical absorption and efficient heat generation, as confirmed by the UV–Vis–NIR and thermal measurements presented in the manuscript (Figure 2D and F). Because the coating is applied identically across all samples, its thickness does not act as a variable influencing comparative performance; instead, it provides a consistent photothermal background that enables fair evaluation of geometry-dependent effects.

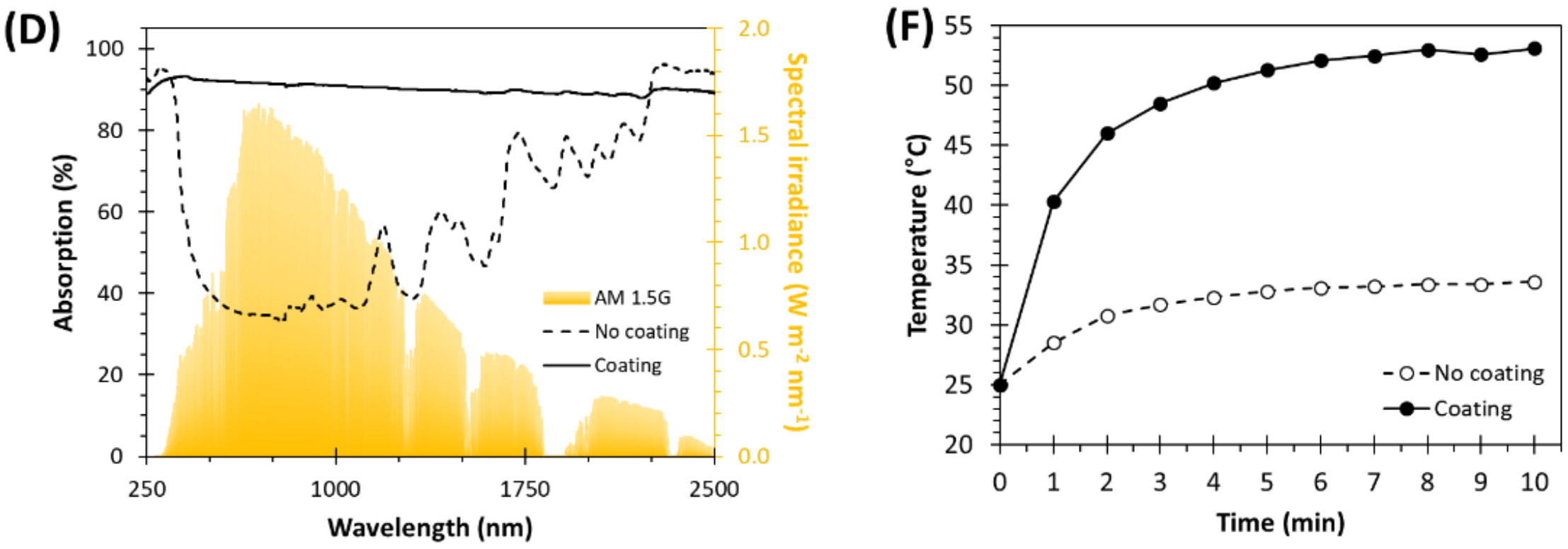

Figure 2. (D) UV–vis–NIR absorption spectra of the multicellular structures. (F) Temperature evolution of uncoated and coated multicellular structures under 1 sun irradiation.

In summary, SEM analysis confirms that the graphite coating is uniform and conformal, with a controlled thickness of approximately ~6.5 μm. This thin and homogeneous layer ensures consistent surface properties while minimally affecting the intrinsic transport pathways, thereby allowing the observed performance differences to be attributed primarily to structural geometry. These clarifications and the corresponding discussion have been incorporated into the revised manuscript and supplementary materials.

On the revised manuscript; results

<Addition>

[Line 127-130] Coating uniformity was further assessed by scanning electron microscopy (Figure S3), revealing a conformal and homogeneous graphite layer on the 3D-printed framework. The average frame thickness increased from 400 ± 2 μm (pristine) to 413 ± 1 μm after coating, corresponding to an approximate graphite layer thickness of ~6.5 μm.

On the revised manuscript; methods

<Addition; text>

[Line 610-612] Microstructural features were characterized by scanning electron microscopy (SEM; Quanta FEG 250, FEI). Crystallographic features of the surface were analyzed by X-ray diffraction (XRD; SmartLab, Rigaku).

On the revised supplementary materials

<Addition; Figure S3>

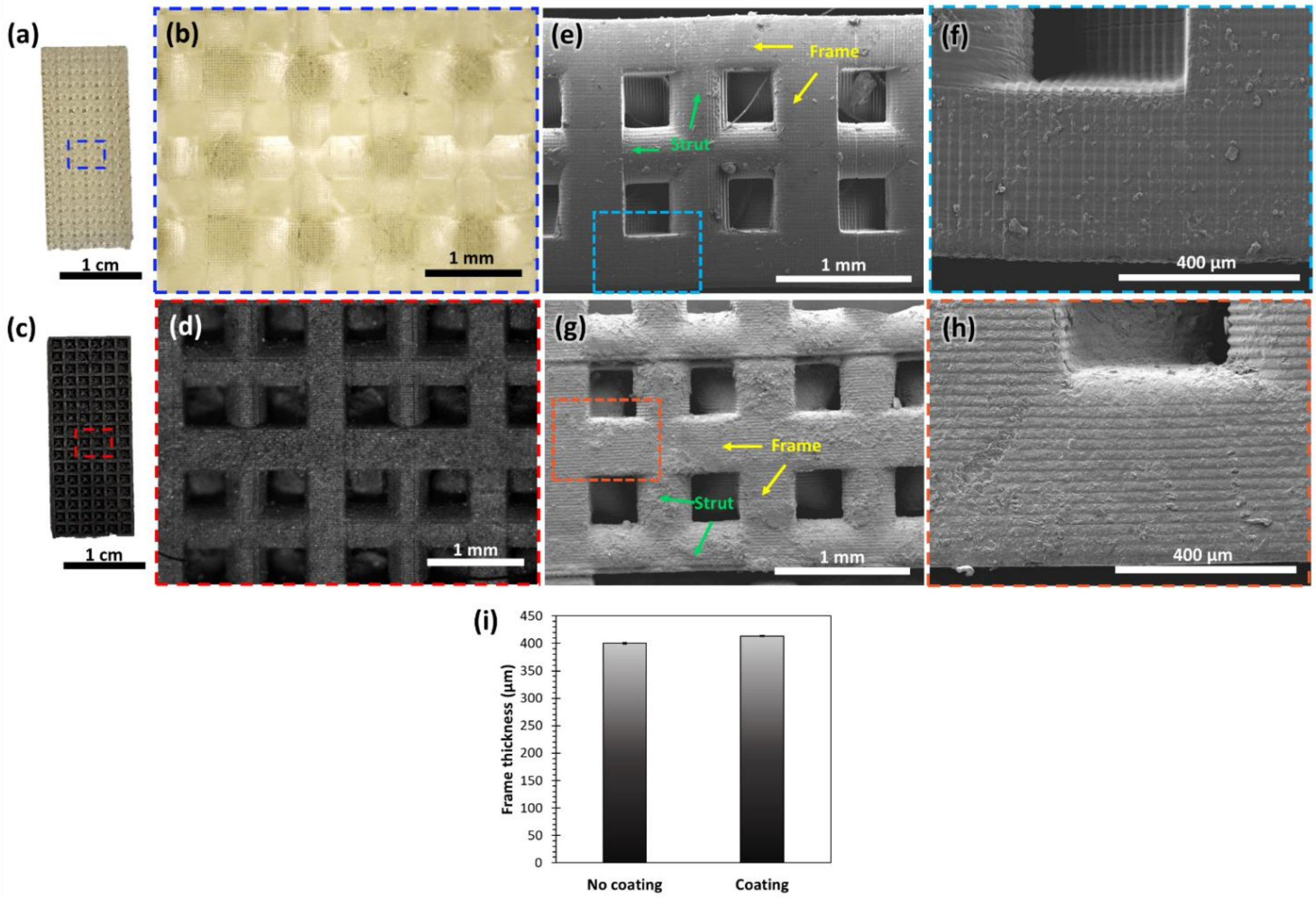

Figure S3. Optical and scanning electron microscopy (SEM) images of the multicellular structures before and after graphite coating. Optical images with corresponding higher-magnification views of (a, b) structure before coating and (c, d) after coating. SEM images of the structure (e) before coating and (g) after coating, with arrows indicating the frame and strut features. (f, h) Higher-magnification of the frame before and after coating, respectively, confirming homogeneous surface coverage. (i) Quantitative comparison of frame thickness before and after coating, analyzed using ImageJ software.

3. The manuscript mainly focused on the effects of different geometric shapes. But did the process of graphene coating also ensure the stability and uniformity of the coating for overly complex and delicate printing devices?

Reply: Thank you for this important question regarding the applicability of the coating process to complex and delicate geometries.

The graphite coating process employed in this study is based on solution-assisted dip coating, which is well-suited for conformal modification of complex three-dimensional structures[2,3]. Because the coating solution infiltrates the interconnected lattice network, both external surfaces and internal features such as thin struts and pores can be uniformly coated without relying on line-of-sight deposition. This characteristic enables effective surface modification even for geometrically complex and delicate 3D-printed architectures.

The uniformity of the coating across these structures was verified by SEM analysis (Figure S3). The images show continuous and homogeneous graphite coverage on both external and internal lattice surfaces, without observable aggregation, discontinuities, or structural damage. Importantly, no deformation or blockage of fine structural features was observed after coating, indicating that the process preserves the integrity of delicate geometries.

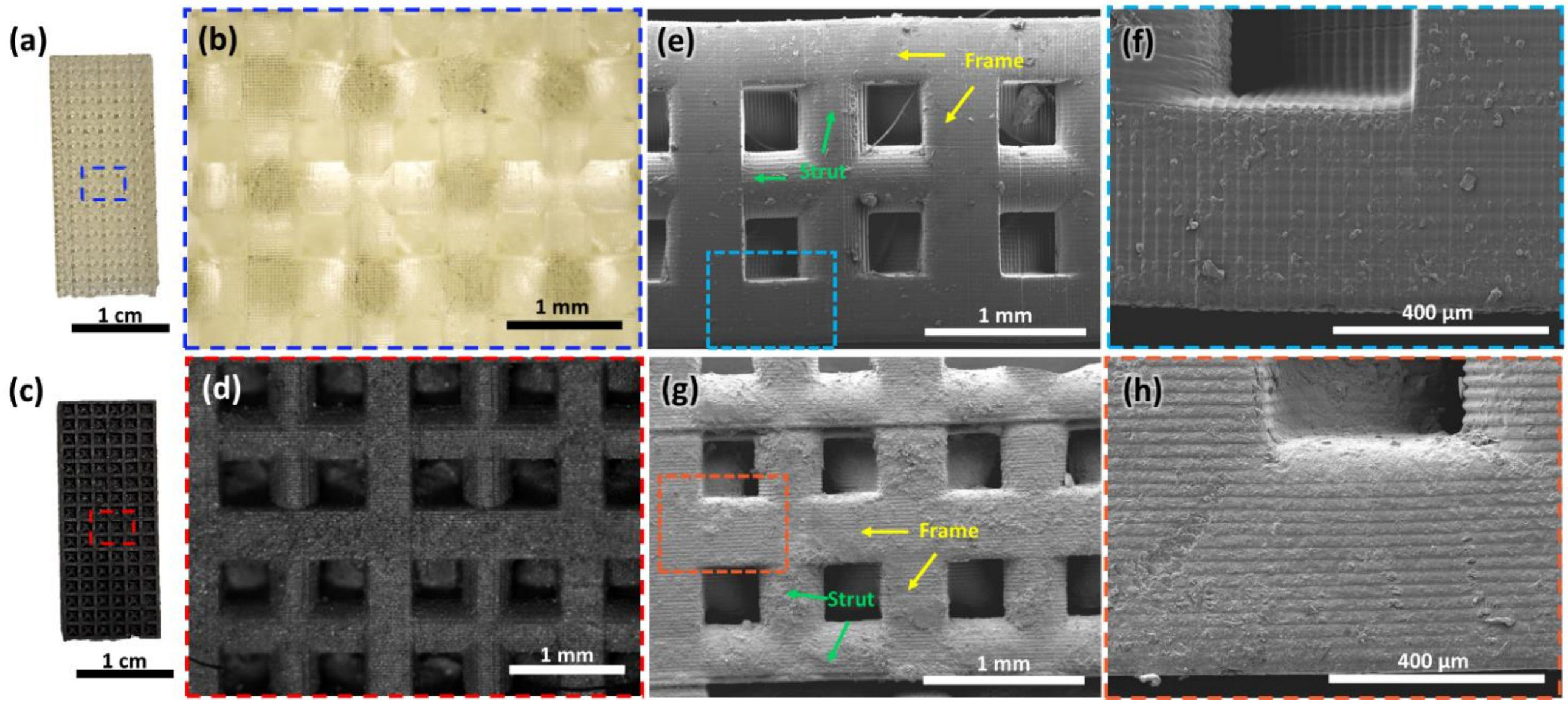

Figure S3. Optical and scanning electron microscopy (SEM) images of the multicellular structures before and after graphite coating. Optical images with corresponding higher-magnification views of (a, b) structure before coating and (c, d) after coating. SEM images of the structure (e) before coating and (g) after coating, with arrows indicating the frame and strut features. (f, h) Higher-magnification of the frame before and after coating, respectively, confirming homogeneous surface coverage.

In addition, the coating process was applied identically to all unit cell geometries, and consistent wettability and photothermal responses were obtained across samples, further supporting the uniformity and reproducibility of the coating. The subsequent oxygen plasma treatment and rinsing steps also did not compromise coating stability, as confirmed by stable hydrophilic behavior and sustained evaporation performance.

These clarifications and the corresponding SEM evidence have been incorporated into the revised manuscript and supplementary materials.

On the revised manuscript; results

<From>

[Line 118-119] … subsequently functionalized with a conformal graphite coating through a dip-coating process to impart photothermal activity.

<To>

[Line 118-121] …subsequently functionalized with a conformal graphite coating through a dip-coating process to impart photothermal activity. This method enables uniform surface coverage of complex three-dimensional lattice geometries, including internal pores and thin struts, without requiring line-of-sight deposition or inducing structural blockage[2,3].

On the revised supplementary materials

<Addition; Figure S3>

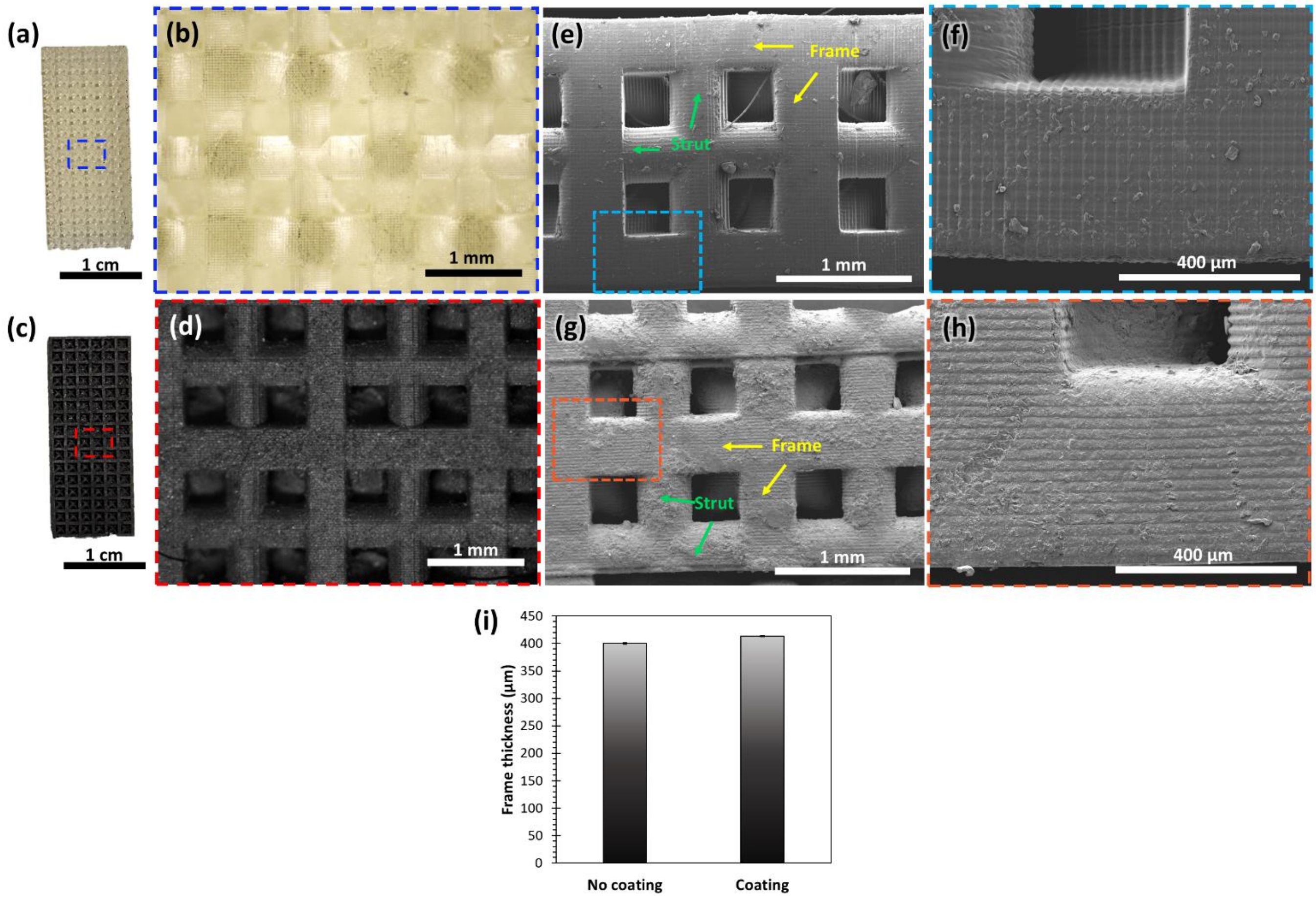

Figure S3. Optical and scanning electron microscopy (SEM) images of the multicellular structures before and after graphite coating. Optical images with corresponding higher-magnification views of (a, b) structure before coating and (c, d) after coating. SEM images of the structure (e) before coating and (g) after coating, with arrows indicating the frame and strut features. (f, h) Higher-magnification of the frame before and after coating, respectively, confirming homogeneous surface coverage. (i) Quantitative comparison of frame thickness before and after coating, analyzed using ImageJ software.

4. The paper identifies liquid-solid contact perimeter, porosity and thermal interface area as key parameters, yet the coupling effects among these three parameters are not analyzed. It is recommended to supplement the following content:

a. Plot the relevant correlation curves to quantify the regulatory effect of liquid-solid contact perimeter on evaporation performance under different porosity conditions;

b. Adopt the control variable method to investigate the contribution of the variation of a single parameter to evaporation performance, thus clarifying the core regulatory parameter.

Reply: Thank you for the insightful suggestion regarding the coupling effects among liquid–solid contact perimeter, porosity, and effective thermal transport area. To address this comment, we performed additional parametric experiments and expanded the analysis to quantitatively evaluate their individual and coupled contributions.

Correlation analyses between liquid–solid contact perimeter, porosity, and evaporation performance were added based on the comparison across different unit-cell geometries. As shown in Figure 3E and the revised Table S1, structures with larger wetting perimeter and lower porosity exhibit faster capillary rise and improved water supply. These parameters jointly determine the balance between adhesive capillary force and gravitational resistance, thereby governing liquid transport.

Table S1. Geometric characteristics and capillary rise of different unit cell architectures

Unit cell	Dimensions* ($W \times L \times H$, mm)	Porosity**	Avg. contact perimeter*** (mm)	Capillary rise height**** (mm)
SC	2 × 2 × 2	0.65	3.85	1.39
BCC	2 × 2 × 2	0.54	7.43	3.57
FCC-X	2 × 2 × 2	0.39	8.22	5.91
FCC-+	2 × 2 × 2	0.45	8.11	5.04
Isotruss	2 × 2 × 2	0.47	8.52	6.70
FBCC-X	2 × 2 × 2	0.30	10.10	16.26
FBCC-+	2 × 2 × 2	0.34	11.75	21.31

*Single unit cell

**$\text{Porosity} = 1 - \frac{Solid\ \ Volume}{Total\ \ volume}$

***Average liquid–solid contact perimeter of single unit cell (normal to capillary direction)

****After 15 minutes

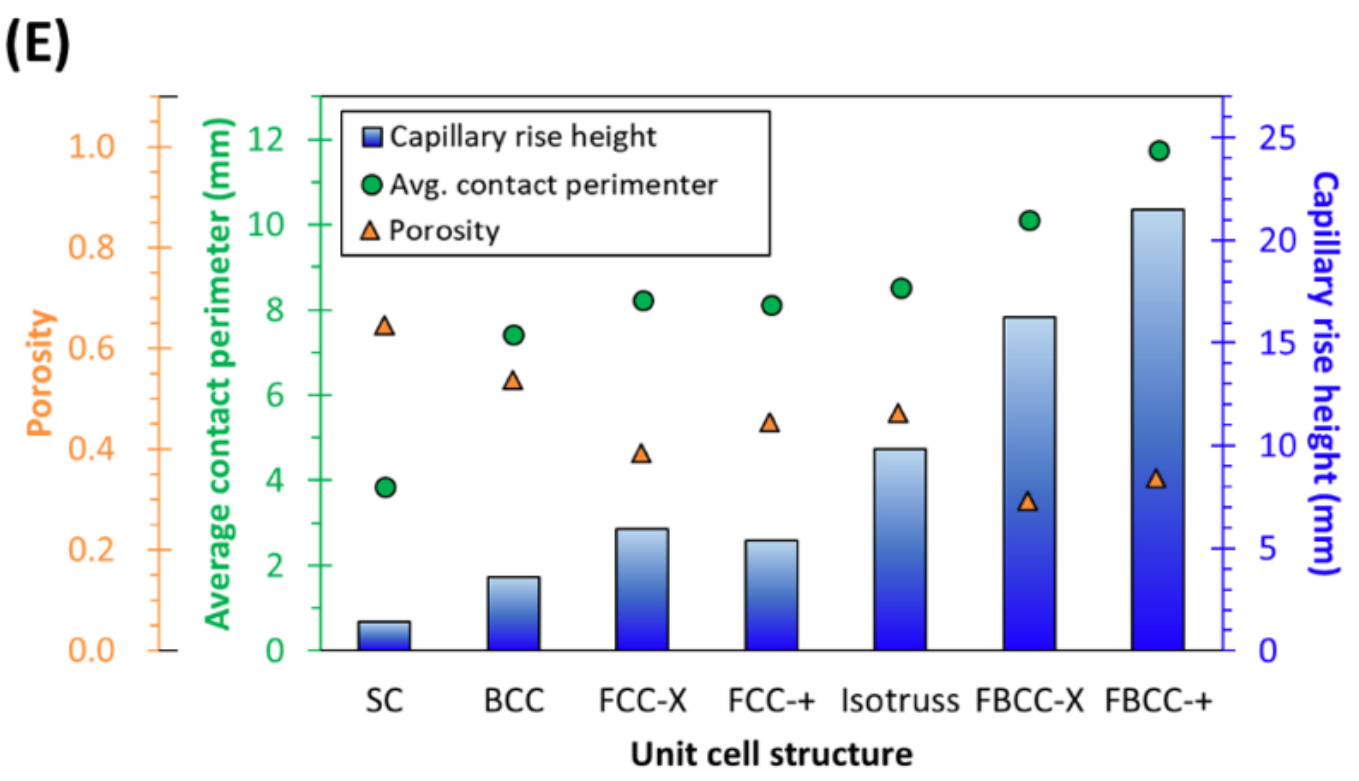

Figure 3. (E) Comparative analysis of porosity, average contact perimeter, and maximum capillary rise across the different unit cell geometries.

In addition, the effective thermal transport area, defined as

$$A_{eff} \approx P_{pore} \cdot H$$

where P_{pore} is the pore perimeter, H is the frame thickness, was calculated and compared with the evaporation rate (Figure S14 and Table S5). A strong correlation was observed, confirming that geometry-dependent solid–liquid contact governs heat delivery to the evaporation interface and directly influences evaporation performance.

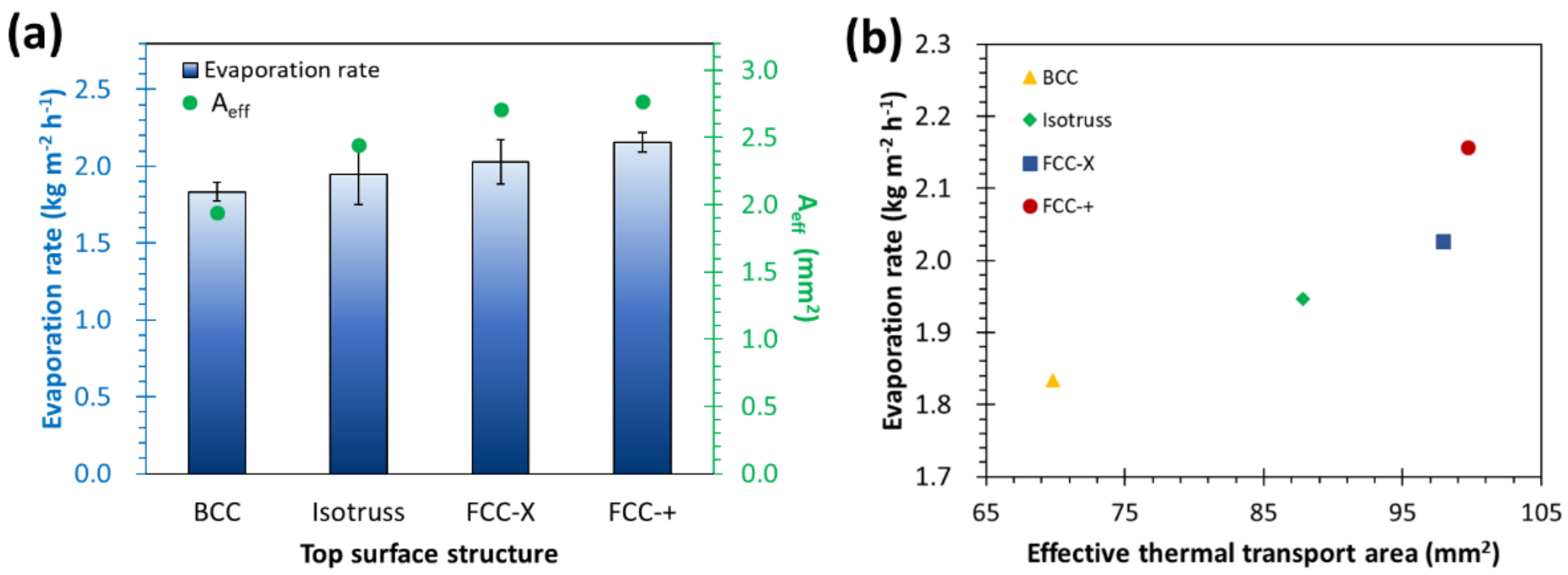

Figure S13. Correlation between evaporation rate and effective thermal transfer area. (a) Comparison of evaporation rates for different top-surface unit-cell architectures together with the effective thermal transport area (A_{eff}) of a single unit cell. (b) Relationship between evaporation rate and the total effective thermal transport area of the corresponding structures.

Furthermore, to isolate the contribution of individual parameters, additional control-variable experiments were conducted using the FBCC-+ topology, which was selected because it exhibits both the highest capillary transport and evaporation performance. The frame thickness (F) and strut diameter (D) were varied simultaneously while maintaining a constant unit-cell size, allowing systematic tuning of liquid–solid contact perimeter, porosity, and effective thermal transport area within a single geometry. The results (Figure S8, Table S2, Figure S16, and Table S6) show that the FBCC-+ structure with $F = D = 400$ μm exhibits the largest contact perimeter, lowest porosity, and highest effective thermal transport area, leading to the fastest capillary rise and highest evaporation rate (2.16 ± 0.06 kg m^{-2} h^{-1}). Conversely, reducing F and D to 200 μm decreases the effective contact region and increases porosity, resulting in both reduced capillary-driven water transport and evaporation performance (1.93 ± 0.16 kg m^{-2} h^{-1}). These parametric experiments isolate the geometric influence within a single topology and confirm that the effective thermal transport area, arising from the coupled effects of contact perimeter and porosity, serves as the dominant governing parameter.

On the revised manuscript, we have included the correlation plots quantifying the relationship between liquid–solid contact perimeter, porosity, and evaporation performance, and control-variable experiments using FBCC-+ unit cells to isolate the contribution of geometric parameters. These additions clarify the coupling effects and identify the effective thermal transport area as the core regulatory parameter governing evaporation performance.

On the revised manuscript; results

<From>

[Line 214-219] A comparative summary of the average contact perimeter, porosity, and experimentally measured rise height is presented in Figure 3E. The consistent trend across all metrics indicates that designs with larger wetting contact perimeter and lower porosity support faster and taller capillary rise. Collectively, these results establish that capillary transport in ordered porous systems can be predictably tuned through rational geometric design, providing a critical foundation for optimizing subsequent solar evaporation performance.

<To>

[Line 233-238] A comparative summary of the average contact perimeter, porosity, and experimentally measured rise height is presented in Figure 3E and Table S1. A clear correlation is observed across all metrics, where structures with larger wetting contact perimeter and lower porosity exhibit faster wicking kinetics. This trend reflects the combined effect of enhanced capillary adhesion and reduced gravitational resistance, which together promote efficient liquid transport through the lattice, consistent with geometry-governed capillary behavior in 3D porous architectures[4,5].

<Addition; text>

[Line 239-247] To further isolate geometric influences by different unit cell types, water transport was additionally examined within a single topology using the FBCC-+ unit cell as a representative structure. The frame thickness (*F*) and strut diameter (*D*) were varied simultaneously while maintaining a constant unit cell edge length of 2 mm. Among the tested configurations, the FBCC-+ structure with $F = D = 400$ μm exhibited the fastest capillary rise, consistent with its larger liquid–solid contact perimeter and lower porosity (Figure S8 and Table S2). This behavior follows the same trend observed across different unit-cell geometries, confirming that enhanced adhesion forces associated with increased wetted perimeter, together with reduced liquid gravitational resistance from lower porosity, collectively govern water transport performance(Figure S9)[5].

<From>

[Line 258] Consistently, architectures with larger contact area exhibited higher evaporation rates (Figure S6).

<To>

[Line 289-293] Notably, the calculated effective thermal transport areas show strong correlation with the experimentally measured evaporation rates (Figure S14), indicating that heat delivery to the evaporation interface is governed a geometry-dependent solid–liquid contact region. This parameter therefore plays a dominant role in determining evaporation performance.

<Addition; text>

[Line 313-321] To further isolate geometric effects, evaporation performance was examined within a single topology using FBCC-+ unit cells with systematically varied frame thickness (*F*) and strut diameter (*D*), while maintaining a constant unit-cell edge length of 2 mm. The FBCC-+ structure with $F = D = 400$ μm exhibited the highest evaporation rate (2.16 ± 0.06 kg m^{-2} h^{-1}), whereas the structure with $F = D = 200$ μm exhibited the lowest value (1.93 ± 0.16 kg m^{-2} h^{-1}) (Figure S16). This results follows the variation in effective thermal transport area (Figure S16 and Table S6) and it is consistent with the behavior observed across different unit-cell geometries. Collectively, these results confirm that the effective thermal transport area serves as a governing parameter for heat delivery and dominates the evaporation performance.

On the revised supplementary materials

<From>

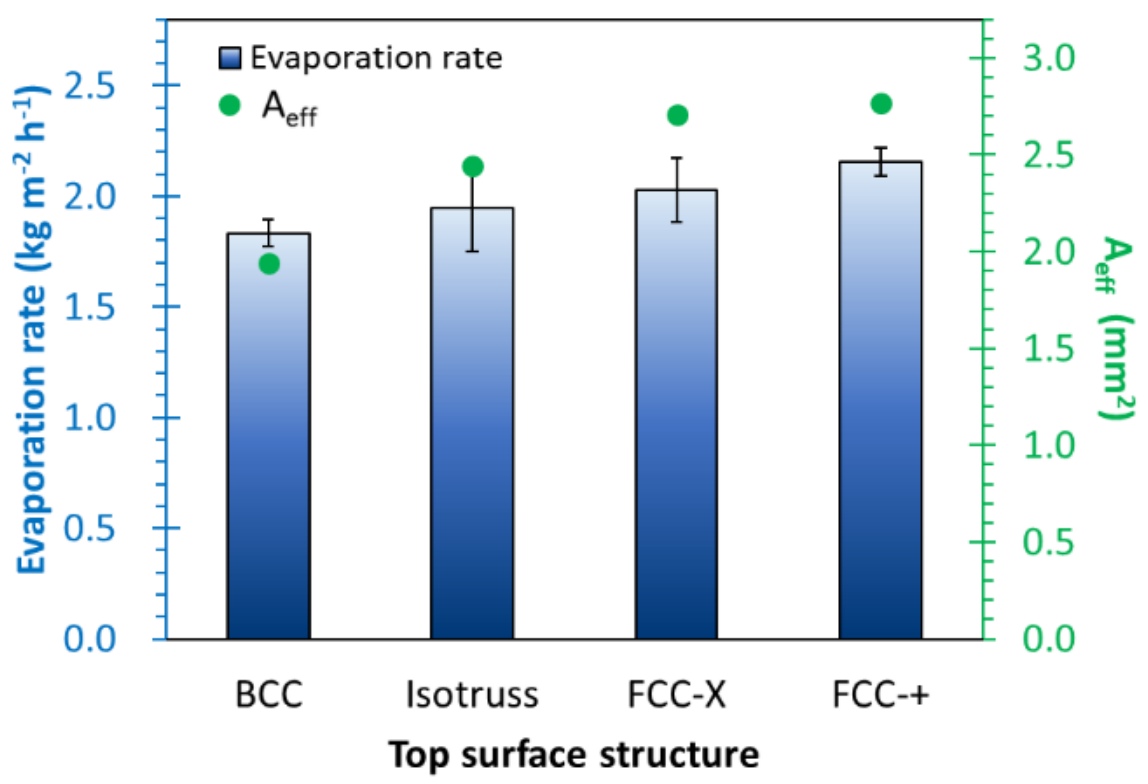

Figure S14. Correlation between evaporation rate and effective thermal transfer area (A_{eff}, of a single unit cell)

<To>

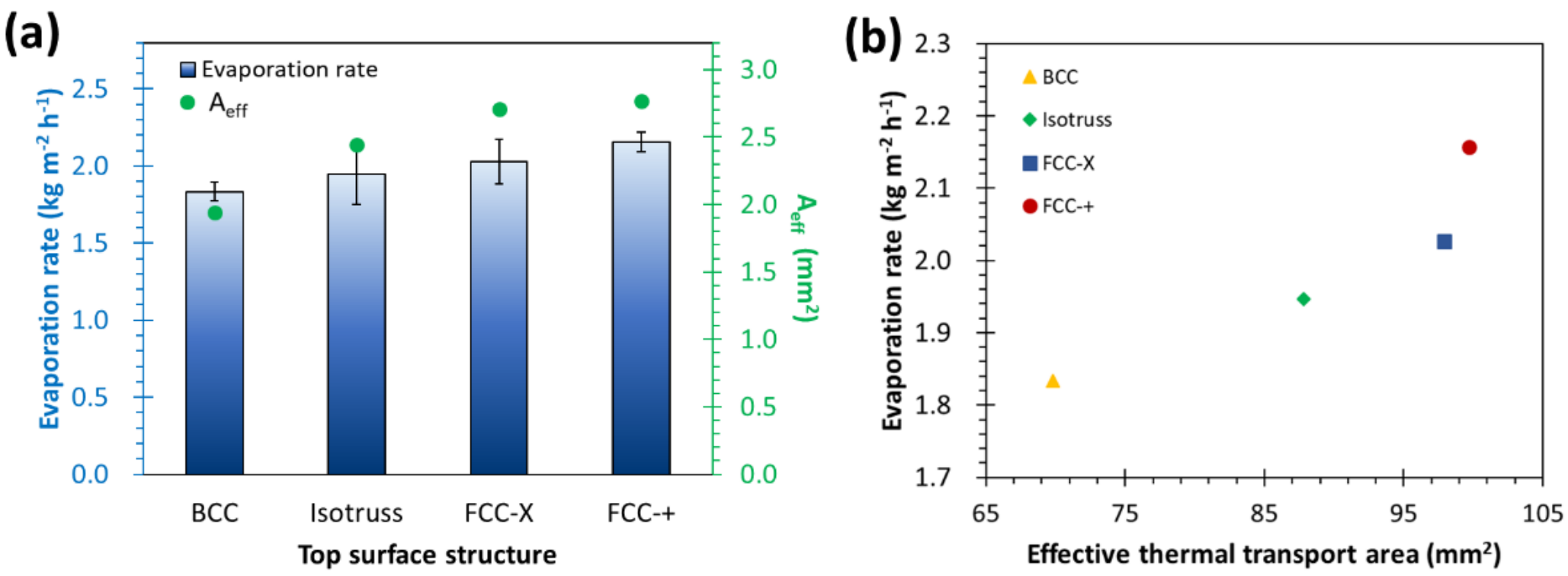

Figure S14. Correlation between evaporation rate and effective thermal transfer area. (a) Comparison of evaporation rates for different top-surface unit-cell architectures together with the effective thermal transport area (A_{eff}) of a single unit cell. (b) Relationship between evaporation rate and the total effective thermal transport area of the corresponding structures.

<Addition; Figure S8>

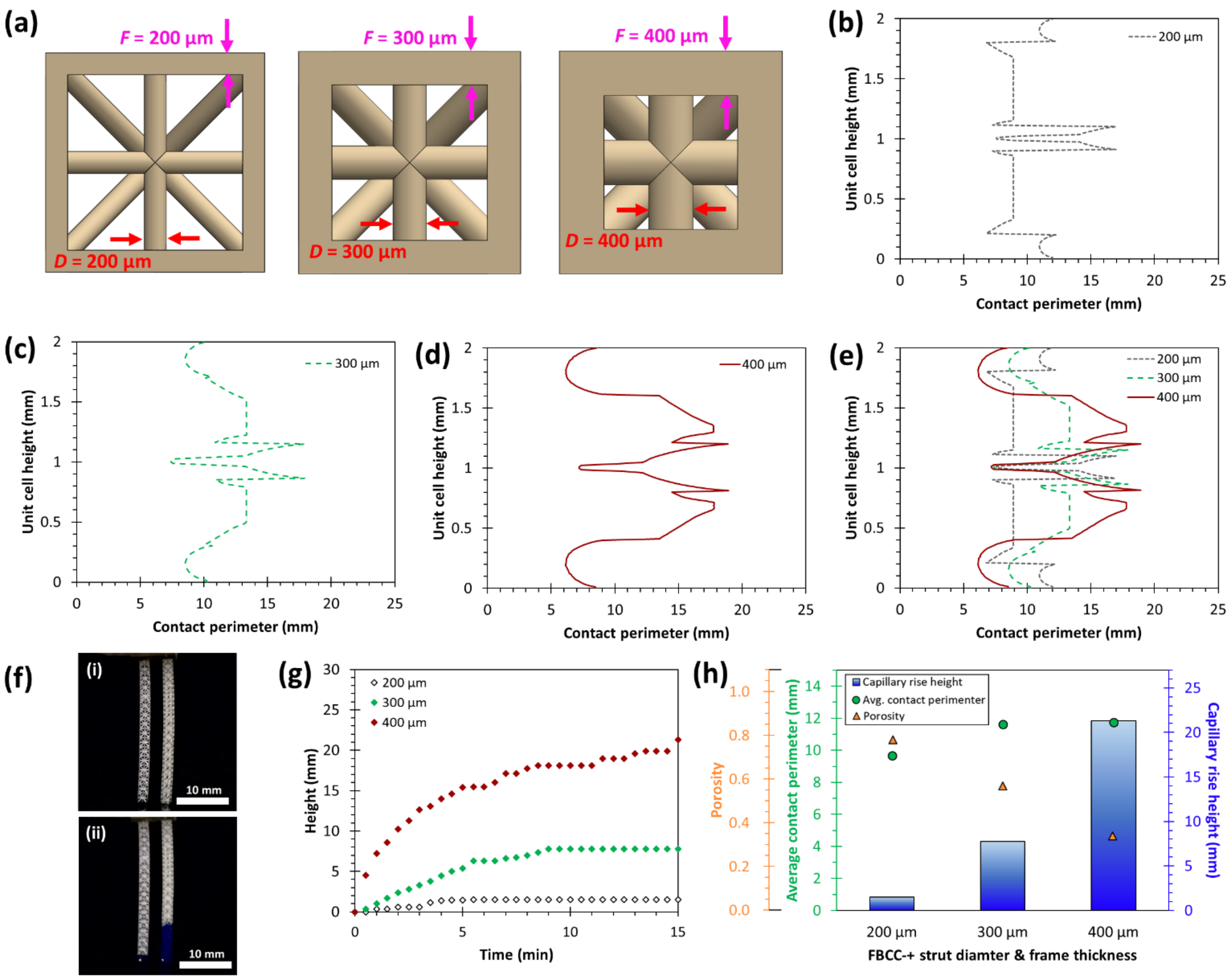

Figure S8. Capillary transport analysis FBCC-+ unit cells with varying frame thickness (*F*) and strut diameter (*D*), with constant unit-cell edge length of 2 mm. (a) Geometrical characteristics of the FBCC-+ structures. (b–d) Contact perimeter as a function of height within FBCC-+ unit cells with F

and D of 200, 300, and 400 μm, respectively; (e) comparison of the three cases. (f) Time-dependent capillary rise images of 200 and 300 μm FBCC-+ cells at (i) initial state and (ii) after 15 min. (g) Corresponding time-dependent capillary rise behavior. (h) Comparative summary of porosity, average contact perimeter, and maximum capillary rise for the different unit-cell geometries.

<Addition; Table S1>

Table S1. Geometric characteristics and capillary rise of different unit cell architectures

Unit cell	Dimensions* ($W \times L \times H$, mm)	Porosity**	Avg. contact perimeter*** (mm)	Capillary rise height**** (mm)
SC	2 × 2 × 2	0.65	3.85	1.39
BCC	2 × 2 × 2	0.54	7.43	3.57
FCC-X	2 × 2 × 2	0.39	8.22	5.91
FCC-+	2 × 2 × 2	0.45	8.11	5.04
Isotruss	2 × 2 × 2	0.47	8.52	6.70
FBCC-X	2 × 2 × 2	0.30	10.10	16.26
FBCC-+	2 × 2 × 2	0.34	11.75	21.31

*Single unit cell

**Porosity $= 1 - \frac{Solid\ \ Volume}{Total\ \ volume}$

***Average liquid–solid contact perimeter of single unit cell (normal to capillary direction)

****After 15 minutes

<Addition; Table S2>

Table S2. Geometric parameters and measured capillary rise of FBCC -+ unit cells with different frame thicknesses and strut diameters at a fixed unit-cell edge length of 2 mm.

Frame thickness (μm)	Strut diameter (μm)	Porosity*	Avg. contact perimeter** (mm)	Capillary rise height*** (mm)
200	200	0.78	9.67	1.52
300	300	0.57	11.62	7.76
400	400	0.34	11.75	21.31

*Porosity $= 1 - \frac{Solid\ \ Volume}{Total\ \ volume}$

**Average liquid–solid contact perimeter of single unit cell (normal to capillary direction)

***After 15 minutes

<Addition; Figure S9>

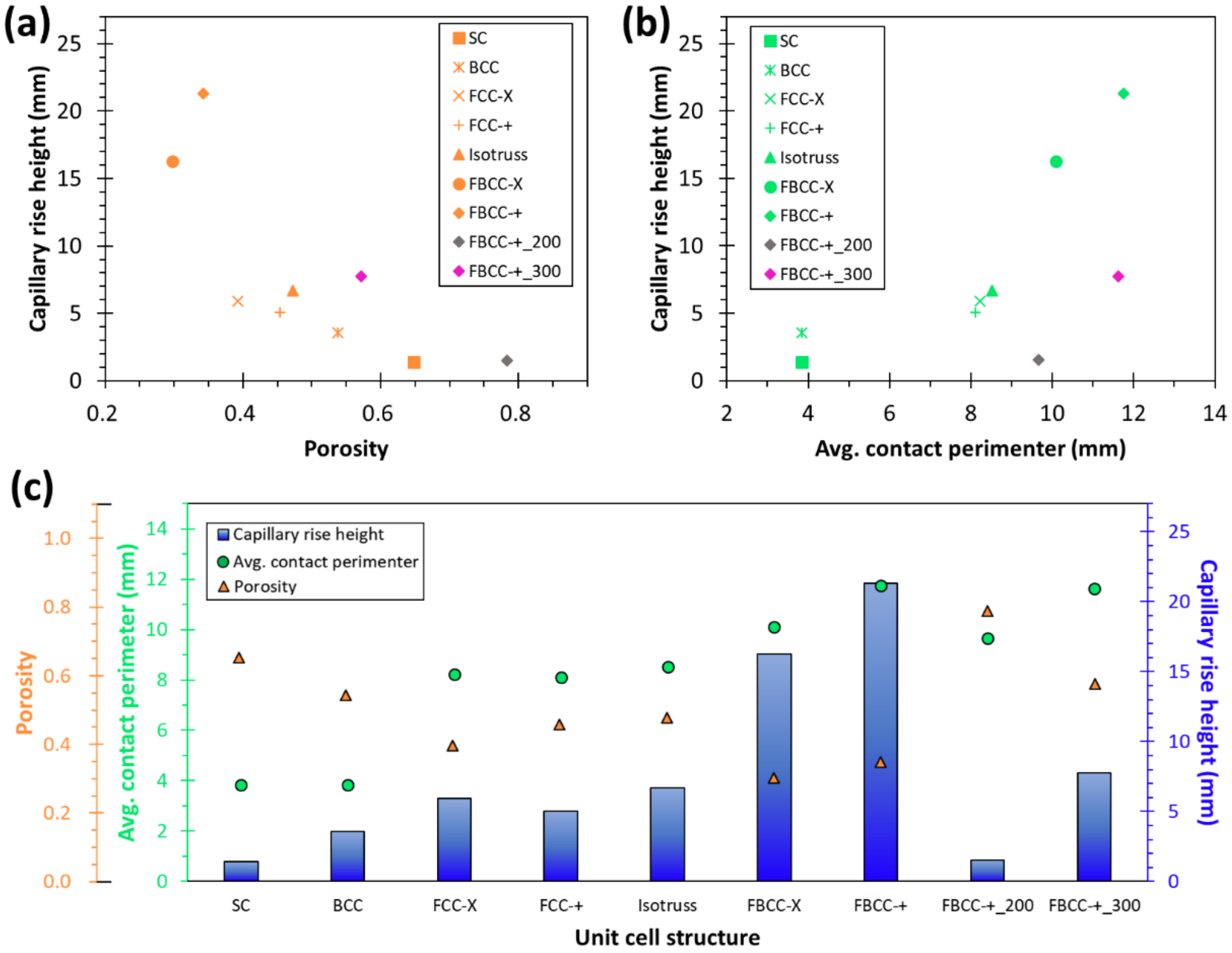

Figure S9. Correlation between geometrical characteristics and capillary performance of different unit cell structures. (a) Relationship between porosity and capillary rise height. (b) Relationship between average contact perimeter and capillary rise height. (c) Comparative summary of porosity, average contact perimeter, and maximum capillary rise for the different unit-cell geometries.

<Addition; Table S5>

Table S5. Effective thermal transport area of different unit cell architectures

Unit cell	Effective thermal transport area* in a single unit cell (mm^2)	Effective thermal transport area in MSE (mm^2)
SC	1.93	69.12
BCC	1.94	69.84
FCC-X	2.72	97.92
FCC-+	2.77	99.72
Isotruss	2.44	87.84
FBCC-X	2.71	97.92
FBCC-+	2.77	99.72

*Calculated as $A_{eff} \approx P_{pore} \cdot H$; P_{pore} : pore perimeter, H : frame thickness

MSE: 36 unit cells on 6 × 6 (width × length) cells configuration

<Addition; Figure S16>

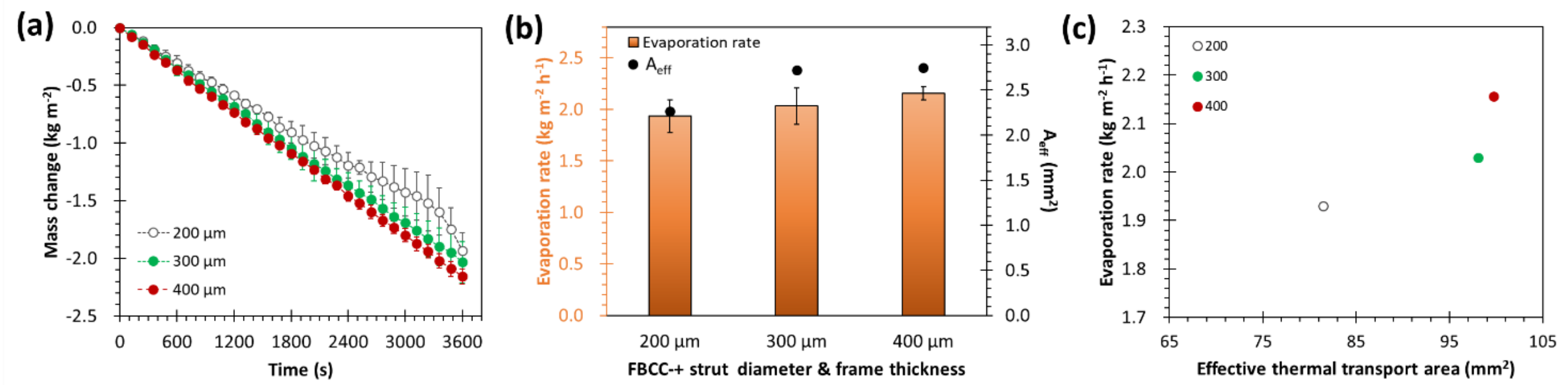

Figure S16. Evaporation performance analysis of FBCC-+ unit cells with varying frame thickness (*F*) and strut diameter (*D*), with constant unit-cell edge length of 2 mm on evaporation rate. (a) Mass change during evaporation, (b) Correlation between evaporation rate and effective thermal transfer area (A_{eff}) of a single unit cell, and (c) Relationship between evaporation rate and the total effective thermal transport area.

<Addition; Table S6>

Table S6. Geometric parameters and evaporation rate of FBCC-+ unit cells with different frame thicknesse and strut diameters at a fixed unit-cell edge length of 2 mm.

Frame thickness (µm)	Strut diameter (µm)	Effective thermal transport area* in a single unit cell (mm^2)	Evaporation rate ($kg\ m^{-2}\ h^{-1}$)
200	200	2.26	1.93
300	300	2.72	2.03
400	400	2.77	2.16

*Calculated as $A_{eff} \approx P_{pore} \cdot H$; P_{pore} : pore perimeter, H : frame thickness

5. In Figures 4B, 4C and S5, the evaporation efficiencies of various shapes were compared. But why was the FBCC sample selected for the subsequent experiments?

Reply: We sincerely thank the reviewer for this valuable comment and for the opportunity to clarify our rationale.

As shown in the original manuscript (Figure 3B, also provided below), the face–body-centered cubic (FBCC) lattices were developed by integrating FCC and BCC unit cells, resulting in two variants (FBCC-X and FBCC-+) depending on the strut configuration at the face planes.

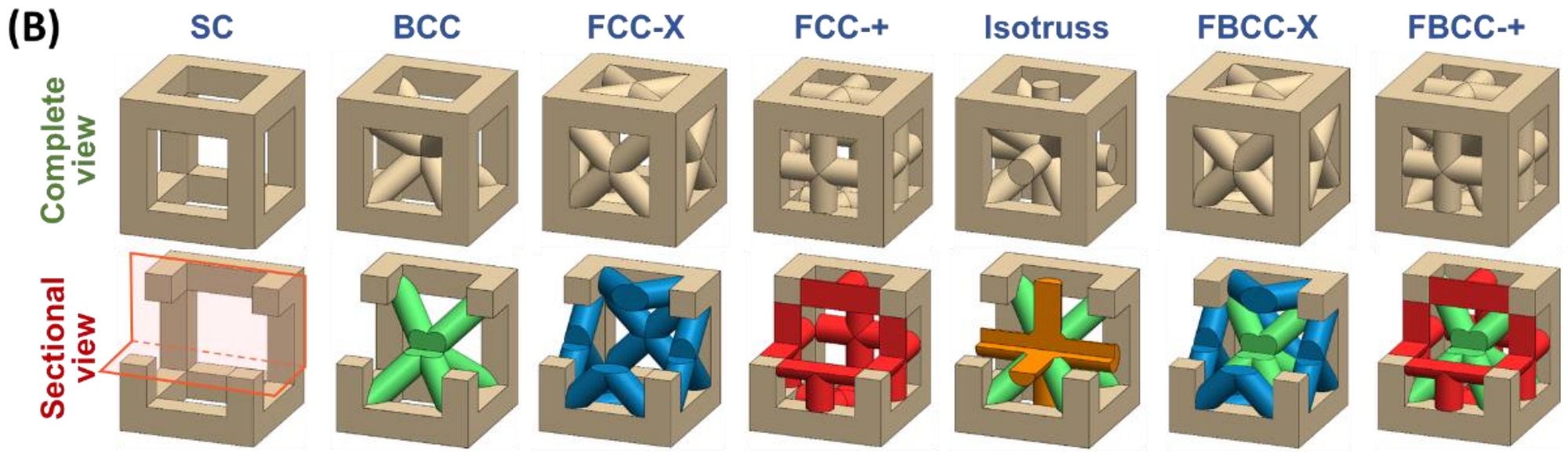

Figure 3. (B) Unit cell types presented with both complete and sectional views for internal structural visualization.

Importantly, capillary-driven water transport and evaporation are governed by different geometric characteristics. Capillary transport is primarily determined by the liquid–solid contact perimeter distributed throughout the unit cell, making the internal architecture a key factor. In contrast, evaporation performance is largely influenced by the effective solid–liquid interfacial region located near the outer pore openings, which is associated with the evaporative face geometry.

In this context, FBCC geometries are particularly advantageous, as they retain the same evaporative face configuration as the FCC structures while incorporating additional internal pathways derived from the BCC architecture. As a result, FBCC structures maintain a comparable effective evaporation interface while simultaneously enhancing capillary-driven water supply. This hybrid characteristic enables efficient liquid replenishment without sacrificing evaporation area.

To further verify this point, control experiments were additionally conducted comparing FCC-+ and FBCC-+ at 0 cm exposed height (Figure S15). The results show comparable evaporation performance between the two structures, consistent with their identical evaporative surface geometry. This confirms that the evaporation interface is preserved in FBCC-+, while the improved performance observed at later stages originates from enhanced capillary transport rather than differences in top-surface evaporation.

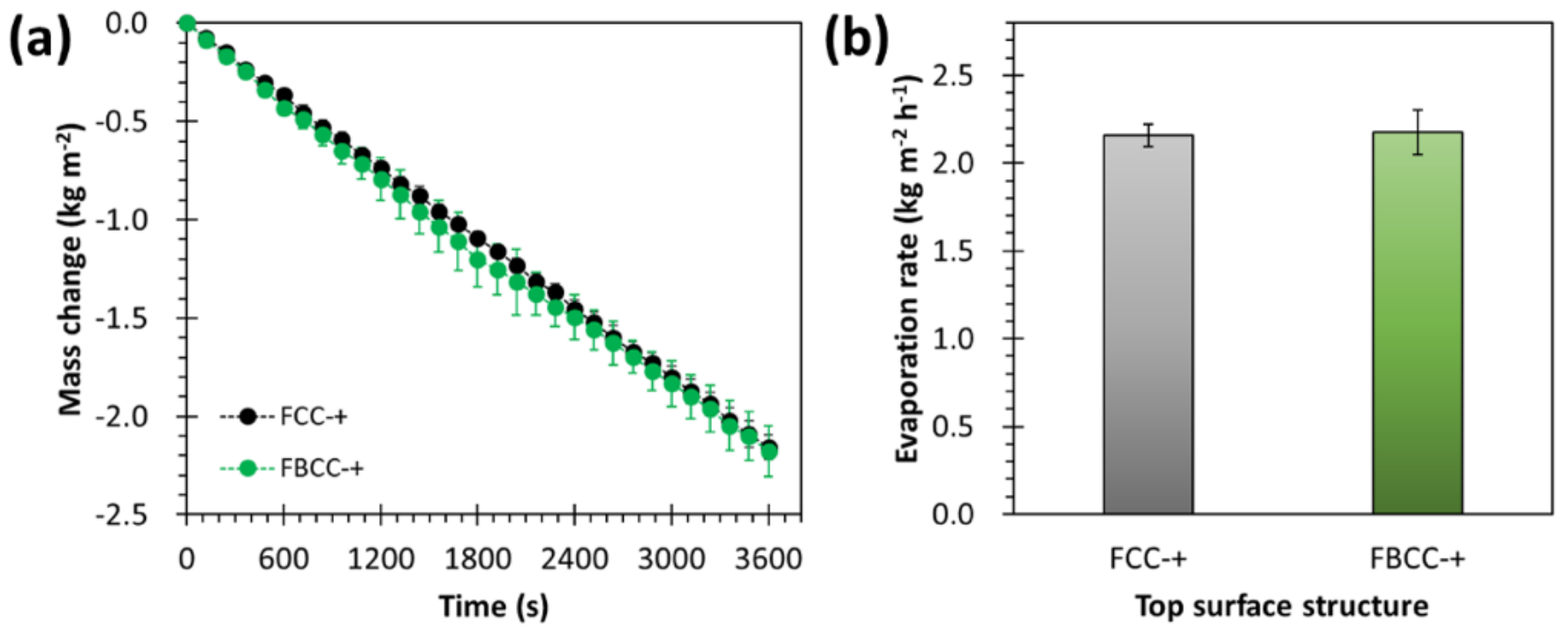

Figure S15. Evaporation performance analysis of FCC-+ and FBCC-+ MSE. (a) Mass change during evaporation, and (b) evaporation rate comparison.

Consistently, among all tested architectures, the FBCC-+ structure exhibited both the highest capillary transport capability and the highest evaporation rate. Based on this combined performance, the FBCC-+ geometry was selected for the subsequent experiments,

including investigations of exposed height effects and long-term stability under saline conditions.

We agree with the reviewer that this point could be more clearly articulated in the manuscript. Accordingly, in the revised manuscript and Supplementary Information, we have included a clarification of the rationale for selecting FBCC-+ for further optimization, along with an additional table summarizing the effective thermal contact area of different unit cell architectures. In addition, the revised manuscript now explicitly states that evaporation measurements at 0 cm confirmed comparable performance between FBCC-+ and FCC-+, consistent with their identical evaporative surface geometry (Figure S14). These additions further support the rationale for selecting FBCC-+ as the representative structure.

On the revised manuscript; results

<From>

[Line 259-261] Based on the combined analyses of water transport and evaporation performance, the FBCC-+ geometry was selected for further optimization, as it retains the same evaporative surface as FCC-+ while providing superior capillary transport.

<To>

[Line 307-313] Based on the combined analyses of water transport and evaporation performance, the FBCC-+ geometry was selected for further optimization. FBCC-+ preserves the same evaporative surface as FCC-+ while exhibiting superior capillary transport and among the highest evaporation rates, making it an optimal candidate for subsequent parametric investigations (Table S5). Evaporation measurements were conducted with only the top surface exposed (i.e. at 0 cm height), confirming comparable performance between FBCC-+ and FCC-+, consistent with their identical evaporative surface geometry (Figure S15).

On the revised supplementary materials

<Addition; Table S5>

Table S5. Effective thermal transport area of different unit cell architectures

Unit cell	Effective thermal transport area* in a single unit cell (mm^2)	Effective thermal transport area in MSE (mm^2)
SC	1.93	69.12
BCC	1.94	69.84
FCC-X	2.72	97.92
FCC-+	2.77	99.72
Isotruss	2.44	87.84
FBCC-X	2.71	97.92
FBCC-+	2.77	99.72

*Calculated as $A_{eff} \approx P_{pore} \cdot H$; P_{pore} : pore perimeter, H : frame thickness

MSE: 36 unit cells on 6 × 6 (width × length) cells configuration

<Addition; Figure S15>

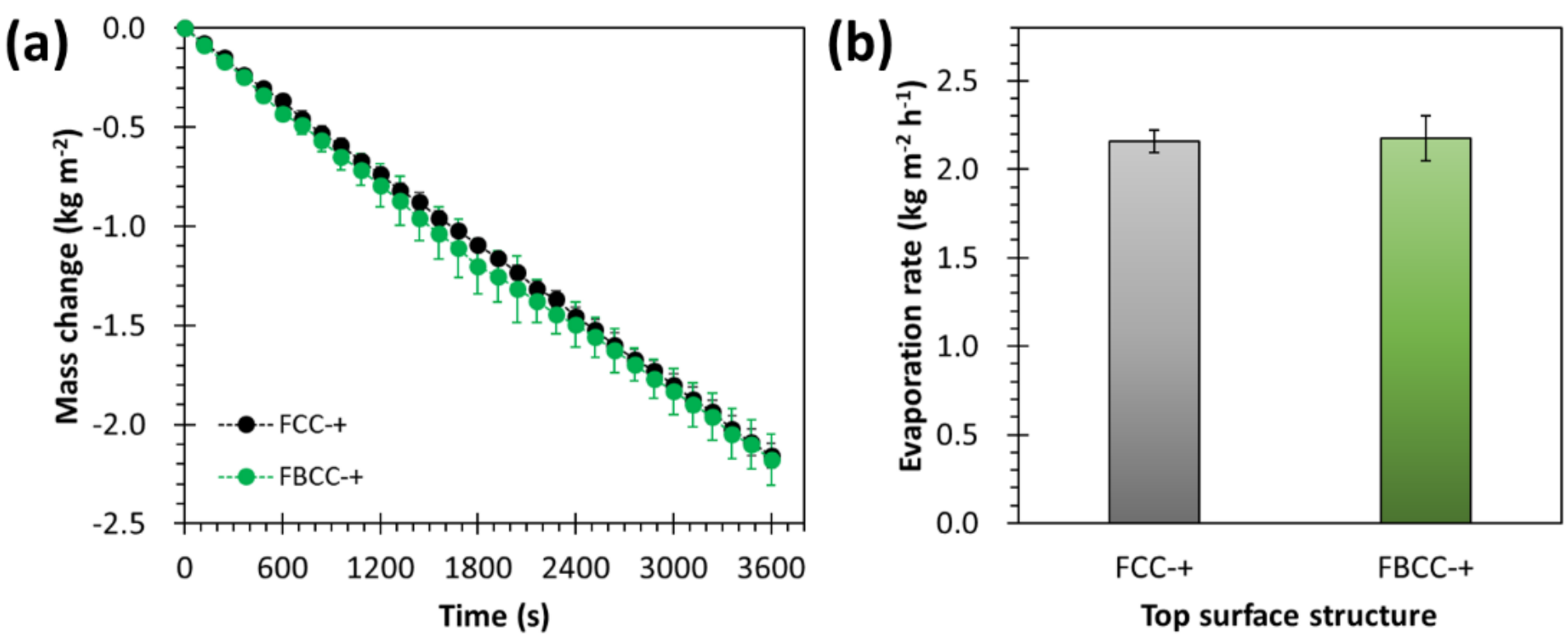

Figure S15. Evaporation performance analysis of FCC-+ and FBCC-+ MSE. (a) Mass change during evaporation, and (b) evaporation rate comparison.

6. This study adopts a system consisting of acrylate-based resin and graphite coating. Is this geometric design strategy applicable to other photothermal materials (e.g., carbon nanotubes, MXene, polypyrrole) and substrate materials?

Reply: Thank you for your insightful question regarding the generality of the proposed geometric design strategy.

The primary focus of this study is to elucidate geometry-dependent transport and evaporation mechanisms, rather than to optimize a specific material system. In this context,

the acrylate-based resin and graphite coating were selected as a representative platform that provides structural integrity, broadband photothermal absorption, and tunable surface wettability. Importantly, the key parameters governing performance, namely capillary-driven water transport (related to liquid–solid contact perimeter) and evaporation (related to the effective thermal transfer area) are fundamentally dictated by the geometric architecture rather than the specific material choice.

Therefore, the geometric design principles demonstrated in this work are expected to be broadly applicable to other photothermal materials, including carbon nanotubes, MXene, and polypyrrole. These materials share common functionalities such as strong light absorption and efficient photothermal conversion, and can be integrated with similar 3D architectures via coating, infiltration, or composite fabrication strategies[2,6]. Similarly, the choice of substrate material is not intrinsically limited to acrylate-based resin. Any substrate that can maintain structural stability, allow fluid transport, and support surface modification (e.g., polymers, ceramics, or biomass-derived frameworks) could be utilized, provided that appropriate surface treatment is applied to ensure hydrophilicity and coating adhesion.

While the absolute evaporation performance may vary depending on material-specific properties (e.g., optical absorption, thermal conductivity, and surface chemistry), the underlying geometric effects and design guidelines identified in this study remain valid and transferable.

To clarify this point, we have added a discussion in the revised manuscript highlighting the material-independent nature of the proposed geometric strategy and its potential applicability to a broader range of photothermal systems.

On the revised manuscript; discussion

<From>

[Line 468-473] Collectively, these results demonstrate that unit cell and macro-scale geometry can be used as programmable design variables to control interfacial transport, heat localization, and salt deposition pathways in solar evaporators. The structure-function relationships identified here provide a generalizable framework for designing scalable, durable, and high-efficiency solar evaporation systems, advancing solar-driven desalination and ZLD toward real-world implementation.

<To>

[Line 572-580] Collectively, these results demonstrate that unit cell and macro-scale geometry can be used as programmable design variables to control interfacial transport, heat localization, and salt deposition pathways in solar evaporators. The structure–function relationships identified here suggest

general design principles under controlled material and wettability conditions. While these geometric effects are expected to be broadly applicable, the absolute performance may depend on material-specific properties, including wettability, optical absorption, and photothermal conversion efficiency. This work thus establishes a geometry-driven framework for designing scalable, durable, and high-performance solar evaporation systems, advancing solar-driven desalination and ZLD toward real-world implementation.

7. In Section 4.3 (Water transport experiments), the manuscript notes treatment of the structures with 1 wt% PVA to ensure hydrophilicity. Please provide detailed process parameters and address the following questions:

a. Was the PVA treatment applied in the solar evaporation performance tests? If not, clearly justify why this treatment is required for capillary transport tests but not for evaporation performance tests.

b. Elucidate whether PVA treatment affects the photothermal conversion efficiency and surface hydrophilicity of the graphite coating. Supplement characterization data (optical absorption spectra, contact angle measurements) for PVA-treated samples to eliminate PVA-induced interference on evaporator performance, and thoroughly discuss the potential limitations and impacts of this treatment.

Reply: Thank you for the careful and detailed comments regarding the use of PVA treatment in the capillary transport experiments.

We agree that clarifying the purpose of the PVA coating and its potential influence on evaporation performance is important.

(a) The PVA treatment was used exclusively for the capillary transport visualization experiments and was not applied in the solar evaporation performance tests. In the revised manuscript, we clarified that the PVA coating was introduced solely to enable clear visualization of liquid transport using methylene blue, as the graphite-coated structures are optically dark and hinder direct observation of the wetting front within the lattice. To ensure that the capillary driving force remains comparable, the surface wettability of the PVA-coated samples was characterized and compared with that of the graphite-coated and O_2 plasma–treated evaporators. The PVA-coated structures exhibited a contact angle of 43.8 ± 5.9°, which is comparable to that of the graphite-coated and O_2 plasma–treated samples (44.7 ± 8.8°), confirming similar hydrophilicity and capillary driving conditions. Therefore, the PVA-

coated samples were used only for visualization purposes, while all evaporation experiments were conducted using the graphite-coated and O_2 plasma–treated structures.

(b) Because the PVA coating was not used in the solar evaporation experiments, it does not affect the reported photothermal conversion or evaporation performance. The optical absorption and photothermal characteristics presented in the manuscript correspond exclusively to the graphite-coated samples. The role of the PVA layer was limited to providing a visually trackable hydrophilic surface for capillary transport observation. Moreover, the comparable contact angle values between the PVA-coated and graphite-coated samples indicate that the PVA treatment does not introduce a significant change in hydrophilicity, ensuring that the capillary transport comparison remains representative of the actual evaporator behavior.

In the revised manuscript we have clarify that PVA coating was used only for visualization of capillary transport and that comparable contact angles confirm similar capillary driving forces between the two cases.

On the revised manuscript; results

<Addition; text>

[Line 225-232] Capillary rise experiments were performed to validate this prediction (Figure 3D). For visualization, poly(vinyl alcohol) (PVA)-coated samples were used instead of the graphite-coated, O_2 plasma–treated evaporators, enabling clear observation of methylene blue transport within the lattice. The PVA-coated structures exhibited a contact angle (43.8 ± 5.9°) comparable to that of the graphite-coated and O_2 plasma–treated samples (44.7 ± 8.8°), ensuring similar capillary driving forces (Figure S6). The experiments confirm the geometric trend, with the FBCC-+ architecture exhibits both the fastest rise kinetics and the highest equilibrium water elevation within 15 min of wicking (Figure S7 and Movie S1).

On the revised supplementary materials

<Addition; Figure S6>

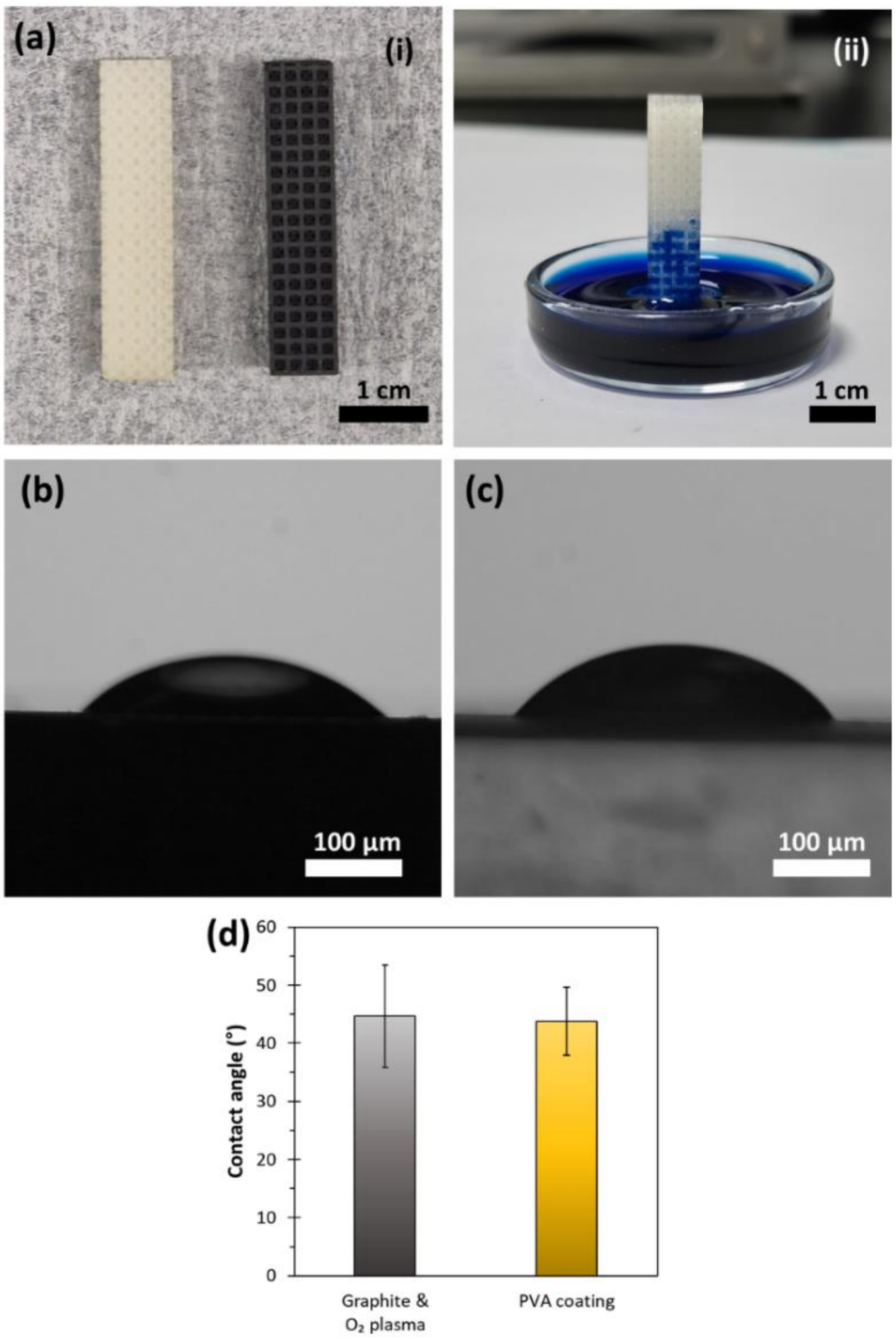

Figure S6. Rationale for using PVA-coated samples for water-transport visualization. (a) Optical images of (i) multicellular structures coated with PVA (left) and graphite only (right), and (ii) water transport visualization in the PVA-coated sample. Contact angle images of (b) graphite-coated surface after O_2 plasma treatment and (c) PVA-coated surface. (d) Comparison of the measured contact angles for each condition.

8. It is suggested to conduct simulation analysis on the impact of different geometric structures on water transport efficiency.

Reply: We thank the reviewer for this thoughtful suggestion. We agree that simulation can be a valuable tool for visualizing local flow fields, transient interface evolution, and pressure distribution in complex 3D architected structures. However, after carefully considering the scope and objective of the present study, we believe that additional simulation is not essential for supporting our main conclusions.

The reason is that the geometry-dependent water transport behavior in open-cell/lattice structures is already well established in the literature[7-9]. Capillary-driven water transport in ordered lattice structures is primarily governed by geometric parameters such as effective pore size, liquid–solid contact perimeter, unit-cell topology, and the balance between capillary adhesion and gravity. Previous studies have shown that capillary rise in periodic lattices can be quantitatively described using classical capillary theory, including Young–Laplace pressure and force-balance analysis, with experimental measurements closely matching analytical predictions[7,9]. These works demonstrate that geometry-dependent trends in capillary transport can be reliably interpreted using structural descriptors, while simulations mainly provide complementary visualization of transient interface dynamics rather than altering the governing physics[7]. Other recent studies on 3D printed capillary lattices similarly report that liquid transport efficiency can be systematically tuned by modifying unit-cell geometry, and that experimental observations agree well with analytical capillary models[5,8,10,11].

Consistent with this established framework, the objective of the present work is to experimentally evaluate how different unit-cell architectures influence water transport efficiency and subsequent evaporation behavior under identical conditions. The experimentally observed trends follow the expected geometry-dependent capillary behavior: structures with larger liquid–solid contact perimeter and lower porosity exhibit faster capillary rise kinetics and higher equilibrium heights (Figure 3D, 3E, and Table S1). These results directly reflect the balance between adhesive capillary forces and gravitational resistance, confirming that the observed differences originate from structural geometry rather than requiring additional numerical validation.

To acknowledge the reviewer’s suggestion in a constructive manner, we have revised the manuscript to more explicitly discuss the physical basis of geometry-dependent water transport and added relevant references to prior studies on capillary-driven transport in ordered lattices. We also note that future numerical simulations could provide complementary insight into transient filling dynamics and local flow behavior within individual unit cells. However, such analysis is beyond the scope of the present study, whose primary focus is experimental demonstration and structure–property–performance relationships.

On the revised manuscript; results

<From>

[Line 215-217] The consistent trend across all metrics indicates that designs with larger wetting contact perimeter and lower porosity support faster and taller capillary rise.

<To>

[Line 234-238] A clear correlation is observed across all metrics, where structures with larger wetting contact perimeter and lower porosity exhibit faster wicking kinetics. This trend reflects the combined effect of enhanced capillary adhesion and reduced gravitational resistance, which together promote efficient liquid transport through the lattice, consistent with geometry-governed capillary behavior in 3D porous architectures[4,5].

9. Did the excessive accumulation of salt on the BCC surface affect the photothermal efficacy?

Reply: We thank the reviewer for this important question regarding the influence of salt accumulation on photothermal performance.

In the hybrid multicellular evaporator, salt crystallization occurs predominantly on the outer BCC region, while the primary photothermal evaporation surface (top and inner surface consisted of FBCC-+ cells) remains largely free of salt deposition. Therefore, the light-absorbing surface responsible for heat generation is preserved, minimizing any direct reduction in photothermal efficacy.

Furthermore, the accumulated salt on the BCC region forms a hydrated porous structure rather than a dense blocking layer. Such porous salt deposits allow continued liquid transport and vapor escape, and do not significantly suppress photothermal conversion[2]. Similar behavior has been reported in previous studies, where porous or dendritic salt layers maintained evaporation performance and even functioned as auxiliary evaporation media rather than passivating layers (Yu et al.[12]; Ding et al.[13]). In these systems, the salt layer remains partially wetted and thermally coupled to the underlying structure, allowing continued heat transfer and evaporation.

Consistently, our experimental results show that the evaporation rate of the hybrid evaporator remains stable during prolonged operation, even after substantial salt accumulation on the BCC region (Figure 5G). No measurable degradation in evaporation performance was observed, indicating that the photothermal efficacy was not significantly affected. In addition, the observed slight increase in evaporation rate after extended operation further supports that the porous salt layer expands the effective evaporation interface instead of blocking solar absorption.

These observations confirm that salt accumulation on the BCC region does not deteriorate the photothermal performance, because crystallization occurs away from the primary photothermal surface, and the formed porous salt layer remains transport-active and thermally coupled to the evaporation structure.

In the revised manuscript, we have included additional discussion of this phenomenon to clarify why the evaporation rate remained unchanged and subsequently increased during long-term evaporation performance experiments.

On the revised manuscript; results

<Addition; text>

[Line 460-469] The increased evaporation rate observed after the second day can be attributed to the formation of a porous salt layer. Similar to the homogeneous FBCC-+_5 MSE, hydrated porous salt crystals develop on the outer surface of the structure. These porous deposits enable continuous brine uptake through capillary transport while growing outward without blocking liquid pathways[12]. Moreover, as shown in Figure 5G (inset), the salt crystals progressively extend outward, forming a coral-like porous network. This evolving porous salt structure effectively expands the evaporation interface and continuously harvests environmental heat, allowing the salt layer to function as an auxiliary evaporating medium rather than a failure mode[13]. This wet-crystal evaporation mechanism enables the cup-shaped hybrid MSE to sustain high-flux evaporation under extreme salinity.

10. Line 129: The paper attributes the absorption bands at 1236-1135 cm^{-1} to C-O and C-O-C stretching vibrations, yet they are labeled as O-H and C-O-C in Figure 2C; this inconsistency should be corrected.

Reply: Thank you for carefully pointing out this inconsistency between the manuscript and the label on Figure 2C.

We acknowledge that the labeling in Figure 2C was incorrect. The absorption bands in the 1236–1135 cm^{-1} region correspond to C–O and C–O–C stretching vibrations, and the previous assignment in the figure was erroneous. This has now been corrected in the revised manuscript to ensure full consistency with the discussion in the main text.

We really appreciate the reviewer’s careful attention to detail, which has helped improve the accuracy and clarity of the manuscript.

On the revised manuscript; Figure 2C

<From>

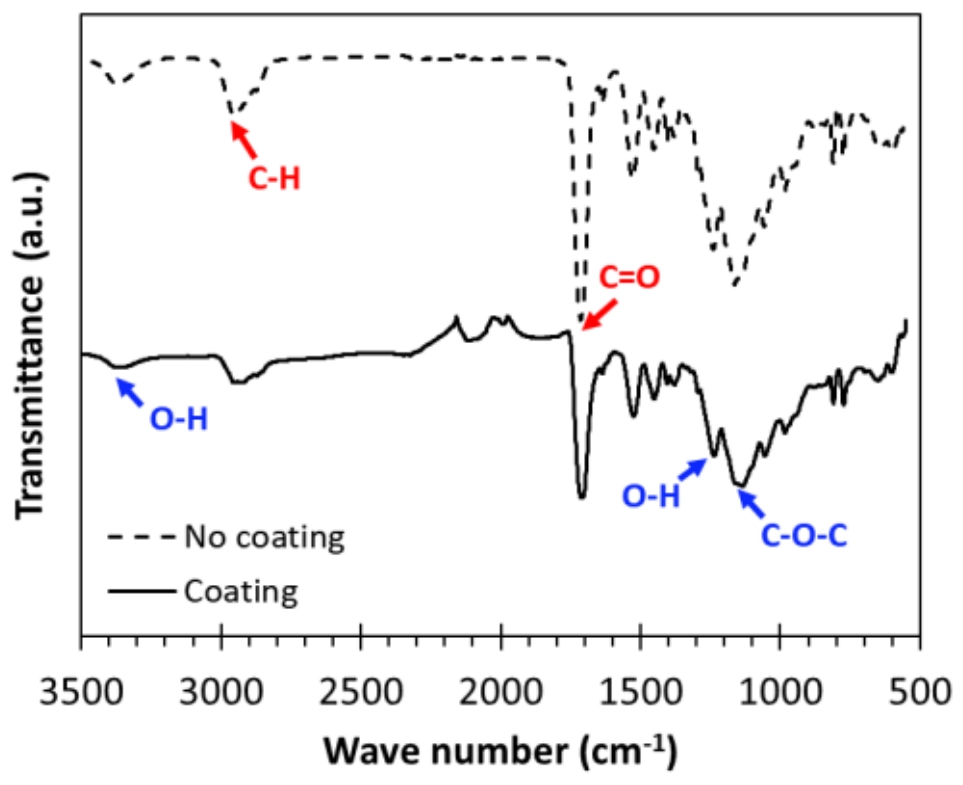

<To>

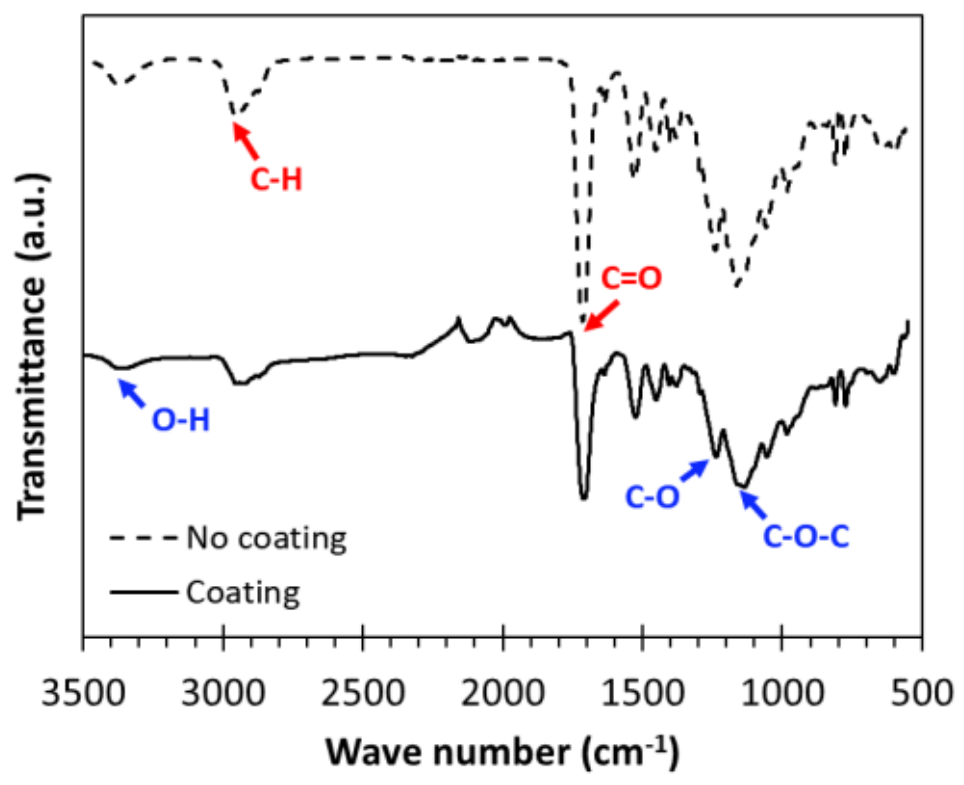

11. Line 246: The unit of the evaporator's evaporation rate under dark conditions is annotated incorrectly. It should be revised from g m^{-2} h^{-1} to kg m^{-2} h^{-1} to maintain consistency with the unit notations in the context and Figure S5.

Reply: We appreciate your constructive comments regarding the inconsistency in the unit notation in the manuscript and Figure S5.

We acknowledge that the unit of the evaporation rate under dark conditions was incorrectly reported as g m^{-2} h^{-1} due to a typographical error. The correct unit is kg m^{-2} h^{-1}, consistent with the notation used throughout the manuscript and in Figure S5.

This error has been corrected in the revised manuscript to ensure consistency and accuracy. In addition, we have carefully reviewed the manuscript and corrected other minor typographical and formatting inconsistencies where necessary. We greatly appreciate the reviewer's attention to detail, which has helped improve the overall quality and clarity of the manuscript.

On the revised manuscript; results

<From>

[Line 245-247] Notably, all architectures also exhibited distinct evaporation rates under dark conditions, ranging from 0.40 to 0.57 g m^{-2} h^{-1}, exceeding …

<To>

[Line 267-268] Notably, all architectures also exhibited distinct evaporation rates under dark conditions, ranging from 0.40 to 0.57 kg m^{-2} h^{-1}, exceeding …

On the revised manuscript; Figure 3D

<From>

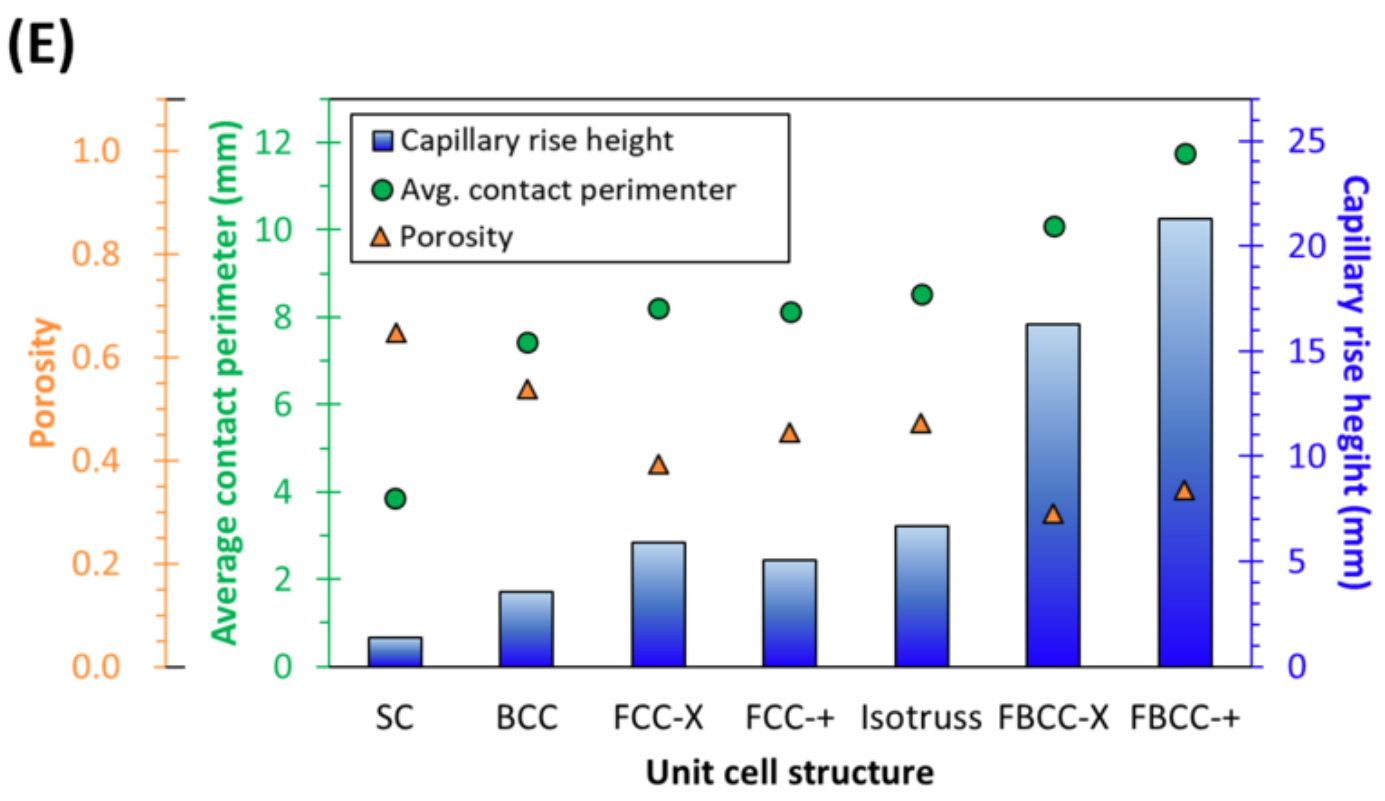

<To>

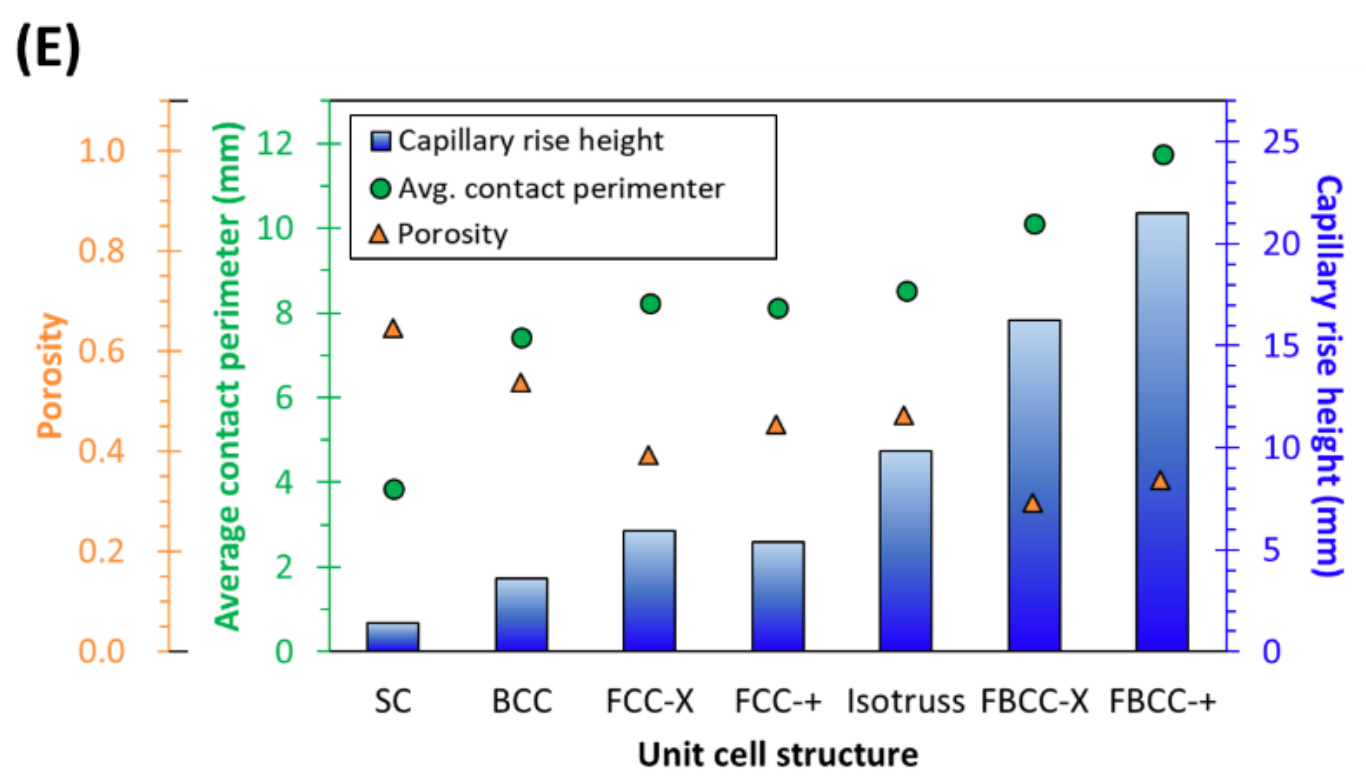

Reviewer #2: This manuscript reports a systematic investigation of 3D-printed multicellular solar evaporators with tailored lattice unit cells, aiming to elucidate the geometry-transport-performance relationships for high-salinity solar evaporation and spatial salt crystallization. The research topic is highly relevant to the sustainable desalination field, and the proposed geometry-engineered design strategy for solar evaporators presents novel insights. The experimental design is relatively systematic, and the obtained results show promising potential for scalable solar desalination applications. However, the manuscript suffers from several critical limitations. Major revisions are required to address these issues and improve the scientific rigor and completeness of the work. Detailed comments are as follows:

1. The liquid-solid contact perimeter and porosity are identified as key geometric parameters governing evaporation rates, while the projected solid area of unit cells directly determines the light-harvesting area, a critical factor correlated with solar evaporation performance. Especially for the 2 mm × 2 mm × 2 mm unit cells used in this study, the influence of projected solid area cannot be neglected. The authors are requested to supplement the projected solid area data of all investigated unit cells (SC, BCC, FCC-X, FCC-+, Isotruss, FBCC-X, FBCC-+), and analyze the contribution of light-harvesting area to the evaporation performance of each unit cell to improve the comprehensiveness of the geometric parameter-evaporation rate correlation system.

Reply: Thank you for this insightful comment regarding the potential role of projected solid area in governing evaporation performance.

Following the reviewer's comment, we quantified the projected solid area, denoted as light harvesting area in the manuscript, of all investigated unit cells (Table S4) and examined its correlation with the measured evaporation performance. Accordingly, the projected solid area was defined as the projected solid area of the topmost surface directly exposed to incident irradiation.

The calculated projected solid areas of the unit cells were 48.16 mm^2 (BCC), 52.84 mm^2 (Isotruss), 85.52 mm^2 (FCC-X), and 76.96 mm^2 (FCC-+). The FBCC-based architectures share the same evaporative face geometry and projected light-harvesting area as their corresponding FCC counterparts (i.e., FCC-X = FBCC-X and FCC-+ = FBCC-+). Despite these substantial differences in projected area, the measured top-surface temperatures during evaporation were nearly identical, with values of 35.12 °C (BCC), 35.87 °C (Isotruss), 36.17 °C (FCC-X), and 36.36 °C (FCC-+). More importantly, the projected solid area does not follow the experimentally observed evaporation-rate trend. For instance, although FCC-X exhibits the largest projected area, its evaporation performance was lower to that of FCC-+, 2.03 kg m^{-2} h^{-1} and 2.16 kg m^{-2} h^{-1} respectively, and the overall trend does not correlate

with projected area. These results indicate that the projected light-harvesting area alone does not govern the evaporation performance under the present configuration.

Instead, the observed evaporation behavior suggests that the additional absorbed energy associated with larger projected areas is not efficiently delivered to the liquid at the evaporation interface. Therefore, the evaporation process is governed by the ability to deliver heat to the liquid at the evaporation interface, rather than by the total absorbed energy.

In the present structures, this heat-delivery capability is determined by the solid–liquid interfacial region surrounding the pores, where evaporation predominantly occurs. Accordingly, we define an effective thermal transport area (A_{eff}) as

$$A_{eff} \approx P_{pore} \cdot H$$

and the rate of heat delivery scales as[14]

$$\mathrm{Q} \propto \; k_s \cdot A_{eff}$$

where P_{pore} is the liquid–solid contact perimeter surrounding the pore and H is the frame thickness and k_s is thermal conductivity of the lattice material. Since all structures were fabricated from the same material, differences in evaporation performance originate from variations in effective solid–liquid contact area.

This parameter represents the solid–liquid interfacial region through which heat is conducted from the photothermal solid to the liquid at the active evaporation sites.

The calculated effective thermal transport areas were 69.84 mm^2 (BCC), 87.84 mm^2 (Isotruss), 97.92 mm^2 (FCC-X), and 99.72 mm^2 (FCC-+). Notably, this parameter follows the same trend as the measured evaporation performance (Figure S13), in contrast to the projected solid area. These results indicate that the effective thermal transport area provides a more appropriate geometric descriptor for evaporation performance under the 0 cm height condition, where top-surface evaporation dominates.

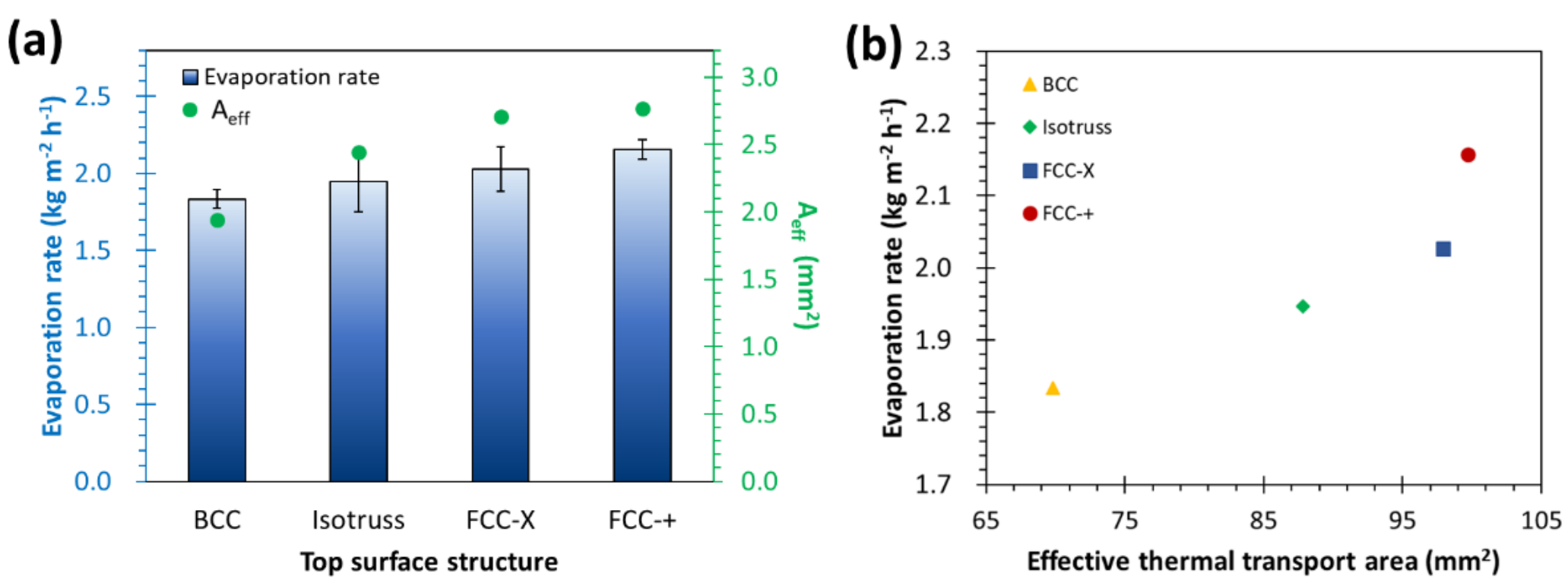

Figure S14. Correlation between evaporation rate and effective thermal transfer area. (a) Comparison of evaporation rates for different top-surface unit-cell architectures together with the effective thermal

transport area (A_{eff}) of a single unit cell. (b) Relationship between evaporation rate and the total effective thermal transport area of the corresponding structures.

To address the reviewer's suggestion, we have expanded the discussions on the revised manuscript and included the projected solid area for all investigated unit cells in the revised supplementary information.

On the revised manuscript; results

<Addition; text>

[Line 269-277] To identify the geometric characteristics governing the observed evaporation differences, the light-harvesting area, defined as the projected solid area of the uppermost surface directly exposed to incident irradiation, was quantified (Table S4). Despite substantial variation in light-harvesting area, ranging from 48.16 to 85.52 mm^2, the surface temperatures during evaporation remained nearly identical (35.12–36.36 °C; Figure 4C and S11), and no correlation with evaporation rate was observed (Figure S13). For instance, although FCC-X exhibits the largest light-harvesting area, its evaporation performance was lower to that of FCC-+. These results indicate that the projected light-harvesting area alone does not govern the evaporation performance under the present configuration.

<From>

[Line 248-258] Since surface temperature alone cannot explain these differences, the observed variations are attributed to geometry-dependent heat transfer through the solid-liquid interface. Assuming that thermal energy is primarily transferred through regions of solid-liquid contact, the effective thermal transfer area can be approximated as:

$$A_{eff} \approx P_{pore} \cdot H$$

and the rate of heat delivery scales as[14]

$$Q \propto k_s \cdot A_{eff}$$

where P_{pore} is the pore perimeter, H is the frame thickness and k_s is thermal conductivity of the lattice material. Since all structures were fabricated from the same material, differences in evaporation performance originate from variations in effective solid–liquid contact area. Consistently, architectures with larger contact area exhibited higher evaporation rates (Figure S6).

<To>

[Line 278-293] Since surface temperature and light-harvesting area cannot explain these differences, the observed variations are attributed to geometry-dependent heat transfer through the solid-liquid interface. Assuming that thermal energy is primarily transferred through regions of solid-liquid contact near the pore edges, the effective thermal transfer area can be approximated as:

$$A_{eff} \approx P_{pore} \cdot H$$

and the rate of heat delivery scales as[14]

$$Q \propto \ k_s \cdot A_{eff}$$

where P_{pore} is the pore perimeter, H is the frame thickness and k_s is thermal conductivity of the lattice material. This effective thermal transport area represents the solid–liquid interfacial region through which heat is delivered from the photothermal solid to the liquid at the active evaporation sites. Since all structures were fabricated from the same material, differences in evaporation performance originate from variations in effective solid–liquid contact area. Notably, the calculated effective thermal transport areas show strong correlation with the experimentally measured evaporation rates (Figure S13), indicating that heat delivery to the evaporation interface is governed a geometry-dependent solid–liquid contact region. This parameter therefore plays a dominant role in determining evaporation performance.

On the revised supplementary materials

<Addition; Table S4>

Table S4. Light-harvesting area of different unit cell architectures

Unit cell	Light-harvesting area in a single unit cell (mm^2)	Light-harvesting area in MSE (mm^2)
SC	2.26	48.16
BCC	2.26	48.16
FCC-X	3.60	85.52
FCC-+	3.36	76.96
Isotruss	2.39	52.84
FBCC-X	3.60	85.52
FBCC-+	3.36	76.96

Light harvesting area: Projected solid area of the uppermost surface directly exposed to incident illumination.

MSE: 36 unit cells on 6 × 6 (width × length) cells configuration

<Addition; Figure S12>

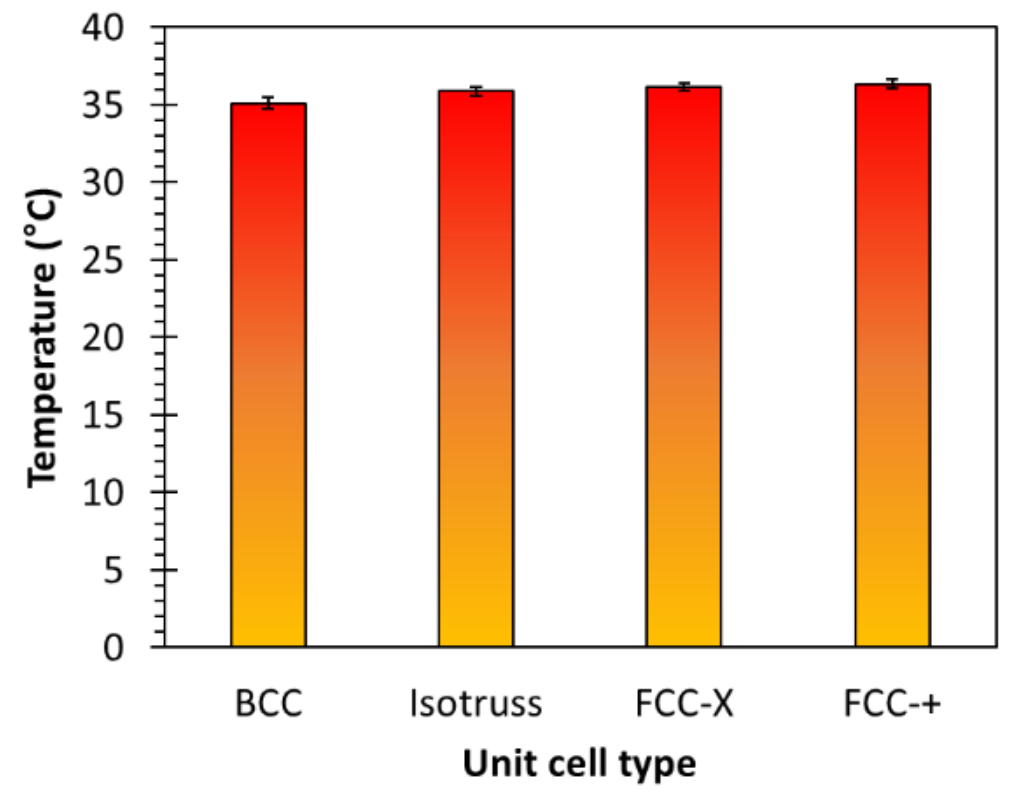

Figure S12. Average surface temperature during evaporation for different unit-cell architectures.

<Addition; Figure S13>

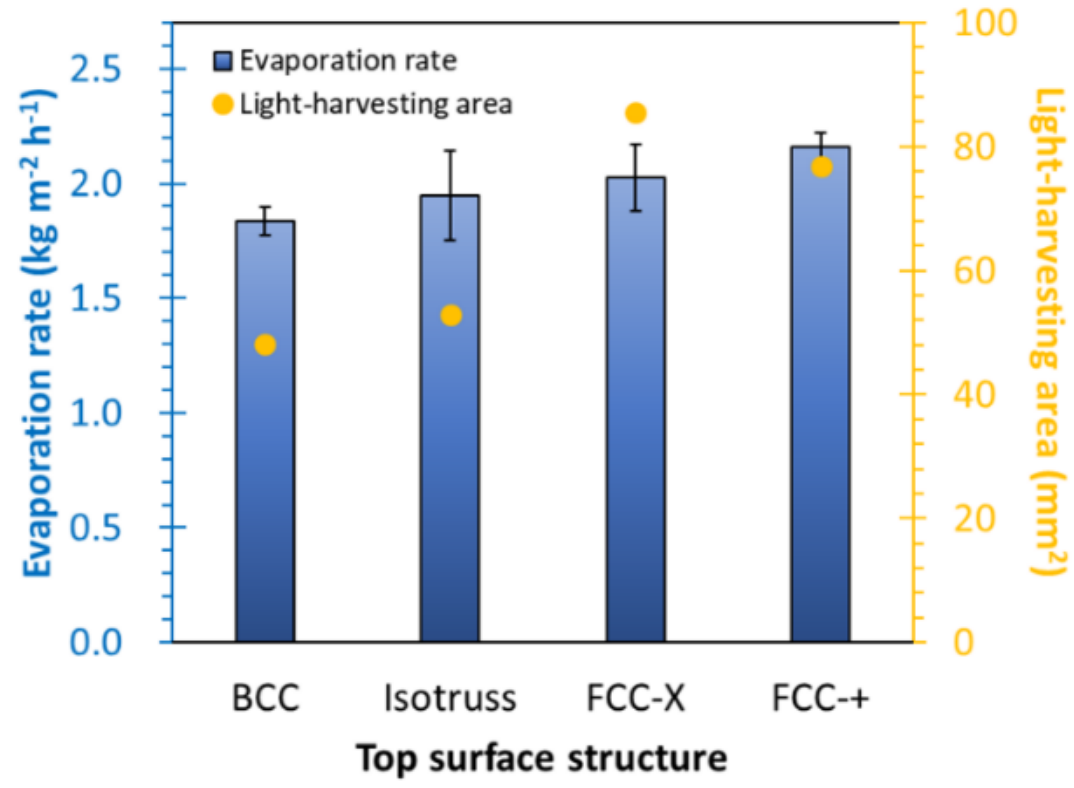

Figure S13. Correlation between evaporation rate and light-harvesting area.

<Addition; Table S5>

Table S5. Effective thermal transport area of different unit cell architectures

Unit cell	Effective thermal transport area* in a single unit cell (mm^2)	Effective thermal transport area in MSE (mm^2)
SC	1.93	69.12
BCC	1.94	69.84
FCC-X	2.72	97.92
FCC-+	2.77	99.72
Isotruss	2.44	87.84
FBCC-X	2.71	97.92
FBCC-+	2.77	99.72

*Calculated as $A_{eff} \approx P_{pore} \cdot H$; P_{pore} : pore perimeter, H : frame thickness

MSE: 36 unit cells on 6 × 6 (width × length) cells configuration

2. The manuscript only compares the porosity, average contact perimeter, capillary rise height and evaporation performance of different unit cell types, revealing a higher correlation between water transport capacity and average contact perimeter. However, no tests on water transport capacity and evaporation performance of the same unit cell type with different dimensional scales are conducted. The authors are suggested to select a typical unit cell (e.g., FBCC-+), design 3-4 groups of gradient dimensions, test its capillary rise kinetics, maximum capillary rise height, evaporation rate and other indicators, analyze the regulation law of unit cell dimensions on water transport and evaporation performance, and discuss its influence on salt ion migration capacity.

Reply: We thank the reviewer for this insightful suggestion regarding the dimensional scaling effects within a single unit-cell topology.

We agree that evaluating water transport and evaporation performance for the same unit cell with different dimensions is important to further validate the geometry–performance relationship. Accordingly, additional parametric experiments were conducted using the FBCC-+ topology as a representative structure, as it exhibited both highest capillary rise and evaporation rate. In these experiments, the frame thickness (F) and strut diameter (D) were systematically varied while maintaining a constant unit-cell edge length of 2 mm.

The capillary rise kinetics and equilibrium capillary height were measured for multiple FBCC-+ configurations. The results show that increasing F and D leads to a larger liquid–solid contact perimeter and reduced porosity, which enhances capillary adhesion and promotes faster water transport. Among the tested configurations, the FBCC-+ structure with $F = D = 400$ μm exhibited the fastest rise kinetics and highest equilibrium capillary height, whereas smaller-dimension structures showed reduced transport capability. This trend is consistent with the behavior observed across different unit-cell geometries, confirming that geometry-dependent variations in wetted perimeter and pore volume govern capillary transport.

The evaporation performance of the same FBCC-+ configurations was also evaluated. The FBCC-+ structure with $F = D = 400$ μm exhibited the highest evaporation rate (2.16 ± 0.06 kg m^{-2} h^{-1}), while the configuration with $F = D = 200$ μm showed a lower evaporation rate (1.93 ± 0.16 kg m^{-2} h^{-1}). These variations follow the change in effective thermal transport area, which scales with pore perimeter and frame thickness, confirming that dimensional scaling within a single topology directly regulates heat delivery and evaporation performance. These results demonstrate that both water transport capacity and evaporation performance can be predictably tuned by adjusting the unit-cell dimensions within the same architecture.

To clarify these findings, the dimensional parametric results using FBCC-+ unit cells have been organized and discussed together with the original comparisons across different unit-cell types. This combined analysis establishes a consistent scaling relationship between unit-cell dimensions, capillary transport capability, and evaporation performance. Furthermore, enhanced capillary transport associated with larger contact perimeter is expected to facilitate continuous brine replenishment and improve salt ion migration away from the evaporation interface, thereby reducing salt accumulation and maintaining stable evaporation performance under saline conditions.

These additional experiments and discussions have been incorporated into the revised manuscript and supplementary information.

On the revised manuscript; results

<Addition; text>

[Line 239-247] To further isolate geometric influences by different unit cell types, water transport was additionally examined within a single topology using the FBCC-+ unit cell as a representative structure. The frame thickness (F) and strut diameter (D) were varied simultaneously while maintaining a constant unit cell edge length of 2 mm. Among the tested configurations, the FBCC-+ structure with $F = D = 400$ μm exhibited the fastest capillary rise, consistent with its larger liquid–solid contact perimeter and lower porosity (Figure S8 and Table S2). This behavior follows the same trend observed across different unit-cell geometries, confirming that enhanced adhesion forces associated with increased wetted perimeter, together with reduced liquid gravitational resistance from lower porosity, collectively govern water transport performance[5].

<Addition; text>

[Line 313-322] To further isolate geometric effects, evaporation performance was examined within a single topology using FBCC-+ unit cells with systematically varied frame thickness (F) and strut diameter (D), while maintaining a constant unit-cell edge length of 2 mm. The FBCC-+ structure with $F = D = 400$ μm exhibited the highest evaporation rate (2.16 ± 0.06 kg m^{-2} h^{-1}), whereas the structure with $F = D = 200$ μm exhibited the lowest value (1.93 ± 0.16 kg m^{-2} h^{-1}) (Figure S16). This result follows the variation in effective thermal transport area (Figure S16 and Table S6) and it is consistent with the behavior observed across different unit-cell geometries. Collectively, these results confirm that the effective thermal transport area serves as a governing parameter for heat delivery and dominates the evaporation performance.

On the revised supplementary materials

<Addition; Figure S8>

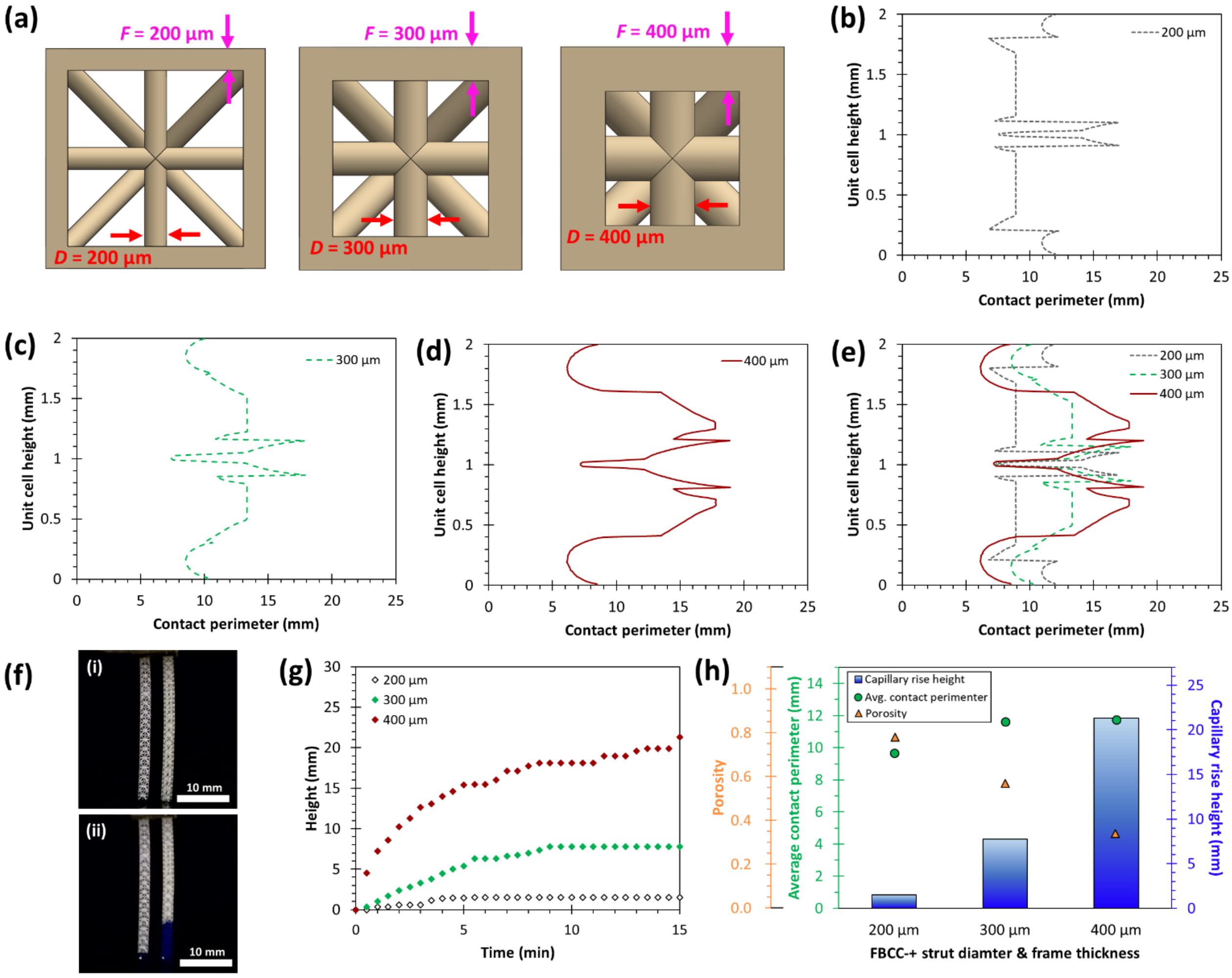

Figure S8. Capillary transport analysis FBCC-+ unit cells with varying frame thickness (*F*) and strut diameter (*D*), with constant unit-cell edge length of 2 mm. (a) Geometrical characteristics of the FBCC-+ structures. (b–d) Contact perimeter as a function of height within FBCC-+ unit cells with F and D of 200, 300, and 400 µm, respectively; (e) comparison of the three cases. (f) Time-dependent capillary rise images of 200 and 300 µm FBCC-+ cells at (i) initial state and (ii) after 15 min. (g) Corresponding time-dependent capillary rise behavior. (h) Comparative summary of porosity, average contact perimeter, and maximum capillary rise for the different unit-cell geometries.

<Addition; Table S2>

Table S2. Geometric parameters and measured capillary rise of FBCC -+ unit cells with different frame thicknesses and strut diameters at a fixed unit-cell edge length of 2 mm.

Frame thickness (μm)	Strut diameter (μm)	Porosity*	Avg. contact perimeter** (mm)	Capillary rise height*** (mm)
200	200	0.78	9.67	1.52
300	300	0.57	11.62	7.76
400	400	0.34	11.75	21.31

*Porosity $= 1 - \frac{Solid\ \ Volume}{Total\ \ volume}$

**Average liquid–solid contact perimeter of single unit cell (normal to capillary direction)

***After 15 minutes

<Addition; Table S6>

Table S6. Geometric parameters and evaporation rate of FBCC-+ unit cells with different frame thicknesse and strut diameters at a fixed unit-cell edge length of 2 mm.

Frame thickness (μm)	Strut diameter (μm)	Effective thermal transport area* in a single unit cell (mm^2)	Evaporation rate ($kg\ m^{-2}\ h^{-1}$)
200	200	2.26	1.93
300	300	2.72	2.03
400	400	2.77	2.16

*Calculated as $A_{eff} \approx P_{pore} \cdot H$; P_{pore} : pore perimeter, H : frame thickness

<Addition; Figure S16>

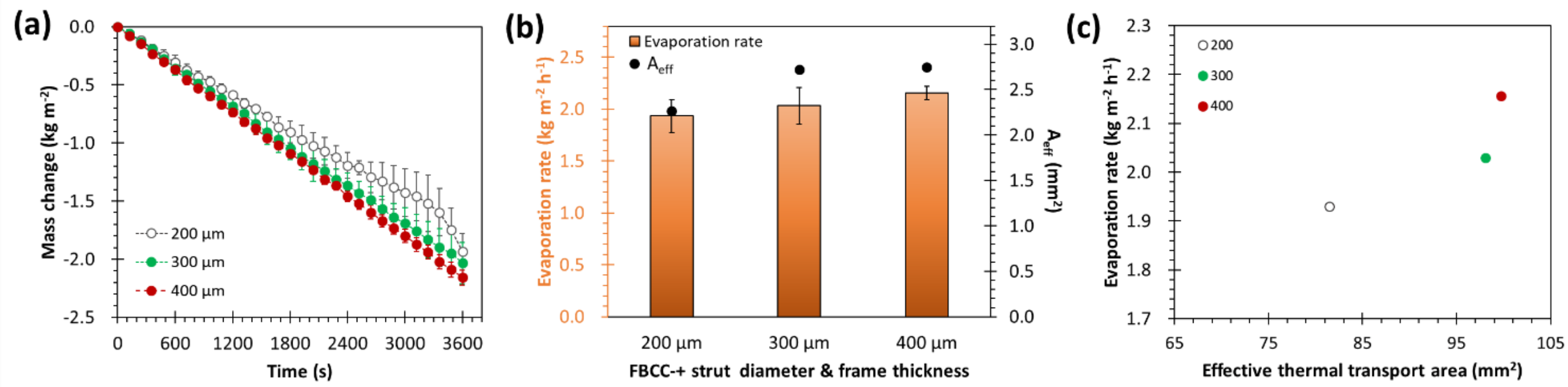

Figure S16. Evaporation performance analysis of FBCC-+ unit cells with varying frame thickness (F) and strut diameter (D), with constant unit-cell edge length of 2 mm on evaporation rate. (a) Mass change during evaporation, (b) Correlation between evaporation rate and effective thermal transfer area (A_{eff}) of a single unit cell, and (c) Relationship between evaporation rate and the total effective thermal transport area.

3. The manuscript focuses on the effect of evaporator height on evaporation rate and emphasizes the contribution of lateral surface evaporation, but fails to provide the specific top surface dimensions (length, width/diameter) of all experimental evaporators, making it impossible to calculate the lateral/top surface area ratio and lack a basis for the quantitative analysis of lateral surface evaporation contribution. The authors are required to supplement the top surface dimensions of the evaporators, and quantitatively analyze the evaporation contribution ratio of lateral and top surfaces in combination with the lateral surface area at different heights.

Reply: We thank the reviewer for this important comment.

We agree that providing the top surface dimensions and quantifying the relative contributions of lateral and top surface evaporation are necessary for a more rigorous analysis of height-dependent evaporation behavior.

In the revised manuscript, we have explicitly provided the geometric dimensions of the multicellular solar evaporators (MSEs). The MSEs were constructed by overlapping adjacent unit-cell frames to form a continuous top surface consisting of a 6 × 6 cells array. To clarify this configuration, we added a schematic illustration defining the geometric parameters of the MSE, including length (*L*), width (*W*), and height (*H*), along with the overlapping regions between adjacent unit cells (Figure S10). Based on this geometry, the top surface dimensions of all experimental evaporators are now explicitly reported.

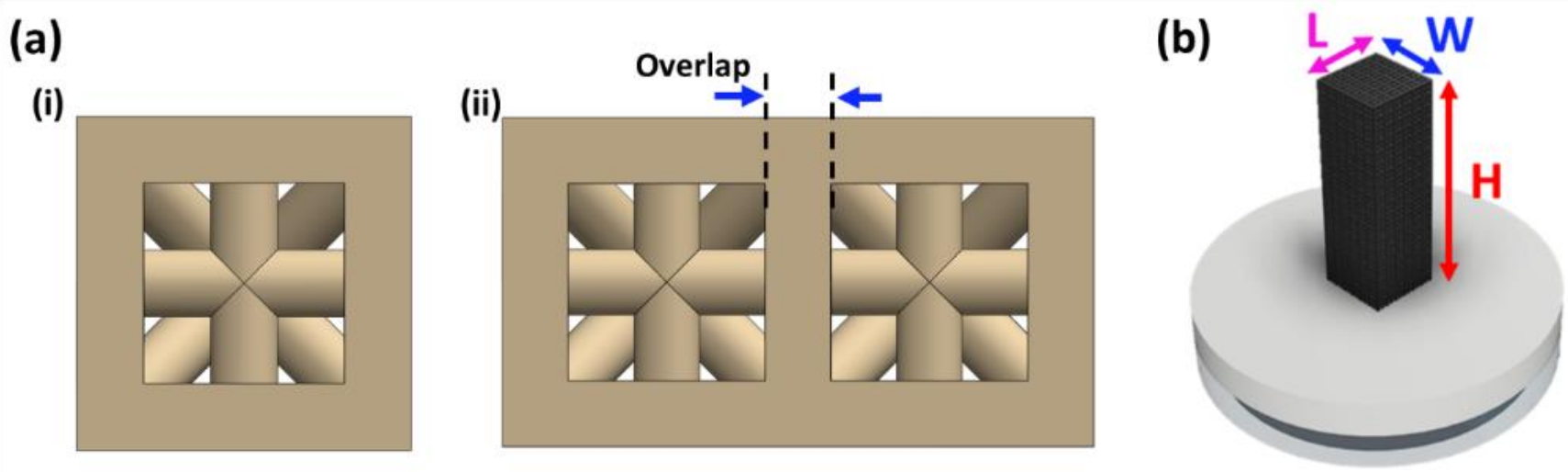

Figure S10. Schematic illustration of the geometrical characteristics of the MSE. (a) Front view of (i) single-cell and (ii) multicellular configurations, highlighting the overlapping region corresponding to the frame thickness in the multicellular structure. (b) Definition of geometric parameters of the MSE, including length (L), width (W), and height (H).

In addition, we have included tables summarizing the geometric parameters of the MSEs at 0 cm exposed height and for FBCC-+ structures at different exposed heights. These parameters enable direct calculation of the lateral surface area, top surface area, and their corresponding ratios as a function of height. Using these values, we performed a quantitative analysis of the relative contributions of lateral and top surface evaporation. The analysis shows that increasing the exposed height substantially increases the available lateral evaporation area, leading to a progressively larger contribution from lateral surfaces, which

explains the observed height-dependent enhancement in evaporation rate. These results provide a quantitative basis for understanding the height-dependent enhancement in evaporation rate and clarify the dominant role of lateral surface evaporation at increased heights.

On the supplementary materials, the schematic illustration and the corresponding geometric parameters and contribution analysis have been added and accordingly discussed in the revised manuscript to improve clarity.

On the revised manuscript; results

<From>

[Line 222-223] The solar evaporation performance of the MSEs was evaluated under 1 sun irradiation using the setup shown in Figure 4A.

<To>

[Line 253-255] The solar evaporation performance of the MSEs was evaluated under 1 sun irradiation using the setup shown in Figure 4A. The MSEs were constructed by overlapping adjacent unit-cell frames to form a continuous top surface consisting of a 6 × 6 array (Figure S9 and Table S3).

<From>

[Line 261-265] To evaluate the effect of exposed geometry, a series of FBCC-+ MSEs with increasing height were fabricated (Figure S7). Increasing the height introduced additional lateral surface area, enabling supplementary evaporation pathways beyond the top surface. Consequently, the evaporation rate increased from 2.16 ± 0.06 kg m^{-2} h^{-1} at 0 cm height to 6.90 ± 0.07 kg m^{-2} h^{-1} for the 5 cm configuration, denoted as FBCC-+_5 (Figure 4D).

<To>

[Line 322-328] To evaluate the effect of exposed geometry, a series of FBCC-+ MSEs with increasing height were fabricated (Figure S17 and Table S7). Increasing the height introduced additional lateral surface area, enabling supplementary evaporation pathways beyond the top surface. Accordingly, the evaporation rate increased from 2.16 ± 0.06 kg m^{-2} h^{-1} at 0 cm height to 6.90 ± 0.07 kg m^{-2} h^{-1} for the 5 cm configuration, denoted as FBCC-+_5 (Figure 4D). The contribution of the lateral surface was quantified (Table S8), showing that the lateral evaporation area increases monotonically with height and accounts for 68.70% of the total evaporation for the FBCC-+_5.

<From>

[Line 348-350] Based on these results, a cup-shaped hybrid configuration was developed to amplify crystallization localization by confining ion migration while preserving open pathways for vapor escape (Figure 5D).

<To>

[Line 427-429] Based on these results, a cup-shaped hybrid configuration was developed to amplify crystallization localization by spatially biasing ion redistribution while preserving open pathways for vapor escape (Figure 5D and Table S10).

On the revised supplementary materials

<Addition; Figure S10>

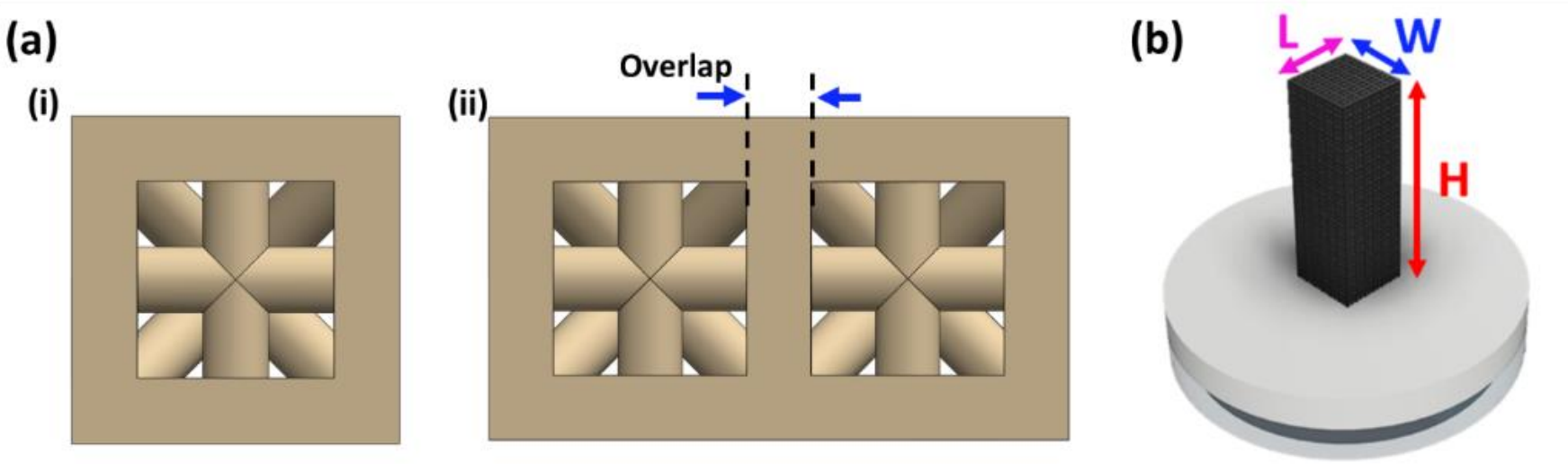

Figure S10. Schematic illustration of the geometrical characteristics of the MSE. (a) Front view of (i) single-cell and (ii) multicellular configurations, highlighting the overlapping region corresponding to the frame thickness in the multicellular structure. (b) Definition of geometric parameters of the MSE, including length (L), width (W), and height (H).

<Addition; Table S3>

Table S3. Geometric parameters of MSEs at 0 cm exposed height.

Unit cell	Dimensions* ($W \times L$, cells)	Dimensions* ($W \times L$, cm)	Light illuminated area (cm^2)
BCC	6 × 6	1 × 1	1
Isotruss	6 × 6	1 × 1	1
FCC-X	6 × 6	1 × 1	1
FCC-+	6 × 6	1 × 1	1

*H = 0 for 0 cm exposed height case

<Addition; Table S7>

Table S7. Geometric parameters of FBCC-+ MSEs at different exposed height.

MSE configuration	Dimensions* ($W \times L \times H$, cells)	Dimensions* ($W \times L \times H$, cm)	Light illuminated area (cm^2)
FBCC-+_0	6 × 6 × 0	1 × 1 × 0	1
FBCC-+_1	6 × 6 × 6	1 × 1 × 1	1
FBCC-+_2	6 × 6 × 12	1 × 1 × 2	1
FBCC-+_3	6 × 6 × 18	1 × 1 × 3	1
FBCC-+_4	6 × 6 × 24	1 × 1 × 4	1
FBCC-+_5	6 × 6 × 30	1 × 1 × 5	1

*Exposed height refers to the portion of the structure extending above the water reservoir.

<Addition; Table S8>

Table S8. Lateral surface contribution to evaporation performance of MSEs.

MSE configuration	Top surface area (cm^2)	Lateral surface area (cm^2)	ER_{total}* ($kg\ m^{-2}\ h^{-1}$)	$ER_{lateral}$** ($kg\ m^{-2}\ h^{-1}$)	Lateral contribution*** (%)
FBCC-+_0	1	0	2.16	-	-
FBCC-+_1	1	4	3.69	1.53	41.46
FBCC-+_2	1	8	4.80	2.64	55.00
FBCC-+_3	1	12	5.59	3.43	61.36
FBCC-+_4	1	16	6.46	2.40	66.56
FBCC-+_5	1	20	6.90	4.74	68.70

*Total evaporation rate.

**Evaporation rate contributed by lateral surfaces, calculated as $ER_{lateral} = ER_{total} - ER_{0cm}$

***Lateral contribution $= \frac{ER_{lateral}}{ER_{total}} \times 100$

<Addition; Table S10>

Table S10. Geometric parameters of cup-shaped hybrid evaporator.

Dimensions* ($W \times L \times H$, cells)	Dimensions* ($W \times L \times H$, cm)	Light illuminated area (cm^2)
8 × 8 × 12	1.32 × 1.32 × 2	1.74

*Exposed height refers to the portion of the structure extending above the water reservoir.

4. The manuscript proposes that in the hybrid multicellular structure, salt ions preferentially crystallize in the BCC region with low evaporation rate, attributing this phenomenon to ion diffusion induced by evaporation rate gradients. However, Figure 3D clearly shows that the capillary rise capacity of the BCC unit cell is significantly weaker than that of FBCC-+, which is mainly due to the combined characteristics of porosity, pore size and contact perimeter of the BCC unit cell leading to poor water transport capacity and limited salt ion migration. Furthermore, under sufficient water supply, a lower evaporation rate should result in less salt accumulation rather than more. The direct correlation between salt ion accumulation and low evaporation rate lacks physical rationality.

Reply: We appreciate the reviewer's thoughtful comment regarding the physical rationale of preferential salt crystallization in the low-evaporation BCC region.

We agree that, when considered in isolation, a unit cell with weaker capillary rise and lower evaporation would generally be expected to accumulate less salt. However, in our hybrid multicellular structure, salt localization is governed by interconnected-domain ionic redistribution, rather than by the intrinsic capillary rise capacity of an isolated unit cell.

Importantly, all evaporation experiments were conducted after fully wetting the samples in the corresponding saline solution prior to light irradiation. Therefore, the structures were already completely wetted at the start of the experiment, and salt transport during evaporation occurred within a continuous liquid phase. Under these conditions, the capillary rise height measured in Figure 3D does not limit ion migration between the FBCC-+ and BCC domains. Instead, ions can redistribute through the hydraulically connected liquid network during evaporation.

Although the BCC unit cell exhibits weaker capillary rise compared to FBCC-+, the liquid phases of the two regions remain connected in the hybrid structure. The high-evaporation FBCC-+ domain continuously enriches the local salt concentration, thereby establishing a salinity gradient across the connected liquid phase. This gradient drives diffusive ion transport toward adjacent lower-flux regions. Because the BCC domain removes water more slowly, ions transported from the FBCC-+ region are cleared less efficiently, leading to progressive accumulation and earlier supersaturation in the BCC region. Consequently, the low-evaporation BCC domain functions as a terminal accumulation zone despite its weaker intrinsic capillary rise.

This type of gradient-driven salt localization has also been reported in directional crystallization systems. For example, Liu et al.[15] demonstrated that evaporation-induced

salinity gradients within a connected evaporation structure can drive ion redistribution toward designated crystallization regions. Similarly, Ding et al.(*Sci. Bull.*)[13] reported preferential crystallization in regions with reduced evaporation flux, where limited ion clearance promotes earlier supersaturation even when water transport is weaker. These studies indicate that salt localization can arise from inter-region concentration gradients, rather than from local evaporation rate or capillary rise alone.

Furthermore, our numerical simulations (Figure 5E) directly show a directional ionic flux from the FBCC-+ region toward the BCC region, confirming that salt accumulation is governed by diffusion driven by evaporation-induced concentration gradients. In this context, the weaker capillary transport in BCC does not prevent ion migration; instead, it reduces dilution and promotes earlier supersaturation once ions arrive from the high-flux region.

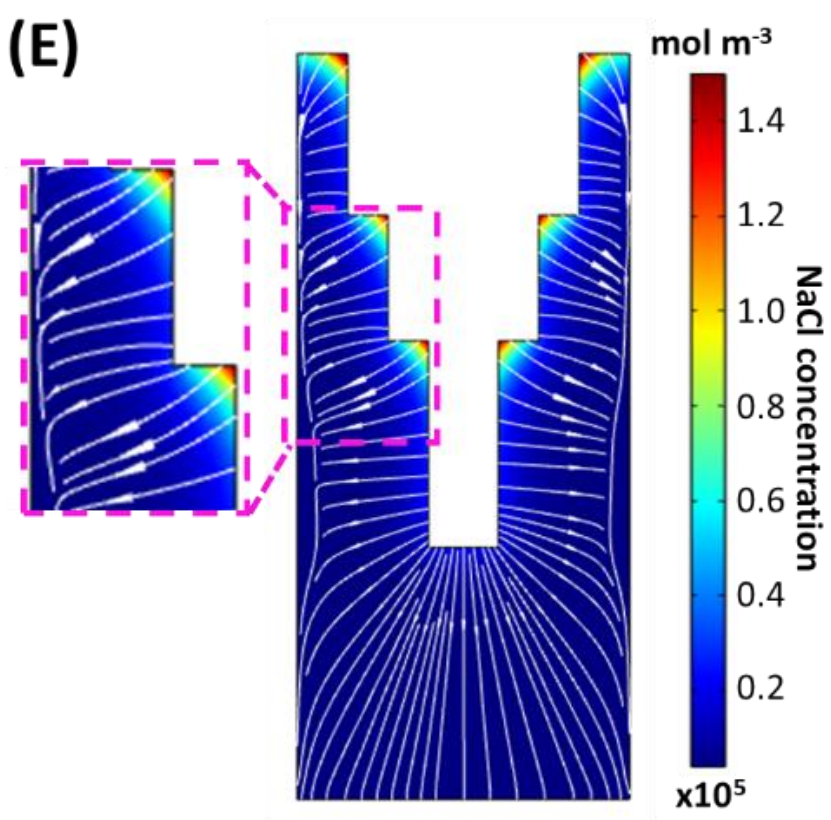

Figure 5. (E) Numerical simulation of ion concentration distribution and transport flux in the hybrid cup-shaped structure.

After crystallization initiates in the BCC domain, the deposited salt forms a hydrated porous structure that maintains liquid transport and enables continued ion supply. Similar transport-active porous salt layers have been reported to sustain evaporation and function as auxiliary evaporation media rather than blocking layers (Yu et al.[12]; Ding et al.(*Adv. Funct. Mater.*)[16]). This behavior further stabilizes salt localization in the designated BCC region during prolonged operation.

These clarifications have been incorporated into the revised manuscript to emphasize that salt localization in the hybrid multicellular evaporator arises from evaporation-gradient-driven ionic redistribution within a fully wetted and connected liquid network, rather than from differences in capillary rise capacity of individual unit cells.

On the revised manuscript; results

<From>

[Line 333-335] While the high-evaporation FBCC-+ region experiences stronger local salt enrichment, it simultaneously generates a concentration gradient that drives diffusive ion transport toward the lower-evaporation BCC region[17].

<To>

[Line 410-414] Although the high-evaporation FBCC-+ region initially experiences stronger local salt enrichment, the resulting salinity gradient continuously redistributes ions toward the adjacent lower-flux BCC region through diffusive transport. The BCC domain, where water removal is slower and ion clearance is limited, therefore acts as a terminal accumulation zone in which supersaturation is reached preferentially[17].

<From>

[Line 350-341] In this architecture, high-evaporation FBCC-+ unit cells were positioned on the inner surface, whereas low-evaporation BCC unit cells formed the outer region.

<To>

[Line 429-433] In this architecture, high-evaporation FBCC-+ unit cells were positioned on the top and inner surface, whereas low-evaporation BCC unit cells formed the outer region. This arrangement shortens the redistribution pathway between the evaporation and crystallization domains and promotes outward ion accumulation within a predefined peripheral zone.

5. Figure S9 shows that the evaporation efficiency is significantly higher than 100% when the evaporator height is 0 cm (without lateral evaporation contribution), and the manuscript does not provide a reasonable interpretation for this phenomenon.

Reply: Thank you for this important comment regarding the apparent evaporation efficiency exceeding 100% at 0 cm height in Figure S9 (Figure S20 in the revised manuscript).

We agree that the apparent energy utilization efficiency exceeding 100% for the 0 cm geometry requires clarification, particularly because lateral evaporation is absent in this case. We emphasize that the reported values correspond to apparent energy utilization efficiency, and the elevated value for the 0 cm configuration arises from coupled heat–mass transfer effects rather than from lateral surface contributions.

The apparent energy utilization efficiency (η) was calculated using the conventional latent-heat-based definition widely adopted in solar interfacial evaporation studies[18-21]. First, the net

evaporation rate under illumination was determined by subtracting the dark evaporation component measured under identical conditions:

$$\dot{m}_{net} = \dot{m}_{light} - \dot{m}_{dark}$$

The energy utilization efficiency was then calculated as:

$$\eta = \frac{\dot{m}_{net}(h_{lv} + C_p \Delta T)}{P_{solar}}$$

where $\dot{m}_{net}$ is the net mass evaporation rate (kg m^{-2} s^{-1}), h_{lv} is the latent heat of vaporization of water (2.26 MJ kg^{-1}), C_p is the specific heat capacity of water (4.18 kJ kg^{-1} K^{-1}), ΔT and P_{solar} is the incident solar power density (1 kW m^{-2}). This formulation represents a baseline-normalized, apparent efficiency commonly used for comparing solar evaporators. Values exceeding unity indicate that part of the latent heat required for evaporation is supplied by environmental heat exchange, rather than implying that more solar energy is converted than incident.

For the 0 cm geometry, the measured surface temperature under illumination was approximately 35 °C, which is significantly higher than ambient. This temperature increase enhances the saturated vapor concentration at the interface and strengthens the evaporation driving force. At the same time, the projected evaporation area in our experiments is relatively small (1 cm^2), which promotes efficient vapor removal from the entire surface. Under such conditions, the vapor boundary layer above the evaporating interface is continuously refreshed, suppressing local humidity accumulation and enabling a higher area-normalized evaporation flux[22,23].

Recent studies have shown that evaporation performance is strongly influenced by vapor removal and local convection rather than by heat input alone. For example, Gao et al. demonstrated that increasing the planar evaporator size leads to the formation of central evaporation dead zones, whereas smaller evaporation surfaces maintain more effective vapor removal and exhibit unusually high area-normalized evaporation rates[22]. Similarly, Li et al. reported that modifying the evaporator geometry to enhance thermal convection improves evaporation performance by reducing local humidity accumulation above the interface[23]. In addition, Wang et al. showed that self-generated convective flow enhances evaporation by facilitating vapor removal from the evaporation surface, highlighting the importance of mass-transfer-controlled evaporation behavior[24].

These studies indicate that even in nominally planar configurations, efficient vapor removal and boundary-layer renewal can significantly increase the measured evaporation rate. In our

case, the combination of elevated surface temperature (~35 °C) and small evaporation area (1 cm^2) enables efficient mass transfer, resulting in an apparent efficiency exceeding 100% even without lateral evaporation. Importantly, this does not imply that the system converts more solar energy than is incident. Rather, part of the latent heat required for evaporation is supplied by environmental heat exchange, which becomes significant when the vapor boundary layer is continuously refreshed. This interpretation is consistent with the widely reported apparent efficiencies exceeding unity in interfacial solar evaporation systems[25].

To clarify this point, we have included a discussion on the supplementary materials to explicitly state that the >100% apparent efficiency observed at 0 cm height originates from enhanced interfacial mass transfer and environmental heat contribution under a small heated evaporation surface, rather than from lateral evaporation.

On the revised supplementary materials

<Addition; text for Figure S20>

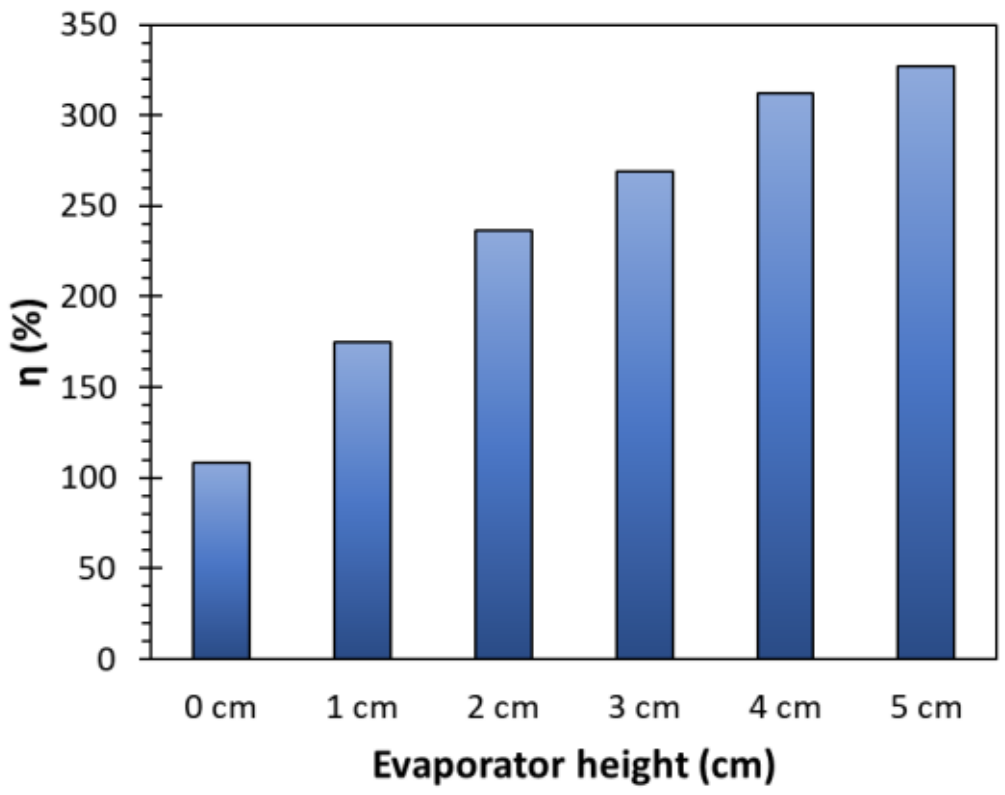

Figure S20. Apparent energy utilization efficiency of FBCC-+ evaporators with different heights.

Notably, the apparent energy utilization efficiency for the 0 cm configuration exceeds unity even in the absence of lateral evaporation. This behavior arises from the elevated interfacial temperature (~35 °C) combined with the small projected evaporation area (1 cm^2), which promotes efficient vapor removal from the entire surface. Under these conditions, the vapor boundary layer is continuously refreshed, suppressing local humidity buildup and enhancing the evaporation flux. Similar behavior has been reported in small-area planar evaporators, where efficient boundary-layer renewal prevents the formation of evaporation dead zones and yields unusually high area-normalized evaporation rates[22,23]. This mass-transfer enhancement allows additional environmental heat uptake even in nominally planar configurations, resulting in apparent efficiencies exceeding 100%.

6. Figure S15b shows that lateral surface evaporation has almost no contribution to evaporation rate, while Figure 4D clearly reveals that lateral evaporation surfaces at different heights have a significant positive contribution to evaporation rate. The contradictory results seriously affect the credibility of the core conclusion of lateral surface evaporation contribution. The authors are requested to supplement targeted verification experiments,

Reply: We appreciate your constructive comment regarding the apparent inconsistency between Figure S15b (on the original manuscript) and Figure 4D. We agree that clarifying this point is essential to avoid misunderstanding of the contribution of lateral surface evaporation.

In the original manuscript, Figure S15b corresponds to experiments performed on the cup-shaped hybrid evaporator, where the outer lateral surface was selectively sealed to isolate the evaporation occurring from different regions.

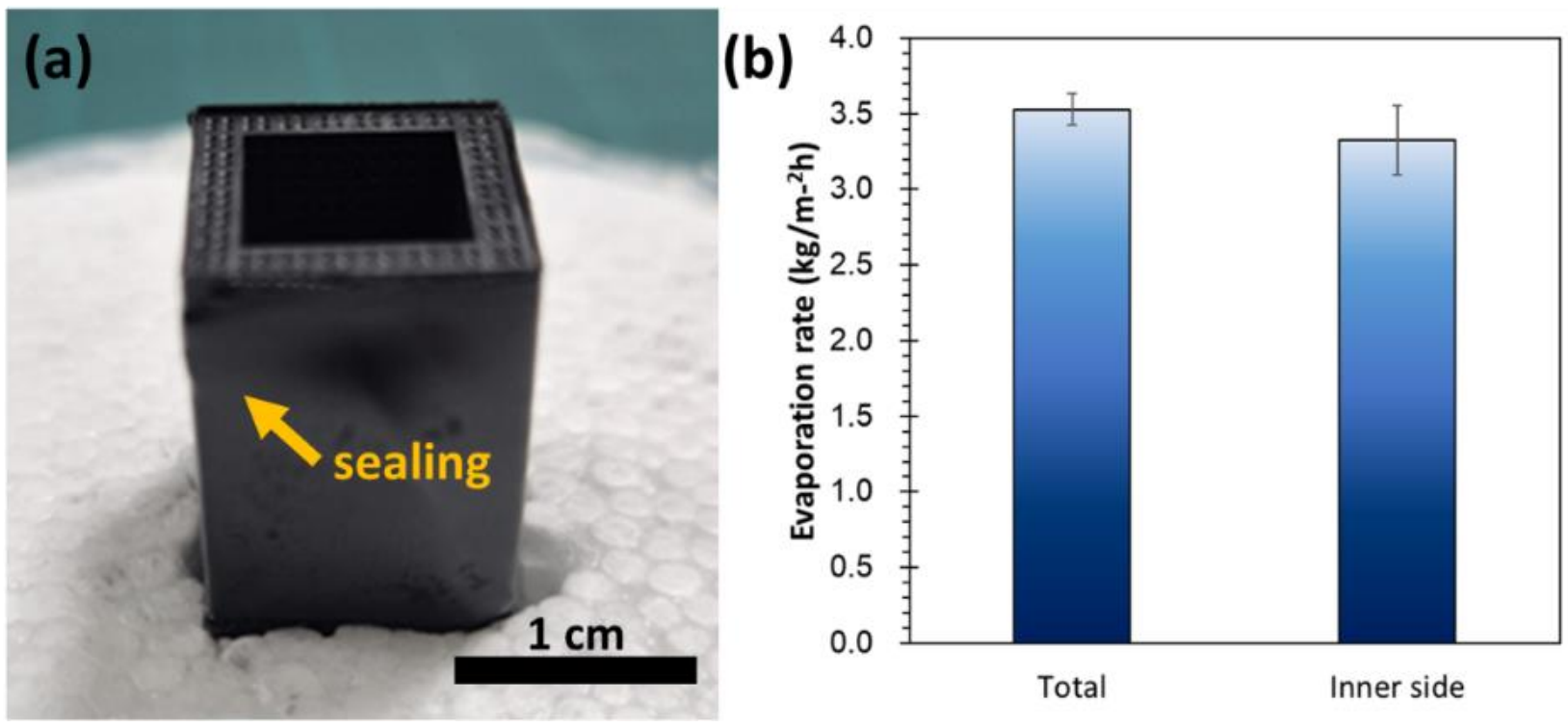

Figure S15. Evaporation performance of the hybrid cup-shape evaporator. (a) Optical images of the experimental setup used to isolate and measure evaporation occurring exclusively from the inner evaporation surface. (b) Comparison of evaporation rates under total (uncovered) and inner-surface-only evaporation conditions (outer lateral surface sealed). The measured evaporation rates were used as input parameters for the numerical simulations. (Original manuscript)

These experiments were designed to quantify the evaporation rates of the outer and inner regions separately, which were subsequently used as input parameters for numerical simulations to analyze the localized salt crystallization mechanism arising from spatial differences in evaporation rate. In this hybrid configuration, the outer region was constructed using BCC unit cells, which exhibit the lowest evaporation rate, while the top and inner regions consisted of FBCC-+ cells with higher evaporation performance.

The apparent contradiction arises from the interpretation of the sealing condition. In Figure S15a, only the outer lateral surfaces were sealed, whereas the top surface and the inner lateral surfaces remained exposed and actively contributed to evaporation. Because the cup-shaped hybrid evaporator contains internal lateral surfaces, the measured evaporation rate

in the “sealed” case still includes contributions from both the top and inner lateral regions. Therefore, the slight reduction in evaporation rate observed in Figure S15b reflects only the removal of the outer lateral contribution, rather than indicating negligible lateral evaporation overall. This is fundamentally different from the height-dependent structures in Figure 4D, where the entire lateral surface area increases with exposed height and contributes significantly to evaporation.

To clarify this point, we have revised the Supplementary Information by adding a schematic illustration of the cup-shaped hybrid evaporator, explicitly indicating the outer lateral region, top and inner lateral surface region. We have also clarified the nomenclature and in the revised manuscript to avoid ambiguity.

On the revised manuscript; results

<From>

[Line 350-351] In this architecture, high-evaporation FBCC-+ unit cells were positioned on the inner surface, whereas low-evaporation BCC unit cells formed the outer region.

<To>

[Line 438-440] In this architecture, high-evaporation FBCC-+ unit cells were positioned on the top and inner surface, whereas low-evaporation BCC unit cells formed the outer region.

On the revised supplementary materials

<From>

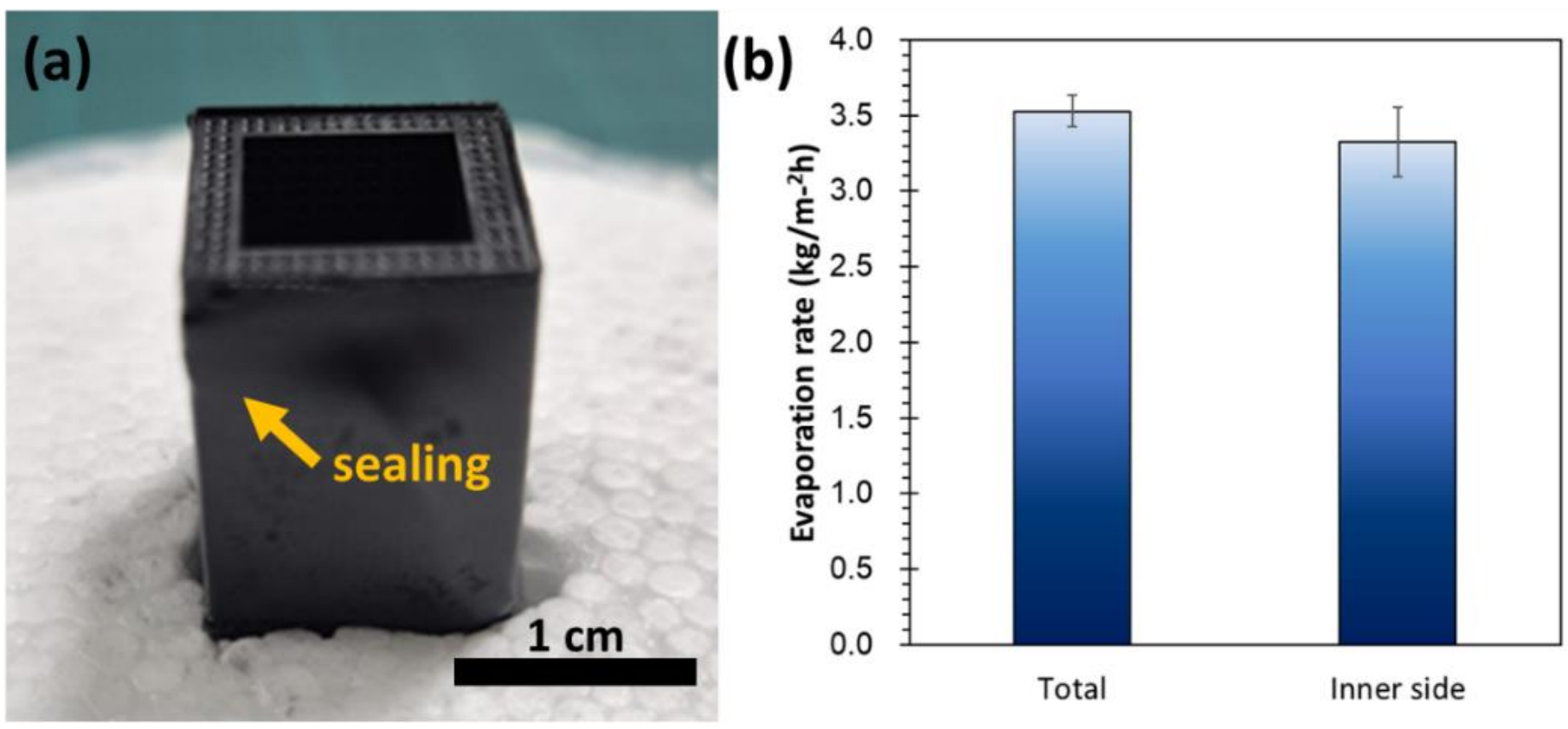

Figure S15. Evaporation performance of the hybrid cup-shape evaporator. (a) Optical images of the experimental setup used to isolate and measure evaporation occurring exclusively from the inner evaporation surface. (b) Comparison of evaporation rates under total (uncovered) and inner-surface-only evaporation conditions (outer lateral surface sealed). The measured evaporation rates were used as input parameters for the numerical simulations.

<To>

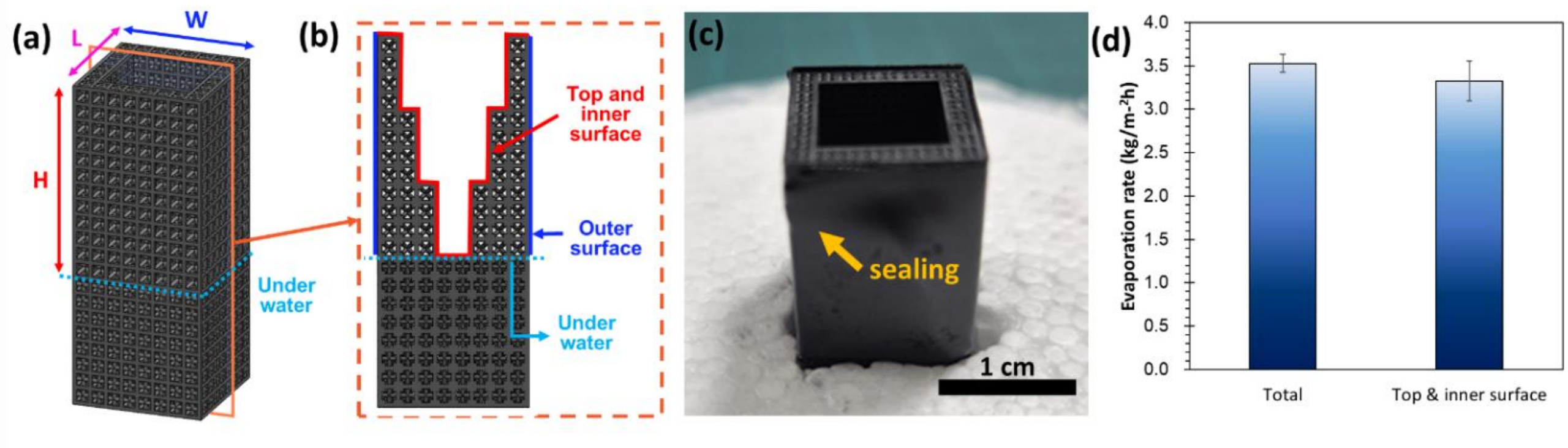

Figure S27. Geometry and evaporation analysis of the cup-shaped hybrid evaporator. (a) Schematic illustration of the geometrical configuration. (b) Cross-sectional view highlighting the outer region (BCC) and the top and internal surfaces (FBCC-+). (c) Optical images of the experimental setup used to isolate evaporation occurring exclusively from the internal surfaces. (d) Comparison of evaporation rates under total (uncovered) and top and inner surface-only evaporation conditions (outer surface sealed). The measured evaporation rates were used as input parameters for the numerical simulations.

<From>

[Mehod S2] Inner fast surface: $m_{evap} = 9.25 \times 10^{-4}$ kg m^{-2} s^{-1} (Inner side evaporation rate, Figure S12)

<To>

[Mehod S2] Top and inner surface: $m_{evap} = 9.25 \times 10^{-4}$ kg m^{-2} s^{-1} (Top and inner ER, Figure S27)

<From>

[Mehod S2] Outer slow surface: $m_{evap} = 5.56 \times 10^{-5}$ kg m^{-2} s^{-1} (Total – inner side evaporation rate, Figure S12)

<To>

[Mehod S2] Outer surface: $m_{evap} = 5.56 \times 10^{-5}$ kg m^{-2} s^{-1} (Total – Top and inner ER, Figure S27)

Reviewer #3: The manuscript presents an interesting study on lattice-structured evaporators and their role in salt management and water purification. The work is generally well organized and the concept of geometry-enabled performance enhancement is appealing and interesting. However, several points would benefit from further clarification or discussion to strengthen the mechanistic interpretation and the practical implications of the design. The following comments are offered for the authors' consideration.

1. Although seven unit-cell architectures were analyzed in the capillary transport study, evaporation performance was evaluated for only four representative designs. Since the manuscript notes that FBCC structures share similar top-surface geometries with their FCC counterparts, it would be helpful to briefly verify whether the omitted architectures exhibit comparable evaporation behavior under the zero-height condition.

Reply: Thank you for your constructive review of our manuscript. We agree that verifying the evaporation behavior of the omitted architectures under the zero-height condition would further support the rationale for focusing on representative unit cell designs.

In the revised manuscript, we performed additional evaporation measurements at 0 cm exposed height for the FBCC-+ unit cell, which was selected for further optimization and shares an identical top-surface geometry with the corresponding FCC-+ structure. The results confirm that FBCC-+ exhibits evaporation performance comparable to FCC-+ under the same 0 cm condition, consistent with their identical evaporative surface configuration (Table S5). This observation supports our assumption that top-surface evaporation is primarily governed by the exposed pore geometry, while differences in overall performance arise mainly from variations in capillary transport.

We have added this clarification and the corresponding control comparison to the revised manuscript, where the evaporation measurements at 0 cm confirm comparable performance between FCC-+ and FBCC-+, consistent with their identical evaporative surface geometry. This additional verification supports the selection of representative architectures for the evaporation study.

On the revised manuscript; results

<From>

[Line 259-261] Based on the combined analyses of water transport and evaporation performance, the FBCC-+ geometry was selected for further optimization, as it retains the same evaporative surface as FCC-+ while providing superior capillary transport.

<To>

[Line 307-313] Based on the combined analyses of water transport and evaporation performance, the FBCC-+ geometry was selected for further optimization. FBCC-+ preserves the same evaporative surface as FCC-+ while exhibiting superior capillary transport and among the highest evaporation rates, making it an optimal candidate for subsequent parametric investigations (Table S5). Evaporation measurements at 0 cm confirmed comparable performance between FBCC-+ and FCC-+, consistent with their identical evaporative surface geometry (Figure S15).

On the revised supplementary materials

<Addition; Figure S15>

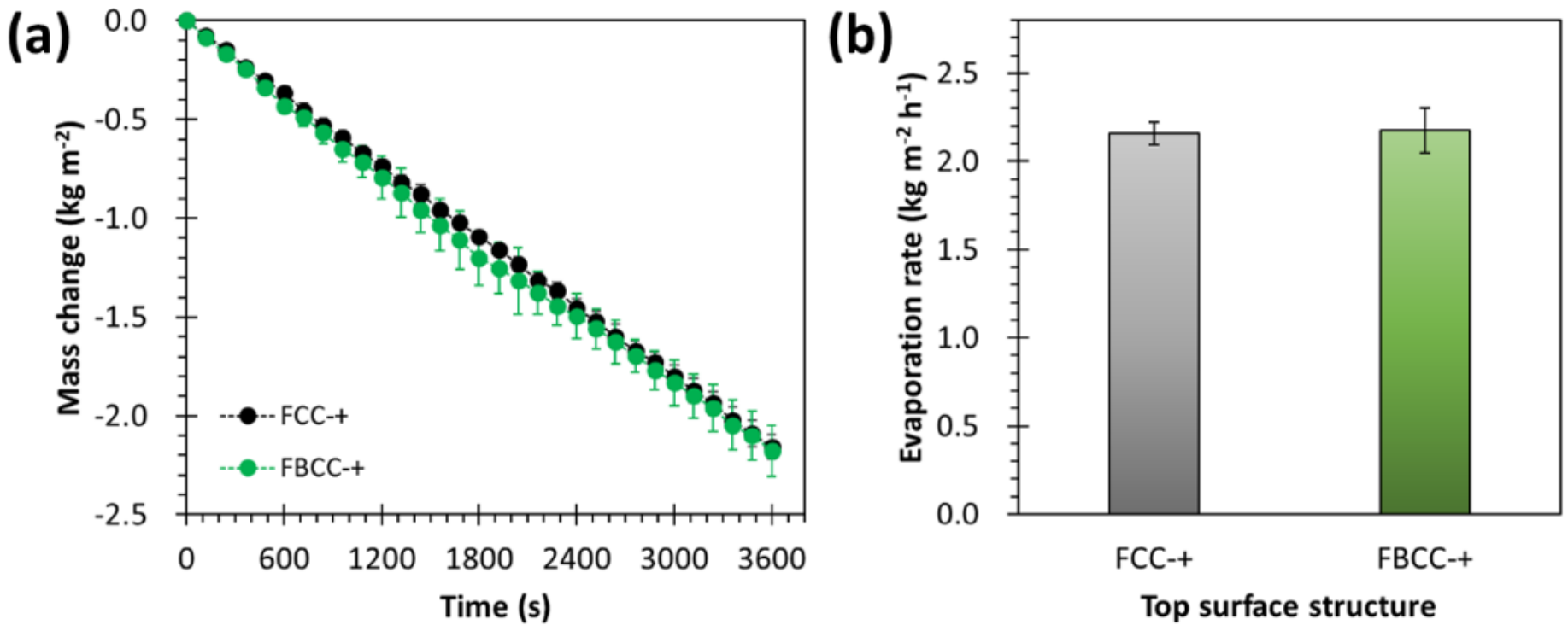

Figure S15. Evaporation performance analysis of FCC-+ and FBCC-+ MSE. (a) Mass change during evaporation, and (b) evaporation rate comparison.

<Addition; Table S5>

Table S5. Effective thermal transport area of different unit cell architectures

Unit cell	Effective thermal transport area* in a single unit cell (mm^2)	Effective thermal transport area in MSE (mm^2)
SC	1.93	69.12
BCC	1.94	69.84
FCC-X	2.72	97.92
FCC-+	2.77	99.72
Isotruss	2.44	87.84
FBCC-X	2.71	97.92
FBCC-+	2.77	99.72

*Calculated as $A_{eff} \approx P_{pore} \cdot H$; P_{pore} : pore perimeter, H : frame thickness

MSE: 36 unit cells on 6 × 6 (width × length) cells configuration

2. The evaporation rate increases from 2.16 to 6.90 kg m⁻² h⁻¹ as the exposed height increases. Clarification of the extent to which this enhancement arises from an increase in effective evaporation area, as opposed to an intrinsic improvement in evaporation efficiency per unit active surface, would strengthen the interpretation of the results.

Reply: We thank the reviewer for this insightful comment. We agree that distinguishing between the increase in effective evaporation area and intrinsic improvement in evaporation efficiency is important for accurately interpreting the height-dependent performance.

To address this point, we performed a quantitative geometric and contribution analysis in the revised manuscript. The multicellular solar evaporators (MSEs) were constructed by overlapping adjacent unit-cell frames to form a continuous top surface consisting of a 6 × 6 array (Figure S10). A schematic illustration defining the geometric parameters of the MSE, including length (L), width (W), and height (H), together with the overlapping regions, has been added (Figure S9). Based on this geometry, the top surface dimensions remain constant with increasing exposed height, while only the lateral surface area increases.

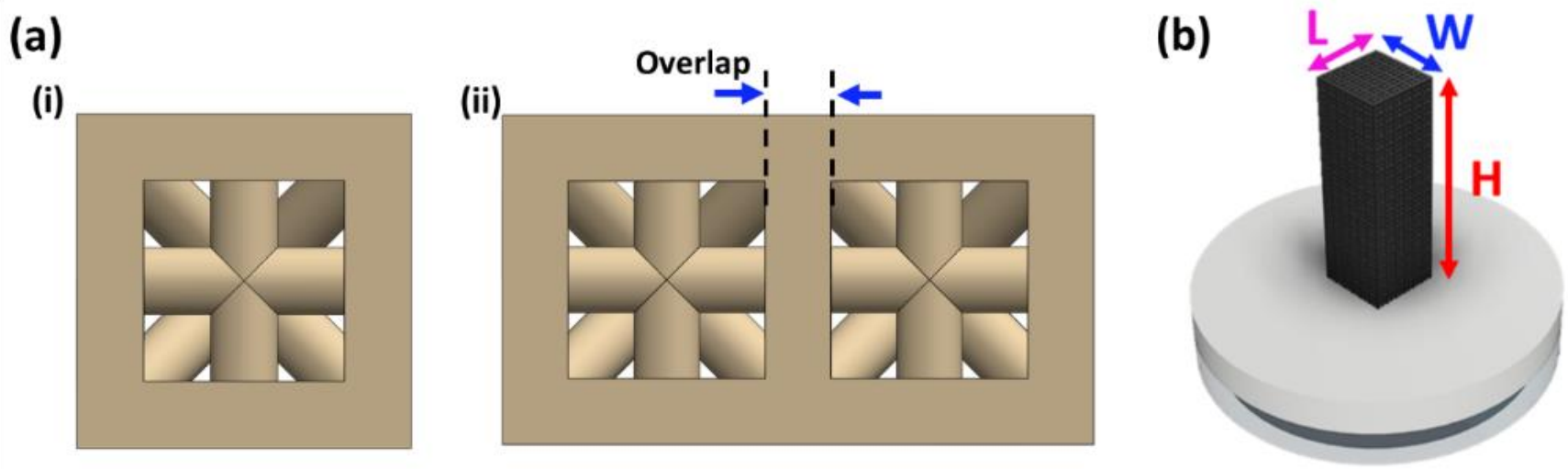

Figure S10. Schematic illustration of the geometrical characteristics of the MSE. (a) Front view of (i) single-cell and (ii) multicellular configurations, highlighting the overlapping region corresponding to the frame thickness in the multicellular structure. (b) Definition of geometric parameters of the MSE, including length (L), width (W), and height (H).

The analysis shows that the increase in evaporation rate from 2.16 to 6.90 kg m^{-2} h^{-1} primarily originates from the increase in effective evaporation area associated with the additional lateral surfaces. The top surface area remains unchanged, and the area-normalized evaporation behavior of the top surface does not exhibit intrinsic enhancement. Instead, the progressively increasing lateral surface area provides additional evaporation pathways and environmental heat harvesting, leading to the observed increase in overall evaporation rate, accounting 68.70% of the total evaporation for the FBCC-+_5

These additions provide a quantitative basis for distinguishing between geometric area effects and intrinsic efficiency changes. The schematic illustration, geometric parameter tables, and lateral contribution analysis have been included in the Supplementary Information

and discussed in the revised manuscript to clarify the mechanism of height-dependent evaporation enhancement.

On the revised manuscript; results

<From>

[Line 261-265] To evaluate the effect of exposed geometry, a series of FBCC-+ MSEs with increasing height were fabricated (Figure S7). Increasing the height introduced additional lateral surface area, enabling supplementary evaporation pathways beyond the top surface. Consequently, the evaporation rate increased from 2.16 ± 0.06 kg m^{-2} h^{-1} at 0 cm height to 6.90 ± 0.07 kg m^{-2} h^{-1} for the 5 cm configuration, denoted as FBCC-+_5 (Figure 4D).

<To>

[Line 322-328] To evaluate the effect of exposed geometry, a series of FBCC-+ MSEs with increasing height were fabricated (Figure S17 and Table S7). Increasing the height introduced additional lateral surface area, enabling supplementary evaporation pathways beyond the top surface. Accordingly, the evaporation rate increased from 2.16 ± 0.06 kg m^{-2} h^{-1} at 0 cm height to 6.90 ± 0.07 kg m^{-2} h^{-1} for the 5 cm configuration, denoted as FBCC-+_5 (Figure 4D). The contribution of the lateral surface was quantified (Table S8), showing that the lateral evaporation area increases monotonically with height and accounts for 68.70% of the total evaporation for the FBCC-+_5.

On the revised supplementary materials

<Addition; Table S8>

Table S8. Lateral surface contribution to evaporation performance of MSEs.

MSE configuration	Top surface area (cm^2)	Lateral surface area (cm^2)	ER_{total}* (kg m^{-2} h^{-1})	$ER_{lateral}$** (kg m^{-2} h^{-1})	Lateral contribution*** (%)
FBCC-+_0	1	0	2.16	-	-
FBCC-+_1	1	4	3.69	1.53	41.46
FBCC-+_2	1	8	4.80	2.64	55.00
FBCC-+_3	1	12	5.59	3.43	61.36
FBCC-+_4	1	16	6.46	2.40	66.56
FBCC-+_5	1	20	6.90	4.74	68.70

*Total evaporation rate.

**Evaporation rate contributed by lateral surfaces, calculated as $ER_{lateral} = ER_{total} - ER_{0cm}$

***Lateral contribution $= \frac{ER_{lateral}}{ER_{total}} \times 100$

3. The experimental device consists of a three-dimensional ordered lattice, whereas the simulation is conducted using a two-dimensional homogenized cross-section. Additional justification of why this reduced model is sufficient to capture the experimentally observed salt localization behavior would be valuable.

Reply: Thank you for this constructive comment. We agree that additional justification for the use of a two-dimensional homogenized model is necessary to clarify its validity in representing the three-dimensional ordered lattice.

In the revised manuscript, we clarified that the purpose of the simulation is to capture the macroscopic salt localization behavior arising from spatial differences in evaporation rate, rather than resolving detailed three-dimensional flow within individual unit cells. The experimental evaporator consists of a periodic ordered lattice, where identical unit cells are repeatedly arranged along the lateral directions. Due to this structural periodicity, the transport behavior in the transverse direction is spatially repetitive, and the dominant gradients occur along the vertical direction associated with height-dependent evaporation and liquid supply. Therefore, the three-dimensional lattice can be reasonably approximated by a representative two-dimensional cross-sectional model without losing the essential physics governing salt localization.

Furthermore, the two-dimensional homogenized model incorporates experimentally measured evaporation rates for different regions (outer and inner surfaces), ensuring that the simulation reflects the actual evaporation-driven transport conditions observed in experiments. This approach captures the key mechanism responsible for salt localization, namely the imbalance between evaporation-induced salt accumulation and capillary-driven liquid replenishment between regions with different evaporation fluxes. As a result, the reduced model is sufficient to reproduce the experimentally observed preferential salt crystallization behavior.

These clarifications have been added to the revised supplementary materials, emphasizing that the periodic lattice structure and vertically dominant transport allow the three-dimensional system to be represented using a two-dimensional homogenized model while preserving the governing physics of salt localization.

On the revised supplementary materials

<From>

[Method S2] The liquid domain was modeled as a homogenized porous region, reflecting the interconnected lattice architecture of the experimental samples.

<To>

[Method S2] The liquid domain was modeled as a homogenized porous region, reflecting the interconnected lattice architecture of the experimental samples. Because the experimental evaporators consist of periodically repeated lattice units, transport in the lateral direction is spatially repetitive. Therefore, the three-dimensional structure can be reasonably represented by a two-dimensional homogenized cross-sectional model capturing the dominant vertical concentration gradients.

4. The manuscript reports salt recovery efficiencies of approximately 96% and 97.49%. A brief discussion clarifying the origin of the unrecovered fraction would improve the completeness of the analysis. In particular, it would be helpful to indicate whether this portion remains trapped within the lattice structure, dissolved in residual brine, or is lost during the mechanical collection process.

Reply: Thank you for your constructive comments regarding the salt recovery efficiencies.

We clarify that the unrecovered fraction primarily originates from minor losses during the mechanical collection process. During salt harvesting by scrapping, a small portion of loosely attached crystals may detach and disperse, or remain adhered to localized regions of the lattice where complete mechanical removal is difficult. This residual salt can subsequently be dissolved during the post-recovery water-rinsing step to further reuse the multicellular solar evaporators in subsequent cycles.

In the revised manuscript, we have clarified the origin of the unrecovered fraction and note that the residual salt does not represent permanent loss of evaporation performance, but can be recovered through rinsing for further reuse.

On the revised manuscript; results

<From>

[Line 375-376] …achieving a salt recovery of 97.49% (Figure 5G and Table S11).

<To>

[Line 458-460] …achieving a salt recovery of 97.49% (Figure 5G and Table S11). The small unrecovered fraction is mainly attributed to minor losses during mechanical collection and trace residues remaining within the lattice, which can be subsequently dissolved during rinsing for reuse.

5. The evaporator is regenerated through gentle scraping and rinsing. Further discussion of the long-term durability of the graphite coating and the structural integrity of the printed lattice under repeated operational cycles would strengthen the practical relevance of the work. In particular, it would be useful to consider whether repeated saline wetting/drying, salt crystallization, and mechanical salt removal might gradually affect coating adhesion, surface wettability, or the geometric fidelity of the lattice.

Reply: We thank the reviewer for this important comment regarding the long-term durability of the graphite coating and structural integrity of the lattice under repeated operation.

We agree that demonstrating stability under repeated salt crystallization and regeneration cycles is essential for practical applications.

After gentle salt removal and rinsing with deionized water, the evaporator fully recovered its original performance without observable structural or functional degradation (Figures S25d). Quantitative cycling tests show minimal variation in evaporation rate, with the first cycle yielding 5.66 ± 0.38 kg m^{-2} h^{-1} and the final cycle yielding 5.53 ± 0.22 kg m^{-2} h^{-1}, indicating negligible performance decay. These results confirm that repeated saline wetting/drying, salt crystallization, and mechanical salt removal do not adversely affect evaporation performance.

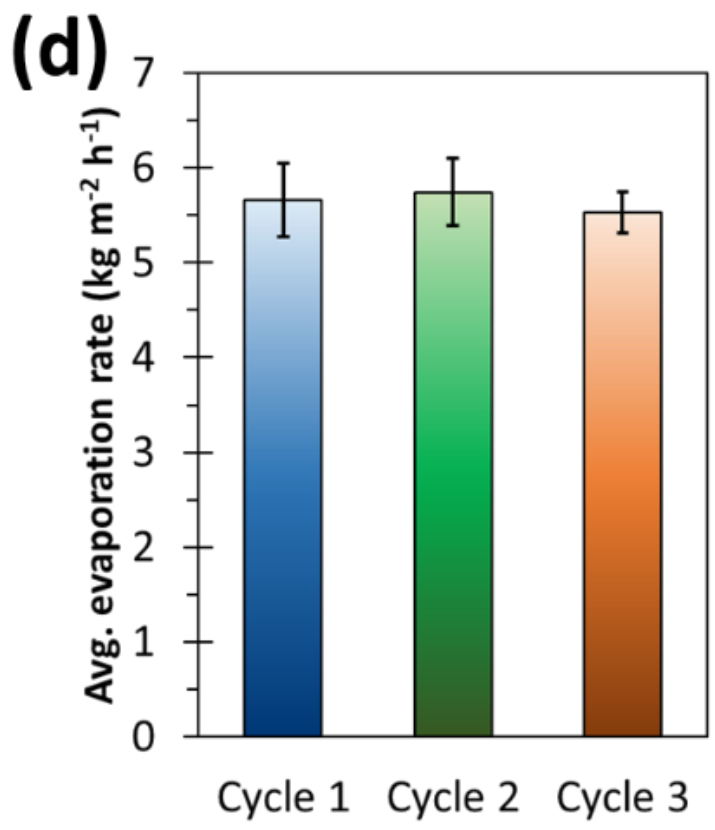

Figure S25. (d) average evaporation rate in each cycle.

Furthermore, the structural integrity of the printed lattice remained intact throughout repeated cycles. No deformation, fracture, or loss of geometric fidelity was observed after multiple crystallization–cleaning operations. The graphite coating also remained stable, with no visible delamination or particle detachment during rinsing or scraping. This stability is attributed to the conformal coating on the rigid lattice framework and the mild salt removal procedure, which avoids excessive mechanical stress.

In addition, outdoor experiments conducted continuously for 6 days showed no observable performance degradation, further supporting the robustness of both the graphite coating and the printed lattice under realistic operating conditions. These results collectively demonstrate that the evaporator maintains coating adhesion, surface wettability, and structural integrity during repeated operation.

These clarifications and quantitative durability results have been added to the revised manuscript to strengthen the practical relevance of the work.

On the revised manuscript; results

<From>

[Line 316-317] Repeated crystallization–cleaning cycles further confirmed excellent durability and reusability (Figure 4I).

<To>

[Line 391-394] Repeated crystallization–cleaning cycles further confirmed excellent durability and reusability, with minimal variation in evaporation rate, with the first cycle yielding 5.66 ± 0.38 kg m^{-2} h^{-1} and the final cycle yielding 5.53 ± 0.22 kg m^{-2} h^{-1}, indicating negligible performance decay (Figure S26).

6. While the manuscript emphasizes unit-cell architecture and macro-scale geometry as scalable design parameters, the current implementation relies on high-resolution DLP printing combined with multiple post-processing steps. A brief discussion on how this design could be translated to larger-scale manufacturing would strengthen the practical perspective of the work.

Reply: We thank the reviewer for this insightful comment regarding the scalability and practical implementation of the proposed geometry-engineered evaporators. We agree that discussing the potential translation to larger-scale manufacturing would strengthen the practical perspective of the work.

In the revised manuscript, we clarified that DLP 3D printing was employed primarily as a high-precision prototyping tool to systematically investigate the effects of unit-cell architecture and macro-scale geometry. The proposed design principles rely on periodic lattice architectures and geometry-controlled transport mechanisms, which are inherently scalable and not restricted to a specific fabrication technique. Therefore, the key performance advantages arise from structural topology rather than the printing method itself.

For large-scale manufacturing, similar ordered lattice structures could be fabricated using alternative scalable approaches such as mold-based replication, extrusion-based additive

manufacturing or modular assembly of repeated lattice units. Because the evaporators consist of periodically repeated unit cells, larger-area devices can be constructed by tiling multiple modules without altering the fundamental transport mechanisms. This modular scalability preserves both capillary transport pathways and lateral evaporation interfaces.

In addition, the post-processing steps used in this work, including graphite coating and surface activation, are solution-based processes that can be readily adapted to large-area fabrication through dip-coating, spray-coating, or continuous coating methods. These approaches are compatible with scalable manufacturing and do not require high-resolution printing at the final production stage.

These considerations have been added to the revised manuscript to emphasize that DLP printing was used for design validation, while the geometry-engineered architecture itself is compatible with scalable manufacturing routes.

On the revised manuscript; results

<From>

[Line 432-434] The liquid domain was modeled as a homogenized porous region, reflecting the interconnected lattice architecture of the experimental samples.

<To>

[Line 533-537] From a manufacturing perspective, digital light processing (DLP) 3D printing was selected for its high precision and structural fidelity, enabling systematic exploration of geometry-property-performance relationships. The evaporators are composed of periodically repeated lattice units, enabling larger-area devices to be realized through modular tiling or replication while preserving the governing capillary transport and lateral evaporation pathways.

Reviewer #4: In this manuscript, the authors employ high-resolution, resin-printed materials to develop 3D evaporators with enhanced evaporation rates for zero-liquid-discharge applications. Specifically, they report that regular geometric patterns can be engineered to control wetting in interfacial experiments, thereby directing salt crystallization to regions away from the evaporating surface. While the material preparation and the 3D-printed configurations are exciting, the novelty in solar interfacial evaporation is unclear. I do not believe the work demonstrates the technical novelty and rigor expected for Nature Communications, and I would strongly suggest that the authors consider a more specialized journal. In addition, my assessment is based on the concerns discussed below:

1. In figure 5G, despite blockage of the material due to increasing salt crystallization, the evaporation continues to increase. How is this possible? As the authors claimed, the purpose of the photo-thermal material was to increase absorption. However, as figs. 4G and 5G suggest, the evaporation rate increases regardless of the photo-thermal surface that is exposed to the incoming sunlight. If white scattering salt surface can already enhance evaporation rates to such large values, then what is the necessity of surface modification?

Reply: We sincerely thank the reviewer for this insightful comment regarding the continued increase in evaporation rate despite visible salt crystallization, and the role of the photothermal surface modification. We agree that this observation requires careful clarification.

The salt layer formed in our system does not act as a dense blocking crust. Instead, as described in the manuscript, the open-cell architecture of FBCC-+ promotes the formation of hydrated, micron-scale porous salt structures that remain hydraulically connected to the underlying lattice[2]. During long-term operation with 20 wt% NaCl, although large salt crystals progressively covered the external surface, the evaporation rate remained stable or slightly increased (Figures 4G and 5G).

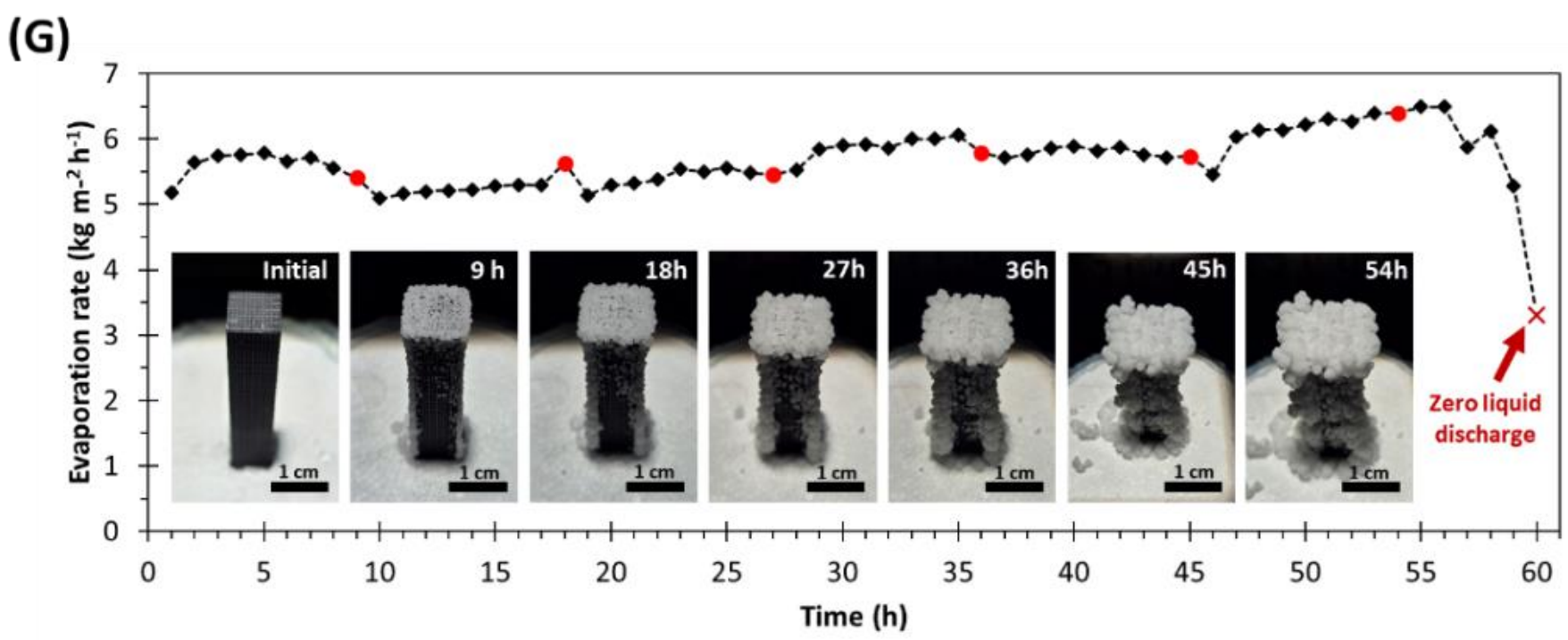

Figure 4. (G) Long-term evaporation stability in 20 wt% NaCl (inset: optical images corresponding to red dots).

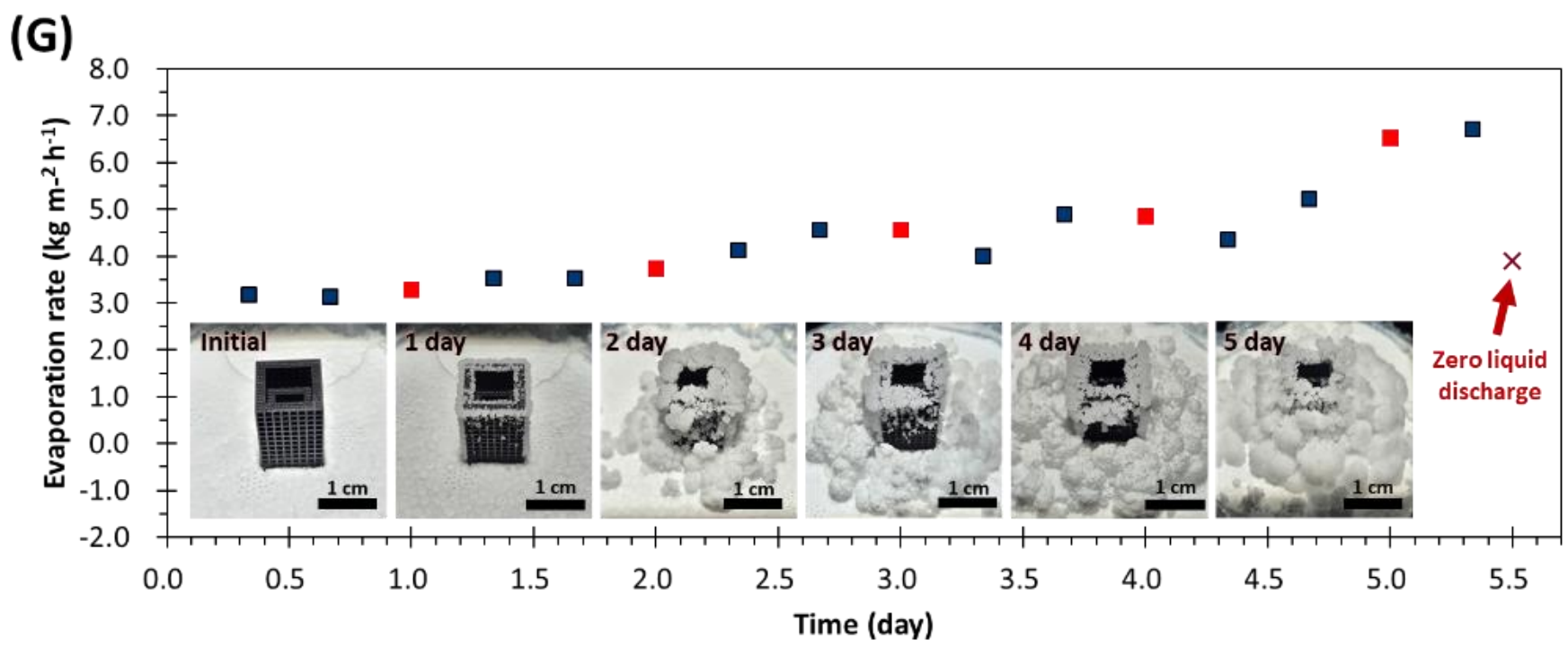

Figure 5. (G) Long-term stability under 20 wt% NaCl, with inset optical images showing localized salt accumulation corresponding to red dots.

Post-operation observations confirmed that the salt layer remained wetted and porous rather than forming a dry insulating shell (Figure S22). Optical inspection beneath the salt layer revealed a continuous liquid interface, and infrared imaging showed comparable temperatures between salt-covered and uncovered regions, indicating that evaporation occurs directly from the hydrated salt layer (Figure S23)[26]. This "wet-crystal evaporation" mechanism allows the salt layer to function as an auxiliary evaporating interface rather than a blocking layer.

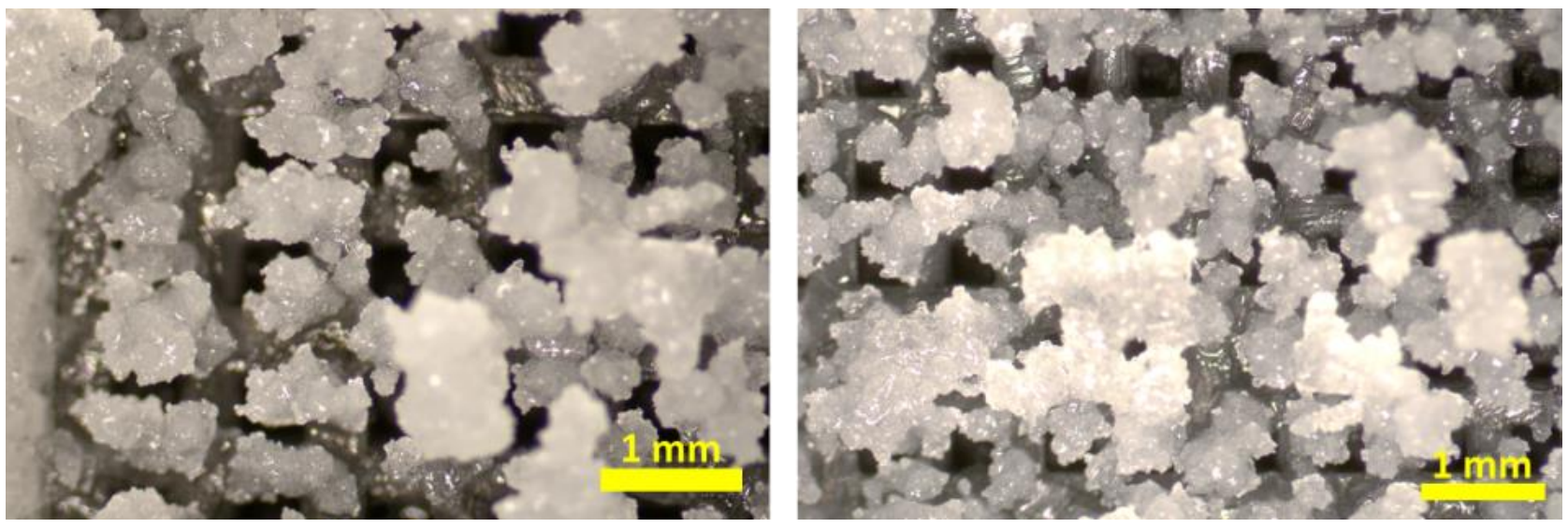

Figure S22. Optical images of wet salt crystals formed during long-term solar evaporation in 20 wt% NaCl solution after 36 h of operation (during third cycle)

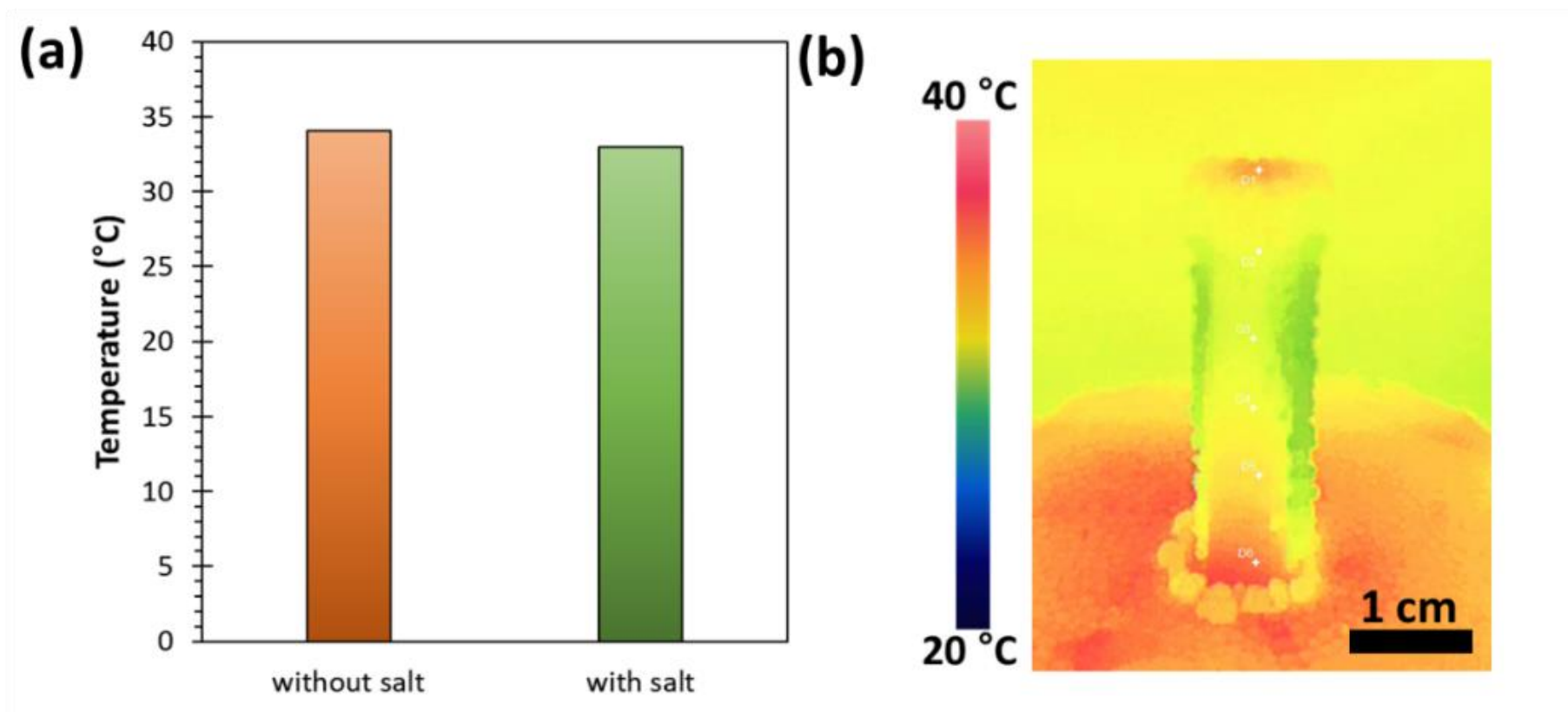

Figure S23. Thermal behavior of salt-covered FBCC-+_5 during evaporation. (a) Comparison of surface temperature profiles with and without salt coverage at the top surface; (b) infrared thermal images of the evaporator under salt-covered conditions.

Such behavior has been reported in several recent solar crystallization studies. For example, in the hierarchical porous salt evaporator reported by Yu et al. (2025), salt crystals self-assemble into a porous evaporation structure that enables passive liquid supply and sustained evaporation even under hypersaline conditions, with the salt layer actively participating in vapor generation rather than suppressing it[12]. Similarly, Ding et al. (2026) demonstrated that bio-inspired solar evaporators form porous coral-like salt shells that preserve brine transport and vapor escape, thereby maintaining stable evaporation despite extensive salt accumulation[13]. In addition, the tree-inspired evaporator reported by Ding et al. (2026) further showed that directional salt growth can produce porous salt structures that remain wetted and thermally coupled to the evaporator, enabling continuous evaporation and even enhanced performance during prolonged operation[16]. These studies collectively demonstrate that hydrated porous salt layers can expand the effective evaporation interface and participate in vapor generation.

Furthermore, solar crystallizer systems have also reported sustained evaporation despite significant salt accumulation, where salt growth occurs on external frames while internal transport pathways remain open. In these designs, the salt layer remains partially wetted and contributes to evaporation rather than blocking it, enabling stable performance under near-saturated brine conditions[17]. These observations are consistent with our results.

Therefore, the observed increase in evaporation rate in Figures 4G and 5G is not caused by white salt scattering alone. Instead, as crystallization progresses, the hydrated porous salt matrix increases the effective liquid–air interfacial area while maintaining continuous water supply. The evaporation interface thus expands from the original photothermal surface to include the salt matrix, leading to sustained or even enhanced evaporation.

Importantly, the photothermal surface modification remains essential. As shown in Figure 2E and 2F, the temperature of graphite-coated samples increased rapidly from ambient temperature (25.0 °C) to 53.1 °C within 10 min. In contrast, the uncoated resin sample exhibited only a slight temperature increase of 8.6 °C under the same conditions. The graphite-coated surface provides the initial photothermal conversion required to establish a high evaporation flux and generate the concentration gradients that drive controlled salt localization. Therefore, without this photothermal enhancement, the system would not produce sufficient heat input to initiate the wet-crystal evaporation regime. Once formed, the hydrated salt layer acts as a secondary evaporating interface, but its operation still relies on the underlying photothermal-driven heat supply.

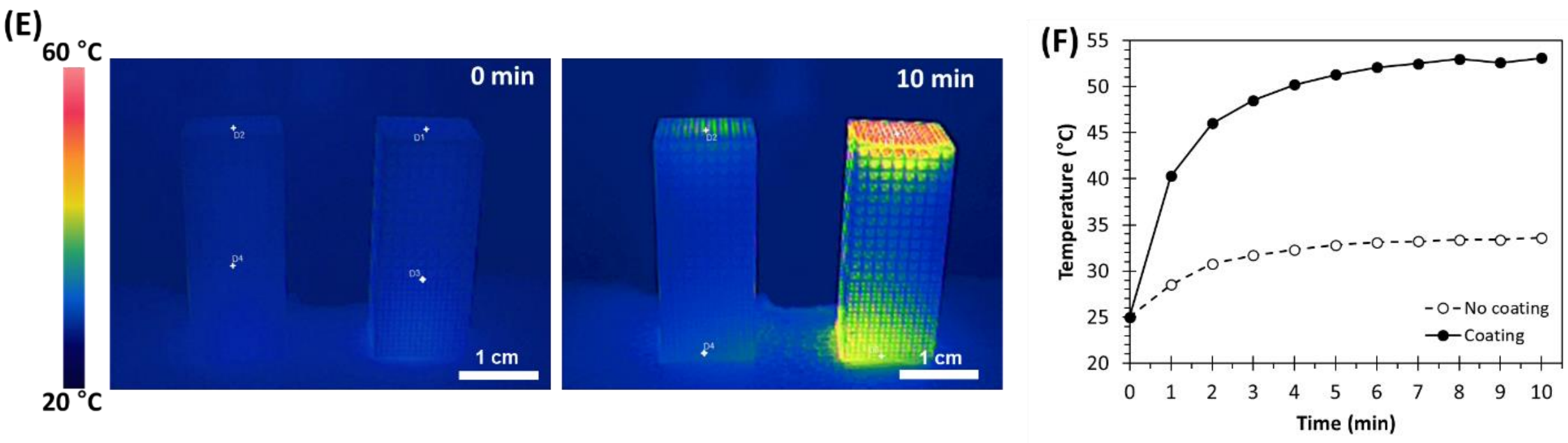

Figure 2. (E) Infrared thermal images of uncoated and coated multicellular structures under 1 sun irradiation in the dry state. (F) Temperature evolution of uncoated and coated multicellular structures under 1 sun irradiation.

To further clarify this mechanism, we have revised the manuscript to explicitly describe the hydrated porous salt growth mechanism, add schematic illustrations showing the wet-crystal evaporation process, and expand the discussion to explain how the salt layer functions as an auxiliary evaporation interface while the photothermal coating provides the primary heat input.

On the revised manuscript; results

<From>

[Line 298-301] This sustained performance arises from the open-cell architecture of FBCC-+_5, which facilitates the growth of hydrated, micron-scale porous salt structures[2]. Such multiscale porosity preserves hydraulic continuity and provides additional evaporative area, effectively offsetting geometric surface coverage[12].

<To>

[Line 367-375] This sustained performance arises from the open-cell architecture of FBCC-+_5, which enables continuous brine transport through the interconnected lattice and supports the growth of hydrated, micron-scale porous salt structures[2]. The relatively large pore openings direct salt nucleation along the lattice frames while maintaining open transport channels, allowing crystals to grow outward through continuous brine uptake without blocking liquid pathways (Figure S21). This multiscale porosity preserves hydraulic continuity and provides additional evaporative area, effectively offsetting geometric surface coverage[12]. Moreover, the porous salt layer formed on FBCC-+_5 reduces vapor diffusion resistance, allowing rapid vapor escape through interconnected pores while preventing mass-transfer limitations caused by vapor retention[13].

<Addition; text>

[Line 460-469] The increased evaporation rate observed after the second day can be attributed to the formation of a porous salt layer. Similar to the homogeneous FBCC-+_5 MSE, hydrated porous salt crystals develop on the outer surface of the structure. These porous deposits enable continuous brine uptake through capillary transport while growing outward without blocking liquid pathways[12]. Moreover, as shown in Figure 5G (inset), the salt crystals progressively extend outward, forming a coral-like porous network. This evolving porous salt structure effectively expands the evaporation interface and continuously harvests environmental heat, allowing the salt layer to function as an auxiliary evaporating medium rather than a failure mode[13]. This wet-crystal evaporation mechanism enables the cup-shaped hybrid MSE to sustain high-flux evaporation under extreme salinity.

On the revised supplementary materials

<Addition; Figure S21>

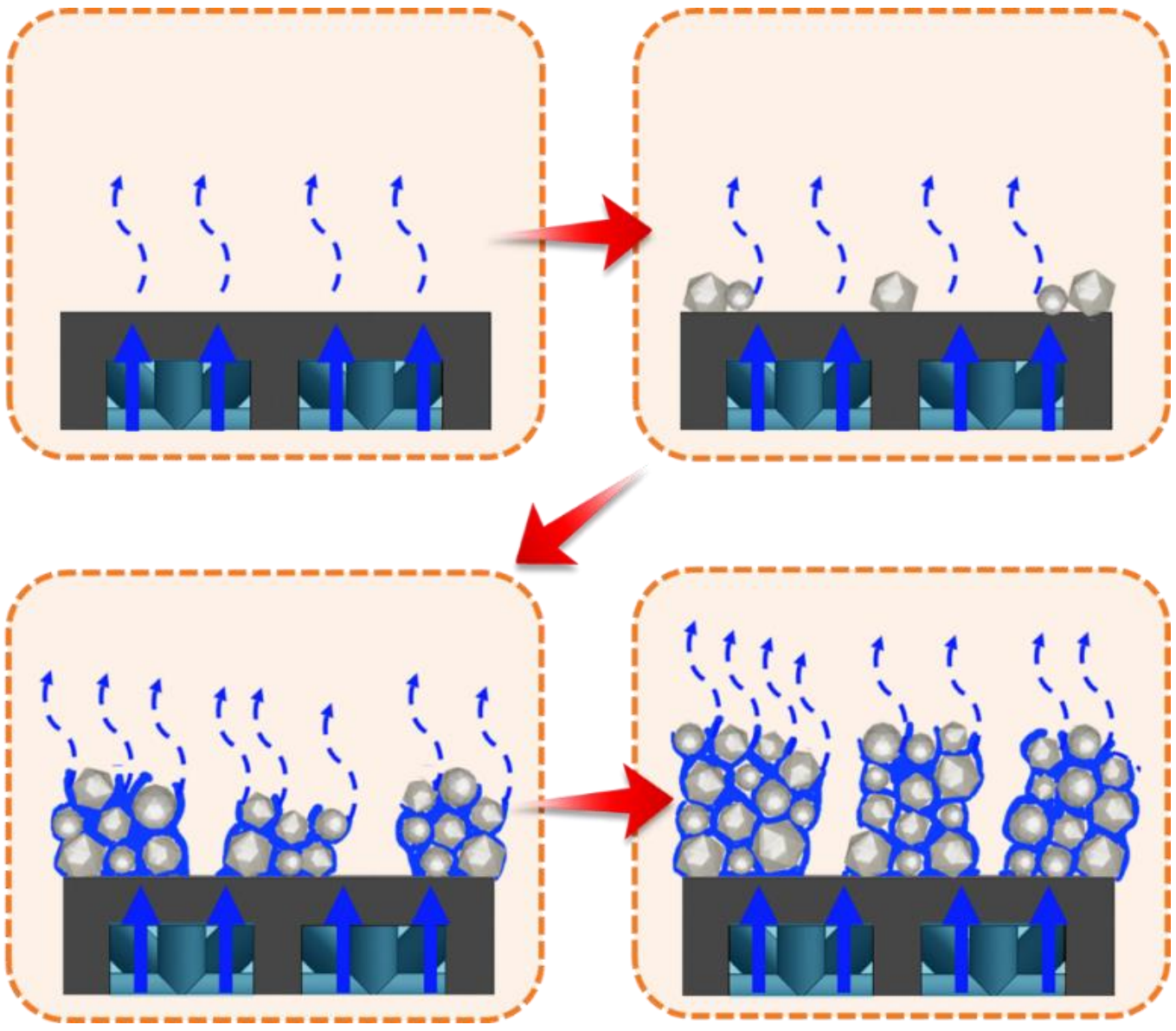

Figure S21. Schematic image illustrating the formation process of porous wet salts crystals on the surface of the MSE during continuous solar desalination.

2. Further, they argue that the lateral surfaces absorb environmental energy leading to the higher evaporation rates observed. However, as seen in the picture corresponding to 4-day mark in Fig. 5G, the lateral surfaces are completely covered with crystallized salts, attenuating environmental heat input via convection and radiation. These unexplainable trends in evaporation rates could be due to experimental artefacts which the authors should carefully check.

Reply: Thank you for your important comment regarding the apparent contradiction between salt coverage on the lateral surfaces and the continued increase in evaporation rate.

We agree that if the crystallized salt formed a dense, insulating shell, it could attenuate environmental heat transfer and reduce evaporation. However, in our system the deposited salt does not form a compact insulating shell. Instead, as clarified in the revised manuscript, the crystallized salt develops into a hydrated porous network that remains evaporation-active and thermally coupled to the underlying structure.

After the second day of operation, hydrated porous salt crystals progressively form on the outer BCC surface. These crystals grow outward while remaining wetted, producing an interconnected coral-like porous network (Figure 5G inset). Because this salt layer is porous and filled with liquid, it does not completely block convection or radiation. Rather, the liquid contained within the salt matrix provides additional liquid–air interfaces, allowing evaporation to occur directly from the salt structure itself[13]. Consequently, even though the lateral surface appears visually covered, the salt layer remains permeable to vapor transport.

Importantly, the porous salt layer continuously draws brine from the underlying lattice through capillary transport (Figure S21 and Figure S22). This maintains a wetted evaporation interface throughout the salt matrix, preventing the formation of a dry insulating crust. As the salt grows outward, the effective evaporation area expands from the original lattice surface to include the entire porous salt structure. Therefore, the lateral region remains active in evaporation despite apparent coverage by crystallized salt.

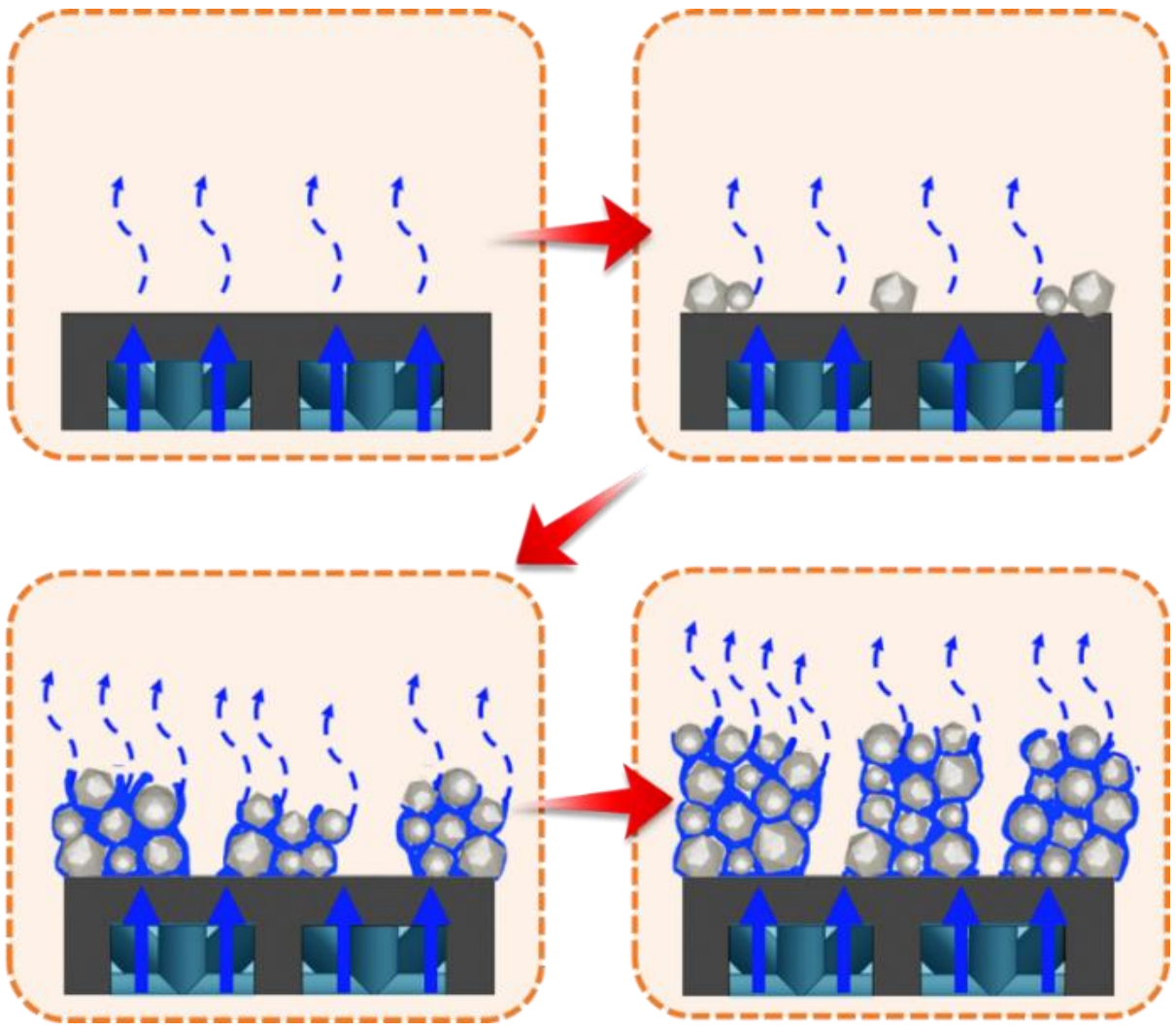

Figure S21. Schematic image illustrating the formation process of porous wet salts crystals on the surface of the MSE during continuous solar desalination.

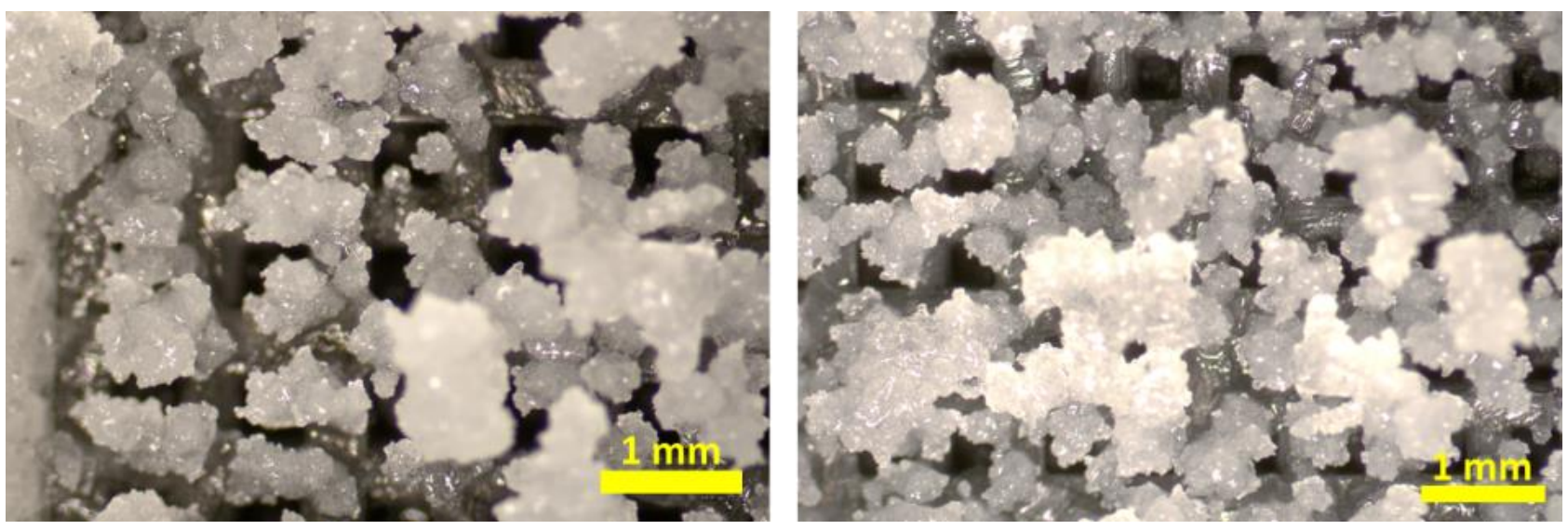

Figure S22. Optical images of wet salt crystals formed during long-term solar evaporation in 20 wt% NaCl solution after 36 h of operation (during third cycle)

This behavior explains why the evaporation rate does not decrease after salt coverage. Instead, the hydrated porous salt layer functions as an auxiliary evaporating medium, where environmental heat input is still accessible through the porous structure and evaporation occurs within the salt matrix itself. As a result, the total effective evaporation interface increases, leading to sustained or slightly enhanced evaporation after prolonged operation as observed in Figure 5G.

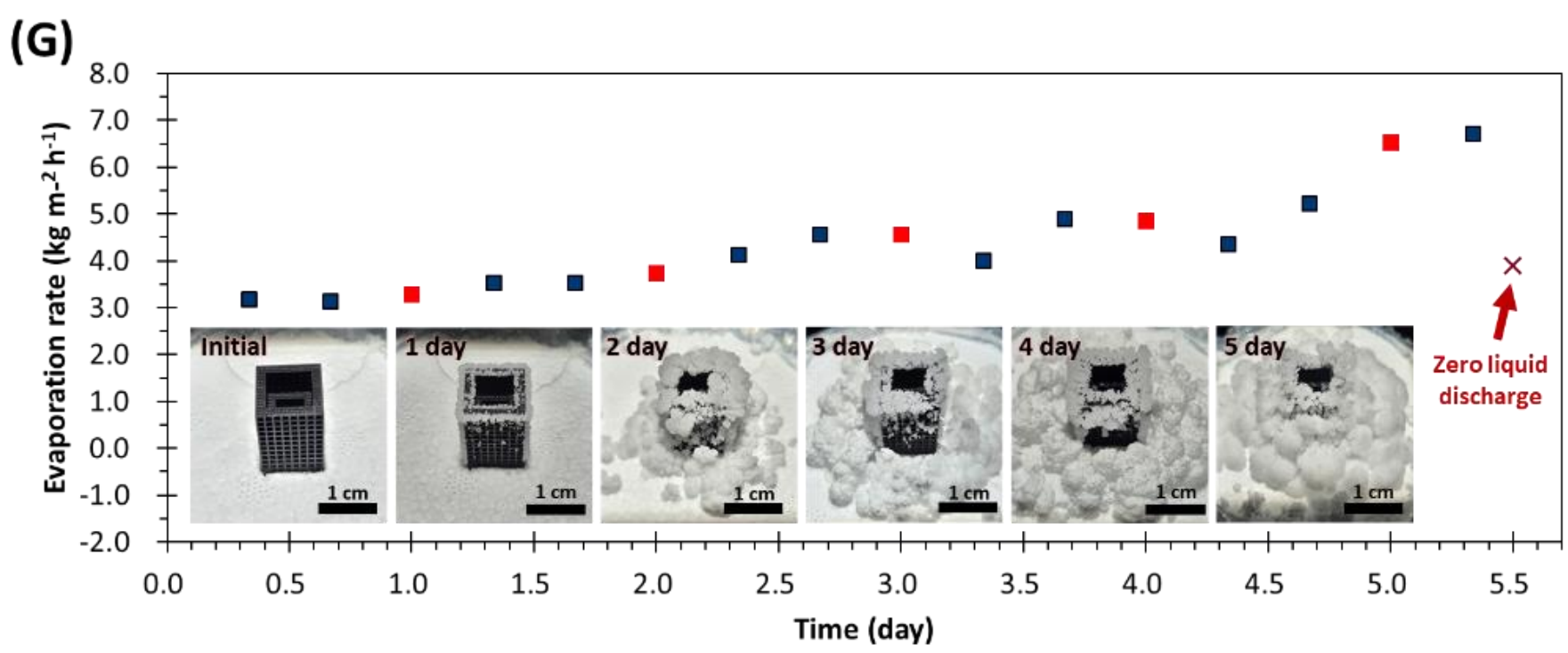

Figure 5. (G) Long-term stability under 20 wt% NaCl, with inset optical images showing localized salt accumulation corresponding to red dots.

To avoid misunderstanding, we have revised the manuscript to emphasize that the salt layer is not a dense blocking film but a wetted porous structure that preserves vapor escape pathways, maintains capillary liquid supply, and expands the effective evaporation interface while remaining thermally coupled to the environment. These clarifications explain why the lateral surfaces remain evaporation-active even when visually covered by salt and confirm that the observed trend is not due to experimental artefacts.

On the revised manuscript; results

<Addition; text>

[Line 460-469] The increased evaporation rate observed after the second day can be attributed to the formation of a porous salt layer. Similar to the homogeneous FBCC-+_5 MSE, hydrated porous salt crystals develop on the outer surface of the structure. These porous deposits enable continuous brine uptake through capillary transport while growing outward without blocking liquid pathways[12]. Moreover, as shown in Figure 5G (inset), the salt crystals progressively extend outward, forming a coral-like porous network. This evolving porous salt structure effectively expands the evaporation interface and continuously harvests environmental heat, allowing the salt layer to function as an auxiliary evaporating medium rather than a failure mode[13]. This wet-crystal evaporation mechanism enables the cup-shaped hybrid MSE to sustain high-flux evaporation under extreme salinity.

3. The authors claim that oxygen plasma treatment renders the surface hydrophilic. However, it is well-known that plasma-treated surfaces return to hydrophobic state due to organic contaminants from the ambient, effectively suppressing the effect of their surface treatment. Since the authors claim that this is essential, I wonder how this fits into their narrative of a sustained interfacial evaporation.

Reply: Thank you for this important comment regarding the long-term stability of plasma-induced hydrophilicity.

We agree that plasma-treated polymer surfaces can often undergo hydrophobic recovery due to surface reorientation or contamination, and therefore additional clarification is necessary.

In this work, the oxygen plasma treatment was applied after graphite coating, followed by rinsing with water to remove loosely bound graphite particles and subsequent overnight drying in an oven. All surface characterizations, including FTIR and contact angle

measurements, were performed after this complete post-treatment process Therefore, the measured wettability and spectroscopic features correspond to the fully stabilized surface rather than a transient plasma-activated state. FTIR analysis indicates the formation of oxygen-containing functional groups on the graphite surface, consistent with partial oxidation that introduces hydroxyl and ether-type functionalities[27,28] (Figure 2C). Unlike plasma-treated polymers, these oxidized carbon surfaces exhibit significantly reduced hydrophobic recovery due to their chemically stabilized surface functionalities. As a result, the hydrophilic behavior is not solely dependent on transient plasma activation but arises from the formation of a partially oxidized graphite layer.

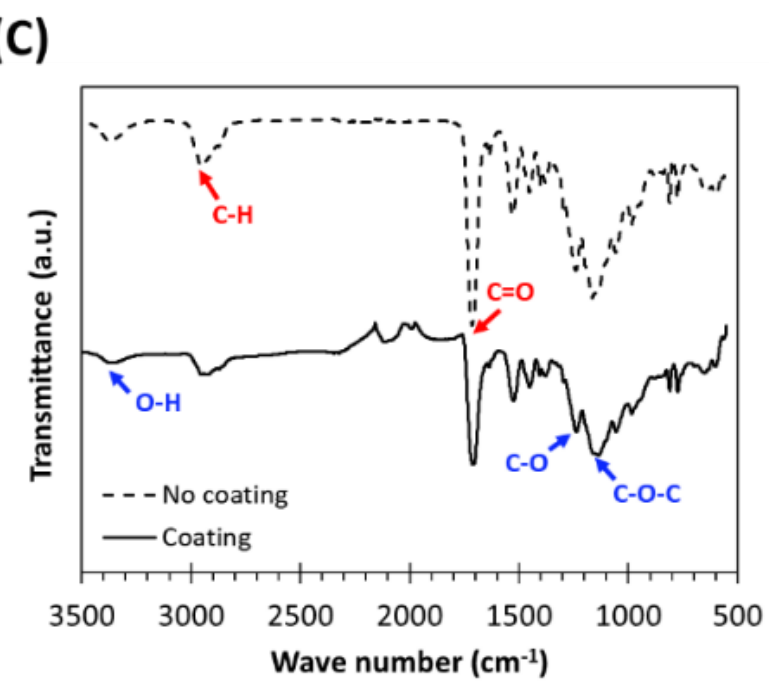

Figure 2. (C) FT-IR spectra of the samples before and after coating.

To further verify this stability, we conducted long-term contact angle measurements. The oxygen plasma–treated graphite-coated surface exhibited a contact angle of 44.7 ± 8.8° after graphite coating, O_2 plasma treatment, rinsing and drying, which remained nearly unchanged after prolonged storage on ambient conditions (46.6 ± 5.0° after 6 months). This negligible variation confirms that the surface does not revert to a hydrophobic state and that the hydrophilicity is maintained over extended periods. These results demonstrate that the plasma treatment leads to a stable hydrophilic interface suitable for sustained interfacial evaporation.

These clarifications and long-term wettability measurements have been added to the revised manuscript to emphasize that the hydrophilicity in this work originates from stable oxidation of the graphite layer and remains effective during prolonged operation.

On the revised manuscript; results

<From>

[Line 124-126] This surface modification enabled rapid wetting and continuous capillary water transport, which are essential for sustained interfacial evaporation.

<To>

[Line 133-140] This surface modification enabled rapid wetting and continuous capillary water transport, which are essential for sustained interfacial evaporation. The graphite-coated surface alone exhibited a slightly higher contact angle (77.6 ± 7.4°) than that of the pristine resin (70.6 ± 8.4°), indicating that graphite itself does not enhance wettability. In contrast, oxygen plasma treatment reduced the contact angle to 44.7 ± 8.8°, which remained stable even after 6 months (46.6 ± 5.0°), demonstrating long-term hydrophilicity arising from permanent surface modification of the graphite layer (Figure S4). This behavior was further examined by Fourier-Transform Infrared Spectroscopy (FTIR) (Figure 2C).

On the revised supplementary materials

<Addition; Figure S4>

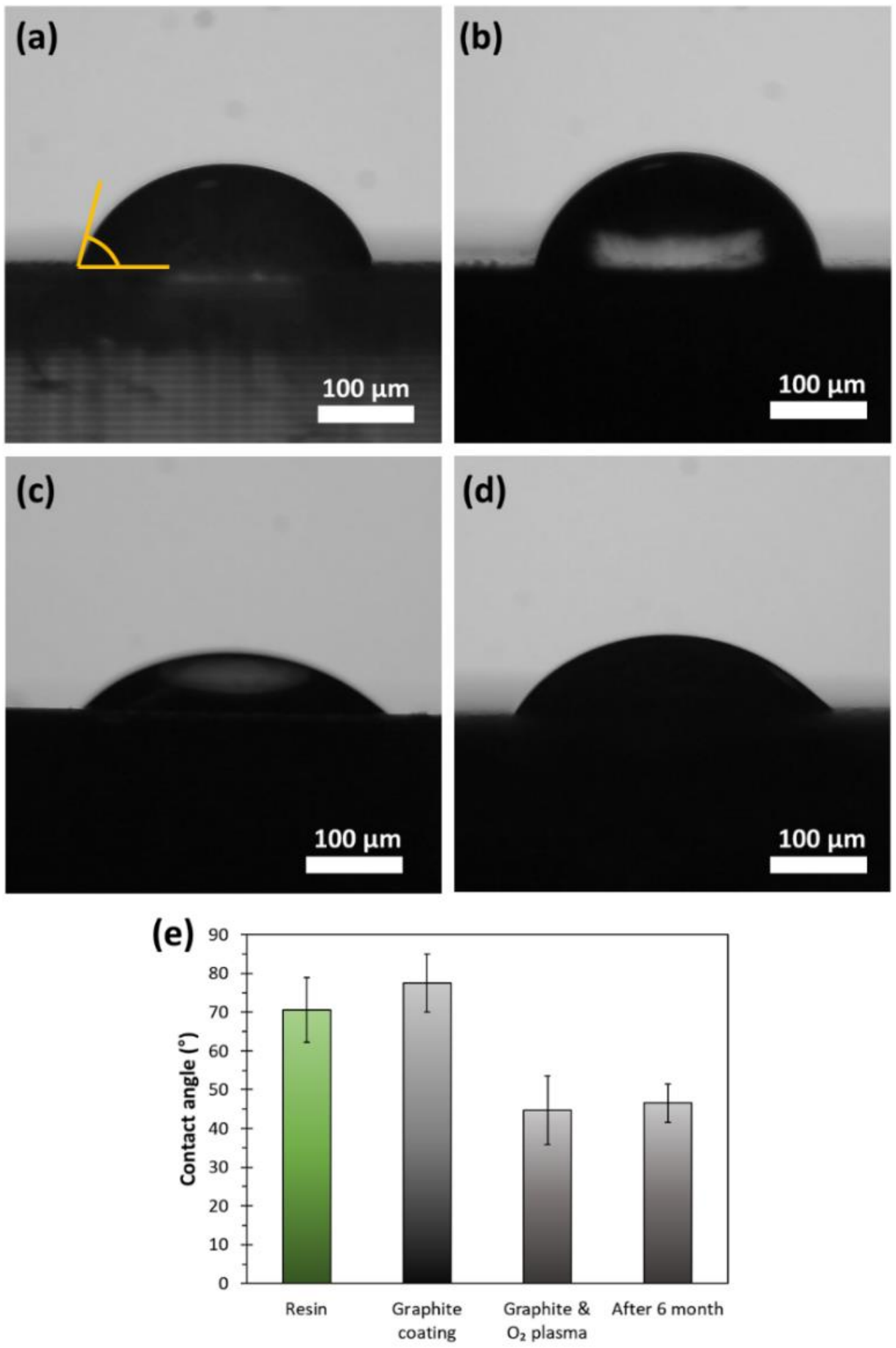

Figure S4. Contact angle measurements of the surface wettability. Optical images of water droplets on (a) pristine resin, (b) graphite-coated surface without O_2 plasma treatment, (c) graphite-coated surface after O_2 plasma treatment, and (d) the plasma-treated sample after 6 months. (e) Comparison of the measured contact angles for each condition.

4. In method 1, the authors subtract the dark condition's evaporation rate from the solar evaporation. This is largely erroneous and displays a lack of understanding of mass transfer. The convection-driven evaporation process is driven by a difference in water vapor concentration at the evaporating surface and the ambient.

$$\boldsymbol{m''_{evap} = g_m * C_g\left[c_{v,s}(T_s) - RH * c_{v,s}(T_\infty)\right]}$$

Where m''_{evap} is the evaporation flux, C_g is the molar density of dry air, $c_{v,s}(T_s)$ is the saturated vapor mole fraction at the surface temperature, RH is the ambient relative humidity, $c_{v,s}(T_\infty)$ is the saturated vapor mole fraction at the ambient temperature, and g_m is the mass transfer coefficient.

One can see that the evaporation rate is determined by the surface and ambient temperatures, and the relative humidity of the ambient. Subtracting the obtained value from the dark condition is erroneous because the dark/natural convection experiment is a separate, independent physical process. The buoyancy flow is downwards as the surface temperature is colder than the ambient. The value obtained for that has nothing to do with the value obtained for the solar evaporation.

Reply: Thank you for this insightful comment regarding the use of dark-condition subtraction in calculating evaporation efficiency.

We fully agree that evaporation is governed by coupled heat and mass transfer processes, and that the evaporation flux is determined by the vapor concentration gradient between the evaporating surface and ambient air:

$$m''_{evap} = g_m * C_g\left[c_{v,s}(T_s) - RH * c_{v,s}(T_\infty)\right]$$

In this framework, illumination modifies the boundary conditions (surface temperature, local convection, and vapor concentration gradient), and therefore the evaporation rate under solar irradiation corresponds to a newly established steady state. Therefore, we agree that subtracting the dark evaporation rate does not represent a strict physical decomposition of solar-driven and ambient-driven evaporation.

However, in the solar interfacial evaporation field, subtraction of dark evaporation is widely adopted as a baseline-normalized comparative metric, intended to quantify the net enhancement under illumination while maintaining identical environmental conditions[19,20,29] . In this context, the calculated value is typically interpreted as an apparent energy utilization efficiency rather than a strict thermodynamic efficiency.

For instance, Zhang et al. (*Nat. Commun.,* 2021) designed a next-generation solar crystallizer for zero-liquid discharge and reported an efficiency of 94.3%, demonstrating enhanced evaporation via structural heat management[17]. Yan et al.(*Chem. Eng. J.,* 2023)

fabricated a PC@PPy porous membrane evaporator and reported an efficiency of 93.57%[30]. Similarly, Meng et al. (*J. Mater. Chem. A*, 2022) reported an N-doped carbon@Cu core–shell evaporator with an apparent energy utilization efficiency of 137.1%, explicitly exceeding unity due to additional environmental heat harvesting[25].

For three-dimensional architectures, efficiencies exceeding 100% are even more common. Wu et al. (*Nat. Commun*., 2020) fabricated a biomimetic 3D solar evaporator with localized crystallization and reported an efficiency of 98.9%[20], while Wang et al. (*Adv. Sci.*, 2024) developed a 3D cellular solar crystallizer (ATSC) achieving 175% efficiency due to enlarged evaporation area and environmental heat harvesting[2]. Li et al. (*J. Clean Prod.*, 2024) reported a pyramid-shaped solar evaporator with an apparent efficiency of 186.76%, attributed to multi-surface evaporation and enhanced heat reuse[21]. Furthermore, Zhu et al. (*Desalination*, 2023) fabricated a volcanic-shaped 3D evaporator exhibiting an apparent energy efficiency of 220.34%, where the authors explicitly attributed efficiencies above unity to multiscale structure–induced environmental energy harvesting and enhanced interfacial evaporation[31].

These representative studies, summarized in Table S9, demonstrate that baseline-subtracted apparent energy utilization efficiency is widely adopted across both 2D and 3D solar evaporators. In particular, three-dimensional architectures introduce lateral evaporation, radiative heat exchange, and convective coupling with the ambient, allowing additional environmental energy input[32]. Consequently, efficiencies exceeding 100% are commonly reported and interpreted as system-level energy harvesting rather than violation of energy conservation.

Therefore, in this work, the subtraction of the dark evaporation rate was used only to quantify the net evaporation enhancement under illumination, consistent with common practice in the solar evaporation field. To avoid ambiguity, we have revised the manuscript to explicitly define the reported metric as apparent energy utilization efficiency, rather than as a strict thermodynamic efficiency. This clarification ensures consistency with prior literature and provides a fair basis for comparison with previously reported solar evaporators.

On the revised manuscript; results

<From>

[Line 274-279] This synergistic coupling of environmental heat with incident solar energy yields an apparent energy utilization efficiency of 326.9% under 1 sun illumination (Method S1, Figure S9), exceeding that of conventional two-dimensional evaporators[18,30,33]. … Together, these observations …

<To>

[Line 342-348] This synergistic coupling of environmental heat with incident solar energy yields an apparent energy utilization efficiency of 326.9% under 1 sun illumination (Method S1 and Figure S20), exceeding that of conventional two-dimensional evaporators[18,30,33] (Table S9). It should be noted that this value includes additional heat uptake from the surrounding environment through the lateral surfaces; therefore, the calculated efficiency should be interpreted as an apparent metric rather than a purely solar-to-vapor conversion efficiency. Together, these observations …

On the revised supplementary materials

<From>

[Method S1] The energy utilization efficiency (η) of the solar evaporators under illumination was calculated using the conventional latent-heat-based definition widely adopted in solar evaporation studies. To isolate the contribution of solar irradiation, the net evaporation rate was first determined by subtracting the dark evaporation component

<To>

[Method S1] The apparent energy utilization efficiency (η) of the solar evaporators under illumination was calculated using the conventional latent-heat-based definition widely adopted in solar evaporation studies[18]. To estimate the additional evaporation associated with solar irradiation under identical environmental conditions, the net evaporation rate was first determined by subtracting the dark evaporation component:

<From>

[Method S1] Values of η exceeding unity reflect the combined contribution of direct solar energy and harvested ambient thermal energy to vapor generation, rather than a violation of energy conservation. This formulation enables consistent comparison of evaporation performance while accounting for geometry-enabled ambient energy harvesting effects.

<To>

[Method S1] Values of η exceeding unity reflect the combined contribution of direct solar energy and harvested ambient thermal energy to vapor generation, rather than a violation of energy conservation. In this context, the subtraction-based formulation serves as a baseline-normalized, comparative metric, and does not represent a strict physical separation of solar and environmental contributions, particularly in three-dimensional architectures where environmental energy exchange is significant.

<Addition; Table S9>

Table S9. Apparent energy utilization efficiency of different solar evaporators find in literature

Solar evaporator	Configuration	Apparent energy utilization efficiency (%)	Ref.
rGO foam	2D	99.4	[1]
Janus CB-PDMS/PMF	2D	75	[2]
PC@ PPy porous membrane	2D	93.57	[3]
PDA/CB@MS salt crystallization system	2D	93	[4]
NC@Cu core–shell nanostructure	2D	137.1	[5]
Umbrella evaporator	3D	85.5	[6]
3D solar crystallizer	3D	94.3	[7]
Biomimetic 3D solar evaporator	3D	98.9	[8]
ATSC	3D	175	[9]
Pyramid-shaped solar evaporator	3D	186.76	[10]
Volcanic evaporator	3D	220.34	[11]
FBCC-+_5	3D	326.9	This study

6. In Figure S9, the authors report an apparent energy utilization of >200% for the 2 cm geometry and above. This is an improbably high value. Considering that the heat transfer coefficient (radiation + natural convection) is ≈10 W m^{-2} K^{-1}, and ΔT ≈ 1 °C, achieving a 100% power enhancement from the environment would require an area roughly 100 times larger than the top, incident area. This claim raises serious concerns about how the ambient energy absorption and evaporation rates were calculated.

Reply: Thank you for this important comment regarding the reported apparent energy utilization efficiency exceeding 200%.

We agree that, when interpreted as a strict thermodynamic conversion efficiency, such values may appear unusually high. However, in this work the reported quantity is apparent energy utilization efficiency, calculated using the widely adopted baseline-subtracted formulation and intended for comparative evaluation, rather than representing the fraction of solar energy converted solely from the incident top surface.

The reviewer's estimation assumes that environmental heat transfer can be approximated using a simplified $q=hA\Delta T$ relation based on a single characteristic area and uniform heat-transfer conditions. However, the present multicellular architecture introduces a distributed evaporation interface, where evaporation occurs simultaneously across the top surface and height-dependent lateral surfaces. As the exposed height increases, the total evaporation interface increases while the projected solar input area remains constant. Under these conditions, the evaporation process is governed by spatially distributed heat and mass transfer rather than by a single planar interface, and environmental heat exchange occurs over the enlarged three-dimensional surface.

Furthermore, infrared measurements indicate that the lateral surfaces remain close to ambient temperature during operation, suggesting that evaporation from these regions is sustained by continuous heat exchange with the surroundings (Figure 3E).

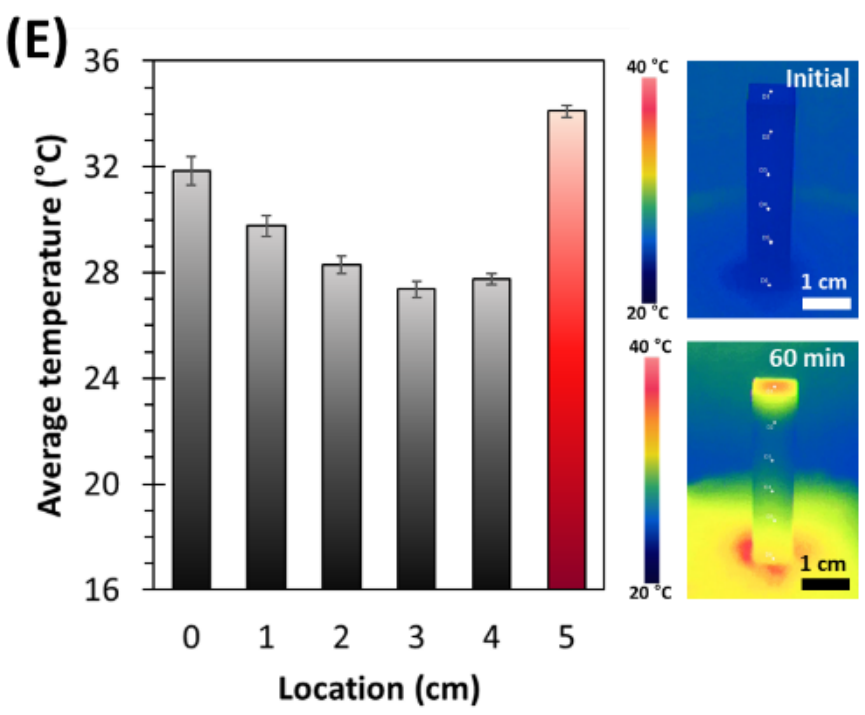

Figure 3. (E) Spatially averaged temperature distribution of FBCC-+_5 during evaporation.

In such distributed evaporation systems, even modest temperature differences can provide a meaningful contribution when integrated over an enlarged interface. Consequently, the net evaporation rate can exceed the value expected from solar input alone, leading to apparent efficiencies greater than unity.

Apparent energy utilization efficiencies exceeding 100% have been widely reported for three-dimensional solar evaporators using the same calculation framework. For instance, Meng et al. (*J. Mater. Chem. A*, 2022)[25] reported an apparent efficiency of 137.1%, Wang et al. (*Adv. Sci.*, 2024)[2] reported 175% for a 3D cellular solar crystallizer, Li et al. (*J. Clean Prod.*, 2024)[21] reported 186.76% for a pyramid-shaped evaporator, and Zhu et al. (*Desalination*, 2023)[31] reported 220.34% for a volcanic-shaped 3D evaporator. Recently, Tang et al. (*Energy Environ. Sci.*, 2025)[32] reported a 3D-printed hierarchical chimney solar evaporator achieving an apparent energy efficiency of 317%. In these studies, efficiencies above unity were attributed to enlarged evaporation interfaces and additional environmental heat harvesting enabled by three-dimensional geometries. The present results follow the same interpretation and calculation methodology for consistency with prior literature.

Importantly, the reported value does not imply that the system extracts more solar power than is incident on the top surface. Rather, it indicates that the latent heat required for evaporation is supplied by both solar irradiation and environmental heat exchange across the enlarged three-dimensional interface. This interpretation is consistent with the observed increase in evaporation rate with increasing structure height.

To avoid potential misunderstanding, we have revised the manuscript to explicitly define the reported metric as apparent energy utilization efficiency, clarify its use for comparative purposes, and emphasize that efficiencies exceeding 100% arise from combined solar and environmental heat harvesting in three-dimensional evaporator architectures.

On the revised manuscript; results

<From>

[Line 274-279] This synergistic coupling of environmental heat with incident solar energy yields an apparent energy utilization efficiency of 326.9% under 1 sun illumination (Method S1, Figure S9), exceeding that of conventional two-dimensional evaporators[18,30,33]. … Together, these observations …

<To>

[Line 342-348] This synergistic coupling of environmental heat with incident solar energy yields an apparent energy utilization efficiency of 326.9% under 1 sun illumination (Method S1 and Figure S20),

exceeding that of conventional two-dimensional evaporators[18,30,33] (Table S9). It should be noted that this value includes additional heat uptake from the surrounding environment through the lateral surfaces; therefore, the calculated efficiency should be interpreted as an apparent metric rather than a purely solar-to-vapor conversion efficiency. Together, these observations …

On the revised supplementary materials

<From>

[Method S1] The energy utilization efficiency (η) of the solar evaporators under illumination was calculated using the conventional latent-heat-based definition widely adopted in solar evaporation studies. To isolate the contribution of solar irradiation, the net evaporation rate was first determined by subtracting the dark evaporation component

<To>

[Method S1] The apparent energy utilization efficiency (η) of the solar evaporators under illumination was calculated using the conventional latent-heat-based definition widely adopted in solar evaporation studies[18]. To estimate the additional evaporation associated with solar irradiation under identical environmental conditions, the net evaporation rate was first determined by subtracting the dark evaporation component:

<From>

[Method S1] Values of η exceeding unity reflect the combined contribution of direct solar energy and harvested ambient thermal energy to vapor generation, rather than a violation of energy conservation. This formulation enables consistent comparison of evaporation performance while accounting for geometry-enabled ambient energy harvesting effects.

<To>

[Method S1] Values of η exceeding unity reflect the combined contribution of direct solar energy and harvested ambient thermal energy to vapor generation, rather than a violation of energy conservation. In this context, the subtraction-based formulation serves as a baseline-normalized, comparative metric, and does not represent a strict physical separation of solar and environmental contributions, particularly in three-dimensional architectures where environmental energy exchange is significant.

<Addition; Table S9>

Table S9. Apparent energy utilization efficiency of different solar evaporators find in literature

Solar evaporator	Configuration	Apparent energy utilization efficiency (%)	Ref.
rGO foam	2D	99.4	[1]
Janus CB-PDMS/PMF	2D	75	[2]
PC@ PPy porous membrane	2D	93.57	[3]
PDA/CB@MS salt crystallization system	2D	93	[4]
NC@Cu core–shell nanostructure	2D	137.1	[5]
Umbrella evaporator	3D	85.5	[6]
3D solar crystallizer	3D	94.3	[7]
Biomimetic 3D solar evaporator	3D	98.9	[8]
ATSC	3D	175	[9]
Pyramid-shaped solar evaporator	3D	186.76	[10]
Volcanic evaporator	3D	220.34	[11]
FBCC-+_5	3D	326.9	This study

7. Comparing the modified geometries with a baseline geometry is a good strategy to understand the effects of internal structure on the evaporation rate. However, a question remains: is this observed effect universally valid for all hydrophilic materials, or is it heavily dependent on the specific surface chemistry of the material used in this study? The authors should comment on this limitation.

Reply: Thank you for this insightful comment regarding the generality of the observed structural effects. We appreciate the reviewer's point on whether the reported enhancement is universally applicable or dependent on the specific surface chemistry of the material.

In this study, all samples were fabricated using the same base material (3D-printed resin) and underwent identical surface treatments, including graphite coating for photothermal conversion and oxygen plasma treatment to ensure hydrophilicity. Therefore, the surface chemistry, wettability, and optical properties were kept consistent across all cases. Under these controlled conditions, the observed differences in evaporation performance can be attributed primarily to variations in geometry (i.e., unit cell type), rather than differences in material properties.

From a physical perspective, the key parameters governing the observed behavior, such as liquid–solid contact perimeter, porosity, and transport pathways, are inherently geometric in nature. These factors directly influence capillary transport, liquid supply, and effective evaporation interface, which are expected to play similar roles in other hydrophilic systems where sufficient wettability is ensured.

At the same time, we agree with the reviewer that the absolute magnitude of evaporation performance can depend on material-specific properties, such as intrinsic wettability, surface roughness, and photothermal conversion efficiency. Therefore, while the qualitative trends induced by geometry (e.g., enhanced capillary transport and increased effective evaporation interface) are expected to be broadly applicable, the quantitative performance may vary depending on the specific material system.

Therefore, the observed effect is not strictly universal for all hydrophilic materials, but is expected to be generally valid for systems that maintain sufficient wettability and comparable liquid transport conditions, while the magnitude of the enhancement remains dependent on the specific surface properties.

To clarify this point, we have revised the manuscript to explicitly state that the conclusions of this work focus on the role of geometry under controlled material conditions, and that extension to other material systems may require consideration of their surface properties. This clarification has been added to avoid overgeneralization and to better define the scope of applicability of the present results.

On the revised manuscript; results

<From>

[Line 468-473] Collectively, these results demonstrate that unit cell and macro-scale geometry can be used as programmable design variables to control interfacial transport, heat localization, and salt deposition pathways in solar evaporators. The structure-function relationships identified here provide

a generalizable framework for designing scalable, durable, and high-efficiency solar evaporation systems, advancing solar-driven desalination and ZLD toward real-world implementation.

<To>

[Line 572-580] Collectively, these results demonstrate that unit cell and macro-scale geometry can be used as programmable design variables to control interfacial transport, heat localization, and salt deposition pathways in solar evaporators. The structure–function relationships identified here suggest general design principles under controlled material and wettability conditions. While these geometric effects are expected to be broadly applicable, the absolute performance may depend on material-specific properties, including wettability, optical absorption, and photothermal conversion efficiency. This work thus establishes a geometry-driven framework for designing scalable, durable, and high-performance solar evaporation systems, advancing solar-driven desalination and ZLD toward real-world implementation.

8. For the evaporation rate with tall structures shown in Figure 4, there might be issues with diffuse light reflections from the polystyrene foam to the tall structure, this should be considered in energy conservation. This might be reason for the enhanced efficiency rather than just from the environmental energy absorption.

Reply: Thank you for your constructive comment regarding the potential influence of diffuse light reflection from the polystyrene (PS) foam on the evaporation performance of tall structures. We agree that this factor should be carefully examined to ensure that the observed enhancement is not artificially influenced by reflected light.

To address this concern, we conducted control experiments using a non-reflective sheet placed on top of the PS foam to suppress any diffuse light reflection toward the tall evaporator structures. The evaporation performance measured under this condition remained nearly identical to that obtained with the original PS foam. In addition, infrared thermal imaging showed that the lateral surfaces remained cooler than the top surface, consistent with the formation of cold-evaporation zones rather than additional photothermal heating from reflected light. These observations indicate that diffuse reflection from the PS foam has a negligible influence on the evaporation performance.

Furthermore, only a slight temperature increase near the base of the structure was observed, which is consistent with localized reflection effects and does not significantly contribute to the overall evaporation rate. The dominant temperature gradient, higher temperature at the top surface and lower temperature along the lateral surfaces, remains unchanged in the control experiments, supporting the interpretation that the enhanced performance arises from

environmental heat harvesting and increased lateral evaporation area rather than reflected illumination.

These control experiments and corresponding infrared thermal images have been added to the revised Supplementary Information (Figure S19), and the discussion has been clarified in the revised manuscript to explicitly address the potential influence of diffuse light reflection.

On the revised manuscript; results

<From>

[Line 276-279] A slight temperature increase near the base (~31.8 °C) was observed, which is attributed to partial light reflection from the PS foam rather than thermal leakage from the evaporation surface, further confirming efficient heat localization.

<To>

[Line 337-342] A slight temperature increase near the base (~31.8 °C) was observed, which is attributed to partial light reflection from the PS foam rather than thermal leakage from the evaporation surface, further confirming efficient heat localization. Control experiments using a non-reflective sheet placed on the PS foam yielded comparable evaporation rates, while the lateral surfaces temperature remained lower than the top surface, indicating that reflected light has a negligible influence on evaporation performance and confirming effective heat localization (Figure S19).

On the revised supplementary materials

<Addition; Figure S19>

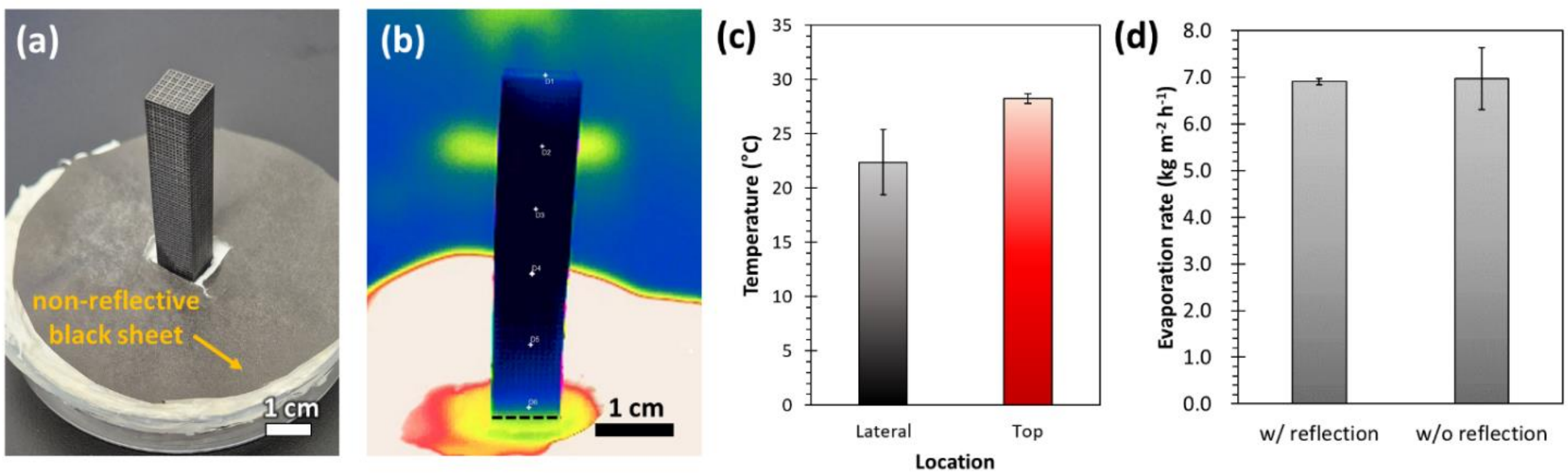

Figure S19. Experiments to identify the effect of partial light reflection from the polystyrene (PS) foam. (a) Optical image of the experimental setup incorporating a non-reflecting sheet on the PS foam. (b) Infrared thermal images of the FBCC-+_5 during evaporation with non-reflecting sheet incorporated. (c) Average temperatures of the lateral and top surfaces during evaporation under 1 sun irradiation. (d) Comparison of evaporation rates with and without PS foam reflection.

9. The authors should comment on the ultimate limits of this spatial localization under a continuous feed scenario. Specifically, if the liquid reservoir is continuously replenished, at what point does the salt volume on the BCC side become so massive that it physically bridges over to the FBCC side or structural degradation occurs? Clarifying the mechanical limits of salt accumulation before a cleaning/washing cycle is triggered would better address the long-term, continuous practicality of this hybrid design

Reply: We thank the reviewer for this important comment regarding the ultimate limits of salt accumulation and the practical operation window under continuous-feed conditions.

We agree that, under continuous replenishment, excessive salt growth could eventually lead to bridging between regions or mechanical interference, and that defining this limit is important for long-term practicality.

Importantly, all experiments in this study were conducted under a ZLD-type batch operation, where the saline reservoir was allowed to fully deplete before regeneration. Under this operation mode, the accumulated salt remained localized within the designated BCC region and did not reach a level that caused bridging to the FBCC-+ region or structural degradation. After completion of each cycle, the accumulated salt could be readily removed by simple washing, and the evaporator retained its structural integrity and evaporation performance, indicating good mechanical robustness and reversibility.

In the present hybrid structure, salt growth occurs outward from the BCC region in the form of a hydrated porous network. Because the salt layer grows away from the primary FBCC-+ evaporation surface, the risk of immediate bridging is reduced. The limiting condition is therefore not the loss of localization itself, but the gradual buildup of attached salt aggregates that could eventually reduce vapor escape pathways or physically extend toward neighboring regions. Within the tested ZLD cycle, this threshold was not reached (Figure 5G).

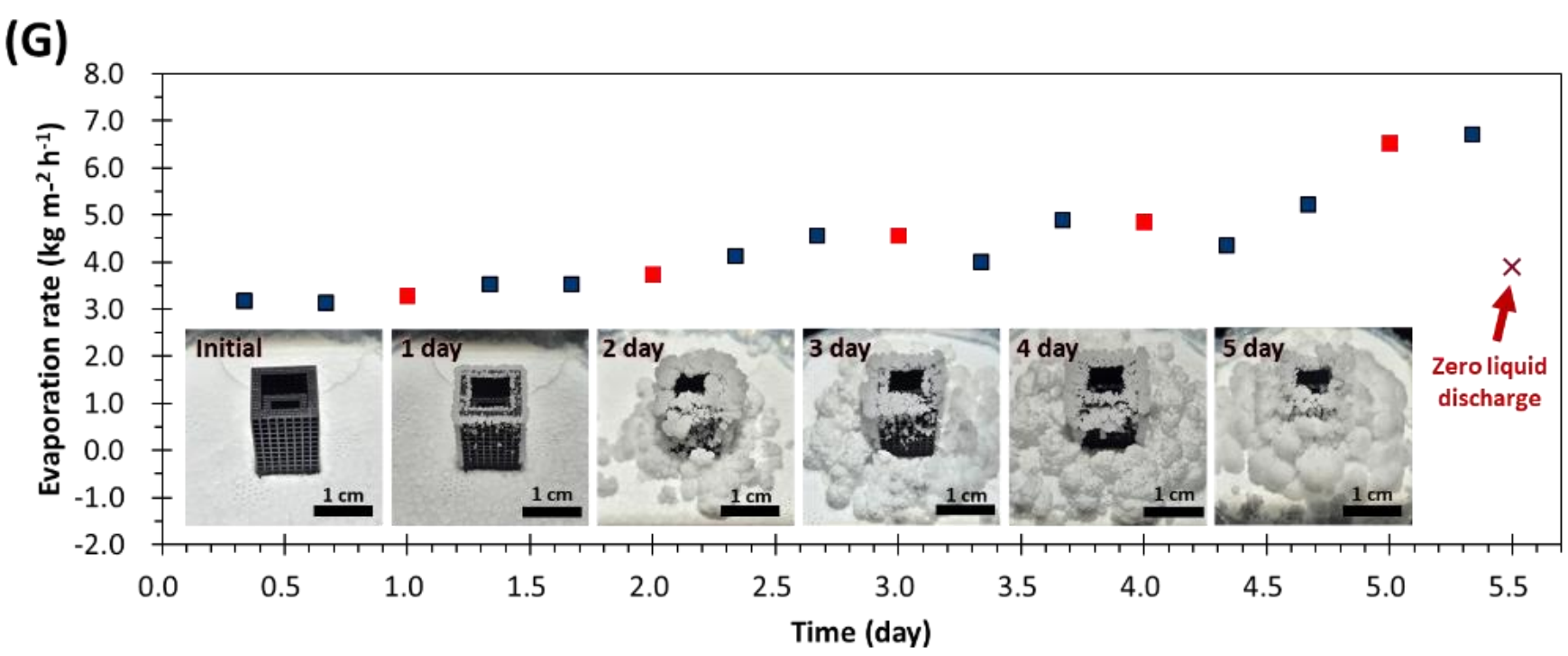

Figure 5. (G) Long-term stability under 20 wt% NaCl, with inset optical images showing localized salt accumulation corresponding to red dots.

Under a continuous-feed scenario, we agree that salt accumulation would eventually approach a critical volume requiring periodic removal. In such a case, the practical limit would be governed primarily by the available crystallization volume in the designated BCC region and the overall device geometry. In the current device geometry (1.32 × 1.32 cm lateral size and 2 cm height), the available salt-growth space is intentionally limited, and therefore a cleaning cycle would be triggered once the salt layer approaches a thickness that risks bridging or transport limitation. This represents an operational design parameter rather than a fundamental limitation of the localization mechanism.

We emphasize that the objective of this work is to demonstrate geometry-modulated salt localization using unit-cell-controlled evaporation asymmetry, rather than to fully optimize a continuous-feed crystallizer. The hybrid structure therefore serves as a proof-of-concept platform. For continuous operation, the operational window can be extended by increasing the volume of the designated crystallization region, modifying the outer geometry to accommodate outward salt growth, or implementing periodic passive salt shedding or washing cycles.

To clarify this point, we have revised the manuscript to explicitly distinguish between the ZLD-based operation demonstrated here and a continuous-feed scenario, and to discuss the mechanical limits of salt accumulation and the need for periodic regeneration in long-term operation.

On the revised manuscript; results

<Addition; text>

[Line 470-477] Under ZLD-type batch operation employed here, salt crystallization remained confined to the designated BCC region and grew outward without bridging to the FBCC-+ evaporation surface. Under continuous feed, salt accumulation would eventually reach a critical volume determined by the available crystallization space and device geometry. Although porous salt growth delays bridging and performance decay, regeneration would be required once the accumulated layer begins to impede vapor escape by extending toward the FBCC-+ region. This operational limit can be further extended through geometric optimization, such as increasing the designated crystallization volume.

Reviewer #5: I co-reviewed this manuscript with one of the reviewers who provided the listed reports. This is part of the Nature Communications initiative to facilitate training in peer review and to provide appropriate recognition for Early Career Researchers who co-review manuscripts.

Reply: We sincerely thank the reviewer for their time and contribution to the peer-review process. We appreciate the effort in carefully evaluating our work and supporting the rigor of the review. The comments associated with this co-review have been addressed in the corresponding responses.

References

1 Destyorini, F. *et al.* Formation of nanostructured graphitic carbon from coconut waste via low-temperature catalytic graphitisation. *Engineering Science and Technology, an International Journal* **24**, 514-523 (2021).

2 Wang, C., Zhang, H., Kang, Z. & Fan, J. 3D cellular solar crystallizer for stable and ultra-efficient high-salinity wastewater treatment. *Advanced Science* **11**, 2305313 (2024).

3 Choi, Y., Son, J. Y. & Kim, H. Geometry-Induced Capillary Rise and Directional Flow in Porous Lattice Structures. *ACS Applied Materials & Interfaces* (2026).

4 Zhang, Y. *et al.* Connected three-dimensional polyhedral frames for programmable liquid processing. *Nature Chemical Engineering* **1**, 472-482 (2024).

5 Xie, M. *et al.* Programmable microfluidics enabled by 3D printed bionic janus porous matrics for microfluidic logic chips. *Small* **19**, 2300047 (2023).

6 Li, L. *et al.* Long-term salt resistance in hypersaline, multi-ion brines via bionic solar evaporator based on thermal field optimization. *Applied Thermal Engineering*, 128057 (2025).

7 Dudukovic, N. A. *et al.* Cellular fluidics. *Nature* **595**, 58-65 (2021).

8 Li, S. *et al.* Bioinspired Integration of Cellular Microlattices and Asymmetric Re-Entrant Microstructures for Directional Capillary Reaction. *ACS Applied Materials & Interfaces* (2026).

9 Shen, J. & Guo, C. Bionic design and analysis of 3D-printed lattice structure wicks for heat pipe application. *Case Studies in Thermal Engineering* **69**, 105967 (2025).

10 Wu, S., Sun, S., Ye, J., Wang, L. & Zhang, Y. Capillary-Driven 3D Open Fluidic Networks for Versatile Continuous Flow Manipulation. *Advanced Materials*, 2503840 (2025).

11 Wu, X. *et al.* Capillary container array structures for efficient, energy-saving, and sustainable evaporative cooling and humidification. *Energy* **309**, 133159 (2024).

12 Yu, J. *et al.* Self-assembled porous salt crystals for solar-powered crystallization. *Energy & Environmental Science* **18**, 454-467 (2025).

13 Ding, M. *et al.* Bio-inspired solar evaporators for stable and efficient desalination of high-salinity brine with zero liquid discharge. *Science Bulletin* (2025).

14 Kim, J. *et al.* Interfacial solar evaporator-physical principles and fabrication methods. *International Journal of Precision Engineering and Manufacturing-Green Technology* **8**, 1347-1367 (2021).

15 Liu, Y., Li, Q., Li, Y., Feng, H. & Deng, R. Directional salt crystallization system for high-efficiency solar-driven evaporation to achieve zero-liquid discharge. *Separation and Purification Technology* **354**, 129412 (2025).

16 Ding, M., Duan, Z., Zhao, D., Liu, C. Y. & Li, C. Tree-inspired 3D biomimetic evaporator for efficient solar desalination and automated salt collection with zero liquid discharge. *Advanced Functional Materials* **36**, e12220 (2026).

17 Zhang, C. *et al.* Designing a next generation solar crystallizer for real seawater brine treatment with zero liquid discharge. *Nature Communications* **12**, 998 (2021).

18 Liang, H. *et al.* Thermal efficiency of solar steam generation approaching 100% through capillary water transport. *Angewandte Chemie International Edition* **58**, 19041-19046 (2019).

19 Ma, X., Jia, X., Yao, G. & Wen, D. Umbrella evaporator for continuous solar vapor generation and salt harvesting from seawater. *Cell Reports Physical Science* **3** (2022).

20 Wu, L. *et al.* Highly efficient three-dimensional solar evaporator for high salinity desalination by localized crystallization. *Nature Communications* **11**, 521 (2020).

21 Li, Y. *et al.* Pyramid-shaped solar evaporator with high-efficient interfacial evaporation and salt harvesting capability. *Journal of Cleaner Production* **434**, 139956 (2024).

22 Gao, T. *et al.* More from less: improving solar steam generation by selectively removing a portion of evaporation surface. *Science bulletin* **67**, 1572-1580 (2022).

23 Li, X. *et al.* Less is more: reducing evaporation dead zones to tune thermal convection in donut-structure evaporators for high-efficiency interfacial solar steam generation. *Advanced Functional Materials* **35**, 2504502 (2025).
24 Wang, D. *et al.* Dyson sphere-like evaporators enhanced interfacial solar evaporation via self-generated internal convection. *Nature Communications* **16**, 7985 (2025).
25 Meng, F. *et al.* N-doped carbon@ Cu core–shell nanostructure with nearly full solar spectrum absorption and enhanced solar evaporation efficiency. *Journal of Materials Chemistry A* **10**, 9575-9581 (2022).
26 Zhang, M. *et al.* Surface Roughness Engineering for High-Salt-Tolerant Solar Evaporator with Enhanced Efficiency in Sustainable Desalination. *ChemSusChem* **18**, e202500714 (2025).
27 Qiu, C., Jiang, L., Gao, Y. & Sheng, L. Effects of Oxygen-Containing Functional Groups on Carbon Materials in Supercapacitors: A Review. *Materials & Design*, 111952 (2023).
28 Luo, L. *et al.* Preparation of graphite oxide containing different oxygen-containing functional groups and the study of ammonia gas sensitivity. *Sensors* **18**, 3745 (2018).
29 Dai, C. *et al.* Strategic design of porous interfacial evaporators: A comprehensive review unveiling the significant role of pore engineering. *Nano Energy* **131**, 110244 (2024).
30 Yan, L. *et al.* PC@ PPy porous membrane prepared by breath figure method with superior mechanical property for efficient solar interfacial evaporation. *Chemical Engineering Journal* **469**, 144059 (2023).
31 Zhu, J. *et al.* FDM 3D-printed volcanic-shaped structure for ultrafast solar-driven interfacial evaporation and efficient energy utilization. *Desalination* **548**, 116275 (2023).
32 Tang, Y. *et al.* A 3D-printed hierarchical chimney for high-yield solar evaporation. *Energy & Environmental Science* **18**, 8220-8231 (2025).
33 Chen, D. *et al.* A Self-Floating Solar Evaporator Based on Carbon Black/Polydimethylsiloxane for Highly Efficient and Stable Desalination. *ACS Applied Materials & Interfaces* (2025).

Response to the reviewer's comments

We sincerely appreciate the reviewer's effort for careful and valuable comments on this manuscript. The insightful feedback you provided has significantly contributed to improve our manuscript. We have thoroughly reviewed and incorporated your comments into our work. Each of your points has been addressed, and the corresponding parts were highlighted in the revised manuscript.

Reviewer #1: I have carefully reviewed the revised manuscript and the authors' point-by-point response to the initial round of reviews. The authors have provided substantial new experimental data and analysis that significantly strengthen the study's conclusions and overall impact. Nevertheless, some issues are still needed to be considered before it can be published on *Nature Communications*.

1. The manuscript enhanced capillary force and water transport capacity through its printed structure. However, excessive water transport might lead to the excessive water required at the evaporation interface, which in turn affected the evaporation efficiency. Therefore, achieving a proper balance in this aspect was of utmost importance. It is recommended to solve this problem through experiments or simulations.

Reply: We sincerely thank the reviewer for this insightful comment. We fully agree that achieving an appropriate balance between capillary water supply and interfacial evaporation demand is critical for efficient solar evaporation. Excessive water transport could potentially cause liquid accumulation at the evaporation interface, formation of a continuous water film, and additional heat loss associated with bulk water heating, which may adversely affect evaporation efficiency.

In our 3D-printed multicellular evaporators, however, the enhanced capillary transport does not result in uncontrolled water oversupply or flooding of the evaporation interface. The transported water is distributed through the interconnected open-pore network, and once it reaches the evaporative region, it forms discrete air–water interfaces within the pores rather than accumulating as a continuous bulk liquid layer on the external surface. During the evaporation experiments, no visible water pooling, dripping, or overflow was observed. Moreover, the evaporation rate remained stable during prolonged operation, indicating that the capillary supply continuously replenished water to the evaporative sites without impairing evaporation by excessive liquid accumulation.

To further investigate this behavior, we analyzed X-ray micro-computed tomography images of the wetted FBCC-+ structure, which exhibited the most effective capillary-driven water transport. The X-ray micro-CT images show that the liquid phase is confined within the internal pore network and forms stable menisci at the pore openings. Importantly, these menisci remain pinned at the pore boundaries rather than overflowing over the external surface of the lattice. This observation supports that the pore geometry regulates liquid distribution by maintaining wet evaporative interfaces while preventing the formation of a continuous external water layer.

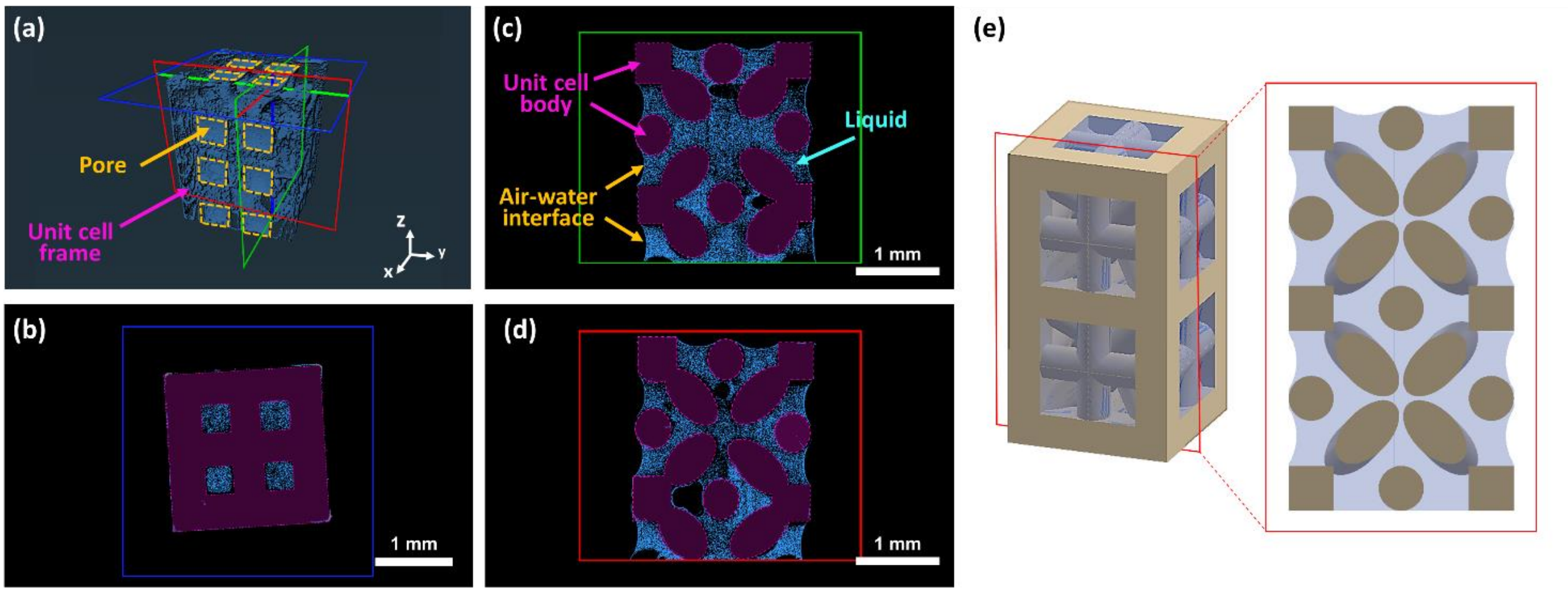

Figure R1. X-ray μ-CT images of the FBCC-+ structure fully saturated with water. (a) Three-dimensional reconstruction, and cross-sectional views in the (b) xy-, (c) xz-, and (d) yz-planes, showing the air–water interface pinned at the evaporative surface. (e) Schematic illustration of the water-filled FBCC-+ structure and its corresponding cross-sectional view to facilitate visualization of the liquid distribution and interfacial configuration.

This behavior can be attributed to the hydrophilic nature of the evaporator surface. Because the fabricated structures are hydrophilic (contact angle = 44.7°), water forms a concave meniscus at the pore openings. The resulting capillary pressure lowers the liquid pressure relative to the surrounding air phase, stabilizing the liquid within the porous network. Consequently, the menisci remain pinned at the pore boundaries, preventing the supplied water from spreading over the external surface as a continuous water film. Instead, the water is retained as localized evaporative interfaces distributed throughout the pore network.

Therefore, the pore geometry and surface wettability work synergistically to regulate liquid distribution, maintaining wet evaporative interfaces while preventing flooding of the external surface. This meniscus-pinning mechanism provides an additional means by which the multicellular lattice balances capillary water supply with interfacial evaporation demand. Together, the stable evaporation performance and direct micro-CT observations confirm that

the enhanced capillary transport in the present architecture promotes efficient and sustained water replenishment without causing excessive liquid accumulation that would otherwise reduce evaporation efficiency.

2. As shown in Figure 4D, the printed device significantly improved the evaporation efficiency by increasing the height. But what would happen if the height exceeded 5 cm?

Reply: We sincerely thank the reviewer for this insightful comment. We agree that understanding the effect of further increasing the evaporator height is important, as the height-dependent enhancement in evaporation performance is one of the key findings of this work.

To address this point, we conducted an additional evaporation experiment using an FBCC-+ multicellular solar evaporator with an exposed height exceeding 5 cm. When the exposed height was increased to 6 cm, the evaporation rate decreased compared with that of the 5 cm structure. Specifically, the evaporation rate of FBCC-+_6 was 6.30 ± 0.65 kg m^{-2} h^{-1}, lower than that of FBCC-+_5 (6.90 ± 0.07 kg m^{-2} h^{-1}). These results indicate that the evaporation enhancement does not increase indefinitely with height and that an optimal height exists under the present design conditions.

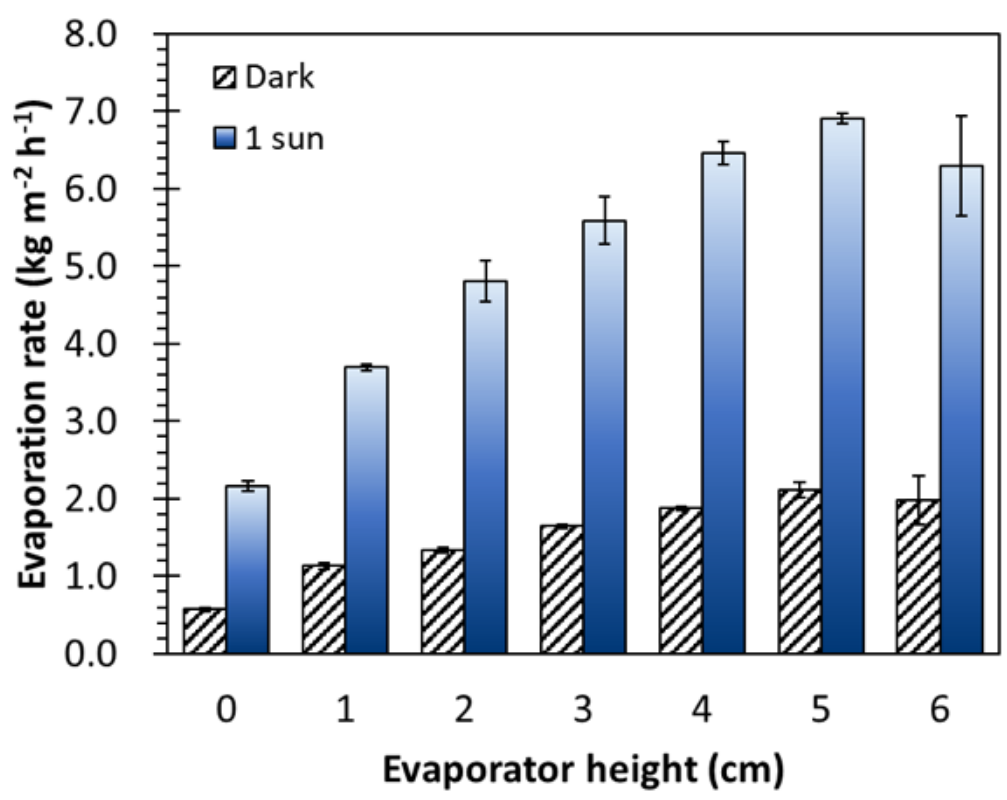

Figure 4. (D) Height-dependent evaporation rate of FBCC-+ MSE.

The observed decrease at 6 cm is attributed to the increasing transport resistance associated with the longer capillary transport pathway. While increasing the exposed height enlarges the lateral evaporative surface area, it also increases the distance over which water must be transported to replenish the evaporative interfaces. Consequently, the capillary pressure available to sustain water delivery becomes progressively offset by gravitational and viscous losses, reducing the efficiency of liquid replenishment at the upper regions of the

structure. As a result, the gain in evaporation area no longer compensates for the reduced water supply efficiency, leading to a decline in the overall evaporation rate.

It should also be noted that the optimal height identified here is not a universal value, but rather depends on the specific evaporator geometry. In open-cell lattice structures, capillary transport is governed by the balance between capillary forces and gravity, which is influenced by parameters such as porosity, strut/frame dimensions, liquid–solid contact perimeter, porosity, unit cell array configuration and meniscus curvature[1,2]. Therefore, the transition observed above 5 cm should be understood as geometry-dependent, and different lattice architectures or device dimensions may exhibit different optimal heights. In particular, the evaporator height and bottom area are coupled design parameters, and their combined optimization may shift the optimum operating height for a given system.

Overall, these results demonstrate that evaporation enhancement through increasing height is subject to diminishing returns and ultimately becomes limited by water transport. Within the investigated range, a height of 5 cm provides the most favorable balance between evaporative area and capillary water supply.

We have included these additional experimental results in the revised manuscript, together with the corresponding analysis of the apparent evaporation efficiency and lateral evaporation contribution.

On the revised manuscript; results

<From>

[Line 325-326] Accordingly, the evaporation rate increased from 2.16 ± 0.06 kg m-2 h-1 at 0 cm height to 6.90 ± 0.07 kg m-2 h-1 for the 5 cm configuration, denoted as FBCC-+_5 (Figure 4D).

<To>

[Line 328-335] Accordingly, the evaporation rate increased from 2.16 ± 0.06 kg m^{-2} h^{-1} at 0 cm height to 6.90 ± 0.07 kg m^{-2} h^{-1} for the 5 cm configuration, denoted as FBCC-+_5 (Figure 4D). However, a further increase to 6 cm reduced the evaporation rate to 6.30 ± 0.65 kg m^{-2} h^{-1}, indicating the existence of an optimal height under the present design conditions. This decline is attributed to increased capillary transport resistance associated with the longer water transport pathway, which ultimately outweighs the benefit of the additional evaporative area. As FBCC-+_5 exhibited the highest evaporation performance among all tested configurations, it was selected as the representative geometry for subsequent salinity, crystallization, and long-term stability studies.

<From>

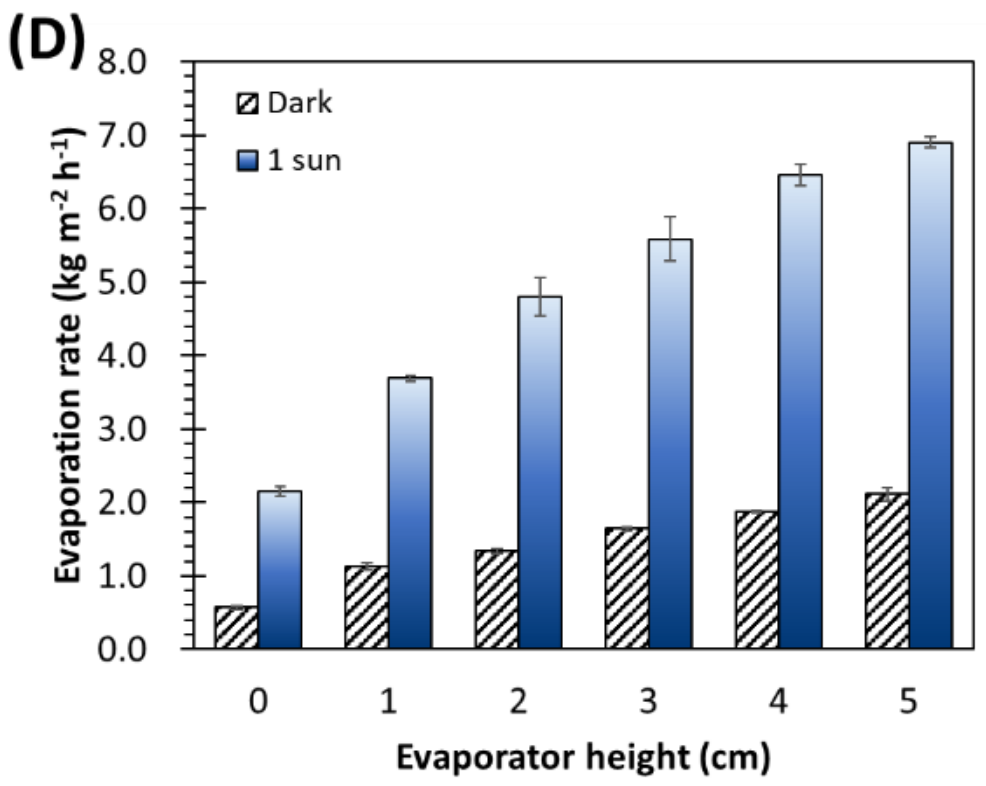

Figure 4. (D) Height-dependent evaporation rate of FBCC-+ MSE.

<To>

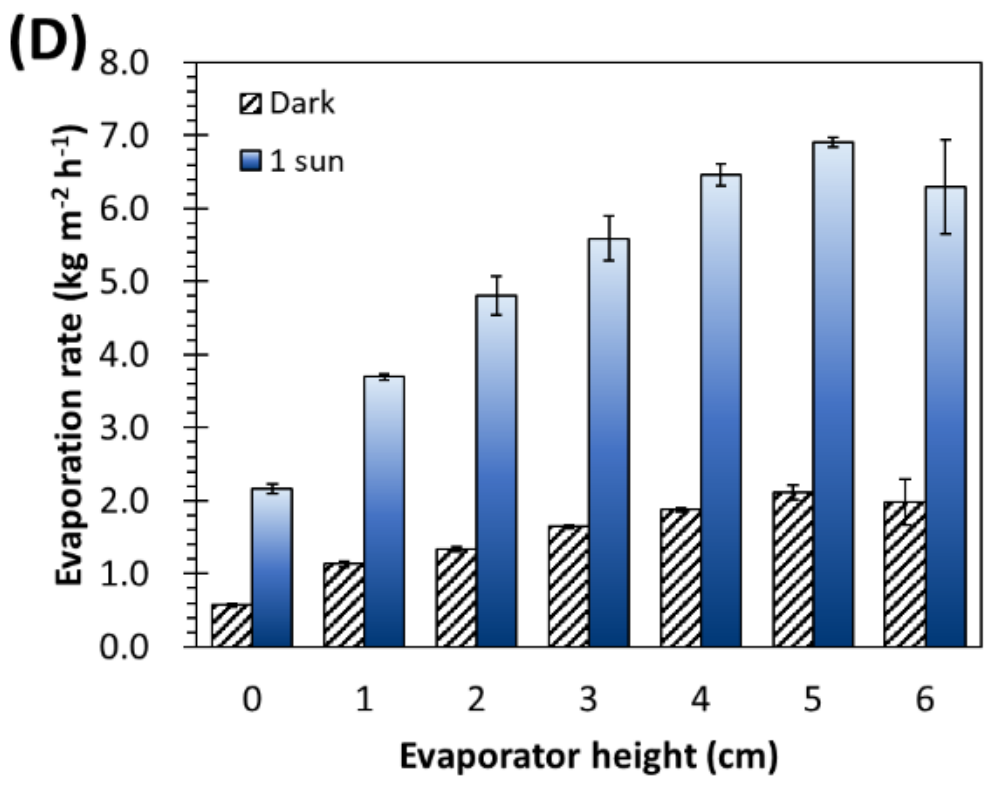

Figure 4. (D) Height-dependent evaporation rate of FBCC-+ MSE.

On the revised supplementary materials

<From>

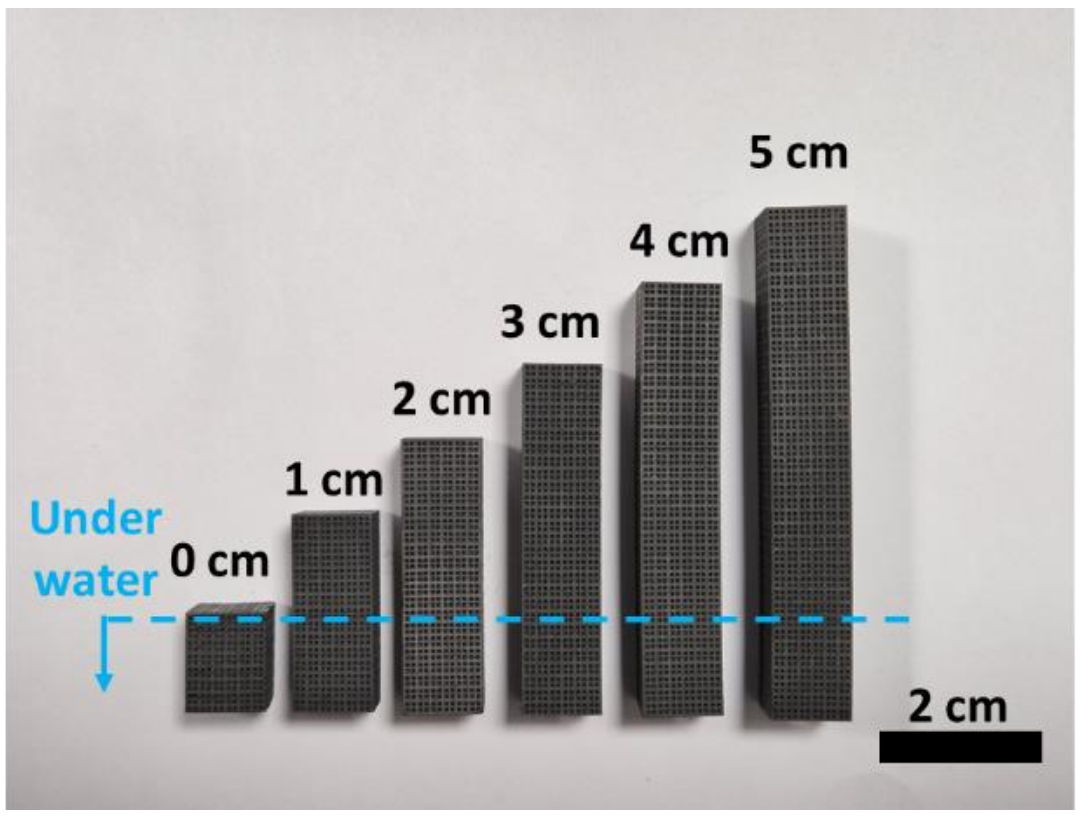

Figure S17. Optical images of FBCC-+ evaporators with varying heights.

<To>

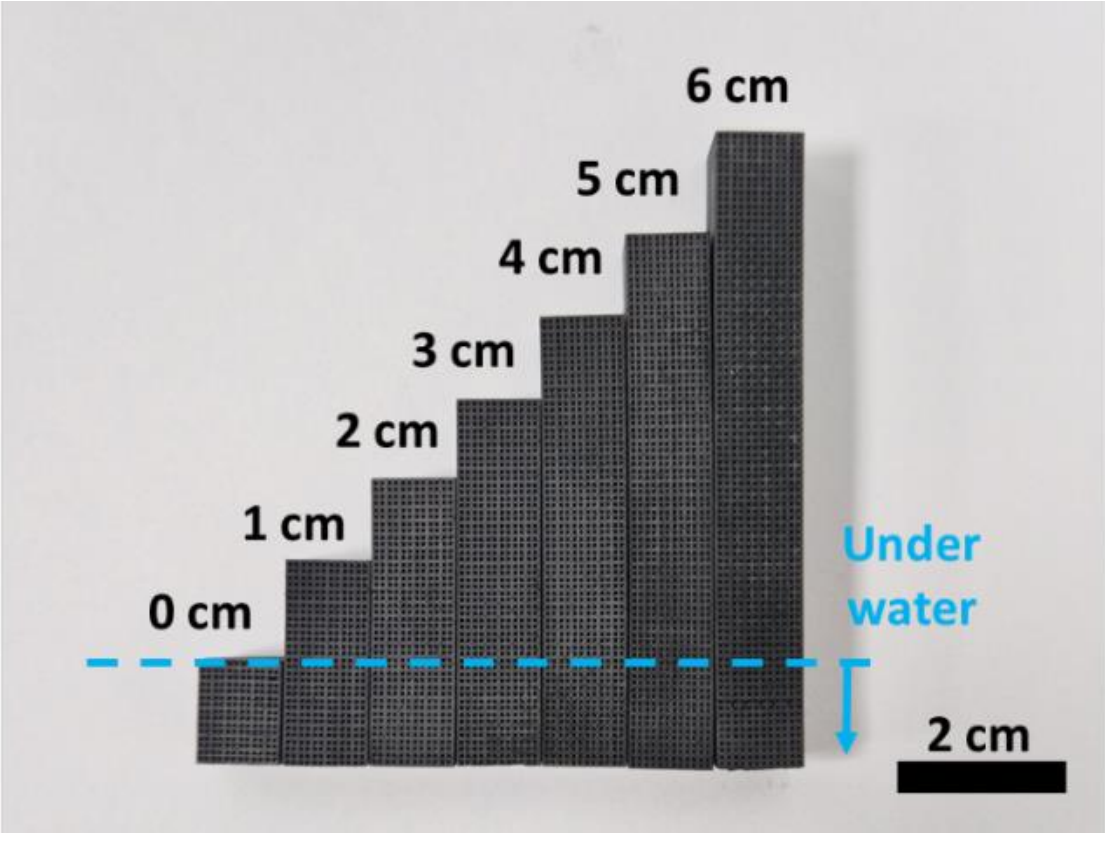

Figure S17. Optical images of FBCC-+ evaporators with varying heights.

<From>

Table S7. Geometric parameters of FBCC-+ MSEs at different exposed height.

MSE configuration	Dimensions* (*W* × *L* × *H*, cells)	Dimensions* (*W* × *L* × *H*, cm)	Light illuminated area (cm^2)
FBCC-+_0	6 × 6 × 0	1 × 1 × 0	1
FBCC-+_1	6 × 6 × 6	1 × 1 × 1	1
FBCC-+_2	6 × 6 × 12	1 × 1 × 2	1
FBCC-+_3	6 × 6 × 18	1 × 1 × 3	1
FBCC-+_4	6 × 6 × 24	1 × 1 × 4	1
FBCC-+_5	6 × 6 × 30	1 × 1 × 5	1

*Exposed height refers to the portion of the structure extending above the water reservoir.

<To>

Table S7. Geometric parameters of FBCC-+ MSEs at different exposed height.

MSE configuration	Dimensions* (*W* × *L* × *H*, cells)	Dimensions* (*W* × *L* × *H*, cm)	Light illuminated area (cm^2)
FBCC-+_0	6 × 6 × 0	1 × 1 × 0	1
FBCC-+_1	6 × 6 × 6	1 × 1 × 1	1
FBCC-+_2	6 × 6 × 12	1 × 1 × 2	1
FBCC-+_3	6 × 6 × 18	1 × 1 × 3	1
FBCC-+_4	6 × 6 × 24	1 × 1 × 4	1
FBCC-+_5	6 × 6 × 30	1 × 1 × 5	1
FBCC-+_6	6 × 6 × 36	1 × 1 × 6	1

*Exposed height refers to the portion of the structure extending above the water reservoir.

<From>

Table S8. Lateral surface contribution to evaporation performance of MSEs.

MSE configuration	Top surface area (cm^2)	Lateral surface area (cm^2)	ER_{total}* ($kg\ m^{-2}\ h^{-1}$)	$ER_{lateral}$** ($kg\ m^{-2}\ h^{-1}$)	Lateral contribution*** (%)
FBCC-+_0	1	0	2.16	-	-
FBCC-+_1	1	4	3.69	1.53	41.46
FBCC-+_2	1	8	4.80	2.64	55.00
FBCC-+_3	1	12	5.59	3.43	61.36
FBCC-+_4	1	16	6.46	4.30	66.56
FBCC-+_5	1	20	6.90	4.74	68.70

*Total evaporation rate.

**Evaporation rate contributed by lateral surfaces, calculated as $ER_{lateral} = ER_{total} - ER_{0cm}$

***Lateral contribution $= \frac{ER_{lateral}}{ER_{total}} \times 100$

<To>

Table S8. Lateral surface contribution to evaporation performance of MSEs.

MSE configuration	Top surface area (cm^2)	Lateral surface area (cm^2)	ER_{total}* ($kg\ m^{-2}\ h^{-1}$)	$ER_{lateral}$** ($kg\ m^{-2}\ h^{-1}$)	Lateral contribution*** (%)
FBCC-+_0	1	0	2.16	-	-
FBCC-+_1	1	4	3.69	1.53	41.46
FBCC-+_2	1	8	4.80	2.64	55.00
FBCC-+_3	1	12	5.59	3.43	61.36
FBCC-+_4	1	16	6.46	4.30	66.56
FBCC-+_5	1	20	6.90	4.74	68.70
FBCC-+_6	1	24	6.30	4.14	65.69

*Total evaporation rate.

**Evaporation rate contributed by lateral surfaces, calculated as $ER_{lateral} = ER_{total} - ER_{0cm}$

***Lateral contribution $= \frac{ER_{lateral}}{ER_{total}} \times 100$

<From>

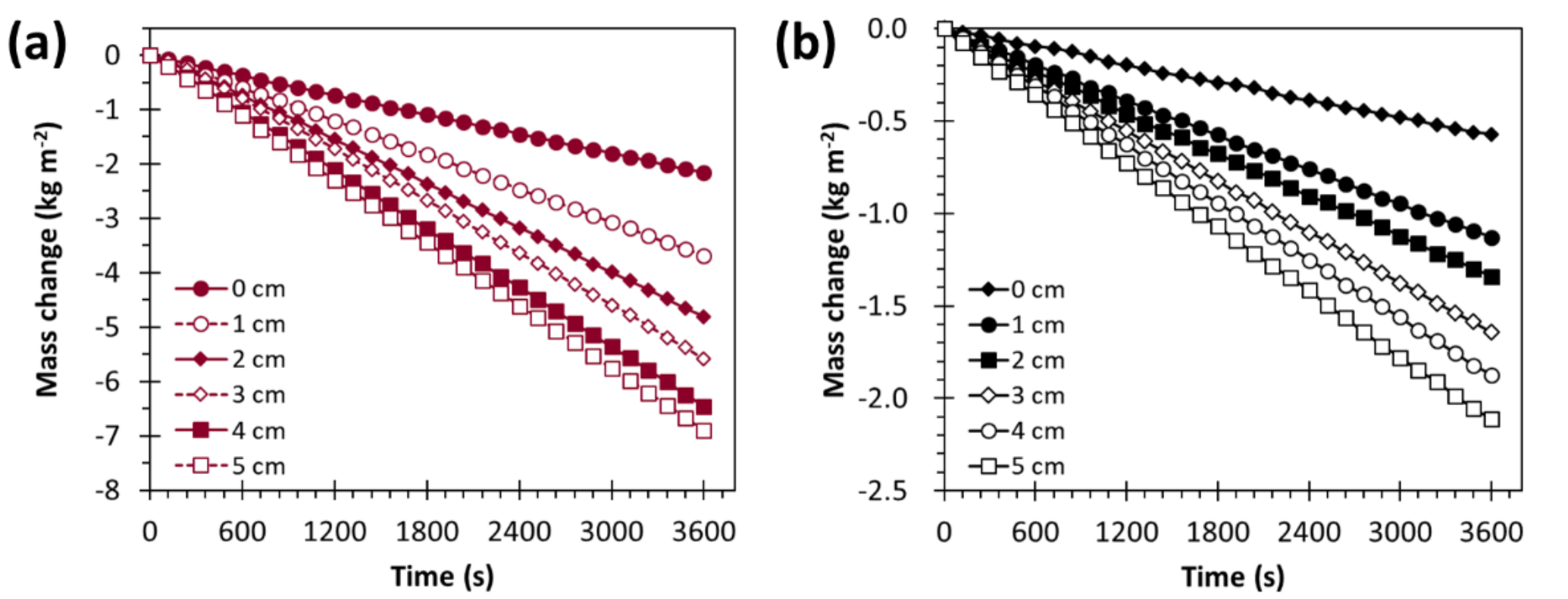

Figure S18. Mass change during evaporation at controlled exposed height. (a) Under 1 sun illumination and (b) under dark condition.

<To>

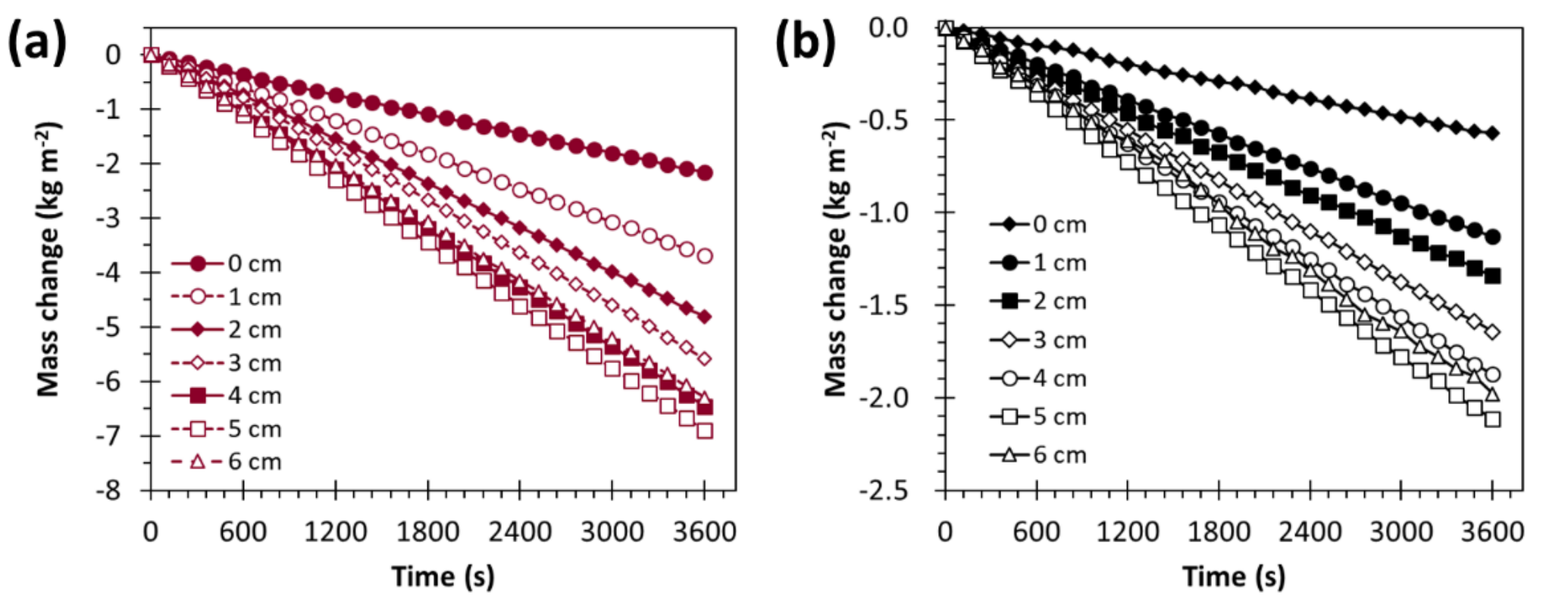

Figure S18. Mass change during evaporation at controlled exposed height. (a) Under 1 sun illumination and (b) under dark condition.

<From>

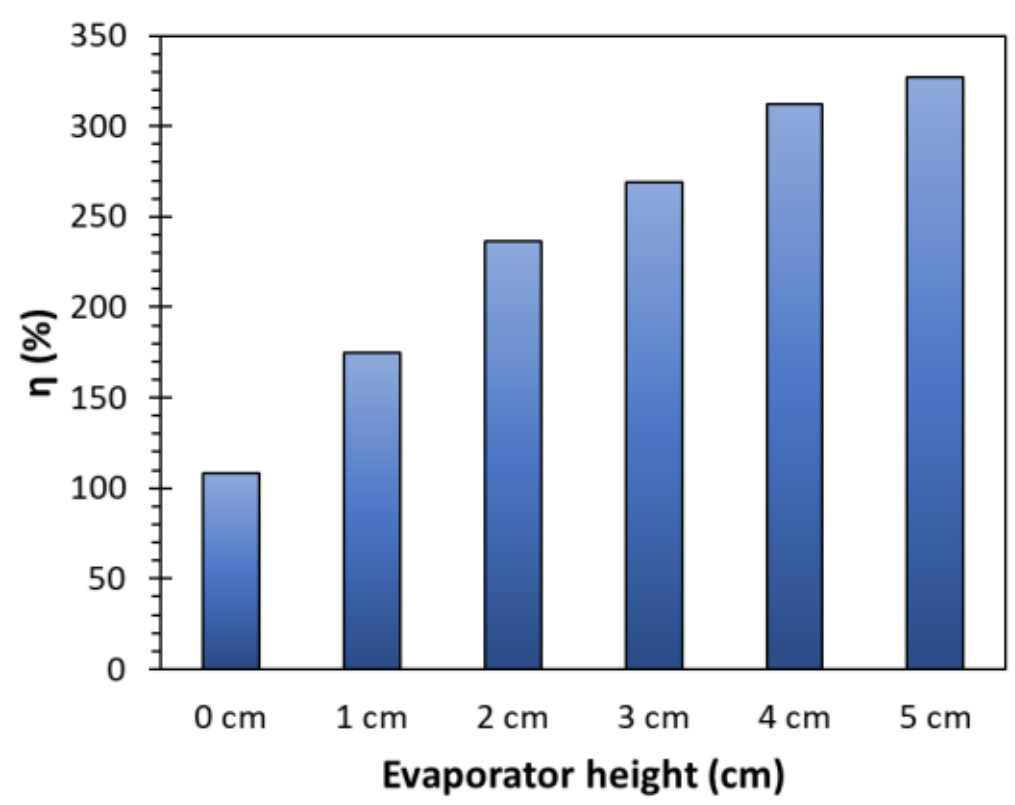

Figure S20. Apparent energy utilization efficiency of FBCC-+ evaporators with different heights.

<To>

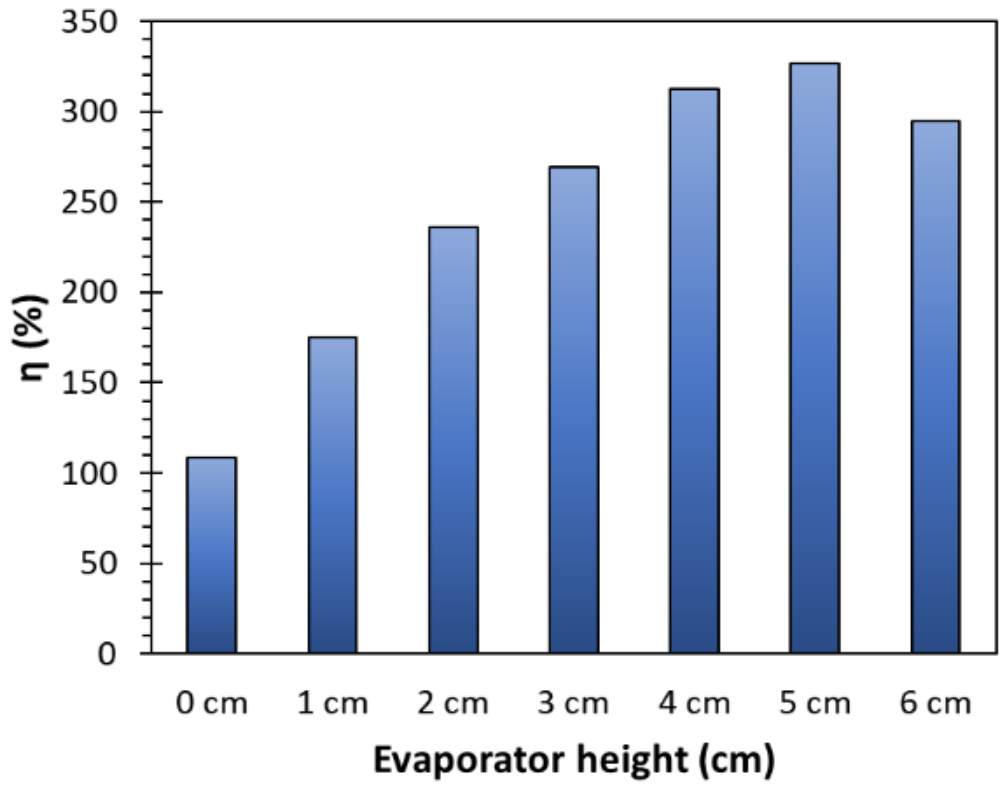

Figure S20. Apparent energy utilization efficiency of FBCC-+ evaporators with different heights.

3. Because the height of the evaporation device also had a significant impact on the evaporation efficiency, therefore, when comparing with the efficiency of other tasks, it is recommended to include the height parameter of the devices.

Reply: Thank you for this valuable suggestion. We agree that the height of the evaporator is an important geometrical parameter that can significantly influence the evaporation performance, particularly for 3D evaporators where the lateral surfaces contribute to additional evaporation and energy harvesting from the surrounding environment.

Following the reviewer's suggestion, we have revised the comparison table to include the geometrical dimensions of the reported 3D-printed solar evaporators, including the illuminated area and device height, wherever such information was available. This additional information allows a more transparent comparison of evaporation performance among different studies.

We note that the reported solar evaporators exhibit considerable variation in geometry, including differences in projected area, exposed height, and overall structural design. Consequently, direct comparison based solely on evaporation rate may not fully capture the influence of device architecture. To provide additional context, both the illuminated area and height have therefore been incorporated into the revised table, allowing the reported performances to be interpreted alongside the corresponding geometric characteristics.

For studies in which the device height or geometric dimensions were not explicitly reported, the corresponding entries have been marked as "Not reported". In cases where the illuminated area was not directly provided but could be determined from the reported

dimensions (e.g., device diameter or lateral length), the area was calculated accordingly, and this procedure has been clarified in the table footnote.

We believe that these revisions improve the completeness of the comparison and provide readers with a more informative assessment of the performance of previously reported 3D solar evaporators relative to the present work.

On the revised supplementary materials

<From>

Table S12. Comparison of recent resin- and filament-based 3D-printed solar evaporators.

Base material	Fabrication method	Evaporation rate* (kg m^{-2} h^{-1})	Water yield** (kg m^{-2} day^{-1})	Ref
Acryl resin	DLP 3D printing	3.5	34.4	[15]
Acryl resin	DLP 3D printing	2.32	14.8	[16]
Acryl resin	DLP 3D printing	1.64	2.5	[17]
Resin	DLP 3D printing	2.63	15.48	[10]
Resin	DLP 3D printing	3.18	20.52	[18]
Acryl resin	DLP 3D printing	2.13	9.95	[19]
Homemade resin	DLP 3D printing	2.249	12.23	[20]
Acryl resin	PμSL 3D printing	5.03	35.1	[21]
Filament	FDM 3D printing	4.02	22.27	[13]
Filament	FDM 3D printing	3.54	24.93	[12]
Resin	DLP 3D printing	4.08	15.1	[22]
Acryl resin	DLP 3D printing	6.9	47.68	This study

*Under constant 1 sun illumination, **Outdoor condition

DLP: Digital Light Processing

FDM: Fused Deposition Modeling

PμSL: Projection Micro-Stereolithography

<To>

Table S12. Comparison of recent resin- and filament-based 3D-printed solar evaporators.

Base material	Fabrication method	Dimensions (area × height, cm^2 × cm)	Evaporation rate* ($kg\ m^{-2}\ h^{-1}$)	Water yield** ($kg\ m^{-2}\ day^{-1}$)	Ref
Acryl resin	DLP 3D printing	4 × 5	3.5	34.4	15
Acryl resin	DLP 3D printing	0.13 × 1.5	2.32	14.8	16
Acryl resin	DLP 3D printing	Not reported	1.64	2.5	17
Resin	DLP 3D printing	0.95 × 2.2	2.63	15.48	10
Resin	DLP 3D printing	1 × 1.2	3.18	20.52	18
Acryl resin	DLP 3D printing	7.07 × 1.75	2.13	9.95	19
Homemade resin	DLP 3D printing	3.14 × 2	2.249	12.23	20
Acryl resin	PμSL 3D printing	0.62 × 0.6	5.03	35.1	21
Filament	FDM 3D printing	1.77 × 1.5	4.02	22.27	13
Filament	FDM 3D printing	1.30 × 2	3.54	24.93	12
Resin	DLP 3D printing	7.07 × 3	4.08	15.1	22
Acryl resin	DLP 3D printing	1 × 5	6.9	47.68	This study

*Under constant 1 sun illumination, **Outdoor condition

DLP: Digital Light Processing

FDM: Fused Deposition Modeling

PμSL: Projection Micro-Stereolithography

“Not reported” indicates that the specific dimension was not provided in the original reference. When the illuminated area was not explicitly reported, it was calculated based on the available geometrical dimensions, such as the lateral length or diameter of the evaporator.

Reviewer #2: The authors have addressed what I am concerned about. Agree to accept.

Reply: Thank you for your insightful comments and thoughtful evaluation of our work. We sincerely appreciate your constructive feedback throughout the review process, which has been invaluable in improving the quality and clarity of our manuscript. We are grateful for your recommendation for acceptance and are pleased that the revisions have addressed your concerns.

Reviewer #3: The authors have made good revisions to address the issues raised, and I believe this version of the paper is worth publishing.

Reply: We sincerely thank the reviewer for the thoughtful assessment of our revised manuscript. We greatly appreciate your recognition of the revisions made in response to the previously raised issues. Your constructive comments have helped us improve the clarity and quality of the manuscript, and we are grateful for your support regarding its publication.

Reviewer #4: The authors have sufficiently addressed my comments and the work is technically accurate. They have also made modifications in response to all the reviewers' comments and the manuscript reads well.

Reply: We sincerely appreciate the reviewer's careful evaluation of our revised manuscript. We are grateful for your acknowledgement that the comments have been sufficiently addressed and that the work is technically accurate. Your constructive feedback throughout the review process has been highly valuable in strengthening the manuscript.

Additional comment from Reviewer #4 communicated by the Editor:

Reviewer #4 noted that the performance and scalability of the present system are not necessarily superior to the state of the art. The Editor requested to include a short discussion on the limitations of the work and potential future strategies.

Reply: We thank reviewer and the editor for this constructive suggestion.

In response, we have revised the Discussion section to more explicitly acknowledge the limitations associated with scaling the present system and to highlight potential directions for future development. While the primary objective of this work is to establish geometry–performance relationships in multicellular solar evaporators, we agree that practical implementation requires consideration of system-level scalability in addition to device-level performance.

Accordingly, we have added a discussion clarifying that, although the periodic lattice architecture provides a potential pathway toward larger-area implementation through modular replication, further advances in manufacturing scalability and system-level optimization will be required to maximize practical water production. Specifically, we now discuss the importance of factors such as evaporator spacing, solar concentration, and thermal and mass-transfer management in future large-scale systems.

These additions have been incorporated into the revised manuscript and provide a more balanced perspective on both the opportunities and the current limitations of the proposed geometry-driven design framework.

On the revised manuscript; discussion

<From>

[Line 572-580] Collectively, these results demonstrate that unit cell and macro-scale geometry can be used as programmable design variables to control interfacial transport, heat localization, and salt deposition pathways in solar evaporators. The structure–function relationships identified here suggest general design principles under controlled material and wettability conditions. While these geometric effects are expected to be broadly applicable, the absolute performance may depend on material-specific properties, including wettability, optical absorption, and photothermal conversion efficiency. This work thus establishes a geometry-driven framework for designing scalable, durable, and high-performance solar evaporation systems, advancing solar-driven desalination and ZLD toward real-world implementation.

<To>

[Line 582-594] Collectively, these results demonstrate that unit cell and macro-scale geometry can be used as programmable design variables to control interfacial transport, heat localization, and salt deposition pathways in solar evaporators. The structure–function relationships identified here suggest general design principles under controlled material and wettability conditions. While these geometric effects are expected to be broadly applicable, the absolute performance may depend on material-specific properties, including wettability, optical absorption, and photothermal conversion efficiency. Although the periodic lattice design provides a clear route toward larger-area implementation through modular replication, further advances in manufacturing scalability and system-level optimization, including evaporator spacing, solar concentration, thermal and mass-transfer management, will be required to maximize practical water production. This work thus establishes a geometry-driven framework for designing scalable, durable, and high-performance solar evaporation systems, advancing solar-driven desalination and ZLD toward real-world implementation.

Additional revisions

In addition to the revisions made in direct response to the reviewers' comments, we have carefully reviewed the manuscript in its entirety and implemented several minor edits to further improve clarity, readability, and consistency. These revisions include refinement of the text, correction of typographical and formatting errors where appropriate. We have also revised several passages to more clearly highlight the motivation, key findings, and broader significance of the present work.

We hope that these revisions further improve the overall presentation of the manuscript.

On the revised manuscript; abstract

<From>

[Line 40-41] These results establish unit cell and macro-scale geometry as programmable design parameters for scalable and durable solar desalination.

<To>

[Line 39-42] Overall, this work reveals geometry–transport–performance relationships in 3D-printed multicellular solar evaporators and establishes unit-cell and macro-scale geometry as programmable design parameters for scalable and durable solar desalination.

On the revised manuscript; introduction

<From>

[Line 49-50] …complex infrastructure, and high operational cost …

<To>

[Line 50-51] … complex infrastructure, and costly operation …

<From>

[Line 76-80] However, most reported evaporators rely on randomly porous structures, making it difficult to systematically correlate structural parameters with heat and mass transport. As a result, prior studies often address individual factors in isolation, limiting the identification of architectures that can simultaneously satisfy multiple performance criteria.

<To>

[Line 76-80] However, most reported evaporators rely on random porous structures, making it difficult to systematically correlate structural parameters with heat and mass transport. As a result, prior studies often addressed individual factors in isolation, limiting the identification of architectures that can simultaneously satisfy multiple performance criteria.

<From>

[Line 85] … offering a rational route to establish geometry–property–performance …

<To>

[Line 85] … offering a rational route to establish geometry–transport–performance …

<From>
[Line 98] … geometry-assisted harvesting of environmental thermal energy …
<To>
[Line 98] … geometry-assisted harvesting of ambient thermal energy …

<From>
[Line 160] … photothermal conversion efficiency of the MSE after graphite coating.
<To>
[Line 160] … photothermal conversion performance of the MSE after graphite coating.

<From>
[Line 230-232] The experiments confirm the geometric trend, with the FBCC-+ architecture exhibits both the fastest rise kinetics and the highest equilibrium water elevation within 15 min of wicking
<To>
[Line 230-232] The experiments confirmed the geometric trend, with the FBCC-+ architecture exhibiting both the fastest rise kinetics and the highest equilibrium water elevation within 15 min of wicking

<From>
[Line 239-241] To further isolate geometric influences by different unit cell types, water transport was additionally examined within a single topology using the FBCC-+ unit cell as a representative structure.
<To>
[Line 241-242] To further isolate geometric influences within a single unit cell topology, water transport was additionally examined using the FBCC-+ unit cell as a representative structure.

<From>
[Line 267] … exceeding that of bulk water and followed the same structural trend …
<To>
[Line 269] … which exceeded that of bulk water and followed the same structural trend …

<From>
[Line 290] … calculated effective thermal transport areas show strong correlation …
<To>
[Line 290] … calculated effective thermal transfer areas showed a strong correlation …

<From>
[Line 404-405] … whereas FBCC-+ showed the highest performance (Section 3.3, Figure 4B) …
<To>
[Line 414-415] … whereas FBCC-+ showed the highest performance (Section 2.3, Figure 4B) …

<From>

[Line 496-497] … to suppress unintended solar absorption and evaporation outside the evaporator region.…

<To>

[Line 507-507] … to suppress unintended solar absorption and evaporation from the regions outside the evaporator region …

<From>

[Line 499] … and closely correlated with …

<To>

[Line 520] … which were closely correlated with …

<From>

[Line 526-528] While many prior systems improve either evaporation rate or freshwater yield, the present multicellular evaporator achieves substantial enhancement in both metrics simultaneously

<To>

[Line 536-538] While many prior systems improved either evaporation rate or freshwater yield, the present multicellular evaporator achieved substantial enhancement in both metrics simultaneously

<From>

[Line 659] … water or NaCl solutions with 3.5 wt%, 10 wt%, …

<To>

[Line 673] … water or NaCl solutions at concentrations of 3.5 wt%, 10 wt%, …

References

1 Dudukovic, N. A. *et al.* Cellular fluidics. *Nature* **595**, 58-65 (2021).

2 Choi, Y., Son, J. Y. & Kim, H. Geometry-Induced Capillary Rise and Directional Flow in Porous Lattice Structures. *ACS Applied Materials & Interfaces* **18**, 21212-21224 (2026).

Response to the reviewer's comments

Reviewer #1: The authors have made relevant revision and it can be considered for publication on *Nature Communications*.

Reply: We sincerely thank the reviewer for the thoughtful assessment of our revised manuscript. We greatly appreciate your acknowledgement that the revisions have adequately addressed the previously raised concerns and that the manuscript can now be considered for publication in *Nature Communications*. Your constructive comments throughout the review process have been highly valuable in improving the clarity and quality of our work.
